# EWS and FUS bind a subset of transcribed genes encoding proteins enriched in RNA regulatory functions

**DOI:** 10.1186/s12864-015-2125-9

**Published:** 2015-11-14

**Authors:** Yonglun Luo, Jenny Blechingberg, Ana Miguel Fernandes, Shengting Li, Tue Fryland, Anders D. Børglum, Lars Bolund, Anders Lade Nielsen

**Affiliations:** Department of Biomedicine, Aarhus University, The Bartholin Building, Aarhus, DK-8000 Denmark; Center for Integrative Sequencing, iSEQ, Aarhus University, Aarhus, Denmark; Lundbeck Foundation Initiative for Integrative Psychiatric Research, iPSYCH, Aarhus University, Aarhus, Denmark; Psychiatric Department P, Aarhus University Hospital, Aarhus, Denmark; BGI-Shenzhen, Shenzhen, China; Present address: Clinical Microbiological Section, Lillebælt Hospital, Vejle, Denmark; Present address: Epigenetic Regulation and Chromatin Architecture group, Berlin Institute for Medical Systems Biology, Max-Delbrück Centre for Molecular Medicine, Berlin, Germany

**Keywords:** Transcriptional-regulation, Transcription-factors, RNA-processing, ChIP-sequencing, RNA-binding, Gene-expression, Amyotrophic lateral sclerosis, Fronto-temporal lobar degeneration

## Abstract

**Background:**

FUS (TLS) and EWS (EWSR1) belong to the FET-protein family of RNA and DNA binding proteins. FUS and EWS are structurally and functionally related and participate in transcriptional regulation and RNA processing. FUS and EWS are identified in translocation generated cancer fusion proteins and involved in the human neurological diseases amyotrophic lateral sclerosis and fronto-temporal lobar degeneration.

**Results:**

To determine the gene regulatory functions of FUS and EWS at the level of chromatin, we have performed chromatin immunoprecipitation followed by next generation sequencing (ChIP-seq). Our results show that FUS and EWS bind to a subset of actively transcribed genes, that binding often is downstream the poly(A)-signal, and that binding overlaps with RNA polymerase II. Functional examinations of selected target genes identified that FUS and EWS can regulate gene expression at different levels. Gene Ontology analyses showed that FUS and EWS target genes preferentially encode proteins involved in regulatory processes at the RNA level.

**Conclusions:**

The presented results yield new insights into gene interactions of EWS and FUS and have identified a set of FUS and EWS target genes involved in pathways at the RNA regulatory level with potential to mediate normal and disease-associated functions of the FUS and EWS proteins.

**Electronic supplementary material:**

The online version of this article (doi:10.1186/s12864-015-2125-9) contains supplementary material, which is available to authorized users.

## Background

The FET-protein family comprises FUS (fused in sarcoma, and also abbreviated TLS (translocated in liposarcoma)), EWS (Ewing sarcoma breakpoint region 1, and also abbreviated EWSR1) and TAF15 (TATA box binding protein associated factor 68 kDa) [1]. The FET-proteins are implicated in cancer and originally identified as N-terminal partners of different fusion oncoproteins [2–4]. FET-proteins are also involved in neurological diseases with FUS and TAF15 mutations identified in familial amyotrophic lateral sclerosis (ALS) [5–8], frontotemporal lobar degeneration (FTLD) [9] and essential tremor disorders [10].

FET-proteins are expressed in most human tissues and cell types [11]. The FET-proteins are composed of several conserved domains: SYGQ-rich N-terminal domain, G-rich domain, RNA-binding domain (RRM), Zn-finger, and C-terminal RGG-rich domain [12]. The N-terminal region of FET-proteins has a transcriptional trans-activating function and is involved in homo and hetero dimerization [2, 13]. The C-terminal region is involved in subcellular localization and constitutes a hot spot for mutations associated with ALS and FTLD [11, 14–17]. FET-proteins shuttle between the cytoplasm and the nucleus and have a C-terminal nuclear localization signal [18, 19]. Cellular stress, as well as FUS and TAF15 mutations present in ALS and FTLD, results in cytoplasmic aggregation, appearing as immune reactive inclusion bodies in cultured cells and brain tissue. The inclusion body formation is concomitant with less nuclear FUS and TAF15 content [7, 8, 11, 13, 20]. The central and C-terminal protein regions including the RRM, Zn-finger and RGG-rich domains are implicated in RNA- and DNA-binding [18, 21–24]. FET-protein RNA target identification showed binding to thousands of RNA species and FET-proteins are functionally involved in transcriptional regulation, mRNA splicing and polyadenylation, RNA transport, RNA translation and microRNA (miRNA) processing [25–27]. FET-proteins associate with a number of factors involved in transcription and RNA processing, such as RNA Polymerase II (RNAPII), the basal transcriptional regulatory complex Transcription Factor IID (TFIID), and splice and polyadenylation factors [1, 3, 18, 28–32]. Moreover, FET-proteins are present in the Drosha miRNA processing complex [33, 34]. FUS and EWS have also been described playing a role in DNA repair. FUS and EWS deficient mice and zebra fish show defects in DNA pairing and DNA repair [35–38] and the FET-proteins are able to pair homologous DNA *in vitro* [39–41]. The pleiotropic functions of EWS and FUS are further illustrated by the role of FUS in DNA damage responses [42]. FUS is rapidly recruited to sites of double strand breaks in a poly(ADP-ribose) polymerase dependent manner and FUS depletion diminishes double strand break repair through both homologous recombination and non-homologous end-joining [42]. Furthermore, in response to DNA damage, FUS binds to a non-coding RNA transcribed upstream of the cyclin D1 (CCND1) gene, which leads to the inhibition of the histone acetyltransferase activities of CREB-binding and p300 proteins, thereby repressing CCND1 transcription [43]. RNA mediated recruitment of FUS to promoter regions goes beyond mechanisms directly related to DNA repair, and i.e. it was shown that in cortical neurons FUS binds the antisense RNA strand at the promoter region for a large set of genes and this results in transcriptional suppression of the coding strand [44]. Other studies have shown transcriptional regulation by FUS through promoter association such as involvement in the regulation of RNAPII C-terminal domain Ser2 phosphorylation and accordingly RNAPII accumulation at transcriptional start sites [24, 27]. This is functional linked with downstream poly(A)-signal selection in a process also dependent on FUS recruitment to the nascent RNA [27, 31]. FUS was also shown to activate transcription of genes related to oxidative stress defense through promoter binding [45].

Considering the fundamental roles the FET-proteins seem to play in normal cellular functions as well as in different types of human diseases, it will be important to elucidate the different mechanisms underlying the function of these proteins. In this study we have performed chromatin immunoprecipitation followed by next generation sequencing (ChIP-seq) to identify potential binding sites of FUS and EWS at the chromatin level. The results show that FUS and EWS bind downstream the poly(A)-signal in a subset of transcribed genes, that target genes are enriched for functions related to various aspects of RNA regulation, and that, for at least some of these genes, FUS and EWS have RNA processing functions.

## Results

### Identification of FUS and EWS genome-wide DNA binding sites

**Fig. 1:**
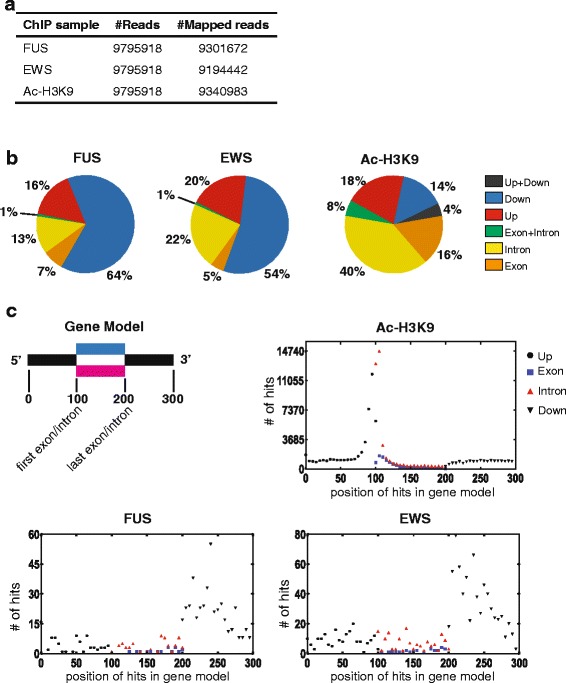
ChIP-seq profile of FUS and EWS binding sites in the human genome. **a** Total number of ChIP-seq reads and the ChIP-seq reads mapped to the human genome for NGS sequenced samples. **b** Distribution in a gene based model of FUS, EWS, and Ac-H3K9 peaks in or nearby (within distance of 10 kb) genes. Upstream region (Up, red) is defined as until 10 kb upstream of annotated gene transcriptional start sites, downstream region (Down, blue) is defined as until 10 kb downstream of annotated gene poly(A)-signals, upstream + downstream (Up + Down, black) specifies a chromosomal localization in where a peak is until 10 kb upstream for one gene but at the same time downstream of a neighboring gene, exon (orange), intron (yellow), and exon and intron (exon is intron of another gene, green). The location is defined accordingly to the summit of a given peak. **c** Position binding profile of FUS, EWS, and Ac-H3K9 based on a transcript based model in where each annotated transcript with a FUS, EWS, or Ac-H3K9 peak, the peak is localized accordingly to the gene model illustrated in the upper left corner including the features upstream (1–100), exon/intron (100–200), and downstream (200–300), and each feature further subdivided in smaller segments. The y-axis represents the number of times (hits) each ChIP-seq peaks map to annotated UCSC gene transcripts at the given segment position in the feature. Since multiple annotated transcripts often appear from one gene due to alternative splicing and promoter usage each ChIP-seq peak can generate several hits. The location is defined accordingly to the summit of the given ChIP-seq peaks

A hallmark of the FET-proteins is their ability to bind nucleic acids including RNAs as well as single and double stranded DNA [1, 12, 40, 41, 46, 47]. To identify target genes for FUS and EWS we conducted ChIP-seq analysis using human HEK-293 cells. We selected HEK-293 cells since genomics and RNomics studies at the time of our experimentation have used this genetic background to dissect regulatory functions of FUS and EWS, thereby allowing comparative analyses. The selected FUS and EWS monoclonal antibodies precipitated the expected proteins in cross-linked cell samples without any detectable cross-reactivity. Following ChIP, the eluted DNA fragments were subjected to Next Generation Sequencing (NGS) using the Illumina Hiseq 2000 platform. An equivalent amount of input DNA was used for NGS as a negative control and acetylated lysine 9 of histone H3 (Ac-H3K9) was included as a positive control for actively transcribed genes. 10^7^ sequence reads were extracted for each sample. The obtained raw sequence reads were aligned to the human reference genome (hg19) using Burrows-Wheeler Aligner [48]. For the three ChIP samples, over 94 % of the sequence reads were mapped to the reference genome (Fig. 1a). Aligned sequence reads were further processed with MACS 1.4.0rc2 for peaks calling [49]. A significant peak was defined using the criteria of a threshold ≥ 9 reads and p-value ≤ 10^−8^ [50]. A total of 52 and 133 enrichment peaks were significant for FUS and EWS, respectively, and of these 41 FUS peaks and 103 EWS peaks were positioned inside or proximate (within a distance of 10 kb) annotated genes (Additional files 1 and 2). We in the following focused on such peaks and characterized the distribution over target genes for the EWS and FUS peaks. We reasoned that if a peak is positioned within or in proximity of several genes, each of these genes should be encountered potential target genes (also abbreviated hits in the following). Using this approach, 134, 241, and 15115 genes were scored positive for FUS, EWS and Ac-H3K9, respectively (Table 1 and Additional file 3). It should be noted that since it is the distribution of ChIP peaks per annotated gene present in UCSC, each ChIP peak can results in multiple hits. Therefore, the numerical sum of the presented hits is accordingly not equal to the number of peaks. A more detailed analysis of the position of peaks revealed that a large proportion of FUS (64 % (32 peaks)) and EWS (54 % (62 peaks)) peaks are located downstream the poly(A)-signal in target genes (Figure 1b and Table 1). The remaining enrichment peaks for FUS and EWS were localized in the upstream region (16 % and 20 %, respectively), inside introns (13 % and 22 %, respectively) and within exons (7 % and 5 %, respectively). We note that Ac-H3K9 peaks more often localized upstream of genes and in introns and exons, whereas only a smaller number (14 %) were assigned to localize downstream the poly(A)-signal (Fig. 1b and Table 1).

**Table 1:** Gene distribution of FUS, EWS and Ac-H3K9 ChIP-seq peaks in a gene based model

Protein	All	Exon	Intron	Exon + Intron	Upstream	Downstream	Upstream + Downstream
FUS	134	9	17	1	21	86	0
EWS	241	11	52	2	47	129	0
Ac-H3K9	15115	2510	5911	804	3073	2164	653

Gene distributions were subdivided into six groups: exon, intron, exon + intron (exon is intron sequence in another gene), upstream (10 kb distance), downstream (10 kb distance), and upstream + downstream. The latter specifies a chromosomal localization in where a peak is until 10 kb upstream for one gene but at the same time downstream of a neighboring gene

To identify the pathways to which FUS and EWS target genes belongs we performed a gene ontology (GO) and Kyoto encyclopedia of genes and genomes (KEGG) pathways analysis using the software ChIP-Enrich specifically developed for gene set enrichment testing of ChIP-seq results [51]. For these analyses we used a cut-off FDR < 0.01. The most significant results are presented in Table 2 and the complete output in Additional file 4. ChIP-Enrich analysis revealed that for both FUS and EWS the most enriched KEGG pathway is Ribosome (p-values 5.79E-06 and 2.32E-07 for FUS and EWS, respectively) (Table 2 and Additional file 4). The most enriched GO Biological Process for both FUS and EWS is Translational Elongation (p-values 1.27E-08 and 7.86E-13 for FUS and EWS, respectively). Additionally, a prominent enrichment of regulatory processes at the RNA level was observed within the GO Cellular Component for FUS. The 5 most significant enriched gene sets were heterogeneous nuclear ribonucleoprotein complex, cytosolic ribosome, ribonucleoprotein complex, ribosomal subunit, and cytosolic large ribosomal subunit (Table 2 and Additional file 4). Four of these five gene sets were also among the five most significant EWS GO Cellular Component gene sets (Table 2 and Additional file 4). Inspection of the entire FUS and EWS ChIP-Enrich analysis revealed existence of many additional GO enriched gene sets involved in regulatory processes at the RNA level (Additional file 4).

**Table 2:** ChIP-Enrich analysis of FUS and EWS ChIP-seq peaks

FUS ChIP-seq gene set enrichment analysis (FDR value < 0.01)				
GO/KEGG ID	GO/KEGG category	Gene Set Description	P value	FDR
GO:0006414	Gene Ontology Biological Process	translational elongation	1.27E-08	5.13E-05
GO:2000602	Gene Ontology Biological Process	regulation of interphase of mitotic cell cycle	2.64E-07	5.34E-04
GO:0006415	Gene Ontology Biological Process	translational termination	2.55E-06	0.0027
GO:0007346	Gene Ontology Biological Process	regulation of mitotic cell cycle	2.67E-06	0.0027
GO:0006614	Gene Ontology Biological Process	SRP-dependent cotranslational protein targeting to membrane	6.51E-06	0.00404
GO:0030530	Gene Ontology Cellular Component	heterogeneous nuclear ribonucleoprotein complex	7.58E-08	3.74E-05
GO:0022626	Gene Ontology Cellular Component	cytosolic ribosome	1.56E-06	3.85E-04
GO:0030529	Gene Ontology Cellular Component	ribonucleoprotein complex	2.20E-05	0.00347
GO:0044391	Gene Ontology Cellular Component	ribosomal subunit	2.81E-05	0.00347
GO:0022625	Gene Ontology Cellular Component	cytosolic large ribosomal subunit	3.54E-05	0.00349
GO:0043566	Gene Ontology Molecular Function	structure-specific DNA binding	8.88E-06	0.0077
path:hsa03010	KEGG Pathway	Ribosome	5.79E-06	0.00108
EWS ChIP-seq gene set enrichment analysis (FDR value < 0.01)				
GO/ KEGG ID	GO/KEGG category	Gene Set Description	P value	FDR
GO:0006414	Gene Ontology Biological Process	translational elongation	7.86E-13	3.18E-09
GO:0006613	Gene Ontology Biological Process	cotranslational protein targeting to membrane	1.58E-12	3.19E-09
GO:0006415	Gene Ontology Biological Process	translational termination	4.60E-11	6.20E-08
GO:0006614	Gene Ontology Biological Process	SRP-dependent cotranslational protein targeting to membrane	4.17E-10	3.91E-07
GO:0045047	Gene Ontology Biological Process	protein targeting to ER	4.83E-10	3.91E-07
GO:0022626	Gene Ontology Cellular Component	cytosolic ribosome	1.92E-11	9.49E-09
GO:0044391	Gene Ontology Cellular Component	ribosomal subunit	4.70E-09	8.13E-07
GO:0030530	Gene Ontology Cellular Component	heterogeneous nuclear ribonucleoprotein complex	4.95E-09	8.13E-07
GO:0030529	Gene Ontology Cellular Component	ribonucleoprotein complex	1.67E-07	2.06E-05
GO:0005730	Gene Ontology Cellular Component	nucleolus	2.95E-07	2.69E-05
GO:0003735	Gene Ontology Molecular Function	structural constituent of ribosome	5.34E-08	2.70E-05
path:hsa03010	KEGG Pathway	Ribosome	2.32E-07	4.33E-05

The presented ChIP-Enrich results represents for FUS the 5 most significant GO signatures for Biological Process and for Cellular Component, as well as the single present GO signature for Molecular Function and KEGG pathway. For comparison the same set-up for ChIP-Enrich results were presented for EWS. The entire ChIP-Enrich analyze list is included in Additional file 4

**Fig. 2:**
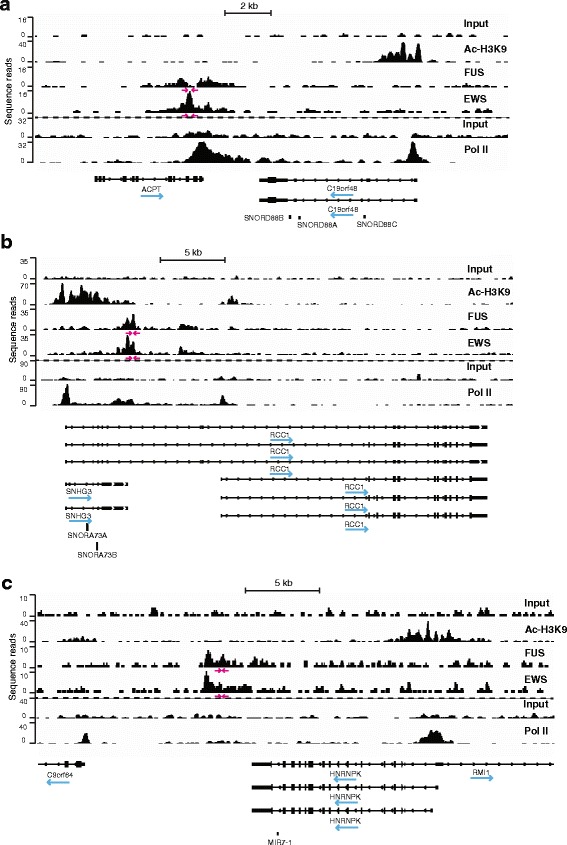
Graphical illustrations of representative ChIP-seq results. ChIP-seq sequence reads for input, FUS, EWS, Ac-H3K9 and RNAPII were aligned to representative genes by Integrative Genomics Viewer 2.0. The number of sequence reads is presented on the y-axis within each figure. Transcripts from the UCSC hg19 genomic database are shown in the bottom section of each figure. **a** ChIP-seq reads aligned to the neighboring *ACPT* and *C19orf48* genes. **b** ChIP-seq reads aligned to the overlapping *RCC1* and *SNHG3* genes with additional inclusion of *SNORA73A* and *SNORA73B* genes. We note the presence of major FUS and EWS peaks corresponding to the position of the annotated poly(A)-signal of *SNHG3* as well as minor FUS and EWS peaks 4 kb further 3’ end positioned. **c** ChIP-seq reads aligned to the *HNRNPK* gene with additional inclusion of *miR-7-1*. Dashed lines separate the paired input, ac-H3K9, FUS and EWS ChIP seq dataset from the paired input and RNAPII ChIP-seq dataset. Red arrows show positions of primers used for qPCR based verification of ChIP results. Blue arrows under gene names show direction of transcription

To illustrate by an alternative approach the position of FUS and EWS ChIP-seq peaks we next used a transcript based model in which each annotated transcript was analyzed for the presence of FUS, EWS, and Ac-H3K9 peaks within or in close distance to the transcript (up to 10 kb). The location of such peaks was mapped relative to a standard gene model consisting of four genetic features: upstream (before first exon), exon and intron, as well as downstream representing a location after the poly(A)-signal (Fig. 1c). Each of these features was sub-divided into 100 segments for more precise mapping. As shown in Figure 1c, FUS and EWS preferentially bind the feature downstream of the poly(A)-signal. It should again be noted that, since it is the distribution of ChIP peaks per annotated gene transcript present in UCSC, each ChIP peak can result in multiple hits. Fig 1c also illustrates that Ac-H3K9 ChIP peaks, if designated to the upstream feature mostly correspond to the 3’ end of this feature, and if ChIP-peaks are designated to exons and introns feature, the peaks mostly correspond to the 5’ end of this feature. This is in accordance with Ac-H3K9 being a mark for transcriptional start sites. Examples of FUS, EWS, and Ac-H3K9 enrichment peaks are shown for the *ACPT* and *C19orf48* gene complex (with p values scored number 7 of the 103 peaks and 16 of the 41 peaks for EWS and FUS, respectively), *RCC1* and *SNHG3* gene complex (with p values scored number 5 of the 103 peaks and 3 of the 41 peaks for EWS and FUS, respectively), and *HNRNPK* gene (with p values scored number 34 of the 103 peaks and 26 of the 41 peaks for EWS and FUS, respectively) (Fig. 2a-c). The read number is shown at the left axis of each figure. The scale difference for the Ac-H3K9 ChIP-seq enrichment (upper left) should be noted. The transcripts from UCSC hg19 genomic database are shown at the bottom. ChIP-seq results were validated by repeated ChIP experiments and analysis of representative genomic regions by quantitative PCR (qPCR) (Additional file 5).

### FUS and EWS bind actively transcribed genes

**Fig. 3:**
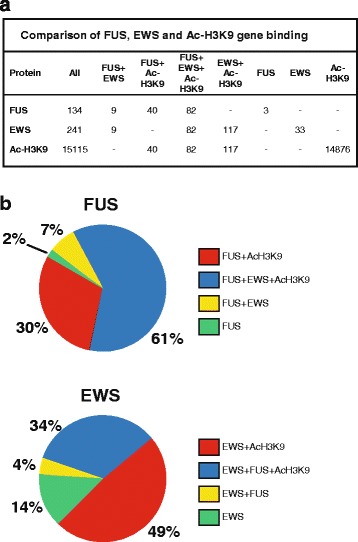
Characterization of FUS and EWS target genes. **a** The 134, 241, and 15115 genes identified as potential targets for FUS, EWS or Ac-H3K9, respectively, were in addition examined for also the presence of peaks representing the two other ChIP-seq experiments. **b** The percentage distribution of genes with either individual or combined FUS, EWS, and Ac-H3K9 peaks. Percentages were calculated from the numbers in (**a**)

**Fig. 4:**
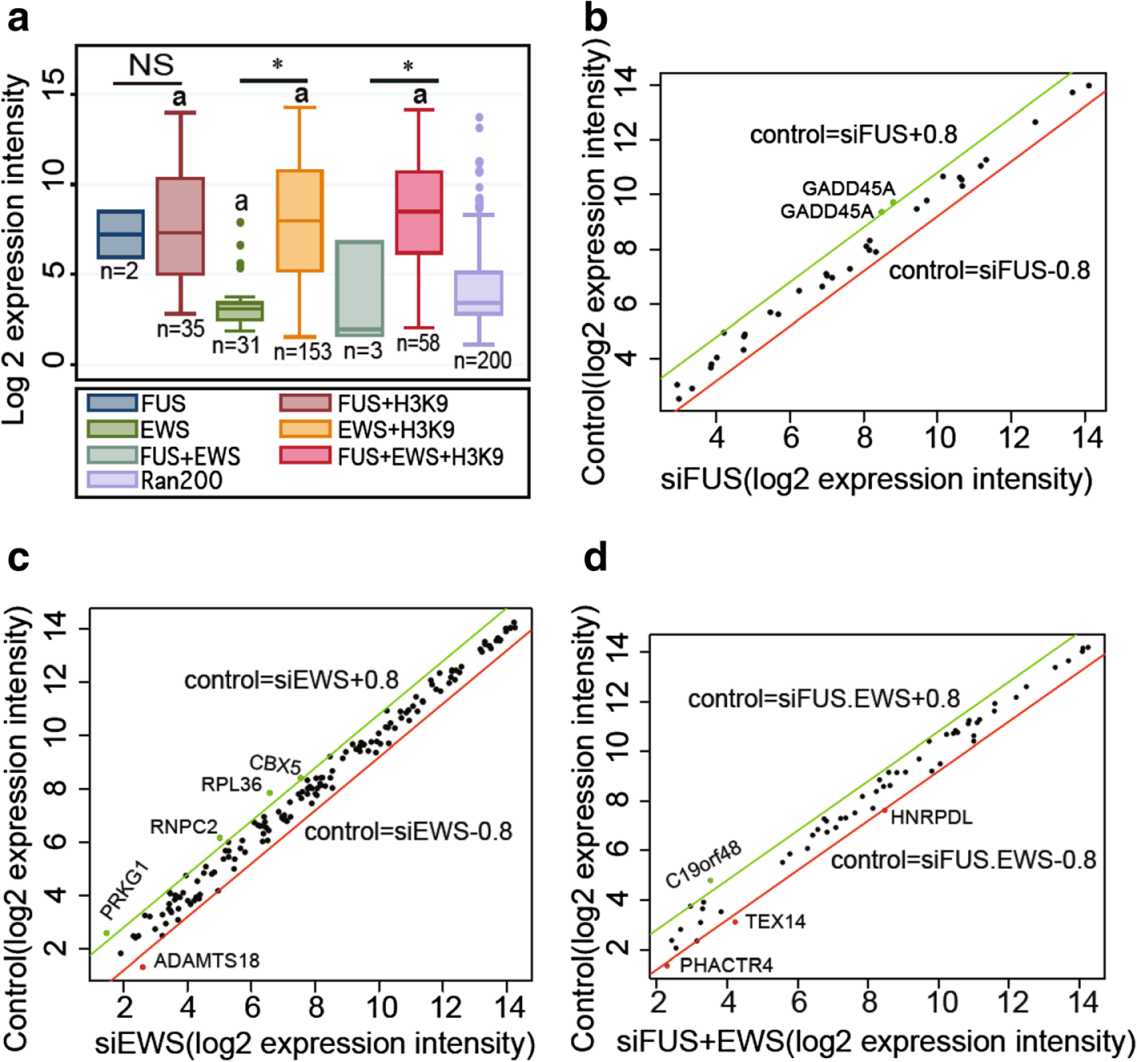
FUS and EWS bind with preference actively transcribed genes. **a** Box plot of log 2 expression intensity of FUS, EWS, and combined FUS and EWS target genes either without or with additional Ac-H3K9 enrichment. A random selection of 200 probe sets was used as microarray control probe set. “a”, p-value < 0.05, for data sets compared against the randomly selected 200 probe sets; “*”, p–value < 0.05, when comparing ChIP-seq signatures with and without H3K9; NS, non-significance. **b-d** Dot plots comparing microarray data for control and individual or coupled FUS and EWS siRNA transfected cells with ChIP-seq signatures for FUS and Ac-H3K9 (n = 35) (**b**), EWS and Ac-H3K9 (n = 153) (**c**) or FUS and EWS and Ac-H3K9 (n = 58) (**d**). The red line indicates the significant up-regulation boundary of log (siRNA-control) fold change = 0.8, and the green line indicates the significant down-regulation boundary of log (siRNA-control) fold change = −0.8

Cross comparison of the identified target genes for FUS and Ac-H3K9 showed that out of 134 genes with FUS binding assigned, 122 were also enriched for Ac-H3K9, indicating that most FUS target genes are actively transcribed (Fig. 3a and b and Additional file 6). The same was observed for EWS target genes, since out of 241 genes with EWS binding assigned, 199 were also enriched for Ac-H3K9 (Fig. 3 and Additional file 6). Further analysis of FUS and EWS ChIP-seq peaks revealed that a large proportion of target genes were bound by both FUS and EWS (Fig. 3 and Additional file 6). To validate if FUS and EWS target genes represented actively transcribed genes, we compared our ChIP-seq datasets with mRNA expression analyses previously conducted in HEK-293 cells [17]. We analyzed the expression profile of targeted genes in two DNA microarray datasets (expression arrays A664_04 and A664_06 [17]). Among the 48,803 probe sets representing 17,202 unique genes in the Illumina GPL6884 microarray, we identified 35 probe sets representing ChIP-seq target genes for FUS and Ac-H3K9, 135 probe sets for EWS and Ac-H3K9, and 58 probe sets for FUS, EWS, and Ac-H3K9 (Additional file 7). In addition, we identified 2, 31 or 3 probe sets representing the ChIP-seq targeted genes with binding of FUS, EWS, or both, respectively, in the absence of Ac-H3K9 (Additional file 7). We note that the transcripts presented on the microarray are enriched for mRNA with protein coding potential and thereby most ncRNAs are excluded from our analyses. By comparing the relative expression level of FUS, EWS, or FUS and EWS target genes with or without Ac-H3K9, an active transcriptional status could be attributed to target genes (Fig. 4a). The number of FUS target genes without Ac-H3K9 was very low (2 genes) which most likely explains the lack of significant change in expression levels between FUS target genes without Ac-H3K9 relative to the FUS and Ac-H3K9 target genes (Fig. 4a). A control set of 200 randomized selected probes representing the microarray was less expressed than probe sets with FUS and Ac-H3K9, as well as EWS and Ac-H3K9, further supporting that FUS and EWS target genes are actively transcribed (Fig. 4a). We performed GO analysis of the above described probe set groups representing actively transcribed genes with ChIP-seq peaks of FUS, EWS and Ac-H3K9 (Additional file 8). We note that this GO analysis is not directly comparable with the ChIP-Enrich analysis presented in Table 2 due to different grouping and definition of input genes. Nevertheless, consistence with the ChIP-Enrich analysis was evident (Additional file 8). For EWS and Ac-H3K9 probe set group the most significant GO Biological Process was translational elongation and GO Molecular Function was RNA binding (Additional file 8). For the FUS and Ac-H3K9 probe set we note that there was significant enrichment for GO Cellular Component gene sets representing cytosolic ribosome, ribosomal subunit, and cytosolic large ribosomal subunit, which were also detected by ChIP-Enrich. For the probe set representing FUS, EWS, and Ac-H3K9 target genes we observed enrichment of GO gene sets including GO Molecular Function RNA binding and several enriched gene sets related to ribonucleotide binding, as well as the GO Biological Processes gene-expression and translation (Additional file 8).

We further compared the actively transcribed gene groups FUS and Ac-H3K9, EWS and Ac-H3K9, and FUS, EWS, and Ac-H3K9, with microarray datasets representing HEK-293 cells with siRNA mediated depletion of FUS, EWS, or a combined FUS and EWS depletion (Fig. 4b-d) [47]. Few of the transcribed EWS and FUS target genes had altered transcription as a consequence of EWS and FUS depletion, suggesting that the binding of EWS and FUS to target genes is not essential for the given transcriptional level (Fig. 4b-d).

**Fig. 5:**
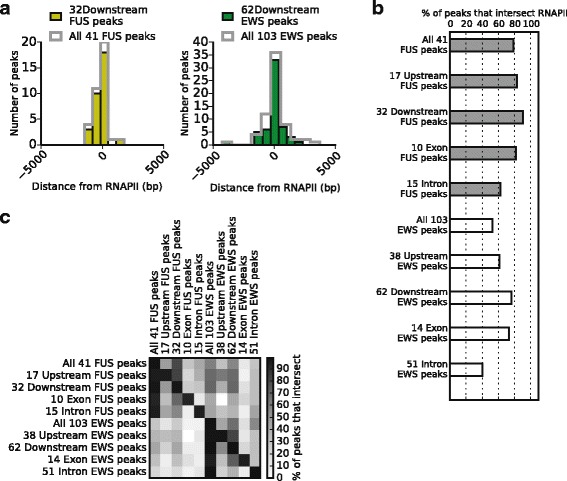
EWS and FUS binding downstream the poly(A)-signal overlap with RNAPII binding. **a** The distance in base pairs (bp) for the 41 FUS gene localized and the 32 FUS downstream localized ChIP-seq peaks (yellow), as well as for the 103 EWS gene localized and the 62 EWS downstream localized ChIP-seq peaks (green), to RNAPII binding (26,323 ChIP-seq peaks from ENCODE Project Consortium). Distances were plotted as histograms, where x = 0 indicate the center of RNAPII binding. The y-axis show the number of FUS and EWS ChIP-seq peaks in each bar. **b** Presentation of the percentage of FUS (grey) and EWS (white) ChIP-seq peaks, both for the total number of gene localized peaks, as well as peaks belonging to different categories for gene localization, intersecting at least one base with RNAPII peaks. **c** Presentation of the percentage of FUS and EWS ChIP-seq peaks, both for the total number of gene localized peaks, as well as peaks belonging to different categories for gene localization, that intersect (at least one base). At the right side is shown the coloring code for the intersection percentages. The percentage of FUS, EWS, and RNAPII intersection with a randomized generated peak set was < 1.8 % based on 1 x 10^5^ simulations of the randomized peaks having the same length and chromosome distribution as the RNAPII ChIP-seq peaks yielding a significance level of p < 1 x 10^−5^

Accumulation of RNAPII in the downstream region of genes has been described to be relatively common [52]. Considering that FUS and EWS largely target genes that are actively transcribed, we hypothesized that FUS and EWS binding downstream the poly(A)-signal could be associated with RNAPII accumulation. To investigate this, we calculated the distance between RNAPII and FUS or EWS peaks downstream the poly(A)-signal. RNAPII genomic occupancy profile in HEK-293 cells was obtained from ENCODE ChIP-seq data and FUS and EWS ChIP-seq peaks located downstream the poly(A)-signal is presented in Additional file 9. The analysis showed that FUS and EWS binding overlaps to RNAPII binding, supporting that binding of FUS and EWS downstream from the last exon is associated with RNAPII accumulation (Fig. 5a). We next examined whether FUS and EWS ChIP-seq peaks were intersecting (defined as an overlap of at least one base pair) with RNAPII ChIP-seq peaks. FUS and EWS ChIP-seq peaks were categorized accordingly to their gene localization as upstream, exon, intron, or downstream (see also Table 1 and Fig. 1c). Note that one ChIP-seq peak can belong to several categories. The intersection analyses showed that in all FUS peak categories, most peaks intersected with RNAPII binding (Fig. 5b). For FUS peaks, the most prominent intersection with RNAPII peaks was for peaks localized downstream (91 %) (Fig. 5b). For EWS peaks, we observed a general lower intersection with RNAPII peaks (Fig. 5b). Nevertheless, we also observed for EWS peaks a prominent intersection between peaks localizing downstream and RNAPII peaks (76 %) (Fig. 5b). Thus, we conclude that FUS and EWS binding downstream the poly(A)-signal often associates with RNAPII accumulation at the same position. Extending the analyses to also examine for intersection of FUS and EWS ChIP-seq peaks, we observed peak intersections within all localization categories (Fig. 5c). This is in alignment with the previous presented results showing that FUS and EWS have a common group of target genes (Fig. 3).

### Functional analysis of exemplified FUS and EWS ChIP-seq peaks

**Fig. 6:**
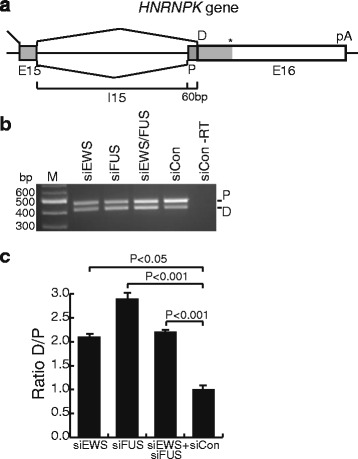
FUS and EWS influence *HNRNPK* splice site selection. **a** Graphical illustration of *HNRNPK* alternative splice acceptor site selection in exon 16. P, proximal splice site; D, distal splice site. On top are indicated the coding region in gray and positions of translational stop (*) and Poly(A)-signal (pA). **b** RT-PCR and gel electrophoresis of siEWS, siFUS, siEWS and siFUS, and siControl depleted HEK-293 cells. This image is representative of 3 independent experiments. M, 100 bp DNA ladder. –RT, negative control of PCR mix without template cDNA. **c** Ratio of D (distal) to P (proximal) splice acceptor site usage in siRNA transfected cells. The ratio was calculated from relative band intensity values by GelQuant software. The ratio for siCon was given the value 1 and the ratios from siRNA-depleted samples were calculated accordingly. Experiments were performed in biological triplicates. Data presented as mean + SEM

To investigate the functional implications of EWS and FUS binding in representative genes, we performed depletion of FUS, EWS or both, by transfecting HEK-293 cells with siRNA. Depletion efficiency was determined at both mRNA and protein levels by qPCR and western blot (Additional file 10). Firstly, we analyzed the genomic region annotated with *C19orf48* and *ACPT* genes. For *C19orf48,* EWS, FUS, and RNAPII ChIP-seq peaks were detected downstream the poly(A)-signal, as well as having Ac-H3K9 and RNAPII ChIP-seq peaks at the promoter region (Fig. 2a). We note that the *ACPT* gene, which directly overlaps with the FUS and EWS ChIP-seq peaks, but not Ac-H3K9, was not expressed to a detectable level in HEK-293 cells, either with or without FUS and EWS depletion, decreasing the likelihood that *ACPT* represents a functional EWS and FUS target gene (data not shown). To determine the eventual functional effects of FUS and EWS, we next examined by RT-qPCR the expression levels of *C19orf48* isoforms, *C19orf48* intron retention, and amounts of the *SNORD88* precursors generated from *C19orf48* intron sequences. The results showed that FUS and EWS depletion had no direct effect on the expression of the examined RNA species using a significance threshold of 1.5 fold changes (Additional file 11). We next analyzed the overlapping *RCC1* and *SNHG3* gene complex (Fig. 2b). We note the presence of major FUS and EWS enrichment corresponding to the position of the annotated poly(A)-signal of *SNHG3,* as well as minor FUS and EWS peaks approximately 4 kb further downstream (Fig. 2b). The *RCC1-*gene has different mRNA isoforms due to alternative transcription start sites and/or alternative splicing (Fig. 2b). The *RCC1* downstream transcription start site is located after *RCC1* exon 4 and displays overlapping Ac-H3K9 and RNAPII enrichment peaks indicating active transcription (Fig. 2b). The *RCC1* upstream transcription start site overlaps with the transcription start site of *SNHG3* gene, which is a non-coding gene. The first two exons of the large forms of *RCC1* and *SNHG3* mRNAs are identical and two *SNHG3* mRNA isoforms can be produced from *RCC1*. This transcriptional start site also contains overlapping enrichment peaks for Ac-H3K9 and RNAPII (Fig. 2b). Additionally, two small nucleolar RNA precursors, *SNORA73A* and *SNORA73B*, are produced from the introns of *RCC1* and *SNHG3* genes (Fig. 2b). RT-qPCR was used to investigate if the various *RCC1*, *SNHG3* and *SNORA73* RNA isoforms generated from this genomic region were affected by depletion of FUS and EWS. The only significant change observed was reduction in *RCC1* transcripts initiated from the upstream transcription start site in FUS depleted cells (Additional file 12). Finally, we analyzed the *HNRNPK* gene, with FUS and EWS binding downstream of the poly(A)-signal (Fig. 2c). Distinct *HNRNPK* mRNA isoforms are produced by usage of two alternative first exons, as well as alternative splice acceptor sites at the last exon (Exon 16). Moreover, one of the microRNA precursors of *miR7-1* is generated from *HNRNPK* intron 15. The expression levels of *HNRNPK* transcripts were not significantly changed by EWS and FUS depletion (Additional file 13). To evaluate FUS and EWS effect on alternative splicing involving exon 16, RT-PCR followed by gel electrophoresis was performed. Two bands with distinct sizes were observed for both coupled and individual FUS and EWS depletions as well as for control, reflecting the usage of proximal (P) and distal (D) acceptor sites (Fig 6a-c). FUS and EWS depletion caused a significant increase of *HNRNPK* transcripts, which used the distal exon 16 splice acceptor site (D) (Fig 6a-c and Additional file 13).

## Discussion

The coupling of transcription with RNA processing is widely accepted and, in recent years, it has become more evident that the functional importance of FUS and EWS may reside in this interface. Several studies have addressed the association of FUS and EWS with RNA. However, despite early *in vitro* evidence for the DNA-binding properties of the FET-proteins [1, 12, 40, 41, 46, 47], few studies identifying *in situ* target genes for FUS and EWS have been conducted [24, 30, 53]. We here present a profile for genomic binding of FUS and EWS by ChIP-seq in HEK-293 cells. To our knowledge this is the first presentation of EWS ChIP-seq data whereas one FUS ChIP-seq and one FUS ChIP-chip dataset were previously published [24, 30]. Our studies were conducted in HEK-293 cells since other FUS and EWS RNomics and genomics studies, including genome-wide RNA binding analysis and FUS ChIP-seq analysis, have used the HEK-293 genetic background to dissect the regulatory functions. Thereby, cross-experimental comparative analyses are possible by minimizing potential interference from cell line genetic differences and cell type specific gene expression profiles. Nevertheless, we acknowledge that use of the single HEK-293 cell line model has limitations in terms of delineating the general biological implications of the identified FUS and EWS gene binding profiles. It will be informative to extend FUS and EWS ChIP-seq profiling to other cell models to study the protein functions in more details and in relation to specific diseases involving FUS and EWS deregulation, such as cancer and neurodegenerative disorders.

We identified a small number of significant peaks for FUS (52 peaks) and EWS (133 peaks). Of these peaks, 41 for FUS and 103 for EWS were located within or in close proximity (distance of 10 kb) of annotated genes. Given that one peak at a given genomic position often can be assigned to more than one annotated gene, 134 and 241 genes were identified as potential targets for FUS and EWS, respectively. Interestingly, 91 genes were common *in situ* targets for FUS and EWS. Moreover, our peak intersection analysis showed that FUS and EWS ChIP-seq peaks often overlap, which altogether supports a functional cooperation or redundancy of these proteins. This observation correlates with previous descriptions of FUS and EWS being together in protein complexes [13, 54]. The hereby identified binding of FUS and EWS to genes in HEK-293 cells could be directly mediated through multiple nucleic acid interaction domains in EWS and FUS, or indirect as cross linking in the ChIP protocol strengthens protein-protein and tripartite protein-RNA-DNA interactions. In our study 16 % of FUS and 20 % of EWS enrichment peaks were in the upstream gene region including the promoter. FUS and EWS were reported to both enhance and inhibit transcription, being a common characteristic of these studies the binding of FUS and EWS mainly at the promoter region [30, 43–45, 55, 56]. Additionally, genome-wide RNA cross-linking and IP (RNA-CLIP) analysis has shown that FUS interacts with antisense RNA originating from promoter regions [44]. This FUS-RNA interaction mediates transcriptional down regulation from the coding strand, indicating a function of FUS in transcription in a position-dependent manner [44]. Schwarts et al. showed that FUS binds the C-terminal domain (CTD) of RNAPII, preventing inappropriate CTD Ser-2 hyperphosphorylation at thousands of human genes [30]. FUS depletion caused RNAPII accumulation at the transcription start site and a shift in the corresponding mRNA expression profile towards usage of early poly(A)-signals [30]. FUS ChIP-seq analyses in HEK-293 cells by Schwarts et al. showed binding of FUS to the transcriptional start site (TSS) in 68 % of transcribed genes, concomitant with RNAPII presence [30]. This contrasts with our ChIP-seq data, where promoter and 5’ gene region binding of FUS only constitutes a minor proportion of the FUS *in situ* targets. We here also note that a FUS ChIP-chip experiment by Tan et al. identified direct FUS interaction to promoter sequences in HeLa cells [24]. Whereas Tan et al. data were not straightforward comparable with our results due to different sample genetics and ChIP methodology, a dataset comparison with FUS ChIP-seq data from Schwarts et al. was more straightforward [24, 30]. The ChIP-seq experiment by Schwarts et al. was performed in HEK-293 T/17 cells and our study in the parental HEK-293 cell line. In ChIP-seq data analysis we have compared input DNA sequences versus FUS ChIP DNA sequences whereas Schwarts et al. compared FUS ChIP DNA sequences generated from cells either pre-transfected with a scrambled siRNA control or siRNA against FUS with the latter ChIP-seq only resulted in relative few sequence reads which could be mapped to hg19 (Additional file 14 and [30]). Secondly, Schwarts et al. used the peak-calling software FindPeaks, whereas we used MACS, with only MACS also using input DNA sequence data for peak estimation [57–59]. We reanalyzed our and Schwarts et al. FUS ChIP-seq data with the FindPeaks and MACS peak calling programs. The p-value distribution for called peaks was similar between the two datasets using MACS, whereas when using FindPeaks a high proportion of the peaks called from Schwarts et al. data clustered with high p-value (Additional file 15). The distribution of MACS and FindPeaks called peaks over a model gene was next analyzed using a serial decrease in p-value cut-off levels. Using MACS peak calling, FUS ChIP-seq peaks corresponding to promoter regions were identified in our data, but not in numerical alignment with Schwarts et al., which described preferential promoter association to thousands of genes (Additional file 16). Moreover, MACS identified in both datasets a small number of FUS ChIP-seq peaks downstream the poly(A)-signal (Additional file 16). Using FindPeaks peak calling, FUS ChIP-seq peaks corresponding to promoter association to thousands of genes were identified only in Schwarts et al. data, and preferentially with high p-values (Additional file 17). Moreover, using FindPeaks identified in our and Schwarts et al. datasets a small number of FUS ChIP-seq peaks downstream the poly(A)-signal (Additional file 17). We conclude that overall discrepancies in obtained results seem to be a combination of the DNA co-immunoprecipitated under the given ChIP experimental conditions, criteria used for peak calling, and that the small number of downstream the poly(A)-signal peaks do not appear evident in the Schwarts et al. study given the much larger FUS enrichment at promoter regions. However, we find it important that the identification of FUS and EWS binding downstream the poly(A)-signal of a small subset of transcribed target genes irrespectively of peak calling methods and sequence datasets, indicates an overseen putative regulatory mechanism for gene targeting of FUS and EWS with potential implications for regulation at the RNA level.

RNA processing factors can be recruited to transcribed genes in multiple, not mutually exclusive, ways: i) by binding to the DNA template either directly or through recruiting factors, ii) by binding to processing signals present in the nascent RNA and iii) by binding to defined regions of the RNAPII elongating complex i.e. CTD [60]. In mammals, RNAPII transcriptional termination can occur anywhere from a few bases to several kb downstream from the poly(A)-signal [61]. Previous studies have revealed a general higher average RNAPII density downstream from the poly(A)-signal compared to the transcribed region [62, 63]. However, a subsequent study proposed that these observations were biased, considering that most active genes have RNAPII evenly distributed before and after the poly(A)-signal, but that a subset of actively transcribed genes (7 % to 14 %) contained RNAPII enrichment downstream the poly(A)-signal [52]. Association of 3’-end processing factors, capping factors, Spt5, and Ser-2 hyper-phosphorylated paused RNAPII was identified approximately 0.5-1.5 kb downstream of the poly(A)-signal [60]. The CDK9 kinase component of P-TEFb, which mediates RNAPII CTD Ser-2 phosphorylation, is also present downstream of the poly(A)-signal and this is being coupled with correct assembly of the spliceosome [64]. Thus, RNAPII pausing downstream of the poly(A)-signal, transcription termination and pre-mRNA processing seems to be highly interconnected. A link to transcriptional re-initiation is also opened [65]. GO analysis showed that genes with and without downstream RNAPII enrichment are implicated in different cellular functions [52]. It has been shown that FUS and EWS interact with different spliceosome components but also with RNAPII and TFIID [1, 28, 66, 67]. In this line, EWS is shown to function as a co-transcriptional regulator of alternative splicing which can bind alternatively spliced exons at both the chromatin template level and in the nucleoplasm [68]. FUS RNA-CLIP analyses showed widespread cross-linking along the whole length of associated pre-RNAs, suggesting that FUS associates with target RNAs until splicing is completed [44, 69–71]. So far, simple RNA binding motifs which could explain RNA-binding patterns of EWS and FUS have not been identified, but a preference for GU-rich motifs and short-stem loops was proposed to facilitate binding [25]. The hereby described binding of FUS and EWS downstream the poly(A)-signal of actively transcribed genes often overlaps RNAPII binding, and this could be mechanistically linked with the gene recruitment process of FUS and EWS. Comparative analysis of the identified FUS and EWS target genes with expression data showed that target genes are mostly transcribed, but that neither FUS nor EWS have in general major impact on basal transcriptional activity. This is in alignment with other studies pointing that, due to the discrepancy in the number of identified FUS RNA targets and the number of genes differentially expressed after alterations in the FUS expression level, basal transcriptional regulation seems to not be the main function of FUS [30, 70, 71]. Numerous studies have identified RNA targets for FUS and EWS and characterized their regulatory impact. RNA interactions have been extensively analyzed, particular after the identification of FUS mutations associated with ALS [26, 30, 44, 69–71]. A comparison of RNA-CLIP identified RNA targets for FUS and EWS [26] and the genes hereby identified by ChIP-seq, showed that 37 out of 134, and 68 out of 241 genes for FUS and EWS, respectively, were overlapping (Additional file 18). Note that FUS and EWS target genes characterized in terms of expression in this report were also identified by RNA-CLIP in Hoell et al. [26]*.* While addressing the regulatory function of FUS and EWS in this study, we were unable to identify significant effects for *C19orf48*. For the *RCC1* gene, FUS and EWS seem to regulate the relative levels of *RCC1* transcript isoforms. One possible mechanism could be that FUS and EWS binding in the *SNHG3* gene, which is intragenic to *RCC1*, enhance the transcription initiated from the upstream *RCC1* promoter. Another possibility could be that binding of FUS and EWS is associated with RNAPII pausing, which in turn could modulate the subsequent splicing pattern of *RCC1* mRNA. A similar mechanism is found regulating the alternative splicing of *CD45* mRNA mediated by the DNA-binding protein CCCTC-binding factor [72]. In addition, transcriptional pausing at terminal exons was described as a general phenomenon linking chromatin structure to RNA metabolism [73]. For the *HNRNPK* gene, we observed a FUS and EWS function on alternative splice site selection at the last exon. In accordance, FUS was also previously identified as a splicing regulator of *HNRNPK* [25, 69, 71]. We note that depletion of FUS and EWS results in up-regulation of the third FET-protein, TAF15, and this indirectly could also participate in the regulation (data not shown). During the finalization of this manuscript it was described that position-specific binding of FUS to nascent RNA regulates mRNA length through alternative poly(A)-signal usage [31]. FUS was shown to stall RNAPII and prematurely terminate transcription when FUS RNA binding was downstream the poly(A)-signal [31]. We note that this scenario could be in alignment with our observations of FUS and EWS binding downstream the poly(A)-signal in conjugation with RNAPII accumulation, and that FUS and EWS could be involved in the functional regulation of 3’ end processing related events of such target genes. It is clear that EWS and FUS regulate alternative RNA processing of genes involved in neurodevelopmental and neurodegenerative processes [25, 31, 44, 70]. FUS also contributes to the biogenesis of a specific subset of miRNAs, including species relevant for neuronal function, differentiation and synaptogenesis [53]. FUS RNA CLIP-tag distributions are similar in mouse and human neurons, indicating conserved functions for these genes, and target mRNA GO terms implicated FUS in the regulation of vital genes for neuronal maintenance, development and function [31, 71]. GO analysis of FUS and EWS mRNA targets revealed enrichment in biological processes of DNA repair and spliceosome assembly [26, 74, 75], and preferential binding of FUS with pre-mRNAs encoding RBPs was associated with a regulatory function of FUS in alternative splicing of RBP encoding genes in neurons [71]. Thus, FUS seems to participate in a cross-regulatory network with other RBPs, further suggesting that perturbations of FUS in ALS and FTLD may result in both direct and indirect transcriptome changes through the effect of FUS on other RBPs [71]. Our results are in line with the above mentioned studies, as GO profiles of EWS and FUS target genes showed enrichment in signaling networks related to ribosomal functions, RNA processing, and translation. This further highlight the putative functions of FUS and EWS in regulating genes encoding proteins associated with the RNA regulome and that deregulation of particular components of this program may be an important factor in cancer and neurological diseases.

## Conclusions

The presented results show that FUS and EWS associate with a subset of transcribed genes, often downstream the poly(A)-signal, and that the identified target genes are functionally enriched for encoding proteins with role in RNA regulatory mechanisms. The presented results yield mechanistic insight into possible recruitment mechanisms of FUS and EWS to target genes for regulation of cellular pathways at the RNA level, and have identified novel FUS and EWS target genes with potential to be mediators of disease-associated cellular functions.

## Methods

### Biological material

HEK-293 cells were grown in Dulbecco’s Modified Eagle Medium (DMEM) with 10 % Fetal Bovine Serum, streptomycin (2.0 g/l), penicillin (1.2 g/l) and glutamine (0.3 g/l) (complete DMEM). HEK-293 cells were obtained from the American Type Culture Collection (CRL-1573). Cells were grown at 37 °C and 5 % CO_2_. None of the experiments were using primary human material and cells and all experiments were performed in accordance with the declaration of Helsinki and under conditions approved by Danish institutional and governmental legislation.

### Chromatin Immunoprecipitation (ChIP)

The protocol used was merged from [76], [55] and Magnify chromatin immunoprecipitation system (Invitrogen). Briefly, HEK-293 cells (1x10^7^per 10 cm petri dish) were cross-linked directly on plates by adding 37 % formaldehyde to the media in a 1.4 % final concentration for 15 min at room temperature. Cross-linking was stopped by adding glycine (final concentration 125 mM) followed by incubation for 5 min, at room temperature. Cells were scraped off in 500 μl of ice-cold 2x Phosphate Buffered Saline (PBS) and transferred to eppendorf-tubes and placed on ice. Cells were centrifuged at 2,000 g for 5 min, at 4 °C, and washed twice with ice-cold PBS. Cells were lysed in 500 μl of ice-cold immunoprecipitation (IP)-Buffer (150 mM NaCl, 50 mM Tris–HCl (pH 7.5), 5 mM EDTA, 1 % Triton X-100, 0.5 % NP40) supplemented with Complete Mini protease inhibitor cocktail (Roche), hereafter referred to as supplemented IP-Buffer. Samples were centrifuged at 12,000 g for 1 min, at 4 °C, and washed once in ice-cold supplemented IP-Buffer. 500 μl of ice-cold supplemented IP-Buffer were added to the pelleted nuclei and sample re-suspended. Chromatin was fragmented by sonication using a Bioruptor (Diagnode) at high effect (80 cycles, 30s on plus 30s off) to generate fragments of 150–300 bp. Fragment lengths were examined by agarose gel electrophoresis. Samples were centrifuged at 12,000 g for 10 min, at 4 °C. Supernatants were transferred to fresh ice-cold tubes and stored at −80 °C.

For each ChIP experiment, material corresponding to 5x10^6^ starting cells was used. Samples were diluted with an equal volume of D-Buffer (150 mM NaCl, 50 mM Tris–HCl (pH 7.5), 5 mM EDTA, 1 % Triton X-100). 5 μg of antibody (FUS sc-47711 Santa Cruz and EWS sc-48404 Santa Cruz) or 2 μg antibody for acetylated histone protein (ac-H3K9 ab4441-50 Abcam) were used for each ChIP. Samples were incubated in an ultrasonic water bath for 25 min, at 4 °C, and afterwards centrifuged at 12,000 g for 10 min, at 4 °C. 20 μl magnetic AG-beads (Invitrogen) were used for each ChIP. Beads were washed 3 times in 1 ml of supplemented IP-Buffer before use. After the final wash, beads were suspended in 40 μl of supplemented IP-Buffer and mixed with 90 % of the chromatin supernatant described above. The mix was incubated in rotation overnight, at 4 °C. Beads were washed twice with 200 μl of ice-cold supplemented IP-Buffer and three times with 200 μl ice-cold D-Buffer supplemented with protease inhibitors. Cross-linking was reversed by adding Reverse X-link Buffer and Protease K (Invitrogen) and incubating in water bath for 20 min, at 55 °C. The remaining 10 % of the reversed cross-linking chromatin supernatant was used as input. Supernatants were transferred to fresh tubes and incubated at 95 °C to inactivate Protease K. DNA was purified using DNA Purification Buffer plus DNA purification magnetic beads, as recommended (Invitrogen). DNA was stored at −20 °C.

### ChIP-sequencing (ChIP-seq) and bioinformatics

DNA from single FUS, EWS, and Ac-H3K9 ChIP experiment, as well as the corresponding input, was sequenced using the Illumina Hiseq 2000 platform at BGI in Shenzhen, China. Library preparation, cluster generation and sequencing by synthesis were performed according to manufacturer’s protocol. All raw reads were aligned using Burrows-Wheeler Aligner (BWA version 0.5.8c (r1536) [48]) to the human reference genome (hg19). Aligned reads were processed by Model-based Analysis of ChIP-seq (MACS) 1.4.0rc2 [49] for peak calling. Significant peaks were defined using the criteria of a threshold of minimally 9 reads and p-value less than 10^−8^ as suggested previously [50]. Input ChIP DNA was used as negative control. ChIP experiments were independently repeated and ChIP-seq called peaks verified by qPCR.

RNAPII binding sites in HEK293 cells were identified using ChIP-seq data from the ENCODE Project Consortium (PMID: 22955616) [77]. ChIP-seq fastq files were downloaded from http://genome.ucsc.edu/ENCODE/downloads.html. Sequenced reads were mapped to hg19 with Bowtie (PMID: 19261174) allowing one mismatch. Peak calling was performed using the Model-based Analysis of ChIP-seq v1.4 (MACS) (PMID: 18798982) identifying 26,323 peaks.

### FUS and EWS depletion

siRNAs against the FUS and the EWS mRNAs were used as a mix of two siRNAs for each protein (MWG, Germany). siRNA sequences for EWS: 5’-CGAGGAGGAAGGAGAGAAA-3’ and 5’-GAGTAGCTATGGTCAACAA-3’, and for FUS: 5’-ACAGCCCATGATTAATTTGTA-3’ and 5’-GGGAGAAGGCCAAATGATA-3’. Control siRNA is a non-specific siRNA with the sequence 5’-CUGAUGCAGGUAAUCGCGU-3’. 24 h before transfections HEK-293 cells were reseeded in fresh media at 40 % confluence. 2x10^5^ HEK-293-cells were seeded in a 6-well plate and transfected in suspension using Dharmafect-1 (Dharmacon) and 100 nM of final siRNA concentration. Cells were incubated for 48 h before switching to fresh complete DMEM and transfected again using TransIT-siQUEST (Mirus) and 100 nM of final siRNA concentration. Cells were harvested after another 48 h. Transfections were performed in duplicates and pooled upon harvest.

### Western blotting

For ChIP control experiments samples were processed according to the ChIP protocol until the reverse cross-linking step. Beads and input samples were given 2.5x Loading Buffer and Reducing Agent (Fermentas), followed by heating to 95 °C for 5 min. For siRNA mediated depletion experiments samples were processed essential as described previously [17]. Antibodies used were FUS (FUS sc-47711 Santa Cruz) and EWS (EWS sc-48404 Santa Cruz) in 1:2000 dilutions and 4 F4 primary antibody as a loading control in 1:10,000 dilution. All procedures were essential as previously described [17].

### RNA extraction and cDNA synthesis

RNA was extracted using TRI-Reagent (Sigma) according to the manufacturer’s protocol. cDNA was synthesized from 1 μg of total RNA in 20 μl reactions using iScript™ cDNA synthesis Kit (Biorad). After synthesis, the cDNA was diluted five times with double distilled water and stored at −20 °C.

### Quantitative PCR (qPCR)

FUS and EWS peak enrichment validation was performed by qPCR analysis. Briefly, 1 μl ChIP-DNA was used as template with 3 pmol of each primer. Primer sequences are shown in Additional file 19. qPCR analysis was performed on a Lightcycler 480 (Roche). All reactions were done in triplicates in a total volume of 10 μl each using Lightcycler 480 SYBR Green I Master (Roche), according to manufacturer’s instructions. Cycle conditions: 95 °C 10 s, 58 °C 20 s, 72 °C 30 s, 45 repeats. Reaction specificity was confirmed by melting curve analysis and gel electrophoresis. Primer efficiency (above 95 %) was measured by dilution standard curves. The DNA amount was quantified as percent of the amount measured in the input sample using the X_0_-method [78]. For negative control, primers were designed targeting the promoter region of the non-transcribed *IFRG28* gene [79]. For RNA quantifications by RT-qPCR, conditions were similar to above-described. Specificity and primer efficiency were evaluated as described above. The DNA amount was normalized to the expression of the house-keeping genes *GAPDH* (Glyceraldehyde 3-phosphate dehydrogenase) and *TBP* and the relative cDNA levels of each individual gene quantified relative to the amount detected in the control cells treated with the unspecific siRNA using the X_0_-method [78]. EWS, FUS, EWS and FUS siRNA-depleted and control HEK-293 cells were tested for variations of the alternative splicing pattern at the last exon of *HNRNPK* mRNA. A primer set was designed which amplified a fragment of 491 bp corresponding to the proximal splice acceptor site usage and a fragment of 431 bp corresponding to the distal splice acceptor site usage. PCR amplification was performed with a 2720 Thermal Cycler (Applied Biosystems). Amplification was done within the exponential phase (28–30 cycles) to ensure that the PCR reaction was in the linear amplification range. PCR products were run on a 2 % agarose TAE gel with a 100 bp DNA ladder (Bio Labs, Ipswich, Massachusetts) and relative band intensities were quantified by GelQuant.NET software provided by http://biochemlabsolutions.com. All qPCR experiments were done in triplicates.

## Availability of data and materials

Supporting data is included in Additional files 1, 2, 5, 6, 7, 8, 9, 14, 15, 16, 17, 18, and ChIP-seq and chip data available at NCBI Bio projects accession PRJNA185008, NCBI Gene Expression Omnibus (GEO) entry: GSE35578 and GEO entry: GSE73492.

## Additional files

Additional file 1:
**List of significant FUS binding peaks within or close (within 10 kb distance) to annotated genes.** (XLSX 11 kb) Additional file 2:
**List of significant EWS binding peaks within or close (within 10 kb distance) to annotated genes.** (XLSX 15 kb) Additional file 3:
**Annotated genes overlapping the significant enrichment peaks determined from FUS, EWS and Ac-H3K9 ChIP-seq analysis.** Chromosome (CH): the peak start and end in base pairs; Length: the peak length in base pairs; Tags: the number of sequences included in the peak; p-value: p-value on log2 scale; fold-change (fc): relative to input sample; FDR: false discovery rate in percentage; ENS ID: gene ID in the ensemble database; gene location: location of the enrichment in the gene (upstream, downstream, intron, exon); and T: number of known transcript variants produced from the given gene. (PDF 1497 kb) Additional file 4:
**Gene ontology (GO) and Kyoto encyclopedia of genes and genomes (KEGG) analysis by the web based software ChIP-Enrich of FUS and EWS ChIP-seq peaks** [51]. (DOCX 19 kb) Additional file 5:
**qPCR validation of enrichment peaks identified by FUS and EWS ChIP-seq.** The enrichment of DNA was quantified as percentage of the amount in the input sample and the p-values for enrichment of FUS and EWS were calculated. *IFG28* was used as a negative control for enrichment. A control ChIP experiment was also performed with inclusion of pre-immune antiserum added to the AG beads used for chromatin purification (AG) instead of FUS and EWS antibodies. Data represents three independent experiments and standard deviation shown by error bars. **A.** *C19orf48* and *ACPT*; **B.** *RCC1* and *SNHG3*; **C.** *HNRNPK. (PDF 23 kb)*
Additional file 6:
**Cross-comparison of the genes identified to be associated with FUS, EWS or Ac-H3K9 by ChIP-seq analysis.** The displayed overlap categories are FUS and EWS, FUS and Ac-H3K9, EWS and Ac-H3K9, FUS and EWS and Ac-H3K9, FUS and EWS. Chromosome (CH): the peak start and end in base pairs; Length: the peak length in base pairs; Tags: the number of sequences included in the peak; p-value: p-value on log2 scale; fold-change (fc): relative to input sample; FDR: false discovery rate in percent; ENS ID: gene ID in the ensemble database; gene location: location of the enrichment in the gene (upstream, downstream, intron, exon); and T: number of known transcript variants produced from the given gene. (DOCX 325 kb) Additional file 7:
**Expression analyses based on microarray data of genes presented with FUS, EWS, FUS and EWS, and Ac-H3K9 ChIP-seq peaks.** (XLS 70 kb) Additional file 8:
**Microarray data gene probe sets for actively transcribed genes also presented with FUS and Ac-H3K9 (n = 35), EWS and Ac-H3K9 (n = 135), or FUS and EWS and Ac-H3K9 (n = 58) ChIP-seq peaks.** The probe sets were used in GO analysis. (XLSX 41 kb) Additional file 9:
**List of FUS and EWS ChIP-seq peaks assigned with a position downstream the poly(A)-signal of a target gene.** (XLSX 14 kb) Additional file 10:
**Consequences of siRNA mediated depletion of FUS and EWS for gene expression.** HEK-293 cells were double-transfected with specific siRNAs for FUS, EWS and FUS plus EWS, and as well as with a control siRNA (siControl). **A.** siRNA-depleted cells were used for relative mRNA quantification of FUS and EWS by qPCR. FUS and EWS expression was normalized to reference gene TBP. Experiments were performed in triplicates. Data presented as mean + SEM. Similar qPCR expression analyses showed a 1.5 to 2 fold increase in TAF15 mRNA by EWS and FUS depletion (not shown). **B**. Protein quantification by western blot of FUS and EWS from siRNA and control transfected cells. 4 F4 antibody recognizing HNRNP C1 + C2 was used as a loading control. (PDF 276 kb) Additional file 11:
**Analysis of ChIP-seq FUS and EWS enrichment peaks in the** ***ACPT*** **and** ***C19orf48*** **gene complex.** **A.** Graphic distribution of ChIP-seq reads aligned to the *ACPT* and *C19orf48* genes from the input, FUS, EWS and Ac-H3K9 ChIP-seq samples. The number of reads is shown on the scale to the left of each figure. The transcripts from the genes in the UCSC hg19 genomic database are shown in the bottom. The arrows beneath peaks illustrate location of the several qPCR amplicons used. The arrows above the transcripts illustrate location of RT-qPCR amplicons. Numbers above arrows denotes target exon numbers. **B-C**. FUS and EWS effect in *ACPT* and *C19orf48* expression. The expression levels of *C19orf48* transcripts were determined by qPCR from HEK-293 cells transfected with siRNA for FUS and EWS or control siRNA. Three independent experiments were performed and error bars indicate standard deviation. In **B.** simultaneous depletion of FUS and EWS was performed, whereas in **C.** FUS and EWS were individually depleted. (PDF 483 kb) Additional file 12:
**Analysis of ChIP-seq FUS and EWS enrichment peaks in the** ***RCC1*** **and** ***SNHG3*** **gene complex, including** ***SNORA73A*** **and** ***SNORA73B*** **genes.** **A.** Graphic distribution of ChIP-seq reads aligned to the *RCC1* and *SNHG3* genes from the input, FUS, EWS and Ac-H3K9 ChIP-seq samples. The number of reads is shown on the scale to the left of each figure. The transcripts from the genes in the UCSC hg19 genomic database are shown in the bottom. The arrows beneath peaks illustrate location of the several qPCR amplicons used. The arrows above the transcripts illustrate location of RT-qPCR amplicons. Numbers above arrows denotes target exon numbers. **B-C**. FUS and EWS effect for *RCC1*, *SNHG3* and *snoRNA* expression. The expression levels were determined by qPCR from HEK-293 cells transfected with siRNA for FUS and EWS or control siRNA. Three independent experiments were performed and error bars indicate standard deviation. In **B**., simultaneous depletion of FUS and EWS was performed, whereas in **C.** FUS and EWS were individually depleted. (PDF 552 kb) Additional file 13:
**FUS and EWS regulate RNA processing in the 3’-end of** ***HNRNPK*** **.** **A.** Graphic distribution of ChIP-seq reads aligned to *HNRNPK*. The number of reads is shown on the scale to the left of each figure. The transcripts from the genes in the UCSC hg19 genomic database are shown in the bottom. The arrows beneath peaks illustrate location of qPCR amplicons. The arrows above the transcripts illustrate location of RT-qPCR amplicons. Numbers above arrows denote target exon numbers. **B-C.** The expression levels were determined by qPCR from HEK-293 cells transfected with siRNA for FUS and EWS or control siRNA. In B, simultaneous depletion of FUS and EWS was performed, whereas in C FUS and EWS were individually depleted. All values were normalized to reference gene *TBP*. All experiments were performed in triplicates. **P* < 0.05; ***P* < 0.01. Data presented as mean + SEM. **D-E**. FUS and EWS involvement in the production of *miR7-1* from *HNRNPK* intron 15. By RT-qPCR the relative amounts of mature *miR7*, *premiR7-1* and *pri-miR7-1* were determined by RT-qPCR from HEK-293 cells transfected with siRNA for FUS and EWS or control siRNA. In **D.**, simultaneous depletion of FUS and EWS was performed, whereas in **E.** FUS and EWS were individually depleted Values were normalized to reference gene *RNU48*. ***P* < 0.01, ****P* < 0.001, *****P* < 0.0001. All experiments were performed in triplicates. Data presented as mean + SEM. (PDF 549 kb) Additional file 14:
**Comparative analysis and alignment to the human genome (UCSC hg19) of raw FUS ChIP-seq reads from this study and recalculated from the few data files presented in the study by Schwartz et al.** [30]. (DOCX 16 kb) Additional file 15:
**Comparative distribution of p-values for ChIP-seq peaks called by FindPeaks and MACS of data from this study and the study by Schwarts et al.** [30]. A. Distribution of p-values for ChIP-seq peaks called by FindPeaks (F). Most of the ChIP-seq peaks from the hereby presented FUS ChIP-seq data (unFUS_JB) have a low p-value. Most of the ChIP-seq peaks from Schwartz’s et al. (unFUS_ref) have a high P-value. B. Distribution of p-values for ChIP-seq peaks called by MACS. A lower number of ChIP-seq peaks were identified compared to with usage of FindPeaks and the p-value distribution is more comparable between the two datasets. (DOCX 106 kb) Additional file 16:
**Comparison of the distribution of MACS called ChIP-seq peaks across a gene model using data from this study or the study by Schwarts et al.** [30]. **A.** ChIP-seq data from this study for FUS (unFUS-JB) and **B.** ChIP-seq data from Schwarts et al. for FUS from cells not pretreated with FUS siRNA (unFUS-ref). Cut-off levels for P-values are indicated to the left for each figure panel. Data were plotted using gene modelling with number of hits, which represents the number of annotated UCSC transcripts corresponding to the position of a given ChIP-seq peak, shown at the y-axis. The gene model presented on the x-axis is based on the following features: position 1 – 100: UP, 10 kb upstream region of the transcription start site of coding genes; NCUp, 10 kb upstream region of the transcription start site of noncoding genes; Position 100–200: U5Exon, 5’ untranslated exon region; U5Intron, 5’ intron region upstream of the translation start site; Position 200–300: Exon, Coding region of the exon sequences; Intron, Intron region between the translation start site and stop site; NCExon, Exon region of noncoding genes; NCIntron, Intron region of noncoding genes; Position 300–400: U3Exon, 3’ untranslated exon region; U3Intron, 3’ intron region downstream of the translation stop site; Position 400–500: Down, 10 kb downstream region of the poly(A)-signal of coding genes; UCDown, 10 kb downstream region of the transcription poly(A) of noncoding genes. The features were subdivided in smaller segments for precision mapping. **C.** MACS peak output from Schwarts et al. data of the exemplified *ACPT* and *C19ORF48*, *SNHG3* and *RCC1* and *HNRNPK* genes. (DOCX 842 kb) Additional file 17:
**Comparison of the distribution of FindPeaks called ChIP-seq peaks across a model gene using data from this study or the study by Schwarts et al.** [30]. For **A.** and **B.** legend details see Additional file 16. (DOCX 693 kb) Additional file 18:
**Genes co-identified in the hereby presented ChIP-seq data and RNA-CLIP from Hoell et al.** [26]. (DOCX 37 kb) Additional file 19:
**List of primer sequences for PCR.** (DOCX 20 kb)
