# Supplementary material for: EWS and FUS bind a subset of transcribed genes encoding proteins enriched in RNA regulatory functions
Source: BMC Genomics. 2015 Nov 14;16:929. doi: 10.1186/s12864-015-2125-9 (PMC4647676; doi:10.1186/s12864-015-2125-9)
Supplement: Additional file 3: — Annotated genes overlapping the significant enrichment peaks determined from FUS, EWS and Ac-H3K9 ChIP-seq analysis. Chromosome (CH): the peak start and end in base pairs; Length: the peak length in base pairs; Tags: the number of sequences included in the peak; p-value: p-value on log2 scale; fold-change (fc): relative to input sample; FDR: false discovery rate in percentage; ENS ID: gene ID in the ensemble database; gene location: location of the enrichment in the gene (upstream, downstream, intron, exon); and T: number of known transcript variants produced from the given gene. (PDF 1497 kb) [file 12864_2015_2125_MOESM3_ESM.pdf]

Additional file 3. Annotated genes to the significant peaks in the FUS, EWS, and Ac-H3K9 ChIP samples.

Sample	Ch.	peak start	peak end	length	tags	p-value	F.C.	FDR	ENS ID	Gene name	location	T
FUS	7	2.62E+07	2.62E+07	3011	95	504.94	15.2	100	ENSG00000050344	NFE2L3	Down	2
FUS	7	2.62E+07	2.62E+07	2147	51	201.43	9.2	100	ENSG00000050344	NFE2L3	Exon	2
FUS	1	7.63E+07	7.63E+07	1524	39	179.54	9.5	100	ENSG00000057468	MSH4	Up	1
FUS	2	1.02E+08	1.02E+08	1037	18	84.45	7.5	100	ENSG00000071082	RPL31	Intron	11
FUS	16	7.43E+07	7.43E+07	692	13	88.91	9.7	100	ENSG00000103035	PSMD7	Exon	2
FUS	2	8.58E+07	8.58E+07	1733	42	180.31	9.5	100	ENSG00000115486	GGCX	Down	10
FUS	2	8.82E+06	8.82E+06	611	14	96.3	12.5	100	ENSG00000115738	ID2	Exon	4
FUS	2	1.78E+08	1.78E+08	1785	33	154.27	8.0	100	ENSG00000116044	NFE2L2	Down	12
FUS	1	6.82E+07	6.82E+07	691	18	84.49	9.2	100	ENSG00000116717	GADD45A	Down	4
FUS	1	7.63E+07	7.63E+07	1524	39	179.54	9.5	100	ENSG00000117054	ACADM	Down	19
FUS	1	1.74E+08	1.74E+08	1673	47	251.36	11.5	100	ENSG00000117593	DARS2	Down	4
FUS	13	7.44E+07	7.44E+07	1121	24	82.35	7.6	100	ENSG00000118922	KLF12	Intron	4
FUS	17	5.68E+07	5.68E+07	988	26	107.78	10.7	100	ENSG00000121101	TEX14	Intron	3
FUS	16	1.19E+07	1.19E+07	302	9	87.29	11.3	100	ENSG00000122299	ZC3H7A	Intron	2
FUS	7	2.62E+07	2.62E+07	3011	95	504.94	15.2	100	ENSG00000122566	HNRNPA2B1	Down	8
FUS	7	2.62E+07	2.62E+07	2147	51	201.43	9.2	100	ENSG00000122566	HNRNPA2B1	Down	8
FUS	17	7.48E+06	7.48E+06	726	13	86	12.9	100	ENSG00000129194	SOX15	Down	2
FUS	17	7.48E+06	7.48E+06	726	13	86	12.9	100	ENSG00000129226	CD68	Up	2
FUS	17	7.48E+06	7.48E+06	726	13	86	12.9	100	ENSG00000129255	MPDU1	Up	5
FUS	19	1.02E+07	1.02E+07	1072	21	142.23	13.9	100	ENSG00000130810	PPAN	Intron	10
FUS	19	1.02E+07	1.02E+07	1072	21	142.23	13.9	100	ENSG00000130811	EIF3G	Down	1
FUS	5	1.77E+08	1.77E+08	661	15	84.47	12.1	100	ENSG00000131188	PRR7	Up	5
FUS	13	4.59E+07	4.59E+07	1393	32	106.51	9.3	100	ENSG00000133112	TPT1	Down	13
FUS	6	7.42E+07	7.42E+07	1387	32	107.03	7.1	100	ENSG00000135297	MTO1	Down	20
FUS	13	7.59E+07	7.59E+07	1139	27	85.31	6.9	100	ENSG00000136111	TBC1D4	Intron	8
FUS	8	1.29E+08	1.29E+08	1533	31	89.81	7.9	100	ENSG00000136997	MYC	Down	6
FUS	9	1.94E+07	1.94E+07	1232	38	148.76	9.4	100	ENSG00000137145	DENND4C	Exon	10
FUS	9	1.94E+07	1.94E+07	1232	38	148.76	9.4	100	ENSG00000137154	RPS6	Down	5
FUS	11	7.26E+07	7.26E+07	520	13	85.01	12.5	100	ENSG00000137478	FCHSD2	Intron	10
FUS	1	7.63E+07	7.63E+07	1524	39	179.54	9.5	100	ENSG00000137955	RABGGTB	Down	16
FUS	19	5.13E+07	5.13E+07	1001	20	139.43	12.1	100	ENSG00000142513	ACPT	Exon	2
FUS	19	5.13E+07	5.13E+07	837	14	90.31	12.1	100	ENSG00000142513	ACPT	Intron	2
FUS	4	8.33E+07	8.33E+07	1671	37	155.59	7.4	100	ENSG00000145293	ENOPH1	Up	4
FUS	5	4.08E+07	4.08E+07	1760	34	108.99	8.0	100	ENSG00000145592	RPL37	Exon	6
FUS	5	1.81E+08	1.81E+08	1656	49	202.06	10.4	100	ENSG00000146063	TRIM41	Down	11
FUS	4	8.33E+07	8.33E+07	1671	37	155.59	7.4	100	ENSG00000152795	HNRPDL	Exon	5
FUS	6	7.42E+07	7.42E+07	1387	32	107.03	7.1	100	ENSG00000156508	EEF1A1	Down	12
FUS	3	1.87E+08	1.87E+08	596	14	91.11	12.5	100	ENSG00000156976	EIF4A2	Down	28
FUS	17	7.48E+06	7.48E+06	726	13	86	12.9	100	ENSG00000161956	SENP3	Down	2
FUS	17	7.48E+06	7.48E+06	726	13	86	12.9	100	ENSG00000161960	EIF4A1	Down	3
FUS	1	7.84E+07	7.84E+07	1856	41	107.89	6.1	100	ENSG00000162613	FUBP1	Down	16
FUS	1	7.84E+07	7.84E+07	1856	41	107.89	6.1	100	ENSG00000162614	NEXN	Intron	10
FUS	3	1.87E+08	1.87E+08	596	14	91.11	12.5	100	ENSG00000163918	RFC4	Exon	14
FUS	9	8.66E+07	8.66E+07	722	16	91.77	9.5	100	ENSG00000165118	C9orf64	Up	3
FUS	9	8.66E+07	8.66E+07	722	16	91.77	9.5	100	ENSG00000165119	HNRNPK	Down	15
FUS	14	3.49E+07	3.49E+07	1015	18	86.05	7.5	100	ENSG00000165389	C14orf147	Intron	1
FUS	19	5.13E+07	5.13E+07	1001	20	139.43	12.1	100	ENSG00000167747	C19orf48	Down	2
FUS	19	5.13E+07	5.13E+07	837	14	90.31	12.1	100	ENSG00000167747	C19orf48	Down	2
FUS	2	8.58E+07	8.58E+07	1733	42	180.31	9.5	100	ENSG00000168906	MAT2A	Down	7
FUS	2	1.78E+08	1.78E+08	1785	33	154.27	8.0	100	ENSG00000170144	HNRNPA3	Down	8
FUS	13	4.59E+07	4.59E+07	1393	32	106.51	9.3	100	ENSG00000170919	XXyac-R12DG2.2	Up	21
FUS	1	2.88E+07	2.88E+07	1523	77	409.11	15.0	100	ENSG00000180198	RCC1	Intron	12
FUS	1	2.88E+07	2.88E+07	1267	36	150.89	7.1	100	ENSG00000180198	RCC1	Intron	12
FUS	22	4.30E+07	4.30E+07	562	12	84.45	15.0	100	ENSG00000182841	RP1-222E13.10	Intron	4
FUS	22	4.30E+07	4.30E+07	562	12	84.45	15.0	100	ENSG00000183569	SERHL2	Intron	11
FUS	1	1.74E+08	1.74E+08	1673	47	251.36	11.5	100	ENSG00000185278	ZBTB37	Up	7
FUS	5	1.77E+08	1.77E+08	661	15	84.47	12.1	100	ENSG00000198055	GRK6	Down	11
FUS	13	9.20E+07	9.20E+07	2989	72	179.39	6.7	100	ENSG00000199149	MIR20A	Down	1
FUS	13	9.20E+07	9.20E+07	1954	55	125.65	6.7	100	ENSG00000199149	MIR20A	Down	1
FUS	13	9.20E+07	9.20E+07	2877	82	211.85	6.4	100	ENSG00000199149	MIR20A	Down	1
FUS	13	9.20E+07	9.20E+07	2989	72	179.39	6.7	100	ENSG00000199180	MIR18A	Down	1
FUS	13	9.20E+07	9.20E+07	1954	55	125.65	6.7	100	ENSG00000199180	MIR18A	Down	1
FUS	13	9.20E+07	9.20E+07	2877	82	211.85	6.4	100	ENSG00000199180	MIR18A	Down	1
FUS	13	4.59E+07	4.59E+07	1393	32	106.51	9.3	100	ENSG00000199477	SNORA31	Down	1
FUS	1	1.74E+08	1.74E+08	1673	47	251.36	11.5	100	ENSG00000200016	SNORD76	Down	1

FUS	1	2.88E+07	2.88E+07	1523	77	409.11	15.0	100	ENSG00000200087	SNORA73B	Down	1
FUS	1	2.88E+07	2.88E+07	1267	36	150.89	7.1	100	ENSG00000200087	SNORA73B	Down	1
FUS	17	1.90E+07	1.90E+07	439	13	113.36	15.2	100	ENSG00000200229	SNORD3B-1	Down	1
FUS	3	1.87E+08	1.87E+08	596	14	91.11	12.5	100	ENSG00000200320	SNORA63	Down	1
FUS	3	1.87E+08	1.87E+08	596	14	91.11	12.5	100	ENSG00000200418	SNORA63.6	Down	1
FUS	1	1.74E+08	1.74E+08	1673	47	251.36	11.5	100	ENSG00000200710	SNORD81	Down	1
FUS	1	1.74E+08	1.74E+08	1673	47	251.36	11.5	100	ENSG00000200729	SNORD79	Down	1
FUS	1	1.74E+08	1.74E+08	1673	47	251.36	11.5	100	ENSG00000200954	SNORD74	Down	1
FUS	17	5.68E+07	5.68E+07	988	26	107.78	10.7	100	ENSG00000200997	U1.36	Down	1
FUS	1	7.63E+07	7.63E+07	1524	39	179.54	9.5	100	ENSG00000201487	SNORD45B	Down	1
FUS	1	1.74E+08	1.74E+08	1673	47	251.36	11.5	100	ENSG00000201692	SNORD80	Down	1
FUS	17	1.90E+07	1.90E+07	439	13	113.36	15.2	100	ENSG00000201750	SNORD3B-2	Exon	1
FUS	1	2.88E+07	2.88E+07	1523	77	409.11	15.0	100	ENSG00000201808	SNORA73A	Down	1
FUS	1	2.88E+07	2.88E+07	1267	36	150.89	7.1	100	ENSG00000201808	SNORA73A	Down	1
FUS	1	1.74E+08	1.74E+08	1673	47	251.36	11.5	100	ENSG00000202394	SNORD47	Down	1
FUS	1	2.88E+07	2.88E+07	1523	77	409.11	15.0	100	ENSG00000204138	PHACTR4	Down	5
FUS	6	3.29E+07	3.30E+07	1829	50	226.58	11.6	100	ENSG00000204256	BRD2	Down	16
FUS	5	1.81E+08	1.81E+08	1656	49	202.06	10.4	100	ENSG00000204628	GNB2L1	Down	35
FUS	2	1.02E+08	1.02E+08	1037	18	84.45	7.5	100	ENSG00000204634	TBC1D8	Exon	10
FUS	19	4.37E+07	4.37E+07	4008	167	605.17	11.8	100	ENSG00000204941	PSG5	Up	9
FUS	1	1.74E+08	1.74E+08	1673	47	251.36	11.5	100	ENSG00000206607	SNORD44	Down	1
FUS	1	7.63E+07	7.63E+07	1524	39	179.54	9.5	100	ENSG00000206620	SNORD45C	Down	1
FUS	17	5.68E+07	5.68E+07	988	26	107.78	10.7	100	ENSG00000206917	U1.78	Up	1
FUS	17	7.48E+06	7.48E+06	726	13	86	12.9	100	ENSG00000207152	SNORA67	Down	1
FUS	1	7.63E+07	7.63E+07	1524	39	179.54	9.5	100	ENSG00000207241	SNORD45A	Down	1
FUS	13	9.20E+07	9.20E+07	2989	72	179.39	6.7	100	ENSG00000207560	MIR19B1	Down	1
FUS	13	9.20E+07	9.20E+07	1954	55	125.65	6.7	100	ENSG00000207560	MIR19B1	Down	1
FUS	13	9.20E+07	9.20E+07	2877	82	211.85	6.4	100	ENSG00000207560	MIR19B1	Down	1
FUS	9	8.66E+07	8.66E+07	722	16	91.77	9.5	100	ENSG00000207603	MIR7-1	Down	1
FUS	13	9.20E+07	9.20E+07	2989	72	179.39	6.7	100	ENSG00000207610	MIR19A	Down	1
FUS	13	9.20E+07	9.20E+07	1954	55	125.65	6.7	100	ENSG00000207610	MIR19A	Down	1
FUS	13	9.20E+07	9.20E+07	2877	82	211.85	6.4	100	ENSG00000207610	MIR19A	Down	1
FUS	13	9.20E+07	9.20E+07	2877	82	211.85	6.4	100	ENSG00000207745	MIR17	Down	1
FUS	13	9.20E+07	9.20E+07	2989	72	179.39	6.7	100	ENSG00000207745	MIR17	Down	1
FUS	13	9.20E+07	9.20E+07	1954	55	125.65	6.7	100	ENSG00000207745	MIR17	Down	1
FUS	13	9.20E+07	9.20E+07	2989	72	179.39	6.7	100	ENSG00000207968	MIR92A1	Down	1
FUS	13	9.20E+07	9.20E+07	1954	55	125.65	6.7	100	ENSG00000207968	MIR92A1	Down	1
FUS	13	9.20E+07	9.20E+07	2877	82	211.85	6.4	100	ENSG00000207968	MIR92A1	Down	1
FUS	1	1.74E+08	1.74E+08	1673	47	251.36	11.5	100	ENSG00000208310	SNORD75	Down	1
FUS	1	1.74E+08	1.74E+08	1673	47	251.36	11.5	100	ENSG00000208313	SNORD77	Down	1
FUS	1	1.74E+08	1.74E+08	1673	47	251.36	11.5	100	ENSG00000208317	SNORD78	Down	1
FUS	5	1.81E+08	1.81E+08	1656	49	202.06	10.4	100	ENSG00000208342	SNORD96A	Down	1
FUS	17	7.48E+06	7.48E+06	726	13	86	12.9	100	ENSG00000209582	SNORA48	Down	1
FUS	19	1.02E+07	1.02E+07	1072	21	142.23	13.9	100	ENSG00000209645	SNORD105	Down	1
FUS	5	4.08E+07	4.08E+07	1760	34	108.99	8.0	100	ENSG00000212296	SNORD72	Down	1
FUS	13	9.20E+07	9.20E+07	2989	72	179.39	6.7	100	ENSG00000215417	MIR17HG	Down	1
FUS	13	9.20E+07	9.20E+07	1954	55	125.65	6.7	100	ENSG00000215417	MIR17HG	Down	1
FUS	13	9.20E+07	9.20E+07	2877	82	211.85	6.4	100	ENSG00000215417	MIR17HG	Down	1
FUS	19	5.13E+07	5.13E+07	1001	20	139.43	12.1	100	ENSG00000220988	SNORD88C	Down	1
FUS	19	5.13E+07	5.13E+07	837	14	90.31	12.1	100	ENSG00000220988	SNORD88C	Down	1
FUS	19	5.13E+07	5.13E+07	837	14	90.31	12.1	100	ENSG00000221233	AC010325.2	Down	1
FUS	19	5.13E+07	5.13E+07	1001	20	139.43	12.1	100	ENSG00000221233	AC010325.2	Down	1
FUS	19	5.13E+07	5.13E+07	1001	20	139.43	12.1	100	ENSG00000221241	SNORD88A	Down	1
FUS	19	5.13E+07	5.13E+07	837	14	90.31	12.1	100	ENSG00000221241	SNORD88A	Down	1
FUS	19	5.13E+07	5.13E+07	1001	20	139.43	12.1	100	ENSG00000221381	SNORD88B	Down	1
FUS	19	5.13E+07	5.13E+07	837	14	90.31	12.1	100	ENSG00000221381	SNORD88B	Down	1
FUS	3	1.87E+08	1.87E+08	596	14	91.11	12.5	100	ENSG00000221420	SNORA81	Down	1
FUS	2	1.02E+08	1.02E+08	1037	18	84.45	7.5	100	ENSG00000223947	AC016738.4	Up	1
FUS	3	8.63E+06	8.63E+06	1073	24	80.98	6.4	100	ENSG00000224884	AC034187.2	Intron	1
FUS	2	1.78E+08	1.78E+08	1785	33	154.27	8.0	100	ENSG00000225808	AC079305.9	Down	1
FUS	3	8.63E+06	8.63E+06	1073	24	80.98	6.4	100	ENSG00000227110	AC087859.1	Intron	7
FUS	6	7.42E+07	7.42E+07	1387	32	107.03	7.1	100	ENSG00000229862	RP11-505P4.7	Up	2
FUS	1	1.74E+08	1.74E+08	1673	47	251.36	11.5	100	ENSG00000231792	RP5-1198E17.1	Up	1
FUS	17	7.48E+06	7.48E+06	726	13	86	12.9	100	ENSG00000233223	AC113189.5	Down	2
FUS	5	1.81E+08	1.81E+08	1656	49	202.06	10.4	100	ENSG00000233937	CTC-338M12.4	Up	4
FUS	1	1.74E+08	1.74E+08	1673	47	251.36	11.5	100	ENSG00000234741	GAS5	Down	29
FUS	9	1.94E+07	1.94E+07	1232	38	148.76	9.4	100	ENSG00000234853	RP11-513M16.5	Down	1
FUS	2	8.82E+06	8.82E+06	611	14	96.3	12.5	100	ENSG00000235092	AC011747.7	Up	7

FUS	9	8.66E+07	8.66E+07	722	16	91.77	9.5	100	ENSG00000235298	RP11-575L7.8	Up	1
FUS	19	1.02E+07	1.02E+07	1072	21	142.23	13.9	100	ENSG00000238531	SNORD105B	Down	1
FUS	1	2.88E+07	2.88E+07	1523	77	409.11	15.0	100	ENSG00000238821	snoU13.263	Up	1
FUS	1	2.88E+07	2.88E+07	1267	36	150.89	7.1	100	ENSG00000238821	snoU13.263	Up	1
FUS	17	7.48E+06	7.48E+06	726	13	86	12.9	100	ENSG00000238917	SNORD10	Down	1
FUS	3	1.87E+08	1.87E+08	596	14	91.11	12.5	100	ENSG00000238942	snR39B.2	Down	1
FUS	13	9.20E+07	9.20E+07	2989	72	179.39	6.7	100	ENSG00000239594	RP11-282D2.3	Down	1
FUS	13	9.20E+07	9.20E+07	1954	55	125.65	6.7	100	ENSG00000239594	RP11-282D2.3	Down	1
FUS	13	9.20E+07	9.20E+07	2877	82	211.85	6.4	100	ENSG00000239594	RP11-282D2.3	Down	1
FUS	13	9.20E+07	9.20E+07	2989	72	179.39	6.7	100	ENSG00000240532	RP11-282D2.5	Down	1
FUS	13	9.20E+07	9.20E+07	1954	55	125.65	6.7	100	ENSG00000240532	RP11-282D2.5	Down	1
FUS	13	9.20E+07	9.20E+07	2877	82	211.85	6.4	100	ENSG00000240532	RP11-282D2.5	Down	1
FUS	13	9.20E+07	9.20E+07	2877	82	211.85	6.4	100	ENSG00000242008	RP11-282D2.2	Down	1
FUS	13	9.20E+07	9.20E+07	2989	72	179.39	6.7	100	ENSG00000242008	RP11-282D2.2	Down	1
FUS	13	9.20E+07	9.20E+07	1954	55	125.65	6.7	100	ENSG00000242008	RP11-282D2.2	Down	1
FUS	1	2.88E+07	2.88E+07	1523	77	409.11	15.0	100	ENSG00000242125	SNHG3	Down	3
FUS	1	2.88E+07	2.88E+07	1267	36	150.89	7.1	100	ENSG00000242125	SNHG3	Down	3
FUS	13	9.20E+07	9.20E+07	2989	72	179.39	6.7	100	ENSG00000243108	RP11-282D2.6	Down	1
FUS	13	9.20E+07	9.20E+07	1954	55	125.65	6.7	100	ENSG00000243108	RP11-282D2.6	Down	1
FUS	13	9.20E+07	9.20E+07	2877	82	211.85	6.4	100	ENSG00000243108	RP11-282D2.6	Down	1
FUS	19	4.37E+07	4.37E+07	4008	167	605.17	11.8	100	ENSG00000243137	PSG4	Down	6
FUS	19	1.02E+07	1.02E+07	1072	21	142.23	13.9	100	ENSG00000243207	PPAN-P2RY11	Intron	2
FUS	13	9.20E+07	9.20E+07	2989	72	179.39	6.7	100	ENSG00000243839	RP11-282D2.4	Down	1
FUS	13	9.20E+07	9.20E+07	1954	55	125.65	6.7	100	ENSG00000243839	RP11-282D2.4	Down	1
FUS	13	9.20E+07	9.20E+07	2877	82	211.85	6.4	100	ENSG00000243839	RP11-282D2.4	Down	1
FUS	19	1.02E+07	1.02E+07	1072	21	142.23	13.9	100	ENSG00000244165	P2RY11	Intron	2
FUS	1	2.88E+07	2.88E+07	1267	36	150.89	7.1	100	ENSG00000245178	AL513497.1	Up	1
FUS	1	2.88E+07	2.88E+07	1523	77	409.11	15.0	100	ENSG00000245178	AL513497.1	Up	1
FUS	5	1.77E+08	1.77E+08	661	15	84.47	12.1	100	ENSG00000246334	RP11-1334A24.4	Intron	4
FUS	5	1.81E+08	1.81E+08	1656	49	202.06	10.4	100	ENSG00000247049	CTC-338M12.7	Up	1
FUS	5	1.77E+08	1.77E+08	661	15	84.47	12.1	100	ENSG00000248342	AC145098.1	Down	1
FUS	8	1.29E+08	1.29E+08	1533	31	89.81	7.9	100	ENSG00000249375	RP11-1136L8.1	Up	3
FUS	13	4.59E+07	4.59E+07	1393	32	106.51	9.3	100	ENSG00000253051	SNORA31.25	Down	1
FUS	13	4.59E+07	4.59E+07	1393	32	106.51	9.3	100	ENSG00000255137	RP11-290D2.4	Down	1
FUS	8	1.29E+08	1.29E+08	1533	31	89.81	7.9	100	ENSG00000256129	AC103819.1	Down	1
FUS	1	2.88E+07	2.88E+07	1267	36	150.89	7.1	100	ENSG00000256960	AL513497.2	Down	1
FUS	1	2.88E+07	2.88E+07	1523	77	409.11	15.0	100	ENSG00000256960	AL513497.2	Down	1
EWS	3	1.70E+08	1.70E+08	826	15	86.92	15.7	65.7	ENSG00000008952	SEC62	Exon	12
EWS	6	1.37E+08	1.37E+08	1242	30	107.98	7.1	82.8	ENSG00000029363	BCLAF1	Down	23
EWS	6	1.37E+08	1.37E+08	1227	31	86.63	7.2	65.1	ENSG00000029363	BCLAF1	Down	23
EWS	7	2.62E+07	2.62E+07	742	24	131.86	9.2	100	ENSG00000050344	NFE2L3	Exon	2
EWS	7	2.62E+07	2.62E+07	4900	178	968.63	20.3	100	ENSG00000050344	NFE2L3	Exon	2
EWS	7	2.62E+07	2.62E+07	1155	26	85.75	6.4	63.3	ENSG00000050344	NFE2L3	Intron	2
EWS	1	7.63E+07	7.63E+07	2217	73	285.41	8.1	100	ENSG00000057468	MSH4	Intron	1
EWS	15	6.08E+07	6.08E+07	715	17	128.12	20.4	100	ENSG00000069667	RORA	Intron	4
EWS	17	7.47E+07	7.47E+07	1001	19	100.74	9.5	75.3	ENSG00000070495	JMJD6	Up	5
EWS	17	7.47E+07	7.47E+07	1140	21	80.16	7.0	56.1	ENSG00000070495	JMJD6	Up	5
EWS	1	2.28E+08	2.28E+08	1688	32	141.7	7.6	100	ENSG00000081692	JMJD4	Down	5
EWS	9	3.30E+07	3.30E+07	1235	25	108.9	8.2	85.3	ENSG00000086061	DNAJA1	Down	5
EWS	19	5.00E+07	5.00E+07	1490	35	115.84	8.6	86	ENSG00000090554	FLT3LG	Down	2
EWS	17	7.47E+07	7.47E+07	1140	21	80.16	7.0	56.1	ENSG00000092931	MFSD11	Up	2
EWS	17	7.47E+07	7.47E+07	1001	19	100.74	9.5	75.3	ENSG00000092931	MFSD11	Up	2
EWS	12	5.47E+07	5.47E+07	1207	31	117.63	9.3	93.3	ENSG00000094916	CBX5	Up	2
EWS	10	1.02E+08	1.02E+08	719	18	95.23	10.0	69.9	ENSG00000099194	SCD	Exon	2
EWS	22	2.01E+07	2.01E+07	933	17	106	11.7	81.8	ENSG00000099901	RANBP1	Down	15
EWS	22	2.01E+07	2.01E+07	933	17	106	11.7	81.8	ENSG00000099904	ZDHHC8	Up	7
EWS	22	3.89E+07	3.89E+07	1598	41	137.79	6.7	100	ENSG00000100201	DDX17	Down	14
EWS	9	3.78E+07	3.78E+07	728	15	91.64	13.5	66	ENSG00000107371	EXOSC3	Up	8
EWS	2	8.58E+07	8.58E+07	1036	21	87.99	8.0	63.7	ENSG00000115486	GGCX	Down	10
EWS	2	8.58E+07	8.58E+07	1050	18	87.16	7.9	64.4	ENSG00000115486	GGCX	Down	10
EWS	2	1.78E+08	1.78E+08	3253	92	154.27	7.3	100	ENSG00000116044	NFE2L2	Down	12
EWS	1	7.63E+07	7.63E+07	2217	73	285.41	8.1	100	ENSG00000117054	ACADM	Down	19
EWS	1	1.74E+08	1.74E+08	1261	34	156.97	10.3	100	ENSG00000117593	DARS2	Down	4
EWS	17	5.68E+07	5.68E+07	1103	35	156.68	13.6	100	ENSG00000121101	TEX14	Intron	3
EWS	17	5.67E+07	5.67E+07	810	17	116.76	13.3	93.5	ENSG00000121101	TEX14	Intron	3
EWS	7	2.62E+07	2.62E+07	4900	178	968.63	20.3	100	ENSG00000122566	HNRNPA2B1	Down	8
EWS	7	2.62E+07	2.62E+07	742	24	131.86	9.2	100	ENSG00000122566	HNRNPA2B1	Down	8
EWS	7	2.62E+07	2.62E+07	1155	26	85.75	6.4	63.3	ENSG00000122566	HNRNPA2B1	Down	8

EWS	9	3.30E+07	3.30E+07	1235	25	108.9	8.2	85.3	ENSG00000122692	SMU1	Down	2
EWS	9	3.78E+07	3.78E+07	728	15	91.64	13.5	66	ENSG00000122741	DCAF10	Intron	5
EWS	12	5.47E+07	5.47E+07	1207	31	117.63	9.3	93.3	ENSG00000123405	NFE2	Down	3
EWS	20	3.43E+07	3.43E+07	613	14	83.93	11.8	60.7	ENSG00000125995	ROMO1	Down	5
EWS	7	1.30E+08	1.30E+08	441	12	92.47	13.6	68.1	ENSG00000128607	KLHDC10	Intron	4
EWS	14	3.50E+07	3.50E+07	969	19	97.1	11.3	72.5	ENSG00000129515	SNX6	Down	4
EWS	19	5.70E+06	5.70E+06	1226	20	127.85	11.9	100	ENSG00000130255	RPL36	Down	2
EWS	20	3.43E+07	3.43E+07	613	14	83.93	11.8	60.7	ENSG00000131051	RBM39	Down	45
EWS	5	1.77E+08	1.77E+08	1116	32	209.84	20.0	100	ENSG00000131188	PRR7	Up	5
EWS	6	7.42E+07	7.42E+07	1322	34	108.22	8.8	84.1	ENSG00000135297	MTO1	Down	20
EWS	11	3.64E+07	3.64E+07	1198	24	120.03	9.6	95.5	ENSG00000135362	PRR5L	Intron	21
EWS	12	5.47E+07	5.47E+07	1207	31	117.63	9.3	93.3	ENSG00000135486	HNRNPA1	Down	3
EWS	17	6.22E+07	6.22E+07	1487	38	162.5	9.1	100	ENSG00000136478	TEX2	Exon	1
EWS	3	1.86E+08	1.86E+08	1123	20	96.88	9.2	71.6	ENSG00000136527	TRA2B	Down	16
EWS	1	2.20E+08	2.20E+08	955	24	102.02	7.2	78.6	ENSG00000136628	EPRS	Down	7
EWS	8	1.29E+08	1.29E+08	1385	35	151.69	8.7	100	ENSG00000136997	MYC	Down	6
EWS	9	1.94E+07	1.94E+07	1298	35	116.41	6.3	89.6	ENSG00000137145	DENND4C	Exon	10
EWS	9	1.94E+07	1.94E+07	1298	35	116.41	6.3	89.6	ENSG00000137154	RPS6	Down	5
EWS	1	7.63E+07	7.63E+07	2217	73	285.41	8.1	100	ENSG00000137955	RABGGTB	Down	16
EWS	16	7.74E+07	7.74E+07	849	22	112.6	12.0	88.7	ENSG00000140873	ADAMTS18	Intron	3
EWS	16	2.01E+06	2.01E+06	1114	22	88.6	8.0	64.4	ENSG00000140986	RPL3L	Up	1
EWS	16	2.01E+06	2.01E+06	1114	22	88.6	8.0	64.4	ENSG00000140988	RPS2	Down	17
EWS	16	2.01E+06	2.01E+06	1114	22	88.6	8.0	64.4	ENSG00000140990	NDUFB10	Exon	2
EWS	17	7.98E+07	7.98E+07	838	15	91.94	14.1	66.7	ENSG00000141522	ARHGDIA	Down	3
EWS	19	5.13E+07	5.13E+07	1774	45	266.73	20.0	100	ENSG00000142513	ACPT	Intron	2
EWS	19	5.00E+07	5.00E+07	1490	35	115.84	8.6	86	ENSG00000142534	RPS11	Up	1
EWS	19	5.00E+07	5.00E+07	1490	35	115.84	8.6	86	ENSG00000142541	RPL13A	Down	13
EWS	1	6.79E+07	6.79E+07	1193	28	83.2	6.4	59	ENSG00000142864	SERBP1	Exon	8
EWS	1	4.53E+07	4.53E+07	2119	65	184.45	6.5	100	ENSG00000142959	BEST4	Up	1
EWS	1	2.28E+08	2.28E+08	1688	32	141.7	7.6	100	ENSG00000143740	SNAP47	Up	11
EWS	1	2.26E+08	2.26E+08	1743	42	120.75	7.5	100	ENSG00000143799	PARP1	Intron	11
EWS	3	1.29E+07	1.29E+07	921	17	84	11.0	60.3	ENSG00000144712	CAND2	Intron	5
EWS	3	1.29E+07	1.29E+07	921	17	84	11.0	60.3	ENSG00000144713	RPL32	Exon	8
EWS	4	2.03E+07	2.03E+07	960	24	137.3	11.4	100	ENSG00000145147	SLIT2	Intron	12
EWS	4	8.33E+07	8.33E+07	1303	30	142.71	9.0	100	ENSG00000145293	ENOPH1	Up	4
EWS	5	1.81E+08	1.81E+08	2070	55	203.32	8.6	100	ENSG00000146063	TRIM41	Down	11
EWS	6	1.37E+08	1.37E+08	1242	30	107.98	7.1	82.8	ENSG00000146410	FAM54A	Up	7
EWS	6	1.37E+08	1.37E+08	1227	31	86.63	7.2	65.1	ENSG00000146410	FAM54A	Up	7
EWS	X	9.64E+07	9.64E+07	1347	23	100.76	9.5	76.4	ENSG00000147202	DIAPH2	Intron	6
EWS	4	8.33E+07	8.33E+07	1303	30	142.71	9.0	100	ENSG00000152795	HNRPDL	Down	5
EWS	1	2.45E+08	2.45E+08	3824	114	158.54	5.7	100	ENSG00000153187	HNRNPU	Down	9
EWS	1	2.45E+08	2.45E+08	2487	56	109.04	5.7	86.4	ENSG00000153187	HNRNPU	Down	9
EWS	4	7.34E+07	7.34E+07	1576	32	92.05	7.1	67.4	ENSG00000156140	ADAMTS3	Intron	3
EWS	6	7.42E+07	7.42E+07	1322	34	108.22	8.8	84.1	ENSG00000156508	EEF1A1	Down	12
EWS	3	1.87E+08	1.87E+08	980	27	180.95	17.6	100	ENSG00000156976	EIF4A2	Down	28
EWS	21	4.45E+07	4.45E+07	704	20	97.72	10.0	75.3	ENSG00000160200	CBS	Down	20
EWS	22	2.20E+07	2.20E+07	831	21	110.05	11.4	87.7	ENSG00000161179	YDJC	Down	7
EWS	22	2.20E+07	2.20E+07	831	21	110.05	11.4	87.7	ENSG00000161180	CCDC116	Up	2
EWS	17	7.47E+07	7.47E+07	1001	19	100.74	9.5	75.3	ENSG00000161547	SRSF2	Down	5
EWS	17	7.47E+07	7.47E+07	1140	21	80.16	7.0	56.1	ENSG00000161547	SRSF2	Down	5
EWS	1	2.26E+08	2.26E+08	1743	42	120.75	7.5	100	ENSG00000163041	H3F3A	Down	4
EWS	3	1.87E+08	1.87E+08	980	27	180.95	17.6	100	ENSG00000163918	RFC4	Intron	14
EWS	9	8.66E+07	8.66E+07	930	22	120.23	13.1	100	ENSG00000165118	C9orf64	Up	3
EWS	9	8.66E+07	8.66E+07	930	22	120.23	13.1	100	ENSG00000165119	HNRNPK	Down	15
EWS	15	7.96E+07	7.96E+07	1011	23	107.7	12.5	83.1	ENSG00000166557	TMED3	Intron	4
EWS	18	3.26E+07	3.26E+07	1415	36	128.8	7.1	100	ENSG00000166974	MAPRE2	Intron	4
EWS	19	5.70E+06	5.70E+06	1226	20	127.85	11.9	100	ENSG00000167733	HSD11B1L	Down	6
EWS	19	5.13E+07	5.13E+07	1774	45	266.73	20.0	100	ENSG00000167747	C19orf48	Down	2
EWS	3	3.95E+07	3.95E+07	1066	19	85.8	7.1	63.9	ENSG00000168028	RPSA	Down	10
EWS	2	8.58E+07	8.58E+07	1050	18	87.16	7.9	64.4	ENSG00000168906	MAT2A	Down	7
EWS	2	8.58E+07	8.58E+07	1036	21	87.99	8.0	63.7	ENSG00000168906	MAT2A	Down	7
EWS	2	1.78E+08	1.78E+08	3253	92	154.27	7.3	100	ENSG00000170144	HNRNPA3	Down	8
EWS	19	5.47E+07	5.47E+07	1220	22	100.1	7.5	74.3	ENSG00000170889	RPS9	Intron	12
EWS	15	6.60E+07	6.60E+07	801	19	94.51	11.0	66.7	ENSG00000174485	DENND4A	Intron	3
EWS	3	2.40E+07	2.40E+07	1425	29	111.34	9.4	87.3	ENSG00000174748	RPL15	Intron	14
EWS	17	8.08E+06	8.08E+06	811	15	100.02	13.6	73.3	ENSG00000179029	TMEM107	Down	10
EWS	16	2.01E+06	2.01E+06	1114	22	88.6	8.0	64.4	ENSG00000179580	RNF151	Up	1
EWS	1	2.88E+07	2.88E+07	2270	110	495.44	17.0	100	ENSG00000180198	RCC1	Intron	12

EWS	1	2.88E+07	2.88E+07	1371	38	132.6	8.7	100	ENSG00000180198	RCC1	Intron	12
EWS	17	7.47E+07	7.47E+07	1140	21	80.16	7.0	56.1	ENSG00000181038	C17orf95	Intron	2
EWS	17	7.47E+07	7.47E+07	1001	19	100.74	9.5	75.3	ENSG00000181038	C17orf95	Intron	2
EWS	5	1.71E+08	1.71E+08	1372	24	115.83	9.2	84.3	ENSG00000181163	NPM1	Intron	12
EWS	22	3.93E+07	3.93E+07	1211	25	90.05	7.5	67	ENSG00000183741	CBX6	Down	3
EWS	8	3.76E+07	3.76E+07	1734	36	84.4	6.2	62	ENSG00000183779	ZNF703	Down	2
EWS	1	1.74E+08	1.74E+08	1261	34	156.97	10.3	100	ENSG00000185278	ZBTB37	Up	7
EWS	10	5.29E+07	5.29E+07	1005	25	93.16	7.9	70.5	ENSG00000185532	PRKG1	Intron	6
EWS	17	7.98E+07	7.98E+07	838	15	91.94	14.1	66.7	ENSG00000185624	P4HB	Up	16
EWS	22	2.20E+07	2.20E+07	831	21	110.05	11.4	87.7	ENSG00000185651	UBE2L3	Down	4
EWS	12	7.64E+07	7.64E+07	1182	25	108.43	9.8	83.9	ENSG00000187109	NAP1L1	Exon	7
EWS	2	2.33E+08	2.33E+08	2182	63	250.74	10.7	100	ENSG00000187514	PTMA	Down	13
EWS	2	2.35E+08	2.35E+08	588	13	82.49	10.0	58.9	ENSG00000188042	ARL4C	Down	2
EWS	1	2.45E+08	2.45E+08	2487	56	109.04	5.7	86.4	ENSG00000188206	NCRNA00201	Exon	3
EWS	1	2.45E+08	2.45E+08	3824	114	158.54	5.7	100	ENSG00000188206	NCRNA00201	Intron	3
EWS	1	9.30E+05	9.31E+05	888	17	109.74	16.7	86.2	ENSG00000188290	HES4	Down	4
EWS	19	5.70E+06	5.70E+06	1226	20	127.85	11.9	100	ENSG00000196365	LONP1	Intron	3
EWS	3	2.40E+07	2.40E+07	1425	29	111.34	9.4	87.3	ENSG00000197885	NKIRAS1	Intron	8
EWS	5	1.77E+08	1.77E+08	1116	32	209.84	20.0	100	ENSG00000198055	GRK6	Down	11
EWS	13	9.20E+07	9.20E+07	5252	144	294.99	6.4	100	ENSG00000199149	MIR20A	Down	1
EWS	13	9.20E+07	9.20E+07	5252	144	294.99	6.4	100	ENSG00000199180	MIR18A	Down	1
EWS	17	5.67E+07	5.67E+07	810	17	116.76	13.3	93.5	ENSG00000199426	U1.5	Down	1
EWS	19	5.00E+07	5.00E+07	1490	35	115.84	8.6	86	ENSG00000199631	SNORD33	Down	1
EWS	17	6.22E+07	6.22E+07	1487	38	162.5	9.1	100	ENSG00000199753	SNORD104	Down	1
EWS	1	1.74E+08	1.74E+08	1261	34	156.97	10.3	100	ENSG00000200016	SNORD76	Down	1
EWS	1	2.88E+07	2.88E+07	2270	110	495.44	17.0	100	ENSG00000200087	SNORA73B	Down	1
EWS	1	2.88E+07	2.88E+07	1371	38	132.6	8.7	100	ENSG00000200087	SNORA73B	Down	1
EWS	19	5.00E+07	5.00E+07	1490	35	115.84	8.6	86	ENSG00000200259	SNORD35A	Down	1
EWS	3	1.87E+08	1.87E+08	980	27	180.95	17.6	100	ENSG00000200320	SNORA63	Down	1
EWS	3	1.87E+08	1.87E+08	980	27	180.95	17.6	100	ENSG00000200418	SNORA63.6	Down	1
EWS	17	8.08E+06	8.08E+06	811	15	100.02	13.6	73.3	ENSG00000200463	U8.4	Down	1
EWS	19	5.00E+07	5.00E+07	1490	35	115.84	8.6	86	ENSG00000200530	SNORD35B	Up	1
EWS	1	1.74E+08	1.74E+08	1261	34	156.97	10.3	100	ENSG00000200710	SNORD81	Down	1
EWS	1	1.74E+08	1.74E+08	1261	34	156.97	10.3	100	ENSG00000200729	SNORD79	Down	1
EWS	1	1.74E+08	1.74E+08	1261	34	156.97	10.3	100	ENSG00000200954	SNORD74	Down	1
EWS	17	5.68E+07	5.68E+07	1103	35	156.68	13.6	100	ENSG00000200997	U1.36	Down	1
EWS	1	7.63E+07	7.63E+07	2217	73	285.41	8.1	100	ENSG00000201487	SNORD45B	Down	1
EWS	19	5.00E+07	5.00E+07	1490	35	115.84	8.6	86	ENSG00000201675	SNORD32A	Down	1
EWS	1	1.74E+08	1.74E+08	1261	34	156.97	10.3	100	ENSG00000201692	SNORD80	Down	1
EWS	1	2.88E+07	2.88E+07	2270	110	495.44	17.0	100	ENSG00000201808	SNORA73A	Down	1
EWS	1	2.88E+07	2.88E+07	1371	38	132.6	8.7	100	ENSG00000201808	SNORA73A	Down	1
EWS	6	8.64E+07	8.64E+07	680	15	90.99	11.3	66.3	ENSG00000201865	SNORD50B	Down	1
EWS	17	5.67E+07	5.67E+07	810	17	116.76	13.3	93.5	ENSG00000202077	U1.57	Down	1
EWS	3	3.95E+07	3.95E+07	1066	19	85.8	7.1	63.9	ENSG00000202363	SNORA62.4	Down	1
EWS	1	1.74E+08	1.74E+08	1261	34	156.97	10.3	100	ENSG00000202394	SNORD47	Down	1
EWS	19	5.00E+07	5.00E+07	1490	35	115.84	8.6	86	ENSG00000202503	SNORD34	Down	1
EWS	1	2.45E+08	2.45E+08	3824	114	158.54	5.7	100	ENSG00000203667	FAM36A	Down	5
EWS	1	2.45E+08	2.45E+08	2487	56	109.04	5.7	86.4	ENSG00000203667	FAM36A	Intron	5
EWS	6	8.64E+07	8.64E+07	680	15	90.99	11.3	66.3	ENSG00000203875	SNHG5	Down	10
EWS	1	2.88E+07	2.88E+07	2270	110	495.44	17.0	100	ENSG00000204138	PHACTR4	Down	5
EWS	6	3.29E+07	3.30E+07	1473	42	211.43	12.9	100	ENSG00000204256	BRD2	Down	16
EWS	5	1.81E+08	1.81E+08	2070	55	203.32	8.6	100	ENSG00000204628	GNB2L1	Down	35
EWS	19	4.37E+07	4.37E+07	1604	95	508.57	13.0	100	ENSG00000204941	PSG5	Up	9
EWS	19	4.37E+07	4.37E+07	2371	126	519.09	13.8	100	ENSG00000204941	PSG5	Up	9
EWS	14	3.50E+07	3.50E+07	969	19	97.1	11.3	72.5	ENSG00000206588	RNU1-8	Down	1
EWS	14	3.50E+07	3.50E+07	969	19	97.1	11.3	72.5	ENSG00000206596	RNU1-7	Down	1
EWS	1	1.74E+08	1.74E+08	1261	34	156.97	10.3	100	ENSG00000206607	SNORD44	Down	1
EWS	1	7.63E+07	7.63E+07	2217	73	285.41	8.1	100	ENSG00000206620	SNORD45C	Down	1
EWS	3	3.95E+07	3.95E+07	1066	19	85.8	7.1	63.9	ENSG00000206760	SNORA6	Down	1
EWS	16	2.01E+06	2.01E+06	1114	22	88.6	8.0	64.4	ENSG00000206811	SNORA10	Down	1
EWS	17	5.68E+07	5.68E+07	1103	35	156.68	13.6	100	ENSG00000206917	U1.78	Up	1
EWS	6	8.64E+07	8.64E+07	680	15	90.99	11.3	66.3	ENSG00000207066	SNORD50A	Down	1
EWS	1	7.63E+07	7.63E+07	2217	73	285.41	8.1	100	ENSG00000207241	SNORD45A	Down	1
EWS	16	2.01E+06	2.01E+06	1114	22	88.6	8.0	64.4	ENSG00000207405	SNORA64	Down	1
EWS	3	1.29E+07	1.29E+07	921	17	84	11.0	60.3	ENSG00000207496	SNORA7A	Down	1
EWS	17	7.47E+07	7.47E+07	1001	19	100.74	9.5	75.3	ENSG00000207556	MIR636	Down	1
EWS	17	7.47E+07	7.47E+07	1140	21	80.16	7.0	56.1	ENSG00000207556	MIR636	Down	1
EWS	13	9.20E+07	9.20E+07	5252	144	294.99	6.4	100	ENSG00000207560	MIR19B1	Down	1

EWS	9	8.66E+07	8.66E+07	930	22	120.23	13.1	100	ENSG00000207603	MIR7-1	Down	1
EWS	13	9.20E+07	9.20E+07	5252	144	294.99	6.4	100	ENSG00000207610	MIR19A	Down	1
EWS	13	9.20E+07	9.20E+07	5252	144	294.99	6.4	100	ENSG00000207745	MIR17	Down	1
EWS	19	5.00E+07	5.00E+07	1490	35	115.84	8.6	86	ENSG00000207782	MIR150	Down	1
EWS	13	9.20E+07	9.20E+07	5252	144	294.99	6.4	100	ENSG00000207968	MIR92A1	Down	1
EWS	1	1.74E+08	1.74E+08	1261	34	156.97	10.3	100	ENSG00000208310	SNORD75	Down	1
EWS	1	1.74E+08	1.74E+08	1261	34	156.97	10.3	100	ENSG00000208313	SNORD77	Down	1
EWS	1	1.74E+08	1.74E+08	1261	34	156.97	10.3	100	ENSG00000208317	SNORD78	Down	1
EWS	5	1.81E+08	1.81E+08	2070	55	203.32	8.6	100	ENSG00000208342	SNORD96A	Down	1
EWS	X	4.12E+07	4.12E+07	1473	44	123.23	6.5	100	ENSG00000215301	DDX3X	Intron	7
EWS	13	9.20E+07	9.20E+07	5252	144	294.99	6.4	100	ENSG00000215417	MIR17HG	Down	1
EWS	17	8.08E+06	8.08E+06	811	15	100.02	13.6	73.3	ENSG00000220205	VAMP2	Up	5
EWS	19	5.13E+07	5.13E+07	1774	45	266.73	20.0	100	ENSG00000220988	SNORD88C	Down	1
EWS	19	5.13E+07	5.13E+07	1774	45	266.73	20.0	100	ENSG00000221233	AC010325.2	Down	1
EWS	19	5.13E+07	5.13E+07	1774	45	266.73	20.0	100	ENSG00000221241	SNORD88A	Down	1
EWS	19	5.13E+07	5.13E+07	1774	45	266.73	20.0	100	ENSG00000221381	SNORD88B	Down	1
EWS	3	1.87E+08	1.87E+08	980	27	180.95	17.6	100	ENSG00000221420	SNORA81	Down	1
EWS	17	6.22E+07	6.22E+07	1487	38	162.5	9.1	100	ENSG00000221462	SNORA76	Down	1
EWS	1	6.79E+07	6.79E+07	1193	28	83.2	6.4	59	ENSG00000223263	U6.930	Up	1
EWS	19	5.47E+07	5.47E+07	1220	22	100.1	7.5	74.3	ENSG00000223660	AC012314.20	Up	1
EWS	19	5.47E+07	5.47E+07	1220	22	100.1	7.5	74.3	ENSG00000224579	AC012314.19	Up	1
EWS	3	8.63E+06	8.63E+06	1479	47	209.56	11.4	100	ENSG00000224884	AC034187.2	Intron	1
EWS	2	1.78E+08	1.78E+08	3253	92	154.27	7.3	100	ENSG00000225808	AC079305.9	Down	1
EWS	3	8.63E+06	8.63E+06	1479	47	209.56	11.4	100	ENSG00000227110	AC087859.1	Intron	7
EWS	2	1.78E+08	1.78E+08	3253	92	154.27	7.3	100	ENSG00000229337	AC079305.8	Up	1
EWS	6	7.42E+07	7.42E+07	1322	34	108.22	8.8	84.1	ENSG00000229862	RP11-505P4.7	Up	2
EWS	1	1.74E+08	1.74E+08	1261	34	156.97	10.3	100	ENSG00000231792	RP5-1198E17.1	Up	1
EWS	5	1.81E+08	1.81E+08	2070	55	203.32	8.6	100	ENSG00000233937	CTC-338M12.4	Up	4
EWS	1	1.74E+08	1.74E+08	1261	34	156.97	10.3	100	ENSG00000234741	GAS5	Down	29
EWS	9	1.94E+07	1.94E+07	1298	35	116.41	6.3	89.6	ENSG00000234853	RP11-513M16.5	Down	1
EWS	9	8.66E+07	8.66E+07	930	22	120.23	13.1	100	ENSG00000235298	RP11-575L7.8	Up	1
EWS	10	1.02E+08	1.02E+08	719	18	95.23	10.0	69.9	ENSG00000235823	NCRNA00263	Up	1
EWS	16	2.01E+06	2.01E+06	1114	22	88.6	8.0	64.4	ENSG00000238671	SNORA78	Up	1
EWS	1	2.88E+07	2.88E+07	2270	110	495.44	17.0	100	ENSG00000238821	snoU13.263	Up	1
EWS	1	2.88E+07	2.88E+07	1371	38	132.6	8.7	100	ENSG00000238821	snoU13.263	Up	1
EWS	3	1.87E+08	1.87E+08	980	27	180.95	17.6	100	ENSG00000238942	snR39B.2	Down	1
EWS	13	9.20E+07	9.20E+07	5252	144	294.99	6.4	100	ENSG00000239594	RP11-282D2.3	Down	1
EWS	13	9.20E+07	9.20E+07	5252	144	294.99	6.4	100	ENSG00000240532	RP11-282D2.5	Down	1
EWS	22	2.01E+07	2.01E+07	933	17	106	11.7	81.8	ENSG00000240816	AC006547.1	Up	1
EWS	13	9.20E+07	9.20E+07	5252	144	294.99	6.4	100	ENSG00000242008	RP11-282D2.2	Down	1
EWS	1	2.88E+07	2.88E+07	1371	38	132.6	8.7	100	ENSG00000242125	SNHG3	Down	3
EWS	1	2.88E+07	2.88E+07	2270	110	495.44	17.0	100	ENSG00000242125	SNHG3	Exon	3
EWS	13	9.20E+07	9.20E+07	5252	144	294.99	6.4	100	ENSG00000243108	RP11-282D2.6	Down	1
EWS	19	4.37E+07	4.37E+07	2371	126	519.09	13.8	100	ENSG00000243137	PSG4	Down	6
EWS	19	4.37E+07	4.37E+07	1604	95	508.57	13.0	100	ENSG00000243137	PSG4	Down	6
EWS	13	9.20E+07	9.20E+07	5252	144	294.99	6.4	100	ENSG00000243839	RP11-282D2.4	Down	1
EWS	20	3.43E+07	3.43E+07	613	14	83.93	11.8	60.7	ENSG00000244005	NFS1	Up	19
EWS	1	2.88E+07	2.88E+07	2270	110	495.44	17.0	100	ENSG00000245178	AL513497.1	Up	1
EWS	1	2.88E+07	2.88E+07	1371	38	132.6	8.7	100	ENSG00000245178	AL513497.1	Up	1
EWS	11	6.52E+07	6.52E+07	1364	25	96.18	7.0	70.7	ENSG00000245532	NEAT1	Exon	2
EWS	15	6.08E+07	6.08E+07	715	17	128.12	20.4	100	ENSG00000245534	AC087385.1	Intron	1
EWS	11	8.28E+07	8.28E+07	860	15	80.61	9.5	57	ENSG00000246067	RP11-113K21.5	Intron	8
EWS	5	1.77E+08	1.77E+08	1116	32	209.84	20.0	100	ENSG00000246334	RP11-1334A24.4	Intron	4
EWS	5	1.81E+08	1.81E+08	2070	55	203.32	8.6	100	ENSG00000247049	CTC-338M12.7	Up	1
EWS	4	1.49E+07	1.49E+07	1391	30	80.64	6.9	57.5	ENSG00000247624	AC006296.3	Intron	2
EWS	5	1.77E+08	1.77E+08	1116	32	209.84	20.0	100	ENSG00000248342	AC145098.1	Down	1
EWS	8	1.29E+08	1.29E+08	1385	35	151.69	8.7	100	ENSG00000249375	RP11-1136L8.1	Up	3
EWS	16	2.01E+06	2.01E+06	1114	22	88.6	8.0	64.4	ENSG00000255066	AC005363.7	Down	1
EWS	16	2.01E+06	2.01E+06	1114	22	88.6	8.0	64.4	ENSG00000255198	SNHG9	Up	1
EWS	16	2.01E+06	2.01E+06	1114	22	88.6	8.0	64.4	ENSG00000255278	AC005363.8	Down	1
EWS	11	8.28E+07	8.28E+07	860	15	80.61	9.5	57	ENSG00000255503	RP11-113K21.4	Intron	1
EWS	16	2.01E+06	2.01E+06	1114	22	88.6	8.0	64.4	ENSG00000255513	AC005363.9	Down	1
EWS	9	3.78E+07	3.78E+07	728	15	91.64	13.5	66	ENSG00000255872	RP11-613M10.9	Intron	1
EWS	8	1.29E+08	1.29E+08	1385	35	151.69	8.7	100	ENSG00000256129	AC103819.1	Down	1
EWS	10	1.02E+08	1.02E+08	719	18	95.23	10.0	69.9	ENSG00000256545	AL139819.1	Down	1
EWS	1	2.88E+07	2.88E+07	1371	38	132.6	8.7	100	ENSG00000256960	AL513497.2	Down	1
EWS	1	2.88E+07	2.88E+07	2270	110	495.44	17.0	100	ENSG00000256960	AL513497.2	Down	1
Ac-H3K9	3	1.70E+08	1.70E+08	1796	80	419.98	22.0	0.4	ENSG00000008952	SEC62	Up	12

Ac-H3K9	6	1.37E+08	1.37E+08	1111	47	209.04	17.7	0.53	ENSG00000029363	BCLAF1	Down	23
Ac-H3K9	6	1.37E+08	1.37E+08	1171	46	194.74	10.2	0.54	ENSG00000029363	BCLAF1	Down	23
Ac-H3K9	6	1.37E+08	1.37E+08	2514	140	642.89	35.1	0.53	ENSG00000029363	BCLAF1	Intron	23
Ac-H3K9	6	1.37E+08	1.37E+08	1420	101	725.14	41.6	0.49	ENSG00000029363	BCLAF1	Up	23
Ac-H3K9	7	2.62E+07	2.62E+07	4637	359	1772	25.1	2.6	ENSG00000050344	NFE2L3	Down	2
Ac-H3K9	1	7.62E+07	7.63E+07	6074	709	3100	25.9	16.7	ENSG00000057468	MSH4	Up	1
Ac-H3K9	15	6.08E+07	6.08E+07	1425	64	442.21	30.7	0.43	ENSG00000069667	RORA	Down	4
Ac-H3K9	15	6.08E+07	6.08E+07	600	16	97.48	15.3	0.78	ENSG00000069667	RORA	Down	4
Ac-H3K9	17	7.47E+07	7.47E+07	1318	56	407.28	42.1	0.37	ENSG00000070495	JMJD6	Down	5
Ac-H3K9	17	7.47E+07	7.47E+07	3446	188	1255.8	33.1	0.84	ENSG00000070495	JMJD6	Up	5
Ac-H3K9	17	7.47E+07	7.47E+07	1098	32	109.38	13.2	0.75	ENSG00000070495	JMJD6	Up	5
Ac-H3K9	2	1.02E+08	1.02E+08	2273	152	1290.1	44.5	0.92	ENSG00000071082	RPL31	Exon	11
Ac-H3K9	1	2.28E+08	2.28E+08	1064	54	283.14	17.5	0.55	ENSG00000081692	JMJD4	Exon	5
Ac-H3K9	9	3.30E+07	3.30E+07	2548	144	1124.2	50.6	0.91	ENSG00000086061	DNAJA1	Up	5
Ac-H3K9	19	5.00E+07	5.00E+07	2346	298	2807.1	63.7	12.5	ENSG00000090554	FLT3LG	Down	2
Ac-H3K9	19	5.00E+07	5.00E+07	1731	58	285.35	18.4	0.54	ENSG00000090554	FLT3LG	Down	2
Ac-H3K9	17	7.47E+07	7.47E+07	801	20	87.16	8.6	0.78	ENSG00000092931	MFSD11	Exon	2
Ac-H3K9	17	7.47E+07	7.47E+07	1185	42	289.73	27.9	0.55	ENSG00000092931	MFSD11	Intron	2
Ac-H3K9	17	7.47E+07	7.47E+07	1098	32	109.38	13.2	0.75	ENSG00000092931	MFSD11	Up	2
Ac-H3K9	17	7.47E+07	7.47E+07	3446	188	1255.8	33.1	0.84	ENSG00000092931	MFSD11	Up	2
Ac-H3K9	12	5.47E+07	5.47E+07	1966	164	1148.2	23.3	0.98	ENSG00000094916	CBX5	Intron	2
Ac-H3K9	12	5.47E+07	5.47E+07	4376	307	1777	37.6	2.63	ENSG00000094916	CBX5	Up	2
Ac-H3K9	10	1.02E+08	1.02E+08	836	24	100.67	10.6	0.76	ENSG00000099194	SCD	Down	2
Ac-H3K9	10	1.02E+08	1.02E+08	1282	116	934.3	57.3	0.54	ENSG00000099194	SCD	Down	2
Ac-H3K9	10	1.02E+08	1.02E+08	1916	143	1253.2	35.1	0.83	ENSG00000099194	SCD	Exon	2
Ac-H3K9	10	1.02E+08	1.02E+08	1061	50	194.41	16.9	0.54	ENSG00000099194	SCD	Up	2
Ac-H3K9	22	2.01E+07	2.01E+07	1791	116	545.13	20.3	0.51	ENSG00000099901	RANBP1	Intron	15
Ac-H3K9	22	2.01E+07	2.01E+07	1170	57	414.94	32.2	0.39	ENSG00000099901	RANBP1	Intron	15
Ac-H3K9	22	2.01E+07	2.01E+07	1791	116	545.13	20.3	0.51	ENSG00000099904	ZDHHC8	Up	7
Ac-H3K9	22	3.89E+07	3.89E+07	3921	252	1579.4	36.8	1.87	ENSG00000100201	DDX17	Intron	14
Ac-H3K9	16	7.43E+07	7.43E+07	968	28	163.79	14.9	0.64	ENSG00000103035	PSMD7	Intron	2
Ac-H3K9	16	7.43E+07	7.43E+07	917	29	108.88	11.4	0.74	ENSG00000103035	PSMD7	Up	2
Ac-H3K9	9	3.78E+07	3.78E+07	2278	78	501.43	29.8	0.49	ENSG00000107371	EXOSC3	Exon	8
Ac-H3K9	9	3.78E+07	3.78E+07	1012	41	300.66	34.3	0.48	ENSG00000107371	EXOSC3	Intron	8
Ac-H3K9	9	3.78E+07	3.78E+07	2705	124	478.86	13.0	0.45	ENSG00000107371	EXOSC3	Up	8
Ac-H3K9	2	8.58E+07	8.58E+07	4623	297	1956.4	41.9	3.28	ENSG00000115486	GGCX	Down	10
Ac-H3K9	2	8.58E+07	8.58E+07	773	46	382.63	36.4	0.41	ENSG00000115486	GGCX	Up	10
Ac-H3K9	2	8.82E+06	8.83E+06	622	21	133.73	16.9	0.68	ENSG00000115738	ID2	Down	4
Ac-H3K9	2	8.82E+06	8.82E+06	2430	136	1090.3	34.8	0.84	ENSG00000115738	ID2	Exon	4
Ac-H3K9	2	8.82E+06	8.82E+06	1081	41	174.36	13.2	0.55	ENSG00000115738	ID2	Intron	4
Ac-H3K9	2	8.82E+06	8.82E+06	1016	30	167.44	27.6	0.61	ENSG00000115738	ID2	Intron	4
Ac-H3K9	2	8.82E+06	8.82E+06	533	21	152.57	26.2	0.65	ENSG00000115738	ID2	Up	4
Ac-H3K9	2	1.78E+08	1.78E+08	1125	50	358.32	24.2	0.43	ENSG00000116044	NFE2L2	Exon	12
Ac-H3K9	2	1.78E+08	1.78E+08	1117	28	148.06	13.3	0.64	ENSG00000116044	NFE2L2	Intron	12
Ac-H3K9	2	1.78E+08	1.78E+08	1150	45	138.22	9.8	0.67	ENSG00000116044	NFE2L2	Intron	12
Ac-H3K9	2	1.78E+08	1.78E+08	1529	45	213.91	13.1	0.52	ENSG00000116044	NFE2L2	Up	12
Ac-H3K9	1	6.82E+07	6.82E+07	2291	85	366.65	14.2	0.44	ENSG00000116717	GADD45A	Intron	4
Ac-H3K9	1	7.62E+07	7.62E+07	3295	202	1169.9	26.3	1.04	ENSG00000117054	ACADM	Intron	19
Ac-H3K9	1	7.62E+07	7.63E+07	6074	709	3100	25.9	16.7	ENSG00000117054	ACADM	Intron	19
Ac-H3K9	1	1.74E+08	1.74E+08	1513	38	108.04	7.0	0.76	ENSG00000117593	DARS2	Down	4
Ac-H3K9	1	1.74E+08	1.74E+08	2862	311	2345.4	29.1	6.25	ENSG00000117593	DARS2	Down	4
Ac-H3K9	1	1.74E+08	1.74E+08	2506	184	1567.4	48.5	1.8	ENSG00000117593	DARS2	Exon	4
Ac-H3K9	13	7.47E+07	7.47E+07	2837	157	705.51	20.5	0.56	ENSG00000118922	KLF12	Intron	4
Ac-H3K9	13	7.47E+07	7.47E+07	520	28	139.54	14.3	0.67	ENSG00000118922	KLF12	Up	4
Ac-H3K9	13	7.47E+07	7.47E+07	1075	54	244.53	14.7	0.49	ENSG00000118922	KLF12	Up	4
Ac-H3K9	13	7.47E+07	7.47E+07	519	21	86.04	10.7	0.79	ENSG00000118922	KLF12	Up	4
Ac-H3K9	17	5.68E+07	5.68E+07	504	29	159.49	19.2	0.62	ENSG00000121101	TEX14	Intron	3
Ac-H3K9	17	5.67E+07	5.67E+07	924	37	202.7	20.4	0.53	ENSG00000121101	TEX14	Intron	3
Ac-H3K9	17	5.67E+07	5.67E+07	1315	81	442.8	31.4	0.43	ENSG00000121101	TEX14	Intron	3
Ac-H3K9	17	5.68E+07	5.68E+07	1905	101	672.68	33.1	0.59	ENSG00000121101	TEX14	Up	3
Ac-H3K9	16	1.18E+07	1.18E+07	486	20	126.15	17.2	0.68	ENSG00000122299	ZC3H7A	Down	2
Ac-H3K9	16	1.18E+07	1.18E+07	1409	42	261.45	22.4	0.51	ENSG00000122299	ZC3H7A	Down	2
Ac-H3K9	16	1.19E+07	1.19E+07	519	17	123.42	29.9	0.7	ENSG00000122299	ZC3H7A	Intron	2
Ac-H3K9	16	1.19E+07	1.19E+07	725	27	134.25	12.3	0.69	ENSG00000122299	ZC3H7A	Up	2
Ac-H3K9	7	2.62E+07	2.62E+07	4637	359	1772	25.1	2.6	ENSG00000122566	HNRNPA2B1	Exon	8
Ac-H3K9	7	2.62E+07	2.62E+07	2894	185	1325.6	40.1	1	ENSG00000122566	HNRNPA2B1	Up	8
Ac-H3K9	9	3.31E+07	3.31E+07	1348	79	703.5	46.7	0.56	ENSG00000122692	SMU1	Intron	2
Ac-H3K9	9	3.78E+07	3.78E+07	1012	41	300.66	34.3	0.48	ENSG00000122741	DCAF10	Exon	5

Ac-H3K9	9	3.78E+07	3.78E+07	2705	124	478.86	13.0	0.45	ENSG00000122741	DCAF10	Intron	5
Ac-H3K9	12	5.47E+07	5.47E+07	4376	307	1777	37.6	2.63	ENSG00000123405	NFE2	Down	3
Ac-H3K9	20	3.43E+07	3.43E+07	1155	53	374.41	23.0	0.39	ENSG00000125995	ROMO1	Exon	5
Ac-H3K9	20	3.43E+07	3.43E+07	668	19	113.42	14.5	0.75	ENSG00000125995	ROMO1	Up	5
Ac-H3K9	7	1.30E+08	1.30E+08	1566	47	290.51	22.3	0.53	ENSG00000128607	KLHDC10	Intron	4
Ac-H3K9	7	1.30E+08	1.30E+08	1081	52	214.03	13.0	0.52	ENSG00000128607	KLHDC10	Up	4
Ac-H3K9	17	7.49E+06	7.49E+06	1797	93	545.39	37.8	0.51	ENSG00000129194	SOX15	Down	2
Ac-H3K9	17	7.49E+06	7.49E+06	1797	93	545.39	37.8	0.51	ENSG00000129226	CD68	Down	2
Ac-H3K9	17	7.48E+06	7.48E+06	1997	95	468.76	16.7	0.43	ENSG00000129226	CD68	Up	2
Ac-H3K9	17	7.49E+06	7.49E+06	1797	93	545.39	37.8	0.51	ENSG00000129255	MPDU1	Intron	5
Ac-H3K9	17	7.48E+06	7.48E+06	1997	95	468.76	16.7	0.43	ENSG00000129255	MPDU1	Up	5
Ac-H3K9	14	3.50E+07	3.50E+07	632	21	149.4	31.8	0.64	ENSG00000129515	SNX6	Down	4
Ac-H3K9	14	3.51E+07	3.51E+07	1023	28	143.85	14.6	0.66	ENSG00000129515	SNX6	Intron	4
Ac-H3K9	19	5.69E+06	5.69E+06	1493	119	1177.2	54.1	1.06	ENSG00000130255	RPL36	Exon	2
Ac-H3K9	19	5.68E+06	5.68E+06	829	27	184.52	20.6	0.54	ENSG00000130255	RPL36	Up	2
Ac-H3K9	19	1.02E+07	1.02E+07	1102	54	439.39	31.8	0.43	ENSG00000130810	PPAN	Down	10
Ac-H3K9	19	1.02E+07	1.02E+07	1460	81	451.94	22.8	0.45	ENSG00000130810	PPAN	Exon	10
Ac-H3K9	19	1.02E+07	1.02E+07	1460	81	451.94	22.8	0.45	ENSG00000130811	EIF3G	Down	1
Ac-H3K9	19	1.02E+07	1.02E+07	1102	54	439.39	31.8	0.43	ENSG00000130811	EIF3G	Intron	1
Ac-H3K9	20	3.43E+07	3.43E+07	1155	53	374.41	23.0	0.39	ENSG00000131051	RBM39	Down	45
Ac-H3K9	20	3.43E+07	3.43E+07	668	19	113.42	14.5	0.75	ENSG00000131051	RBM39	Down	45
Ac-H3K9	20	3.43E+07	3.43E+07	2038	119	842.14	45.0	0.42	ENSG00000131051	RBM39	Intron	45
Ac-H3K9	20	3.43E+07	3.43E+07	1371	126	1195	85.6	1.12	ENSG00000131051	RBM39	Up	45
Ac-H3K9	13	4.59E+07	4.59E+07	1978	114	955.13	50.3	0.58	ENSG00000133112	TPT1	Exon	13
Ac-H3K9	13	4.59E+07	4.59E+07	1159	36	204.49	18.4	0.53	ENSG00000133112	TPT1	Up	13
Ac-H3K9	6	7.42E+07	7.42E+07	2409	230	2086	46.3	3.92	ENSG00000135297	MTO1	Down	20
Ac-H3K9	6	7.42E+07	7.42E+07	1095	66	534.57	35.3	0.49	ENSG00000135297	MTO1	Exon	20
Ac-H3K9	11	3.63E+07	3.63E+07	754	23	153.79	20.6	0.64	ENSG00000135362	PRR5L	Up	21
Ac-H3K9	12	5.47E+07	5.47E+07	4376	307	1777	37.6	2.63	ENSG00000135486	HNRNPA1	Intron	3
Ac-H3K9	12	5.47E+07	5.47E+07	1966	164	1148.2	23.3	0.98	ENSG00000135486	HNRNPA1	Up	3
Ac-H3K9	13	7.61E+07	7.61E+07	2964	129	465.94	14.3	0.43	ENSG00000136111	TBC1D4	Intron	8
Ac-H3K9	13	7.61E+07	7.61E+07	895	41	103.27	12.3	0.76	ENSG00000136111	TBC1D4	Up	8
Ac-H3K9	17	6.22E+07	6.22E+07	1648	106	627.31	21.9	0.51	ENSG00000136478	TEX2	Down	1
Ac-H3K9	17	6.22E+07	6.22E+07	919	25	107.58	11.6	0.75	ENSG00000136478	TEX2	Down	1
Ac-H3K9	17	6.23E+07	6.23E+07	792	21	113.45	13.8	0.75	ENSG00000136478	TEX2	Intron	1
Ac-H3K9	17	6.23E+07	6.23E+07	598	16	90.9	18.4	0.77	ENSG00000136478	TEX2	Up	1
Ac-H3K9	3	1.86E+08	1.86E+08	5056	318	2035.6	34.0	3.57	ENSG00000136527	TRA2B	Exon	16
Ac-H3K9	1	2.20E+08	2.20E+08	1006	51	298.77	19.4	0.48	ENSG00000136628	EPRS	Intron	7
Ac-H3K9	8	1.29E+08	1.29E+08	665	17	106.01	18.7	0.76	ENSG00000136997	MYC	Exon	6
Ac-H3K9	8	1.29E+08	1.29E+08	847	28	183.67	22.5	0.54	ENSG00000136997	MYC	Intron	6
Ac-H3K9	8	1.29E+08	1.29E+08	735	17	85.81	16.9	0.8	ENSG00000136997	MYC	Intron	6
Ac-H3K9	8	1.29E+08	1.29E+08	631	24	177.97	25.3	0.55	ENSG00000136997	MYC	Up	6
Ac-H3K9	9	1.94E+07	1.94E+07	3850	351	2729	50.6	18.2	ENSG00000137145	DENND4C	Down	10
Ac-H3K9	9	1.94E+07	1.94E+07	3850	351	2729	50.6	18.2	ENSG00000137154	RPS6	Exon	5
Ac-H3K9	11	7.29E+07	7.29E+07	2458	88	301.76	15.3	0.48	ENSG00000137478	FCHSD2	Intron	10
Ac-H3K9	1	7.62E+07	7.63E+07	6074	709	3100	25.9	16.7	ENSG00000137955	RABGGTB	Up	16
Ac-H3K9	16	7.74E+07	7.74E+07	826	19	93.67	12.3	0.78	ENSG00000140873	ADAMTS18	Intron	3
Ac-H3K9	16	1.99E+06	1.99E+06	441	14	102.96	20.6	0.77	ENSG00000140986	RPL3L	Down	1
Ac-H3K9	16	1.99E+06	1.99E+06	609	17	112.13	24.3	0.75	ENSG00000140986	RPL3L	Down	1
Ac-H3K9	16	2.01E+06	2.01E+06	2609	200	982.64	18.4	0.62	ENSG00000140986	RPL3L	Up	1
Ac-H3K9	16	2.01E+06	2.01E+06	1124	32	92.25	8.4	0.77	ENSG00000140986	RPL3L	Up	1
Ac-H3K9	16	2.01E+06	2.01E+06	1486	70	416.63	25.5	0.39	ENSG00000140986	RPL3L	Up	1
Ac-H3K9	16	2.01E+06	2.01E+06	1124	32	92.25	8.4	0.77	ENSG00000140988	RPS2	Down	17
Ac-H3K9	16	2.01E+06	2.01E+06	1486	70	416.63	25.5	0.39	ENSG00000140988	RPS2	Down	17
Ac-H3K9	16	2.01E+06	2.01E+06	2609	200	982.64	18.4	0.62	ENSG00000140988	RPS2	Exon	17
Ac-H3K9	16	2.02E+06	2.02E+06	1430	51	349.89	44.9	0.44	ENSG00000140988	RPS2	Up	17
Ac-H3K9	16	2.01E+06	2.02E+06	1300	61	415.46	28.5	0.39	ENSG00000140988	RPS2	Up	17
Ac-H3K9	16	2.01E+06	2.01E+06	2609	200	982.64	18.4	0.62	ENSG00000140990	NDUFB10	Down	2
Ac-H3K9	16	2.01E+06	2.02E+06	1300	61	415.46	28.5	0.39	ENSG00000140990	NDUFB10	Down	2
Ac-H3K9	16	2.02E+06	2.02E+06	1430	51	349.89	44.9	0.44	ENSG00000140990	NDUFB10	Down	2
Ac-H3K9	16	2.01E+06	2.01E+06	1486	70	416.63	25.5	0.39	ENSG00000140990	NDUFB10	Intron	2
Ac-H3K9	16	2.01E+06	2.01E+06	1124	32	92.25	8.4	0.77	ENSG00000140990	NDUFB10	Intron	2
Ac-H3K9	17	7.98E+07	7.98E+07	956	49	402.69	36.1	0.37	ENSG00000141522	ARHGDIA	Down	3
Ac-H3K9	17	7.98E+07	7.98E+07	1000	41	239.09	27.0	0.48	ENSG00000141522	ARHGDIA	Down	3
Ac-H3K9	17	7.98E+07	7.98E+07	1491	59	426.33	31.8	0.4	ENSG00000141522	ARHGDIA	Intron	3
Ac-H3K9	17	7.98E+07	7.98E+07	1402	81	715.52	50.5	0.57	ENSG00000141522	ARHGDIA	Up	3
Ac-H3K9	19	5.13E+07	5.13E+07	2108	174	1759.9	70.2	2.47	ENSG00000142513	ACPT	Down	2
Ac-H3K9	19	5.00E+07	5.00E+07	1602	173	1514.7	59.3	1.53	ENSG00000142534	RPS11	Intron	1

Ac-H3K9	19	5.00E+07	5.00E+07	2346	298	2807.1	63.7	12.5	ENSG00000142534	RPS11	Up	1
Ac-H3K9	19	5.00E+07	5.00E+07	1731	58	285.35	18.4	0.54	ENSG00000142534	RPS11	Up	1
Ac-H3K9	19	5.00E+07	5.00E+07	1731	58	285.35	18.4	0.54	ENSG00000142541	RPL13A	Down	13
Ac-H3K9	19	5.00E+07	5.00E+07	1602	173	1514.7	59.3	1.53	ENSG00000142541	RPL13A	Down	13
Ac-H3K9	19	5.00E+07	5.00E+07	2346	298	2807.1	63.7	12.5	ENSG00000142541	RPL13A	Intron	13
Ac-H3K9	1	6.79E+07	6.79E+07	4024	289	1856.1	30.7	2.9	ENSG00000142864	SERBP1	Intron	8
Ac-H3K9	1	6.79E+07	6.79E+07	1252	51	130.95	8.7	0.68	ENSG00000142864	SERBP1	Up	8
Ac-H3K9	1	6.79E+07	6.79E+07	1328	89	470.76	20.4	0.43	ENSG00000142864	SERBP1	Up	8
Ac-H3K9	1	4.52E+07	4.52E+07	2156	180	1382.1	35.8	1.16	ENSG00000142959	BEST4	Down	1
Ac-H3K9	1	4.52E+07	4.52E+07	774	33	257.75	48.6	0.5	ENSG00000142959	BEST4	Down	1
Ac-H3K9	1	4.53E+07	4.53E+07	1017	23	126.18	15.0	0.68	ENSG00000142959	BEST4	Exon	1
Ac-H3K9	1	2.28E+08	2.28E+08	1064	54	283.14	17.5	0.55	ENSG00000143740	SNAP47	Intron	11
Ac-H3K9	1	2.26E+08	2.26E+08	3045	126	553.62	15.1	0.53	ENSG00000143799	PARP1	Intron	11
Ac-H3K9	1	2.24E+08	2.24E+08	657	33	168.1	16.1	0.6	ENSG00000143799	PARP1	Intron	11
Ac-H3K9	1	2.24E+08	2.24E+08	1620	99	650.66	29.1	0.55	ENSG00000143799	PARP1	Intron	11
Ac-H3K9	1	2.26E+08	2.26E+08	1294	79	407.72	20.7	0.38	ENSG00000143799	PARP1	Intron	11
Ac-H3K9	1	2.26E+08	2.26E+08	3111	266	1139.2	17.8	0.95	ENSG00000143799	PARP1	Intron	11
Ac-H3K9	1	2.26E+08	2.26E+08	1347	43	180.78	11.4	0.54	ENSG00000143799	PARP1	Intron	11
Ac-H3K9	1	2.26E+08	2.26E+08	1187	41	210.06	14.6	0.51	ENSG00000143799	PARP1	Intron	11
Ac-H3K9	1	2.26E+08	2.26E+08	681	26	104.31	10.0	0.75	ENSG00000143799	PARP1	Intron	11
Ac-H3K9	1	2.26E+08	2.26E+08	1507	99	452.33	19.1	0.45	ENSG00000143799	PARP1	Intron	11
Ac-H3K9	1	2.26E+08	2.26E+08	808	36	187.03	14.2	0.53	ENSG00000143799	PARP1	Intron	11
Ac-H3K9	1	2.26E+08	2.26E+08	3804	174	466.58	12.9	0.43	ENSG00000143799	PARP1	Intron	11
Ac-H3K9	1	2.26E+08	2.26E+08	1090	76	256.8	11.9	0.5	ENSG00000143799	PARP1	Intron	11
Ac-H3K9	1	2.26E+08	2.26E+08	1949	88	533.65	27.0	0.49	ENSG00000143799	PARP1	Intron	11
Ac-H3K9	1	2.26E+08	2.26E+08	3235	204	924.96	23.9	0.53	ENSG00000143799	PARP1	Intron	11
Ac-H3K9	1	2.26E+08	2.26E+08	3935	302	1365	24.1	1.1	ENSG00000143799	PARP1	Intron	11
Ac-H3K9	1	2.26E+08	2.26E+08	1314	50	154.05	11.5	0.64	ENSG00000143799	PARP1	Intron	11
Ac-H3K9	1	2.26E+08	2.26E+08	2372	87	426.55	16.8	0.4	ENSG00000143799	PARP1	Intron	11
Ac-H3K9	1	2.26E+08	2.26E+08	1141	65	303.63	17.7	0.49	ENSG00000143799	PARP1	Intron	11
Ac-H3K9	1	2.26E+08	2.26E+08	1647	96	368.16	18.6	0.41	ENSG00000143799	PARP1	Intron	11
Ac-H3K9	1	2.26E+08	2.26E+08	1128	67	252.37	13.8	0.51	ENSG00000143799	PARP1	Intron	11
Ac-H3K9	1	2.27E+08	2.27E+08	3173	186	952.07	30.7	0.58	ENSG00000143799	PARP1	Intron	11
Ac-H3K9	1	2.25E+08	2.25E+08	1632	80	436.05	34.5	0.42	ENSG00000143799	PARP1	Intron	11
Ac-H3K9	1	2.25E+08	2.25E+08	2554	118	305.66	9.4	0.49	ENSG00000143799	PARP1	Intron	11
Ac-H3K9	1	2.25E+08	2.25E+08	2136	119	620.97	22.1	0.57	ENSG00000143799	PARP1	Intron	11
Ac-H3K9	1	2.25E+08	2.25E+08	2765	240	1558.3	32.5	1.75	ENSG00000143799	PARP1	Intron	11
Ac-H3K9	1	2.25E+08	2.25E+08	1724	76	259.85	9.2	0.5	ENSG00000143799	PARP1	Intron	11
Ac-H3K9	1	2.24E+08	2.24E+08	914	39	151.69	13.3	0.65	ENSG00000143799	PARP1	Intron	11
Ac-H3K9	1	2.24E+08	2.24E+08	2034	104	594.64	27.2	0.59	ENSG00000143799	PARP1	Intron	11
Ac-H3K9	1	2.24E+08	2.24E+08	2428	151	569.49	13.4	0.55	ENSG00000143799	PARP1	Intron	11
Ac-H3K9	1	2.27E+08	2.27E+08	582	33	177.18	22.2	0.55	ENSG00000143799	PARP1	Up	11
Ac-H3K9	3	1.29E+07	1.29E+07	2779	169	1362.3	44.5	1.09	ENSG00000144712	CAND2	Intron	5
Ac-H3K9	3	1.28E+07	1.28E+07	526	14	81.24	12.3	0.8	ENSG00000144712	CAND2	Up	5
Ac-H3K9	3	1.29E+07	1.29E+07	2779	169	1362.3	44.5	1.09	ENSG00000144713	RPL32	Exon	8
Ac-H3K9	4	2.03E+07	2.03E+07	5685	262	1942.4	43.8	3.12	ENSG00000145147	SLIT2	Intron	12
Ac-H3K9	4	2.03E+07	2.03E+07	435	14	89.32	17.5	0.77	ENSG00000145147	SLIT2	Intron	12
Ac-H3K9	4	2.03E+07	2.03E+07	853	37	275.01	27.3	0.54	ENSG00000145147	SLIT2	Up	12
Ac-H3K9	4	8.34E+07	8.34E+07	1569	47	233.49	15.0	0.5	ENSG00000145293	ENOPH1	Intron	4
Ac-H3K9	4	8.33E+07	8.34E+07	2118	105	540.27	33.3	0.5	ENSG00000145293	ENOPH1	Up	4
Ac-H3K9	4	8.34E+07	8.34E+07	790	49	367.39	37.6	0.41	ENSG00000145293	ENOPH1	Up	4
Ac-H3K9	5	4.08E+07	4.08E+07	2084	156	953.66	23.0	0.58	ENSG00000145592	RPL37	Exon	6
Ac-H3K9	5	4.08E+07	4.08E+07	901	56	337.07	23.7	0.45	ENSG00000145592	RPL37	Up	6
Ac-H3K9	5	1.81E+08	1.81E+08	5156	281	1658.6	28.6	2.15	ENSG00000146063	TRIM41	Down	11
Ac-H3K9	5	1.81E+08	1.81E+08	3088	266	2358.1	57.4	6.67	ENSG00000146063	TRIM41	Down	11
Ac-H3K9	5	1.81E+08	1.81E+08	975	34	224.08	29.0	0.55	ENSG00000146063	TRIM41	Exon	11
Ac-H3K9	5	1.81E+08	1.81E+08	647	17	101.52	15.3	0.76	ENSG00000146063	TRIM41	Intron	11
Ac-H3K9	5	1.81E+08	1.81E+08	1238	33	179.27	14.9	0.55	ENSG00000146063	TRIM41	Up	11
Ac-H3K9	6	1.37E+08	1.37E+08	1171	46	194.74	10.2	0.54	ENSG00000146410	FAM54A	Exon	7
Ac-H3K9	6	1.37E+08	1.37E+08	1111	47	209.04	17.7	0.53	ENSG00000146410	FAM54A	Up	7
Ac-H3K9	X	9.59E+07	9.59E+07	2265	101	471.93	15.8	0.44	ENSG00000147202	DIAPH2	Intron	6
Ac-H3K9	4	8.34E+07	8.34E+07	790	49	367.39	37.6	0.41	ENSG00000152795	HNRPDL	Exon	5
Ac-H3K9	4	8.33E+07	8.34E+07	2118	105	540.27	33.3	0.5	ENSG00000152795	HNRPDL	Intron	5
Ac-H3K9	4	8.34E+07	8.34E+07	1569	47	233.49	15.0	0.5	ENSG00000152795	HNRPDL	Up	5
Ac-H3K9	1	2.45E+08	2.45E+08	2906	248	1161.8	17.9	1.01	ENSG00000153187	HNRNPU	Exon	9
Ac-H3K9	1	2.45E+08	2.45E+08	1146	106	715.43	23.1	0.57	ENSG00000153187	HNRNPU	Exon	9
Ac-H3K9	1	2.45E+08	2.45E+08	2663	187	798.25	24.0	0.36	ENSG00000153187	HNRNPU	Up	9
Ac-H3K9	1	2.45E+08	2.45E+08	1276	50	197.58	12.8	0.51	ENSG00000153187	HNRNPU	Up	9

Ac-H3K9	4	7.34E+07	7.34E+07	2865	89	302.32	11.4	0.48	ENSG00000156140	ADAMTS3	Intron	3
Ac-H3K9	6	7.42E+07	7.42E+07	1334	38	198.42	13.5	0.52	ENSG00000156508	EEF1A1	Exon	12
Ac-H3K9	6	7.42E+07	7.42E+07	2409	230	2086	46.3	3.92	ENSG00000156508	EEF1A1	Intron	12
Ac-H3K9	6	7.42E+07	7.42E+07	962	37	265.44	25.3	0.52	ENSG00000156508	EEF1A1	Up	12
Ac-H3K9	3	1.86E+08	1.87E+08	4178	296	2539.6	42.2	9.09	ENSG00000156976	EIF4A2	Exon	28
Ac-H3K9	21	4.45E+07	4.45E+07	1834	83	647.73	33.7	0.54	ENSG00000160200	CBS	Exon	20
Ac-H3K9	21	4.45E+07	4.45E+07	743	22	145.22	20.6	0.65	ENSG00000160200	CBS	Exon	20
Ac-H3K9	21	4.45E+07	4.45E+07	643	18	117.91	18.7	0.74	ENSG00000160200	CBS	Up	20
Ac-H3K9	22	2.20E+07	2.20E+07	1819	72	475.91	37.8	0.44	ENSG00000161179	YDJC	Exon	7
Ac-H3K9	22	2.20E+07	2.20E+07	1085	40	130.75	9.5	0.68	ENSG00000161180	CCDC116	Down	2
Ac-H3K9	22	2.20E+07	2.20E+07	1225	37	191.95	13.9	0.54	ENSG00000161180	CCDC116	Down	2
Ac-H3K9	22	2.20E+07	2.20E+07	1819	72	475.91	37.8	0.44	ENSG00000161180	CCDC116	Up	2
Ac-H3K9	17	7.47E+07	7.47E+07	1098	32	109.38	13.2	0.75	ENSG00000161547	SRSF2	Down	5
Ac-H3K9	17	7.47E+07	7.47E+07	3446	188	1255.8	33.1	0.84	ENSG00000161547	SRSF2	Exon	5
Ac-H3K9	17	7.47E+07	7.47E+07	801	20	87.16	8.6	0.78	ENSG00000161547	SRSF2	Up	5
Ac-H3K9	17	7.47E+07	7.47E+07	1185	42	289.73	27.9	0.55	ENSG00000161547	SRSF2	Up	5
Ac-H3K9	17	7.48E+06	7.48E+06	1997	95	468.76	16.7	0.43	ENSG00000161956	SENP3	Down	2
Ac-H3K9	17	7.46E+06	7.47E+06	2372	100	663.9	35.3	0.57	ENSG00000161956	SENP3	Up	2
Ac-H3K9	17	7.49E+06	7.49E+06	1797	93	545.39	37.8	0.51	ENSG00000161960	EIF4A1	Down	3
Ac-H3K9	17	7.48E+06	7.48E+06	1997	95	468.76	16.7	0.43	ENSG00000161960	EIF4A1	Intron	3
Ac-H3K9	17	7.46E+06	7.47E+06	2372	100	663.9	35.3	0.57	ENSG00000161960	EIF4A1	Up	3
Ac-H3K9	1	7.84E+07	7.84E+07	5392	469	2616.8	36.0	11.1	ENSG00000162613	FUBP1	Intron	16
Ac-H3K9	1	7.84E+07	7.84E+07	1295	46	166.55	15.1	0.62	ENSG00000162614	NEXN	Intron	10
Ac-H3K9	1	2.26E+08	2.26E+08	3111	266	1139.2	17.8	0.95	ENSG00000163041	H3F3A	Intron	4
Ac-H3K9	1	2.26E+08	2.26E+08	1294	79	407.72	20.7	0.38	ENSG00000163041	H3F3A	Up	4
Ac-H3K9	3	1.86E+08	1.87E+08	4178	296	2539.6	42.2	9.09	ENSG00000163918	RFC4	Down	14
Ac-H3K9	3	1.87E+08	1.87E+08	1965	102	846	37.4	0.42	ENSG00000163918	RFC4	Intron	14
Ac-H3K9	9	8.66E+07	8.66E+07	1307	39	229.99	20.5	0.51	ENSG00000165118	C9orf64	Exon	3
Ac-H3K9	9	8.66E+07	8.66E+07	4715	280	2126.3	55.6	4.17	ENSG00000165119	HNRNPK	Intron	15
Ac-H3K9	14	3.49E+07	3.49E+07	838	20	83.71	7.4	0.79	ENSG00000165389	C14orf147	Intron	1
Ac-H3K9	14	3.49E+07	3.49E+07	939	41	186.17	11.5	0.53	ENSG00000165389	C14orf147	Intron	1
Ac-H3K9	14	3.49E+07	3.49E+07	560	14	88.66	14.9	0.76	ENSG00000165389	C14orf147	Up	1
Ac-H3K9	15	7.96E+07	7.96E+07	1128	46	325.33	25.6	0.5	ENSG00000166557	TMED3	Intron	4
Ac-H3K9	18	3.26E+07	3.26E+07	996	22	82.82	8.4	0.79	ENSG00000166974	MAPRE2	Intron	4
Ac-H3K9	18	3.26E+07	3.26E+07	2329	105	379.05	16.2	0.4	ENSG00000166974	MAPRE2	Intron	4
Ac-H3K9	19	5.69E+06	5.69E+06	1493	119	1177.2	54.1	1.06	ENSG00000167733	HSD11B1L	Down	6
Ac-H3K9	19	5.68E+06	5.68E+06	829	27	184.52	20.6	0.54	ENSG00000167733	HSD11B1L	Up	6
Ac-H3K9	19	5.13E+07	5.13E+07	2108	174	1759.9	70.2	2.47	ENSG00000167747	C19orf48	Intron	2
Ac-H3K9	3	3.94E+07	3.95E+07	3059	227	1491.2	34.2	1.46	ENSG00000168028	RPSA	Intron	10
Ac-H3K9	2	8.58E+07	8.58E+07	4623	297	1956.4	41.9	3.28	ENSG00000168906	MAT2A	Up	7
Ac-H3K9	2	1.78E+08	1.78E+08	529	19	115	17.0	0.75	ENSG00000170144	HNRNPA3	Intron	8
Ac-H3K9	2	1.78E+08	1.78E+08	1403	136	775.67	25.3	0.34	ENSG00000170144	HNRNPA3	Intron	8
Ac-H3K9	2	1.78E+08	1.78E+08	1555	152	956.7	19.0	0.59	ENSG00000170144	HNRNPA3	Up	8
Ac-H3K9	19	5.47E+07	5.47E+07	1763	109	791.19	34.4	0.36	ENSG00000170889	RPS9	Exon	12
Ac-H3K9	19	5.47E+07	5.47E+07	1149	28	113.31	11.0	0.75	ENSG00000170889	RPS9	Intron	12
Ac-H3K9	13	4.59E+07	4.59E+07	1159	36	204.49	18.4	0.53	ENSG00000170919	XXyac-R12DG2.2	Intron	21
Ac-H3K9	13	4.59E+07	4.59E+07	1978	114	955.13	50.3	0.58	ENSG00000170919	XXyac-R12DG2.2	Up	21
Ac-H3K9	15	6.61E+07	6.61E+07	1411	73	487.79	22.1	0.46	ENSG00000174485	DENND4A	Intron	3
Ac-H3K9	15	6.61E+07	6.61E+07	828	46	337.34	24.0	0.45	ENSG00000174485	DENND4A	Up	3
Ac-H3K9	3	2.40E+07	2.40E+07	1949	125	845.87	22.2	0.42	ENSG00000174748	RPL15	Exon	14
Ac-H3K9	3	2.40E+07	2.40E+07	1258	47	136.45	9.8	0.69	ENSG00000174748	RPL15	Up	14
Ac-H3K9	17	8.08E+06	8.08E+06	355	25	196.14	26.1	0.51	ENSG00000179029	TMEM107	Exon	10
Ac-H3K9	17	8.09E+06	8.09E+06	435	23	148.79	14.3	0.64	ENSG00000179029	TMEM107	Up	10
Ac-H3K9	16	2.02E+06	2.02E+06	1430	51	349.89	44.9	0.44	ENSG00000179580	RNF151	Down	1
Ac-H3K9	16	2.01E+06	2.01E+06	1486	70	416.63	25.5	0.39	ENSG00000179580	RNF151	Up	1
Ac-H3K9	16	2.01E+06	2.01E+06	1124	32	92.25	8.4	0.77	ENSG00000179580	RNF151	Up	1
Ac-H3K9	16	2.01E+06	2.01E+06	2609	200	982.64	18.4	0.62	ENSG00000179580	RNF151	Up	1
Ac-H3K9	16	2.01E+06	2.02E+06	1300	61	415.46	28.5	0.39	ENSG00000179580	RNF151	Up	1
Ac-H3K9	1	2.88E+07	2.88E+07	1651	90	492.75	24.5	0.47	ENSG00000180198	RCC1	Intron	12
Ac-H3K9	1	2.88E+07	2.88E+07	6730	807	3100	40.7	16.7	ENSG00000180198	RCC1	Up	12
Ac-H3K9	17	7.47E+07	7.47E+07	3446	188	1255.8	33.1	0.84	ENSG00000181038	C17orf95	Down	2
Ac-H3K9	17	7.47E+07	7.47E+07	1185	42	289.73	27.9	0.55	ENSG00000181038	C17orf95	Down	2
Ac-H3K9	17	7.47E+07	7.47E+07	801	20	87.16	8.6	0.78	ENSG00000181038	C17orf95	Down	2
Ac-H3K9	17	7.47E+07	7.47E+07	1098	32	109.38	13.2	0.75	ENSG00000181038	C17orf95	Intron	2
Ac-H3K9	5	1.71E+08	1.71E+08	927	144	1396.5	65.7	1.18	ENSG00000181163	NPM1	Intron	12
Ac-H3K9	5	1.71E+08	1.71E+08	3819	243	1530.2	34.5	1.57	ENSG00000181163	NPM1	Intron	12
Ac-H3K9	22	4.30E+07	4.30E+07	1371	81	625.83	39.3	0.51	ENSG00000182841	RP1-222E13.10	Intron	4
Ac-H3K9	22	4.30E+07	4.30E+07	1371	81	625.83	39.3	0.51	ENSG00000183569	SERHL2	Down	11

Ac-H3K9	22	3.93E+07	3.93E+07	1414	45	255.26	19.9	0.49	ENSG00000183741	CBX6	Intron	3
Ac-H3K9	8	3.76E+07	3.76E+07	5586	388	2740.4	51.4	20	ENSG00000183779	ZNF703	Exon	2
Ac-H3K9	8	3.76E+07	3.76E+07	1801	77	585.26	29.9	0.58	ENSG00000183779	ZNF703	Intron	2
Ac-H3K9	8	3.75E+07	3.75E+07	748	20	92.22	13.5	0.77	ENSG00000183779	ZNF703	Up	2
Ac-H3K9	8	3.76E+07	3.76E+07	1164	60	498.3	31.8	0.48	ENSG00000183779	ZNF703	Up	2
Ac-H3K9	1	1.74E+08	1.74E+08	716	23	119.88	17.2	0.73	ENSG00000185278	ZBTB37	Intron	7
Ac-H3K9	1	1.74E+08	1.74E+08	1513	38	108.04	7.0	0.76	ENSG00000185278	ZBTB37	Up	7
Ac-H3K9	1	1.74E+08	1.74E+08	2862	311	2345.4	29.1	6.25	ENSG00000185278	ZBTB37	Up	7
Ac-H3K9	10	5.28E+07	5.28E+07	1083	28	164.03	14.9	0.64	ENSG00000185532	PRKG1	Exon	6
Ac-H3K9	10	5.28E+07	5.28E+07	776	24	115.49	10.4	0.75	ENSG00000185532	PRKG1	Intron	6
Ac-H3K9	10	5.35E+07	5.35E+07	1772	99	680.92	29.5	0.52	ENSG00000185532	PRKG1	Intron	6
Ac-H3K9	17	7.98E+07	7.98E+07	784	26	180.31	26.2	0.54	ENSG00000185624	P4HB	Down	16
Ac-H3K9	17	7.98E+07	7.98E+07	1000	41	239.09	27.0	0.48	ENSG00000185624	P4HB	Intron	16
Ac-H3K9	17	7.98E+07	7.98E+07	1491	59	426.33	31.8	0.4	ENSG00000185624	P4HB	Up	16
Ac-H3K9	17	7.98E+07	7.98E+07	956	49	402.69	36.1	0.37	ENSG00000185624	P4HB	Up	16
Ac-H3K9	22	2.20E+07	2.20E+07	1819	72	475.91	37.8	0.44	ENSG00000185651	UBE2L3	Down	4
Ac-H3K9	22	2.19E+07	2.19E+07	2444	89	274.12	10.9	0.53	ENSG00000185651	UBE2L3	Intron	4
Ac-H3K9	12	7.65E+07	7.65E+07	600	16	93.98	16.1	0.78	ENSG00000187109	NAP1L1	Intron	7
Ac-H3K9	12	7.65E+07	7.65E+07	2153	86	480.7	20.9	0.45	ENSG00000187109	NAP1L1	Intron	7
Ac-H3K9	2	2.33E+08	2.33E+08	1261	39	244.38	23.5	0.49	ENSG00000187514	PTMA	Exon	13
Ac-H3K9	2	2.33E+08	2.33E+08	1651	64	455.31	43.0	0.41	ENSG00000187514	PTMA	Exon	13
Ac-H3K9	2	2.33E+08	2.33E+08	854	58	549.31	43.0	0.52	ENSG00000187514	PTMA	Intron	13
Ac-H3K9	2	2.33E+08	2.33E+08	1013	53	446.07	35.5	0.44	ENSG00000187514	PTMA	Up	13
Ac-H3K9	2	2.35E+08	2.35E+08	981	26	112.52	10.8	0.75	ENSG00000188042	ARL4C	Exon	2
Ac-H3K9	2	2.35E+08	2.35E+08	526	14	84.12	17.8	0.79	ENSG00000188042	ARL4C	Up	2
Ac-H3K9	1	2.45E+08	2.45E+08	4621	294	1214.2	20.7	1.17	ENSG00000188206	NCRNA00201	Down	3
Ac-H3K9	1	2.45E+08	2.45E+08	2663	187	798.25	24.0	0.36	ENSG00000188206	NCRNA00201	Up	3
Ac-H3K9	1	2.45E+08	2.45E+08	1146	106	715.43	23.1	0.57	ENSG00000188206	NCRNA00201	Up	3
Ac-H3K9	1	2.45E+08	2.45E+08	2906	248	1161.8	17.9	1.01	ENSG00000188206	NCRNA00201	Up	3
Ac-H3K9	1	9.35E+05	9.35E+05	686	18	113.15	18.7	0.75	ENSG00000188290	HES4	Exon	4
Ac-H3K9	1	9.36E+05	9.36E+05	594	28	210.43	30.7	0.51	ENSG00000188290	HES4	Up	4
Ac-H3K9	1	9.36E+05	9.37E+05	1037	68	511.34	31.9	0.46	ENSG00000188290	HES4	Up	4
Ac-H3K9	19	5.69E+06	5.69E+06	1493	119	1177.2	54.1	1.06	ENSG00000196365	LONP1	Down	3
Ac-H3K9	19	5.72E+06	5.72E+06	1702	90	757.62	48.6	0.43	ENSG00000196365	LONP1	Intron	3
Ac-H3K9	19	5.72E+06	5.72E+06	346	18	165.04	31.8	0.63	ENSG00000196365	LONP1	Up	3
Ac-H3K9	3	2.40E+07	2.40E+07	1258	47	136.45	9.8	0.69	ENSG00000197885	NKIRAS1	Exon	8
Ac-H3K9	3	2.40E+07	2.40E+07	934	34	215.55	24.5	0.53	ENSG00000197885	NKIRAS1	Intron	8
Ac-H3K9	3	2.40E+07	2.40E+07	1949	125	845.87	22.2	0.42	ENSG00000197885	NKIRAS1	Intron	8
Ac-H3K9	5	1.77E+08	1.77E+08	1472	80	686.59	35.5	0.53	ENSG00000198055	GRK6	Intron	11
Ac-H3K9	13	9.20E+07	9.20E+07	1257	30	89.04	6.5	0.77	ENSG00000199149	MIR20A	Down	1
Ac-H3K9	13	9.20E+07	9.20E+07	1574	81	398.62	17.5	0.36	ENSG00000199149	MIR20A	Up	1
Ac-H3K9	13	9.20E+07	9.20E+07	1108	31	94.77	9.8	0.78	ENSG00000199149	MIR20A	Up	1
Ac-H3K9	13	9.20E+07	9.20E+07	1680	286	2213.6	36.8	4.88	ENSG00000199149	MIR20A	Up	1
Ac-H3K9	13	9.20E+07	9.20E+07	1257	30	89.04	6.5	0.77	ENSG00000199180	MIR18A	Down	1
Ac-H3K9	13	9.20E+07	9.20E+07	1574	81	398.62	17.5	0.36	ENSG00000199180	MIR18A	Up	1
Ac-H3K9	13	9.20E+07	9.20E+07	1108	31	94.77	9.8	0.78	ENSG00000199180	MIR18A	Up	1
Ac-H3K9	13	9.20E+07	9.20E+07	1680	286	2213.6	36.8	4.88	ENSG00000199180	MIR18A	Up	1
Ac-H3K9	17	5.67E+07	5.67E+07	924	37	202.7	20.4	0.53	ENSG00000199426	U1.5	Down	1
Ac-H3K9	13	4.59E+07	4.59E+07	1159	36	204.49	18.4	0.53	ENSG00000199477	SNORA31	Up	1
Ac-H3K9	13	4.59E+07	4.59E+07	1978	114	955.13	50.3	0.58	ENSG00000199477	SNORA31	Up	1
Ac-H3K9	19	5.00E+07	5.00E+07	1731	58	285.35	18.4	0.54	ENSG00000199631	SNORD33	Down	1
Ac-H3K9	19	5.00E+07	5.00E+07	1602	173	1514.7	59.3	1.53	ENSG00000199631	SNORD33	Down	1
Ac-H3K9	19	5.00E+07	5.00E+07	2346	298	2807.1	63.7	12.5	ENSG00000199631	SNORD33	Up	1
Ac-H3K9	17	6.22E+07	6.22E+07	1648	106	627.31	21.9	0.51	ENSG00000199753	SNORD104	Down	1
Ac-H3K9	17	6.22E+07	6.22E+07	919	25	107.58	11.6	0.75	ENSG00000199753	SNORD104	Up	1
Ac-H3K9	1	1.74E+08	1.74E+08	1513	38	108.04	7.0	0.76	ENSG00000200016	SNORD76	Down	1
Ac-H3K9	1	1.74E+08	1.74E+08	716	23	119.88	17.2	0.73	ENSG00000200016	SNORD76	Up	1
Ac-H3K9	1	1.74E+08	1.74E+08	2862	311	2345.4	29.1	6.25	ENSG00000200016	SNORD76	Up	1
Ac-H3K9	1	2.88E+07	2.88E+07	1651	90	492.75	24.5	0.47	ENSG00000200087	SNORA73B	Down	1
Ac-H3K9	1	2.88E+07	2.88E+07	6730	807	3100	40.7	16.7	ENSG00000200087	SNORA73B	Up	1
Ac-H3K9	17	1.90E+07	1.90E+07	1007	51	383.03	49.4	0.41	ENSG00000200229	SNORD3B-1	Down	1
Ac-H3K9	17	1.90E+07	1.90E+07	878	26	107.51	13.3	0.75	ENSG00000200229	SNORD3B-1	Down	1
Ac-H3K9	17	1.90E+07	1.90E+07	663	23	165.95	33.7	0.63	ENSG00000200229	SNORD3B-1	Down	1
Ac-H3K9	17	1.90E+07	1.90E+07	724	50	371.88	36.6	0.42	ENSG00000200229	SNORD3B-1	Up	1
Ac-H3K9	19	5.00E+07	5.00E+07	1731	58	285.35	18.4	0.54	ENSG00000200259	SNORD35A	Down	1
Ac-H3K9	19	5.00E+07	5.00E+07	1602	173	1514.7	59.3	1.53	ENSG00000200259	SNORD35A	Down	1
Ac-H3K9	19	5.00E+07	5.00E+07	2346	298	2807.1	63.7	12.5	ENSG00000200259	SNORD35A	Up	1
Ac-H3K9	3	1.86E+08	1.87E+08	4178	296	2539.6	42.2	9.09	ENSG00000200320	SNORA63	Up	1

Ac-H3K9	3	1.86E+08	1.87E+08	4178	296	2539.6	42.2	9.09	ENSG00000200418	SNORA63.6	Up	1
Ac-H3K9	17	8.08E+06	8.08E+06	355	25	196.14	26.1	0.51	ENSG00000200463	U8.4	Up	1
Ac-H3K9	19	5.00E+07	5.00E+07	2346	298	2807.1	63.7	12.5	ENSG00000200530	SNORD35B	Up	1
Ac-H3K9	19	5.00E+07	5.00E+07	1731	58	285.35	18.4	0.54	ENSG00000200530	SNORD35B	Up	1
Ac-H3K9	19	5.00E+07	5.00E+07	1602	173	1514.7	59.3	1.53	ENSG00000200530	SNORD35B	Up	1
Ac-H3K9	1	1.74E+08	1.74E+08	1513	38	108.04	7.0	0.76	ENSG00000200710	SNORD81	Up	1
Ac-H3K9	1	1.74E+08	1.74E+08	716	23	119.88	17.2	0.73	ENSG00000200710	SNORD81	Up	1
Ac-H3K9	1	1.74E+08	1.74E+08	2862	311	2345.4	29.1	6.25	ENSG00000200710	SNORD81	Up	1
Ac-H3K9	1	1.74E+08	1.74E+08	1513	38	108.04	7.0	0.76	ENSG00000200729	SNORD79	Down	1
Ac-H3K9	1	1.74E+08	1.74E+08	716	23	119.88	17.2	0.73	ENSG00000200729	SNORD79	Up	1
Ac-H3K9	1	1.74E+08	1.74E+08	2862	311	2345.4	29.1	6.25	ENSG00000200729	SNORD79	Up	1
Ac-H3K9	1	1.74E+08	1.74E+08	1513	38	108.04	7.0	0.76	ENSG00000200954	SNORD74	Down	1
Ac-H3K9	1	1.74E+08	1.74E+08	2862	311	2345.4	29.1	6.25	ENSG00000200954	SNORD74	Down	1
Ac-H3K9	1	1.74E+08	1.74E+08	716	23	119.88	17.2	0.73	ENSG00000200954	SNORD74	Up	1
Ac-H3K9	17	5.68E+07	5.68E+07	504	29	159.49	19.2	0.62	ENSG00000200997	U1.36	Up	1
Ac-H3K9	1	7.62E+07	7.63E+07	6074	709	3100	25.9	16.7	ENSG00000201487	SNORD45B	Up	1
Ac-H3K9	19	5.00E+07	5.00E+07	1731	58	285.35	18.4	0.54	ENSG00000201675	SNORD32A	Down	1
Ac-H3K9	19	5.00E+07	5.00E+07	1602	173	1514.7	59.3	1.53	ENSG00000201675	SNORD32A	Down	1
Ac-H3K9	19	5.00E+07	5.00E+07	2346	298	2807.1	63.7	12.5	ENSG00000201675	SNORD32A	Up	1
Ac-H3K9	1	1.74E+08	1.74E+08	1513	38	108.04	7.0	0.76	ENSG00000201692	SNORD80	Down	1
Ac-H3K9	1	1.74E+08	1.74E+08	716	23	119.88	17.2	0.73	ENSG00000201692	SNORD80	Up	1
Ac-H3K9	1	1.74E+08	1.74E+08	2862	311	2345.4	29.1	6.25	ENSG00000201692	SNORD80	Up	1
Ac-H3K9	17	1.90E+07	1.90E+07	878	26	107.51	13.3	0.75	ENSG00000201750	SNORD3B-2	Down	1
Ac-H3K9	17	1.90E+07	1.90E+07	724	50	371.88	36.6	0.42	ENSG00000201750	SNORD3B-2	Down	1
Ac-H3K9	17	1.90E+07	1.90E+07	1007	51	383.03	49.4	0.41	ENSG00000201750	SNORD3B-2	Up	1
Ac-H3K9	17	1.90E+07	1.90E+07	663	23	165.95	33.7	0.63	ENSG00000201750	SNORD3B-2	Up	1
Ac-H3K9	1	2.88E+07	2.88E+07	6730	807	3100	40.7	16.7	ENSG00000201808	SNORA73A	Up	1
Ac-H3K9	6	8.64E+07	8.64E+07	2833	229	1353	25.9	1.05	ENSG00000201865	SNORD50B	Down	1
Ac-H3K9	6	8.64E+07	8.64E+07	1691	90	438.28	20.7	0.42	ENSG00000201865	SNORD50B	Up	1
Ac-H3K9	17	5.67E+07	5.67E+07	924	37	202.7	20.4	0.53	ENSG00000202077	U1.57	Up	1
Ac-H3K9	3	3.94E+07	3.95E+07	3059	227	1491.2	34.2	1.46	ENSG00000202363	SNORA62.4	Up	1
Ac-H3K9	1	1.74E+08	1.74E+08	1513	38	108.04	7.0	0.76	ENSG00000202394	SNORD47	Up	1
Ac-H3K9	1	1.74E+08	1.74E+08	716	23	119.88	17.2	0.73	ENSG00000202394	SNORD47	Up	1
Ac-H3K9	1	1.74E+08	1.74E+08	2862	311	2345.4	29.1	6.25	ENSG00000202394	SNORD47	Up	1
Ac-H3K9	19	5.00E+07	5.00E+07	1731	58	285.35	18.4	0.54	ENSG00000202503	SNORD34	Down	1
Ac-H3K9	19	5.00E+07	5.00E+07	1602	173	1514.7	59.3	1.53	ENSG00000202503	SNORD34	Down	1
Ac-H3K9	19	5.00E+07	5.00E+07	2346	298	2807.1	63.7	12.5	ENSG00000202503	SNORD34	Up	1
Ac-H3K9	1	2.45E+08	2.45E+08	4621	294	1214.2	20.7	1.17	ENSG00000203667	FAM36A	Up	5
Ac-H3K9	6	8.64E+07	8.64E+07	2833	229	1353	25.9	1.05	ENSG00000203875	SNHG5	Exon	10
Ac-H3K9	6	8.64E+07	8.64E+07	1691	90	438.28	20.7	0.42	ENSG00000203875	SNHG5	Up	10
Ac-H3K9	1	2.88E+07	2.88E+07	6730	807	3100	40.7	16.7	ENSG00000204138	PHACTR4	Down	5
Ac-H3K9	1	2.87E+07	2.87E+07	1256	70	319.85	15.7	0.49	ENSG00000204138	PHACTR4	Intron	5
Ac-H3K9	6	3.29E+07	3.29E+07	3378	243	1977.6	41.3	3.45	ENSG00000204256	BRD2	Intron	16
Ac-H3K9	6	3.29E+07	3.29E+07	4124	230	1585.6	35.4	1.89	ENSG00000204256	BRD2	Intron	16
Ac-H3K9	6	3.29E+07	3.29E+07	662	32	262.59	28.9	0.51	ENSG00000204256	BRD2	Up	16
Ac-H3K9	5	1.81E+08	1.81E+08	3088	266	2358.1	57.4	6.67	ENSG00000204628	GNB2L1	Exon	35
Ac-H3K9	5	1.81E+08	1.81E+08	5156	281	1658.6	28.6	2.15	ENSG00000204628	GNB2L1	Intron	35
Ac-H3K9	2	1.02E+08	1.02E+08	2273	152	1290.1	44.5	0.92	ENSG00000204634	TBC1D8	Down	10
Ac-H3K9	2	1.02E+08	1.02E+08	545	23	128.03	11.3	0.68	ENSG00000204634	TBC1D8	Intron	10
Ac-H3K9	2	1.02E+08	1.02E+08	1142	36	168.79	11.7	0.6	ENSG00000204634	TBC1D8	Up	10
Ac-H3K9	14	3.50E+07	3.50E+07	481	14	84.12	14.8	0.79	ENSG00000206588	RNU1-8	Down	1
Ac-H3K9	14	3.50E+07	3.50E+07	632	21	149.4	31.8	0.64	ENSG00000206588	RNU1-8	Up	1
Ac-H3K9	14	3.50E+07	3.50E+07	632	21	149.4	31.8	0.64	ENSG00000206596	RNU1-7	Down	1
Ac-H3K9	14	3.50E+07	3.50E+07	1230	69	593.84	44.9	0.59	ENSG00000206596	RNU1-7	Up	1
Ac-H3K9	14	3.50E+07	3.50E+07	481	14	84.12	14.8	0.79	ENSG00000206596	RNU1-7	Up	1
Ac-H3K9	1	1.74E+08	1.74E+08	1513	38	108.04	7.0	0.76	ENSG00000206607	SNORD44	Down	1
Ac-H3K9	1	1.74E+08	1.74E+08	716	23	119.88	17.2	0.73	ENSG00000206607	SNORD44	Up	1
Ac-H3K9	1	1.74E+08	1.74E+08	2862	311	2345.4	29.1	6.25	ENSG00000206607	SNORD44	Up	1
Ac-H3K9	1	7.62E+07	7.63E+07	6074	709	3100	25.9	16.7	ENSG00000206620	SNORD45C	Up	1
Ac-H3K9	3	3.94E+07	3.95E+07	3059	227	1491.2	34.2	1.46	ENSG00000206760	SNORA6	Up	1
Ac-H3K9	16	2.01E+06	2.01E+06	1124	32	92.25	8.4	0.77	ENSG00000206811	SNORA10	Down	1
Ac-H3K9	16	2.01E+06	2.01E+06	1486	70	416.63	25.5	0.39	ENSG00000206811	SNORA10	Down	1
Ac-H3K9	16	2.02E+06	2.02E+06	1430	51	349.89	44.9	0.44	ENSG00000206811	SNORA10	Up	1
Ac-H3K9	16	2.01E+06	2.02E+06	1300	61	415.46	28.5	0.39	ENSG00000206811	SNORA10	Up	1
Ac-H3K9	16	2.01E+06	2.01E+06	2609	200	982.64	18.4	0.62	ENSG00000206811	SNORA10	Up	1
Ac-H3K9	17	5.68E+07	5.68E+07	504	29	159.49	19.2	0.62	ENSG00000206917	U1.78	Up	1
Ac-H3K9	6	8.64E+07	8.64E+07	2833	229	1353	25.9	1.05	ENSG00000207066	SNORD50A	Down	1
Ac-H3K9	6	8.64E+07	8.64E+07	1691	90	438.28	20.7	0.42	ENSG00000207066	SNORD50A	Up	1

Ac-H3K9	17	7.49E+06	7.49E+06	1797	93	545.39	37.8	0.51	ENSG00000207152	SNORA67	Down	1
Ac-H3K9	17	7.48E+06	7.48E+06	1997	95	468.76	16.7	0.43	ENSG00000207152	SNORA67	Up	1
Ac-H3K9	1	7.62E+07	7.63E+07	6074	709	3100	25.9	16.7	ENSG00000207241	SNORD45A	Up	1
Ac-H3K9	16	2.01E+06	2.01E+06	1124	32	92.25	8.4	0.77	ENSG00000207405	SNORA64	Down	1
Ac-H3K9	16	2.01E+06	2.01E+06	1486	70	416.63	25.5	0.39	ENSG00000207405	SNORA64	Down	1
Ac-H3K9	16	2.02E+06	2.02E+06	1430	51	349.89	44.9	0.44	ENSG00000207405	SNORA64	Up	1
Ac-H3K9	16	2.01E+06	2.02E+06	1300	61	415.46	28.5	0.39	ENSG00000207405	SNORA64	Up	1
Ac-H3K9	16	2.01E+06	2.01E+06	2609	200	982.64	18.4	0.62	ENSG00000207405	SNORA64	Up	1
Ac-H3K9	3	1.29E+07	1.29E+07	2779	169	1362.3	44.5	1.09	ENSG00000207496	SNORA7A	Up	1
Ac-H3K9	17	7.47E+07	7.47E+07	3446	188	1255.8	33.1	0.84	ENSG00000207556	MIR636	Down	1
Ac-H3K9	17	7.47E+07	7.47E+07	1098	32	109.38	13.2	0.75	ENSG00000207556	MIR636	Down	1
Ac-H3K9	17	7.47E+07	7.47E+07	801	20	87.16	8.6	0.78	ENSG00000207556	MIR636	Up	1
Ac-H3K9	17	7.47E+07	7.47E+07	1185	42	289.73	27.9	0.55	ENSG00000207556	MIR636	Up	1
Ac-H3K9	13	9.20E+07	9.20E+07	1257	30	89.04	6.5	0.77	ENSG00000207560	MIR19B1	Down	1
Ac-H3K9	13	9.20E+07	9.20E+07	1574	81	398.62	17.5	0.36	ENSG00000207560	MIR19B1	Up	1
Ac-H3K9	13	9.20E+07	9.20E+07	1108	31	94.77	9.8	0.78	ENSG00000207560	MIR19B1	Up	1
Ac-H3K9	13	9.20E+07	9.20E+07	1680	286	2213.6	36.8	4.88	ENSG00000207560	MIR19B1	Up	1
Ac-H3K9	9	8.66E+07	8.66E+07	4715	280	2126.3	55.6	4.17	ENSG00000207603	MIR7-1	Up	1
Ac-H3K9	13	9.20E+07	9.20E+07	1257	30	89.04	6.5	0.77	ENSG00000207610	MIR19A	Down	1
Ac-H3K9	13	9.20E+07	9.20E+07	1574	81	398.62	17.5	0.36	ENSG00000207610	MIR19A	Up	1
Ac-H3K9	13	9.20E+07	9.20E+07	1108	31	94.77	9.8	0.78	ENSG00000207610	MIR19A	Up	1
Ac-H3K9	13	9.20E+07	9.20E+07	1680	286	2213.6	36.8	4.88	ENSG00000207610	MIR19A	Up	1
Ac-H3K9	13	9.20E+07	9.20E+07	1257	30	89.04	6.5	0.77	ENSG00000207745	MIR17	Down	1
Ac-H3K9	13	9.20E+07	9.20E+07	1574	81	398.62	17.5	0.36	ENSG00000207745	MIR17	Up	1
Ac-H3K9	13	9.20E+07	9.20E+07	1108	31	94.77	9.8	0.78	ENSG00000207745	MIR17	Up	1
Ac-H3K9	13	9.20E+07	9.20E+07	1680	286	2213.6	36.8	4.88	ENSG00000207745	MIR17	Up	1
Ac-H3K9	19	5.00E+07	5.00E+07	1602	173	1514.7	59.3	1.53	ENSG00000207782	MIR150	Down	1
Ac-H3K9	19	5.00E+07	5.00E+07	1731	58	285.35	18.4	0.54	ENSG00000207782	MIR150	Down	1
Ac-H3K9	13	9.20E+07	9.20E+07	1257	30	89.04	6.5	0.77	ENSG00000207968	MIR92A1	Down	1
Ac-H3K9	13	9.20E+07	9.20E+07	1574	81	398.62	17.5	0.36	ENSG00000207968	MIR92A1	Up	1
Ac-H3K9	13	9.20E+07	9.20E+07	1108	31	94.77	9.8	0.78	ENSG00000207968	MIR92A1	Up	1
Ac-H3K9	13	9.20E+07	9.20E+07	1680	286	2213.6	36.8	4.88	ENSG00000207968	MIR92A1	Up	1
Ac-H3K9	1	1.74E+08	1.74E+08	1513	38	108.04	7.0	0.76	ENSG00000208310	SNORD75	Down	1
Ac-H3K9	1	1.74E+08	1.74E+08	716	23	119.88	17.2	0.73	ENSG00000208310	SNORD75	Up	1
Ac-H3K9	1	1.74E+08	1.74E+08	2862	311	2345.4	29.1	6.25	ENSG00000208310	SNORD75	Up	1
Ac-H3K9	1	1.74E+08	1.74E+08	1513	38	108.04	7.0	0.76	ENSG00000208313	SNORD77	Down	1
Ac-H3K9	1	1.74E+08	1.74E+08	716	23	119.88	17.2	0.73	ENSG00000208313	SNORD77	Up	1
Ac-H3K9	1	1.74E+08	1.74E+08	2862	311	2345.4	29.1	6.25	ENSG00000208313	SNORD77	Up	1
Ac-H3K9	1	1.74E+08	1.74E+08	1513	38	108.04	7.0	0.76	ENSG00000208317	SNORD78	Down	1
Ac-H3K9	1	1.74E+08	1.74E+08	716	23	119.88	17.2	0.73	ENSG00000208317	SNORD78	Up	1
Ac-H3K9	1	1.74E+08	1.74E+08	2862	311	2345.4	29.1	6.25	ENSG00000208317	SNORD78	Up	1
Ac-H3K9	5	1.81E+08	1.81E+08	5156	281	1658.6	28.6	2.15	ENSG00000208342	SNORD96A	Up	1
Ac-H3K9	5	1.81E+08	1.81E+08	3088	266	2358.1	57.4	6.67	ENSG00000208342	SNORD96A	Up	1
Ac-H3K9	17	7.49E+06	7.49E+06	1797	93	545.39	37.8	0.51	ENSG00000209582	SNORA48	Down	1
Ac-H3K9	17	7.48E+06	7.48E+06	1997	95	468.76	16.7	0.43	ENSG00000209582	SNORA48	Up	1
Ac-H3K9	19	1.02E+07	1.02E+07	1460	81	451.94	22.8	0.45	ENSG00000209645	SNORD105	Up	1
Ac-H3K9	5	4.08E+07	4.08E+07	901	56	337.07	23.7	0.45	ENSG00000212296	SNORD72	Up	1
Ac-H3K9	5	4.08E+07	4.08E+07	2084	156	953.66	23.0	0.58	ENSG00000212296	SNORD72	Up	1
Ac-H3K9	X	4.12E+07	4.12E+07	1463	91	761.77	45.6	0.43	ENSG00000215301	DDX3X	Intron	7
Ac-H3K9	X	4.12E+07	4.12E+07	1412	42	164.65	13.3	0.63	ENSG00000215301	DDX3X	Intron	7
Ac-H3K9	X	4.12E+07	4.12E+07	852	26	153.45	17.3	0.64	ENSG00000215301	DDX3X	Up	7
Ac-H3K9	13	9.20E+07	9.20E+07	1108	31	94.77	9.8	0.78	ENSG00000215417	MIR17HG	Exon	1
Ac-H3K9	13	9.20E+07	9.20E+07	1680	286	2213.6	36.8	4.88	ENSG00000215417	MIR17HG	Intron	1
Ac-H3K9	13	9.20E+07	9.20E+07	1257	30	89.04	6.5	0.77	ENSG00000215417	MIR17HG	Intron	1
Ac-H3K9	13	9.20E+07	9.20E+07	1574	81	398.62	17.5	0.36	ENSG00000215417	MIR17HG	Up	1
Ac-H3K9	17	8.05E+06	8.06E+06	791	20	120.81	18.7	0.72	ENSG00000220205	VAMP2	Intron	5
Ac-H3K9	17	8.08E+06	8.08E+06	355	25	196.14	26.1	0.51	ENSG00000220205	VAMP2	Up	5
Ac-H3K9	19	5.13E+07	5.13E+07	2108	174	1759.9	70.2	2.47	ENSG00000220988	SNORD88C	Up	1
Ac-H3K9	19	5.13E+07	5.13E+07	2108	174	1759.9	70.2	2.47	ENSG00000221241	SNORD88A	Up	1
Ac-H3K9	19	5.13E+07	5.13E+07	2108	174	1759.9	70.2	2.47	ENSG00000221381	SNORD88B	Up	1
Ac-H3K9	3	1.86E+08	1.87E+08	4178	296	2539.6	42.2	9.09	ENSG00000221420	SNORA81	Up	1
Ac-H3K9	17	6.22E+07	6.22E+07	919	25	107.58	11.6	0.75	ENSG00000221462	SNORA76	Up	1
Ac-H3K9	17	6.22E+07	6.22E+07	1648	106	627.31	21.9	0.51	ENSG00000221462	SNORA76	Up	1
Ac-H3K9	1	6.79E+07	6.79E+07	4024	289	1856.1	30.7	2.9	ENSG00000223263	U6.930	Down	1
Ac-H3K9	19	5.47E+07	5.47E+07	1149	28	113.31	11.0	0.75	ENSG00000223660	AC012314.20	Up	1
Ac-H3K9	19	5.47E+07	5.47E+07	1763	109	791.19	34.4	0.36	ENSG00000223660	AC012314.20	Up	1
Ac-H3K9	2	1.02E+08	1.02E+08	2273	152	1290.1	44.5	0.92	ENSG00000223947	AC016738.4	Up	1
Ac-H3K9	19	5.47E+07	5.47E+07	1149	28	113.31	11.0	0.75	ENSG00000224579	AC012314.19	Down	1

Ac-H3K9	19	5.47E+07	5.47E+07	1763	109	791.19	34.4	0.36	ENSG00000224579	AC012314.19	Down	1
Ac-H3K9	2	1.78E+08	1.78E+08	1555	152	956.7	19.0	0.59	ENSG00000229337	AC079305.8	Intron	1
Ac-H3K9	2	1.78E+08	1.78E+08	625	21	99.74	14.3	0.76	ENSG00000229337	AC079305.8	Intron	1
Ac-H3K9	2	1.78E+08	1.78E+08	529	19	115	17.0	0.75	ENSG00000229337	AC079305.8	Up	1
Ac-H3K9	2	1.78E+08	1.78E+08	1403	136	775.67	25.3	0.34	ENSG00000229337	AC079305.8	Up	1
Ac-H3K9	6	7.43E+07	7.43E+07	670	15	87.41	15.0	0.78	ENSG00000229862	RP11-505P4.7	Down	2
Ac-H3K9	6	7.42E+07	7.42E+07	962	37	265.44	25.3	0.52	ENSG00000229862	RP11-505P4.7	Intron	2
Ac-H3K9	6	7.42E+07	7.42E+07	2409	230	2086	46.3	3.92	ENSG00000229862	RP11-505P4.7	Up	2
Ac-H3K9	6	7.42E+07	7.42E+07	1334	38	198.42	13.5	0.52	ENSG00000229862	RP11-505P4.7	Up	2
Ac-H3K9	1	1.74E+08	1.74E+08	2862	311	2345.4	29.1	6.25	ENSG00000231792	RP5-1198E17.1	Down	1
Ac-H3K9	1	1.74E+08	1.74E+08	1513	38	108.04	7.0	0.76	ENSG00000231792	RP5-1198E17.1	Down	1
Ac-H3K9	1	1.74E+08	1.74E+08	716	23	119.88	17.2	0.73	ENSG00000231792	RP5-1198E17.1	Down	1
Ac-H3K9	17	7.48E+06	7.48E+06	1997	95	468.76	16.7	0.43	ENSG00000233223	AC113189.5	Down	2
Ac-H3K9	17	7.49E+06	7.49E+06	1797	93	545.39	37.8	0.51	ENSG00000233223	AC113189.5	Up	2
Ac-H3K9	5	1.81E+08	1.81E+08	5156	281	1658.6	28.6	2.15	ENSG00000233937	CTC-338M12.4	Exon	4
Ac-H3K9	5	1.81E+08	1.81E+08	648	18	83.14	11.6	0.79	ENSG00000233937	CTC-338M12.4	Intron	4
Ac-H3K9	5	1.81E+08	1.81E+08	1888	61	189.77	11.6	0.54	ENSG00000233937	CTC-338M12.4	Intron	4
Ac-H3K9	5	1.81E+08	1.81E+08	1091	55	392.6	28.5	0.39	ENSG00000233937	CTC-338M12.4	Intron	4
Ac-H3K9	5	1.81E+08	1.81E+08	3088	266	2358.1	57.4	6.67	ENSG00000233937	CTC-338M12.4	Up	4
Ac-H3K9	1	1.74E+08	1.74E+08	2862	311	2345.4	29.1	6.25	ENSG00000234741	GAS5	Exon	29
Ac-H3K9	1	1.74E+08	1.74E+08	1513	38	108.04	7.0	0.76	ENSG00000234741	GAS5	Exon	29
Ac-H3K9	1	1.74E+08	1.74E+08	716	23	119.88	17.2	0.73	ENSG00000234741	GAS5	Up	29
Ac-H3K9	9	1.94E+07	1.94E+07	3850	351	2729	50.6	18.2	ENSG00000234853	RP11-513M16.5	Down	1
Ac-H3K9	2	8.82E+06	8.82E+06	1016	30	167.44	27.6	0.61	ENSG00000235092	AC011747.7	Intron	7
Ac-H3K9	2	8.82E+06	8.82E+06	1081	41	174.36	13.2	0.55	ENSG00000235092	AC011747.7	Intron	7
Ac-H3K9	2	8.82E+06	8.82E+06	533	21	152.57	26.2	0.65	ENSG00000235092	AC011747.7	Intron	7
Ac-H3K9	2	8.82E+06	8.83E+06	622	21	133.73	16.9	0.68	ENSG00000235092	AC011747.7	Up	7
Ac-H3K9	2	8.82E+06	8.82E+06	2430	136	1090.3	34.8	0.84	ENSG00000235092	AC011747.7	Up	7
Ac-H3K9	9	8.66E+07	8.66E+07	4715	280	2126.3	55.6	4.17	ENSG00000235298	RP11-575L7.8	Down	1
Ac-H3K9	10	1.02E+08	1.02E+08	1282	116	934.3	57.3	0.54	ENSG00000235823	NCRNA00263	Exon	1
Ac-H3K9	10	1.02E+08	1.02E+08	836	24	100.67	10.6	0.76	ENSG00000235823	NCRNA00263	Up	1
Ac-H3K9	19	1.02E+07	1.02E+07	1102	54	439.39	31.8	0.43	ENSG00000238531	SNORD105B	Down	1
Ac-H3K9	19	1.02E+07	1.02E+07	1460	81	451.94	22.8	0.45	ENSG00000238531	SNORD105B	Up	1
Ac-H3K9	16	2.01E+06	2.02E+06	1300	61	415.46	28.5	0.39	ENSG00000238671	SNORA78	Down	1
Ac-H3K9	16	2.02E+06	2.02E+06	1430	51	349.89	44.9	0.44	ENSG00000238671	SNORA78	Down	1
Ac-H3K9	16	2.01E+06	2.01E+06	1486	70	416.63	25.5	0.39	ENSG00000238671	SNORA78	Up	1
Ac-H3K9	16	2.01E+06	2.01E+06	1124	32	92.25	8.4	0.77	ENSG00000238671	SNORA78	Up	1
Ac-H3K9	16	2.01E+06	2.01E+06	2609	200	982.64	18.4	0.62	ENSG00000238671	SNORA78	Up	1
Ac-H3K9	1	2.88E+07	2.88E+07	1651	90	492.75	24.5	0.47	ENSG00000238821	snoU13.263	Down	1
Ac-H3K9	1	2.88E+07	2.88E+07	6730	807	3100	40.7	16.7	ENSG00000238821	snoU13.263	Up	1
Ac-H3K9	17	7.49E+06	7.49E+06	1797	93	545.39	37.8	0.51	ENSG00000238917	SNORD10	Down	1
Ac-H3K9	17	7.48E+06	7.48E+06	1997	95	468.76	16.7	0.43	ENSG00000238917	SNORD10	Up	1
Ac-H3K9	3	1.86E+08	1.87E+08	4178	296	2539.6	42.2	9.09	ENSG00000238942	snR39B.2	Down	1
Ac-H3K9	13	9.20E+07	9.20E+07	1257	30	89.04	6.5	0.77	ENSG00000239594	RP11-282D2.3	Down	1
Ac-H3K9	13	9.20E+07	9.20E+07	1574	81	398.62	17.5	0.36	ENSG00000239594	RP11-282D2.3	Up	1
Ac-H3K9	13	9.20E+07	9.20E+07	1108	31	94.77	9.8	0.78	ENSG00000239594	RP11-282D2.3	Up	1
Ac-H3K9	13	9.20E+07	9.20E+07	1680	286	2213.6	36.8	4.88	ENSG00000239594	RP11-282D2.3	Up	1
Ac-H3K9	13	9.20E+07	9.20E+07	1257	30	89.04	6.5	0.77	ENSG00000240532	RP11-282D2.5	Down	1
Ac-H3K9	13	9.20E+07	9.20E+07	1574	81	398.62	17.5	0.36	ENSG00000240532	RP11-282D2.5	Up	1
Ac-H3K9	13	9.20E+07	9.20E+07	1108	31	94.77	9.8	0.78	ENSG00000240532	RP11-282D2.5	Up	1
Ac-H3K9	13	9.20E+07	9.20E+07	1680	286	2213.6	36.8	4.88	ENSG00000240532	RP11-282D2.5	Up	1
Ac-H3K9	22	2.01E+07	2.01E+07	1791	116	545.13	20.3	0.51	ENSG00000240816	AC006547.1	Down	1
Ac-H3K9	13	9.20E+07	9.20E+07	1257	30	89.04	6.5	0.77	ENSG00000242008	RP11-282D2.2	Down	1
Ac-H3K9	13	9.20E+07	9.20E+07	1574	81	398.62	17.5	0.36	ENSG00000242008	RP11-282D2.2	Up	1
Ac-H3K9	13	9.20E+07	9.20E+07	1108	31	94.77	9.8	0.78	ENSG00000242008	RP11-282D2.2	Up	1
Ac-H3K9	13	9.20E+07	9.20E+07	1680	286	2213.6	36.8	4.88	ENSG00000242008	RP11-282D2.2	Up	1
Ac-H3K9	1	2.88E+07	2.88E+07	1651	90	492.75	24.5	0.47	ENSG00000242125	SNHG3	Down	3
Ac-H3K9	1	2.88E+07	2.88E+07	6730	807	3100	40.7	16.7	ENSG00000242125	SNHG3	Up	3
Ac-H3K9	13	9.20E+07	9.20E+07	1257	30	89.04	6.5	0.77	ENSG00000243108	RP11-282D2.6	Down	1
Ac-H3K9	13	9.20E+07	9.20E+07	1574	81	398.62	17.5	0.36	ENSG00000243108	RP11-282D2.6	Up	1
Ac-H3K9	13	9.20E+07	9.20E+07	1108	31	94.77	9.8	0.78	ENSG00000243108	RP11-282D2.6	Up	1
Ac-H3K9	13	9.20E+07	9.20E+07	1680	286	2213.6	36.8	4.88	ENSG00000243108	RP11-282D2.6	Up	1
Ac-H3K9	19	1.02E+07	1.02E+07	1102	54	439.39	31.8	0.43	ENSG00000243207	PPAN-P2RY11	Down	2
Ac-H3K9	19	1.02E+07	1.02E+07	1460	81	451.94	22.8	0.45	ENSG00000243207	PPAN-P2RY11	Intron	2
Ac-H3K9	13	9.20E+07	9.20E+07	1257	30	89.04	6.5	0.77	ENSG00000243839	RP11-282D2.4	Down	1
Ac-H3K9	13	9.20E+07	9.20E+07	1574	81	398.62	17.5	0.36	ENSG00000243839	RP11-282D2.4	Up	1
Ac-H3K9	13	9.20E+07	9.20E+07	1108	31	94.77	9.8	0.78	ENSG00000243839	RP11-282D2.4	Up	1
Ac-H3K9	13	9.20E+07	9.20E+07	1680	286	2213.6	36.8	4.88	ENSG00000243839	RP11-282D2.4	Up	1

Ac-H3K9	20	3.43E+07	3.43E+07	668	19	113.42	14.5	0.75	ENSG00000244005	NFS1	Exon	19
Ac-H3K9	20	3.43E+07	3.43E+07	909	38	104.38	6.7	0.75	ENSG00000244005	NFS1	Intron	19
Ac-H3K9	20	3.43E+07	3.43E+07	1164	53	325.37	23.3	0.5	ENSG00000244005	NFS1	Intron	19
Ac-H3K9	20	3.43E+07	3.43E+07	1155	53	374.41	23.0	0.39	ENSG00000244005	NFS1	Up	19
Ac-H3K9	19	1.02E+07	1.02E+07	1102	54	439.39	31.8	0.43	ENSG00000244165	P2RY11	Down	2
Ac-H3K9	19	1.02E+07	1.02E+07	1460	81	451.94	22.8	0.45	ENSG00000244165	P2RY11	Up	2
Ac-H3K9	1	2.88E+07	2.88E+07	6730	807	3100	40.7	16.7	ENSG00000245178	AL513497.1	Down	1
Ac-H3K9	1	2.88E+07	2.88E+07	1651	90	492.75	24.5	0.47	ENSG00000245178	AL513497.1	Up	1
Ac-H3K9	11	6.52E+07	6.52E+07	1921	84	520.74	20.9	0.47	ENSG00000245532	NEAT1	Exon	2
Ac-H3K9	11	6.52E+07	6.52E+07	693	18	99.92	15.7	0.76	ENSG00000245532	NEAT1	Up	2
Ac-H3K9	11	6.52E+07	6.52E+07	976	23	92.97	8.6	0.78	ENSG00000245532	NEAT1	Up	2
Ac-H3K9	11	6.52E+07	6.52E+07	552	19	105.57	19.3	0.75	ENSG00000245532	NEAT1	Up	2
Ac-H3K9	15	6.08E+07	6.08E+07	600	16	97.48	15.3	0.78	ENSG00000245534	AC087385.1	Intron	1
Ac-H3K9	15	6.08E+07	6.08E+07	1425	64	442.21	30.7	0.43	ENSG00000245534	AC087385.1	Up	1
Ac-H3K9	11	8.28E+07	8.28E+07	1643	102	882.75	52.8	0.47	ENSG00000246067	RP11-113K21.5	Intron	8
Ac-H3K9	5	1.81E+08	1.81E+08	975	34	224.08	29.0	0.55	ENSG00000247049	CTC-338M12.7	Down	1
Ac-H3K9	5	1.81E+08	1.81E+08	647	17	101.52	15.3	0.76	ENSG00000247049	CTC-338M12.7	Down	1
Ac-H3K9	5	1.81E+08	1.81E+08	1238	33	179.27	14.9	0.55	ENSG00000247049	CTC-338M12.7	Down	1
Ac-H3K9	4	1.50E+07	1.50E+07	451	13	80.81	12.3	0.82	ENSG00000247624	AC006296.3	Up	2
Ac-H3K9	8	1.29E+08	1.29E+08	735	17	85.81	16.9	0.8	ENSG00000249375	RP11-1136L8.1	Up	3
Ac-H3K9	8	1.29E+08	1.29E+08	847	28	183.67	22.5	0.54	ENSG00000249375	RP11-1136L8.1	Up	3
Ac-H3K9	8	1.29E+08	1.29E+08	665	17	106.01	18.7	0.76	ENSG00000249375	RP11-1136L8.1	Up	3
Ac-H3K9	8	1.29E+08	1.29E+08	631	24	177.97	25.3	0.55	ENSG00000249375	RP11-1136L8.1	Up	3
Ac-H3K9	13	4.59E+07	4.59E+07	1159	36	204.49	18.4	0.53	ENSG00000253051	SNORA31.25	Up	1
Ac-H3K9	13	4.59E+07	4.59E+07	1978	114	955.13	50.3	0.58	ENSG00000253051	SNORA31.25	Up	1
Ac-H3K9	16	2.01E+06	2.01E+06	1124	32	92.25	8.4	0.77	ENSG00000255066	AC005363.7	Down	1
Ac-H3K9	16	2.01E+06	2.01E+06	1486	70	416.63	25.5	0.39	ENSG00000255066	AC005363.7	Down	1
Ac-H3K9	16	2.02E+06	2.02E+06	1430	51	349.89	44.9	0.44	ENSG00000255066	AC005363.7	Up	1
Ac-H3K9	16	2.01E+06	2.02E+06	1300	61	415.46	28.5	0.39	ENSG00000255066	AC005363.7	Up	1
Ac-H3K9	16	2.01E+06	2.01E+06	2609	200	982.64	18.4	0.62	ENSG00000255066	AC005363.7	Up	1
Ac-H3K9	13	4.59E+07	4.59E+07	1159	36	204.49	18.4	0.53	ENSG00000255137	RP11-290D2.4	Up	1
Ac-H3K9	13	4.59E+07	4.59E+07	1978	114	955.13	50.3	0.58	ENSG00000255137	RP11-290D2.4	Up	1
Ac-H3K9	16	2.01E+06	2.02E+06	1300	61	415.46	28.5	0.39	ENSG00000255198	SNHG9	Down	1
Ac-H3K9	16	2.02E+06	2.02E+06	1430	51	349.89	44.9	0.44	ENSG00000255198	SNHG9	Down	1
Ac-H3K9	16	2.01E+06	2.01E+06	1486	70	416.63	25.5	0.39	ENSG00000255198	SNHG9	Up	1
Ac-H3K9	16	2.01E+06	2.01E+06	1124	32	92.25	8.4	0.77	ENSG00000255198	SNHG9	Up	1
Ac-H3K9	16	2.01E+06	2.01E+06	2609	200	982.64	18.4	0.62	ENSG00000255198	SNHG9	Up	1
Ac-H3K9	16	2.01E+06	2.01E+06	1124	32	92.25	8.4	0.77	ENSG00000255278	AC005363.8	Down	1
Ac-H3K9	16	2.01E+06	2.01E+06	1486	70	416.63	25.5	0.39	ENSG00000255278	AC005363.8	Down	1
Ac-H3K9	16	2.02E+06	2.02E+06	1430	51	349.89	44.9	0.44	ENSG00000255278	AC005363.8	Up	1
Ac-H3K9	16	2.01E+06	2.02E+06	1300	61	415.46	28.5	0.39	ENSG00000255278	AC005363.8	Up	1
Ac-H3K9	16	2.01E+06	2.01E+06	2609	200	982.64	18.4	0.62	ENSG00000255278	AC005363.8	Up	1
Ac-H3K9	11	8.28E+07	8.28E+07	1643	102	882.75	52.8	0.47	ENSG00000255503	RP11-113K21.4	Down	1
Ac-H3K9	16	2.01E+06	2.01E+06	1124	32	92.25	8.4	0.77	ENSG00000255513	AC005363.9	Down	1
Ac-H3K9	16	2.01E+06	2.01E+06	1486	70	416.63	25.5	0.39	ENSG00000255513	AC005363.9	Down	1
Ac-H3K9	16	2.01E+06	2.01E+06	2609	200	982.64	18.4	0.62	ENSG00000255513	AC005363.9	Exon	1
Ac-H3K9	16	2.02E+06	2.02E+06	1430	51	349.89	44.9	0.44	ENSG00000255513	AC005363.9	Up	1
Ac-H3K9	16	2.01E+06	2.02E+06	1300	61	415.46	28.5	0.39	ENSG00000255513	AC005363.9	Up	1
Ac-H3K9	9	3.76E+07	3.76E+07	1371	59	417.71	34.8	0.39	ENSG00000255872	RP11-613M10.9	Intron	1
Ac-H3K9	9	3.79E+07	3.79E+07	1074	37	192.48	13.5	0.54	ENSG00000255872	RP11-613M10.9	Intron	1
Ac-H3K9	9	3.79E+07	3.79E+07	628	25	172.54	23.0	0.55	ENSG00000255872	RP11-613M10.9	Intron	1
Ac-H3K9	9	3.78E+07	3.78E+07	2278	78	501.43	29.8	0.49	ENSG00000255872	RP11-613M10.9	Intron	1
Ac-H3K9	9	3.78E+07	3.78E+07	1012	41	300.66	34.3	0.48	ENSG00000255872	RP11-613M10.9	Intron	1
Ac-H3K9	9	3.78E+07	3.78E+07	2705	124	478.86	13.0	0.45	ENSG00000255872	RP11-613M10.9	Intron	1
Ac-H3K9	9	3.78E+07	3.78E+07	673	29	230.27	33.7	0.51	ENSG00000255872	RP11-613M10.9	Intron	1
Ac-H3K9	8	1.29E+08	1.29E+08	735	17	85.81	16.9	0.8	ENSG00000256129	AC103819.1	Down	1
Ac-H3K9	8	1.29E+08	1.29E+08	847	28	183.67	22.5	0.54	ENSG00000256129	AC103819.1	Down	1
Ac-H3K9	8	1.29E+08	1.29E+08	665	17	106.01	18.7	0.76	ENSG00000256129	AC103819.1	Exon	1
Ac-H3K9	8	1.29E+08	1.29E+08	631	24	177.97	25.3	0.55	ENSG00000256129	AC103819.1	Up	1
Ac-H3K9	10	1.02E+08	1.02E+08	1282	116	934.3	57.3	0.54	ENSG00000256545	AL139819.1	Down	1
Ac-H3K9	10	1.02E+08	1.02E+08	836	24	100.67	10.6	0.76	ENSG00000256545	AL139819.1	Down	1
Ac-H3K9	1	2.88E+07	2.88E+07	6730	807	3100	40.7	16.7	ENSG00000256960	AL513497.2	Down	1
Ac-H3K9	1	2.88E+07	2.88E+07	1651	90	492.75	24.5	0.47	ENSG00000256960	AL513497.2	Intron	1
