# Supplementary figures and images for: EWS and FUS bind a subset of transcribed genes encoding proteins enriched in RNA regulatory functions
Source: BMC Genomics. 2015 Nov 14;16:929. doi: 10.1186/s12864-015-2125-9 (PMC4647676; doi:10.1186/s12864-015-2125-9)

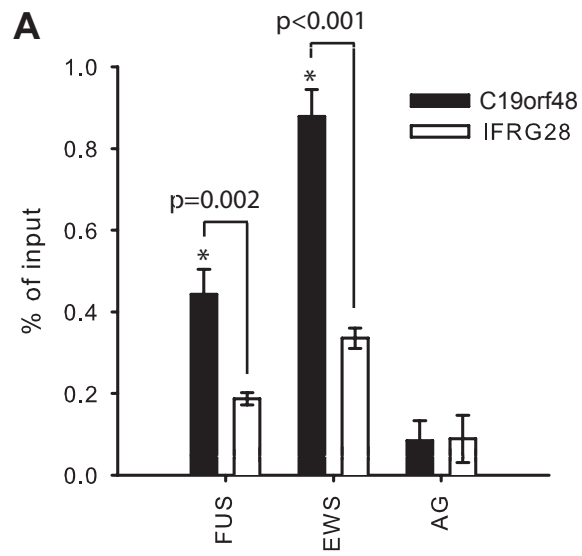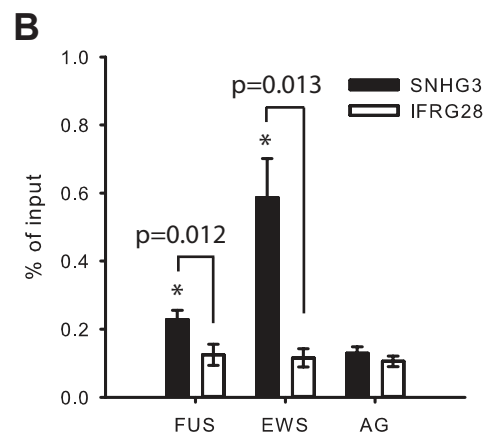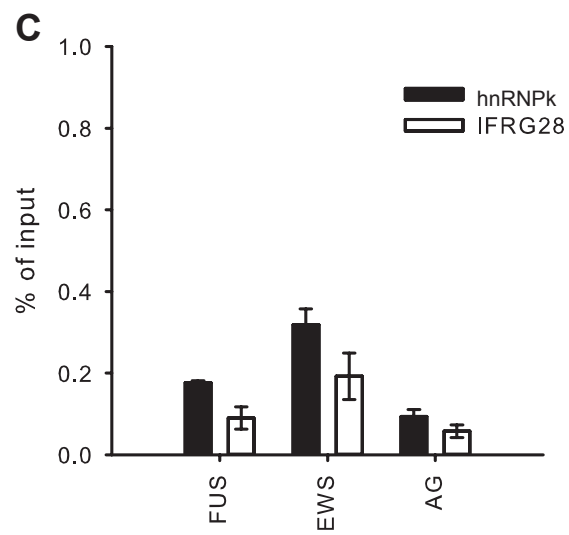

Supplement: Additional file 4: — Gene ontology (GO) and Kyoto encyclopedia of genes and genomes (KEGG) analysis by the web based software ChIP-Enrich of FUS and EWS ChIP-seq peaks [51]. (DOCX 19 kb) [file 12864_2015_2125_MOESM4_ESM.pdf]

**a**

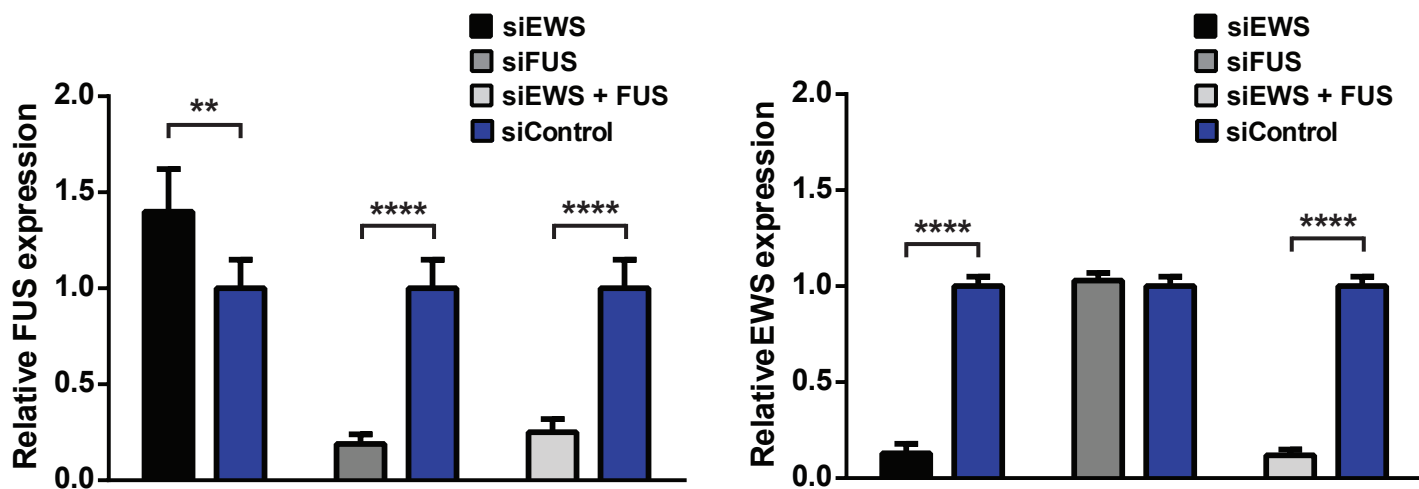

**b**

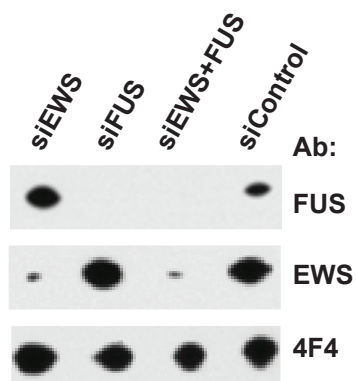

Supplement: Additional file 10: — Consequences of siRNA mediated depletion of FUS and EWS for gene expression. HEK-293 cells were double-transfected with specific siRNAs for FUS, EWS and FUS plus EWS, and as well as with a control siRNA (siControl). A. siRNA-depleted cells were used for relative mRNA quantification of FUS and EWS by qPCR. FUS and EWS expression was normalized to reference gene TBP. Experiments were performed in triplicates. Data presented as mean + SEM. Similar qPCR expression analyses showed a 1.5 to 2 fold increase in TAF15 mRNA by EWS and FUS depletion (not shown). B. Protein quantification by western blot of FUS and EWS from siRNA and control transfected cells. 4 F4 antibody recognizing HNRNP C1 + C2 was used as a loading control. (PDF 276 kb) [file 12864_2015_2125_MOESM10_ESM.pdf]

**A**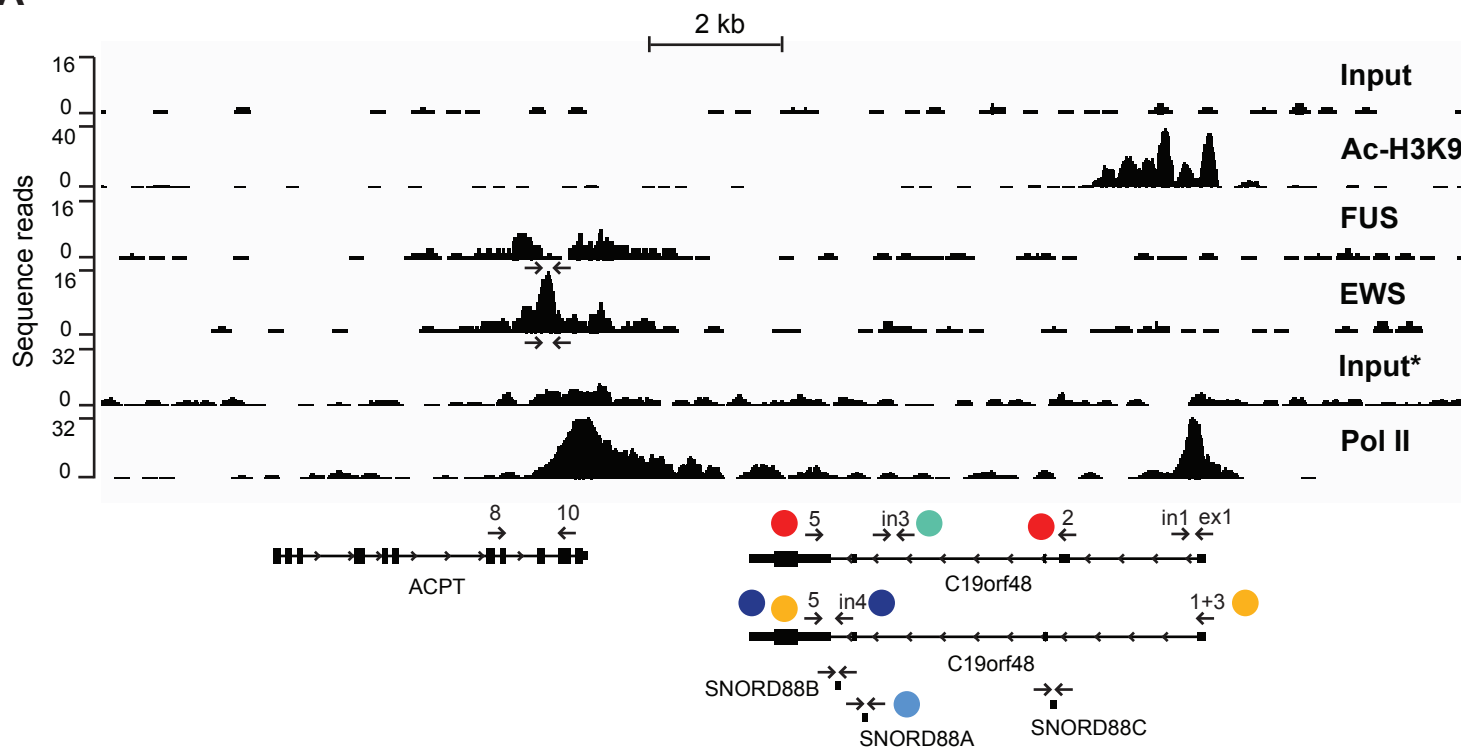**B**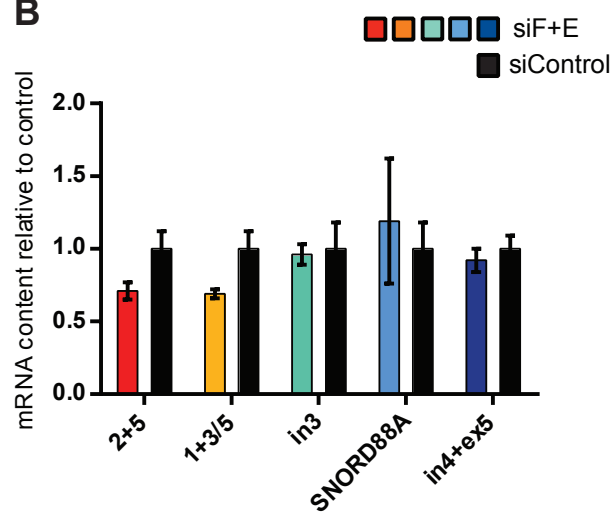**C**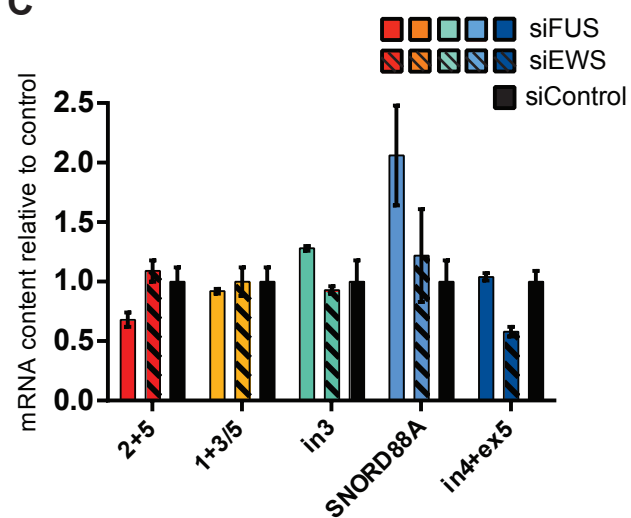

Supplement: Additional file 11: — Analysis of ChIP-seq FUS and EWS enrichment peaks in the ACPT and C19orf48 gene complex. A. Graphic distribution of ChIP-seq reads aligned to the ACPT and C19orf48 genes from the input, FUS, EWS and Ac-H3K9 ChIP-seq samples. The number of reads is shown on the scale to the left of each figure. The transcripts from the genes in the UCSC hg19 genomic database are shown in the bottom. The arrows beneath peaks illustrate location of the several qPCR amplicons used. The arrows above the transcripts illustrate location of RT-qPCR amplicons. Numbers above arrows denotes target exon numbers. B-C. FUS and EWS effect in ACPT and C19orf48 expression. The expression levels of C19orf48 transcripts were determined by qPCR from HEK-293 cells transfected with siRNA for FUS and EWS or control siRNA. Three independent experiments were performed and error bars indicate standard deviation. In B. simultaneous depletion of FUS and EWS was performed, whereas in C. FUS and EWS were individually depleted. (PDF 483 kb) [file 12864_2015_2125_MOESM11_ESM.pdf]

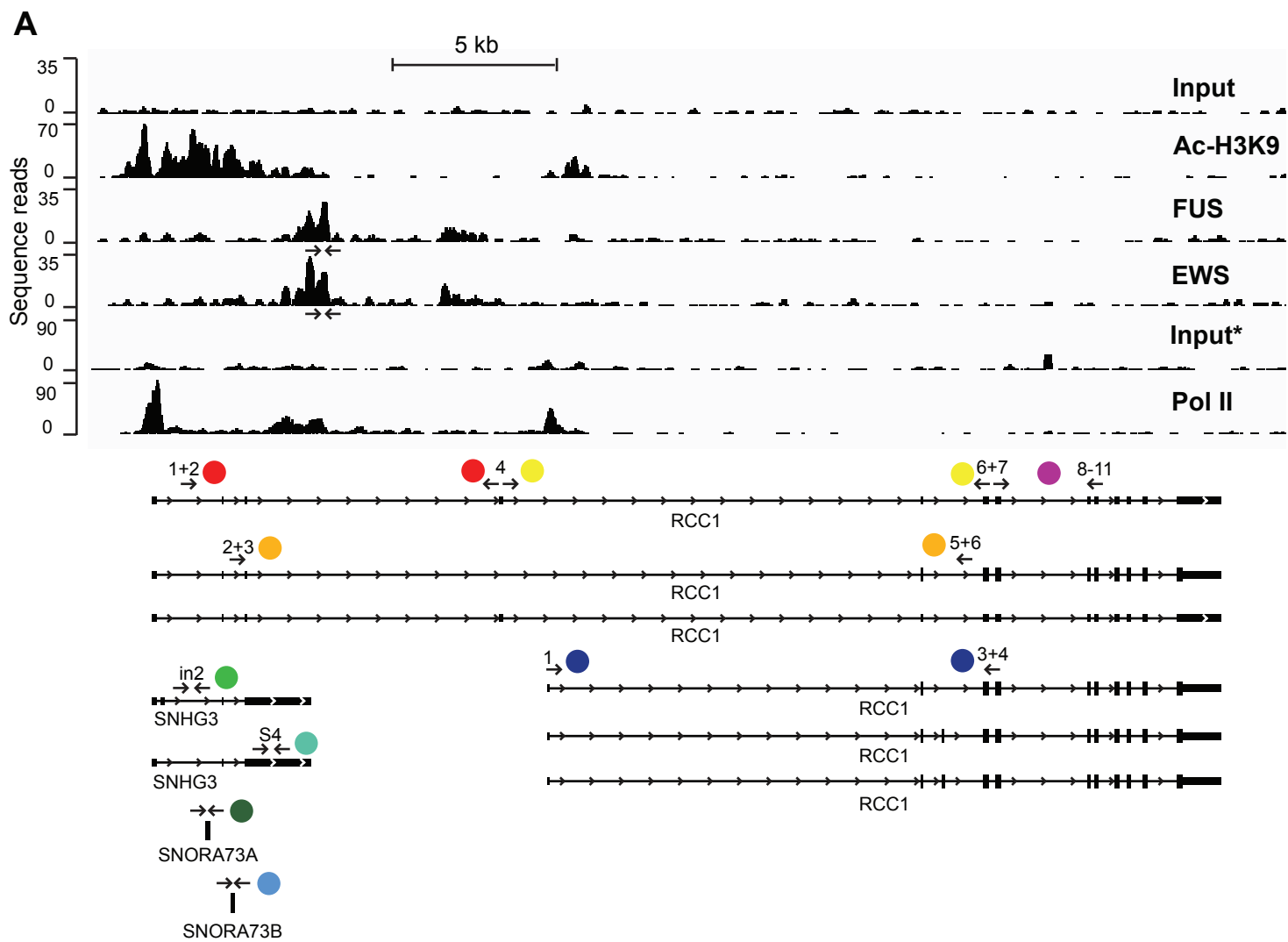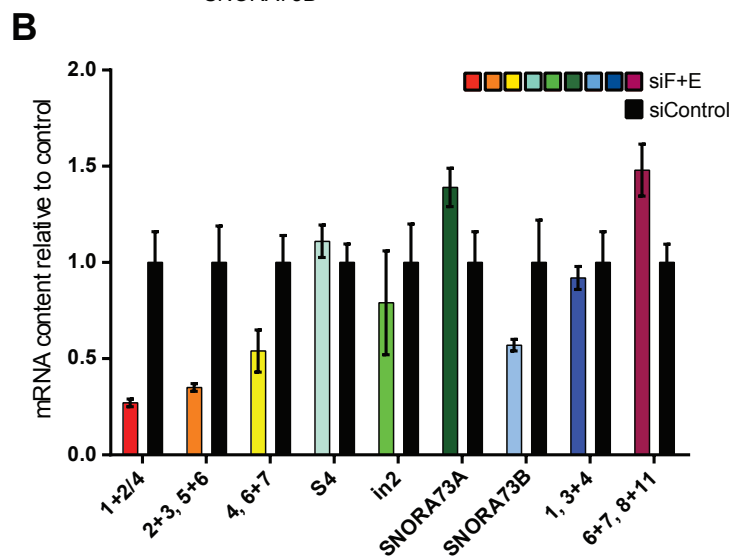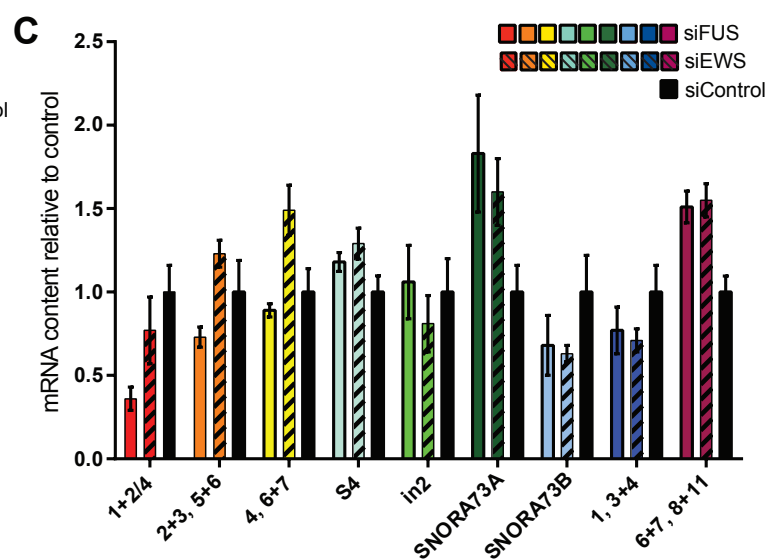

Supplement: Additional file 12: — Analysis of ChIP-seq FUS and EWS enrichment peaks in the RCC1 and SNHG3 gene complex, including SNORA73A and SNORA73B genes. A. Graphic distribution of ChIP-seq reads aligned to the RCC1 and SNHG3 genes from the input, FUS, EWS and Ac-H3K9 ChIP-seq samples. The number of reads is shown on the scale to the left of each figure. The transcripts from the genes in the UCSC hg19 genomic database are shown in the bottom. The arrows beneath peaks illustrate location of the several qPCR amplicons used. The arrows above the transcripts illustrate location of RT-qPCR amplicons. Numbers above arrows denotes target exon numbers. B-C. FUS and EWS effect for RCC1, SNHG3 and snoRNA expression. The expression levels were determined by qPCR from HEK-293 cells transfected with siRNA for FUS and EWS or control siRNA. Three independent experiments were performed and error bars indicate standard deviation. In B., simultaneous depletion of FUS and EWS was performed, whereas in C. FUS and EWS were individually depleted. (PDF 552 kb) [file 12864_2015_2125_MOESM12_ESM.pdf]

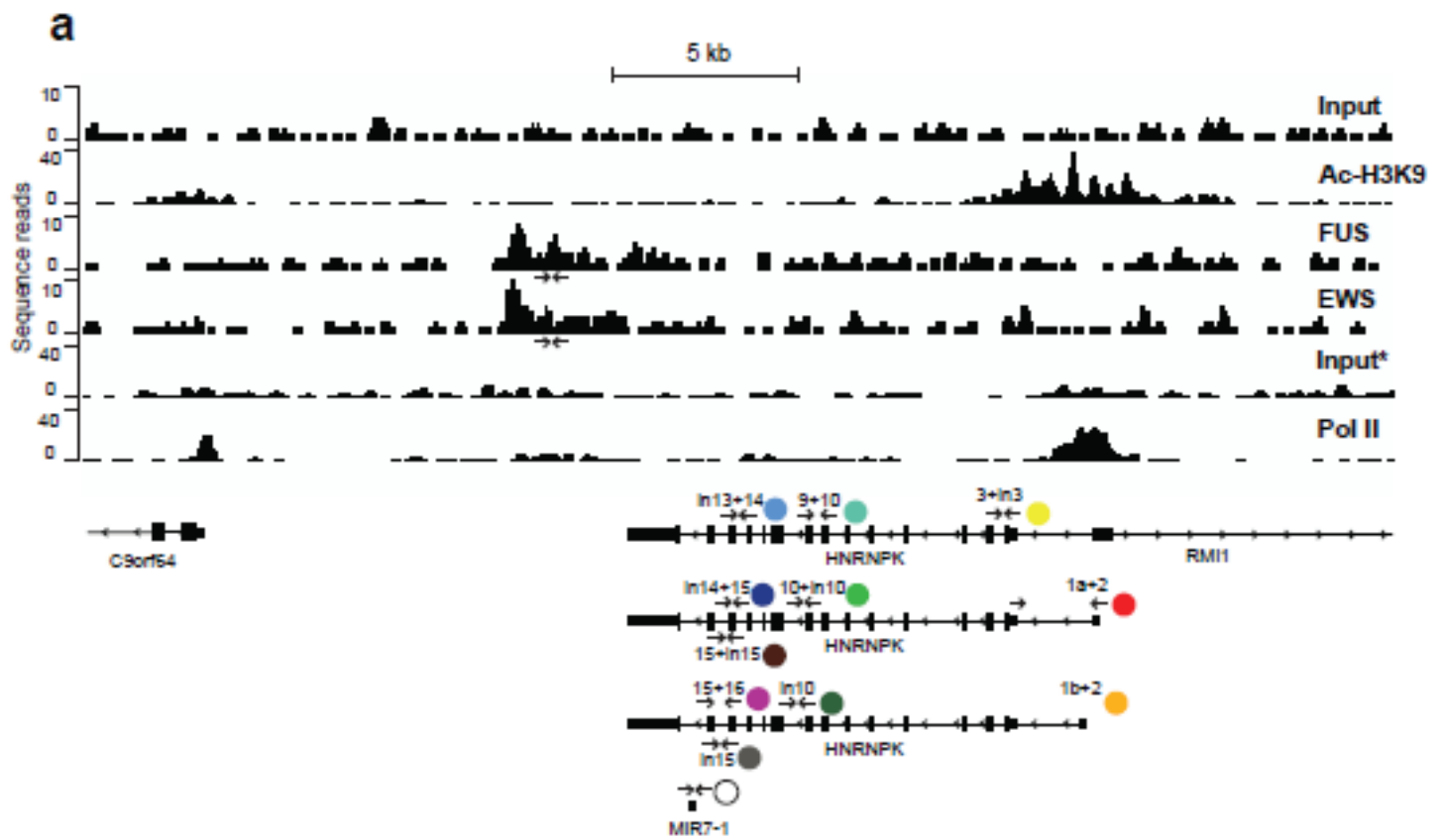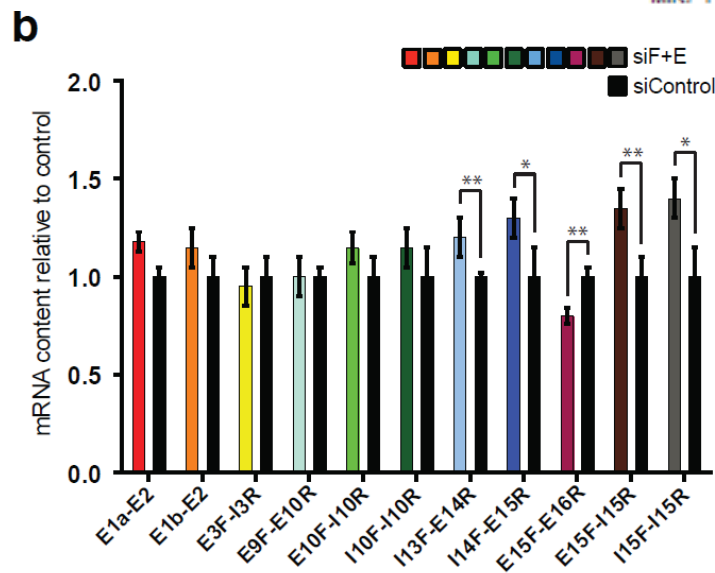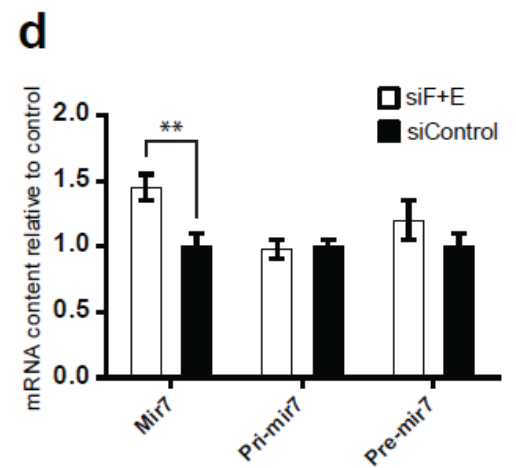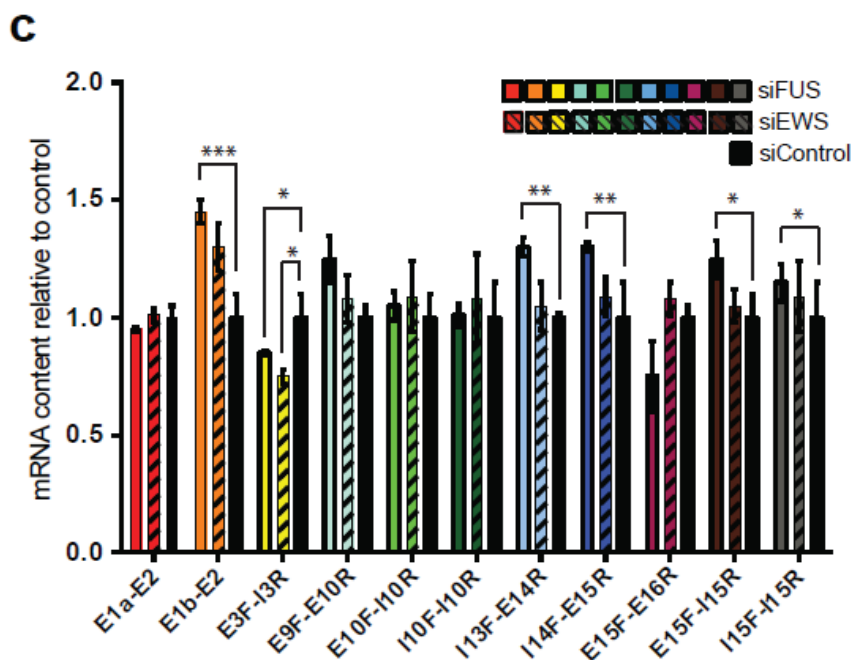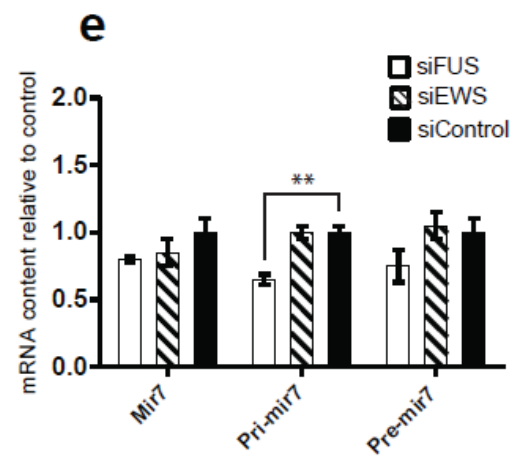

Supplement: Additional file 13: — FUS and EWS regulate RNA processing in the 3’-end of HNRNPK . A. Graphic distribution of ChIP-seq reads aligned to HNRNPK. The number of reads is shown on the scale to the left of each figure. The transcripts from the genes in the UCSC hg19 genomic database are shown in the bottom. The arrows beneath peaks illustrate location of qPCR amplicons. The arrows above the transcripts illustrate location of RT-qPCR amplicons. Numbers above arrows denote target exon numbers. B-C. The expression levels were determined by qPCR from HEK-293 cells transfected with siRNA for FUS and EWS or control siRNA. In B, simultaneous depletion of FUS and EWS was performed, whereas in C FUS and EWS were individually depleted. All values were normalized to reference gene TBP. All experiments were performed in triplicates. *P < 0.05; **P < 0.01. Data presented as mean + SEM. D-E. FUS and EWS involvement in the production of miR7-1 from HNRNPK intron 15. By RT-qPCR the relative amounts of mature miR7, premiR7-1 and pri-miR7-1 were determined by RT-qPCR from HEK-293 cells transfected with siRNA for FUS and EWS or control siRNA. In D., simultaneous depletion of FUS and EWS was performed, whereas in E. FUS and EWS were individually depleted Values were normalized to reference gene RNU48. **P < 0.01, ***P < 0.001, ****P < 0.0001. All experiments were performed in triplicates. Data presented as mean + SEM. (PDF 549 kb) [file 12864_2015_2125_MOESM13_ESM.pdf]
