# Supplementary material for: EWS and FUS bind a subset of transcribed genes encoding proteins enriched in RNA regulatory functions
Source: BMC Genomics. 2015 Nov 14;16:929. doi: 10.1186/s12864-015-2125-9 (PMC4647676; doi:10.1186/s12864-015-2125-9)
Supplement: Additional file 5: — qPCR validation of enrichment peaks identified by FUS and EWS ChIP-seq. The enrichment of DNA was quantified as percentage of the amount in the input sample and the p-values for enrichment of FUS and EWS were calculated. IFG28 was used as a negative control for enrichment. A control ChIP experiment was also performed with inclusion of pre-immune antiserum added to the AG beads used for chromatin purification (AG) instead of FUS and EWS antibodies. Data represents three independent experiments and standard deviation shown by error bars. A. C19orf48 and ACPT; B. RCC1 and SNHG3; C. HNRNPK. (PDF 23 kb) [file 12864_2015_2125_MOESM5_ESM.pdf]

Additional file 5. ChIP-Enrich analysis of FUS and EWS ChIP-seq peaks				
**GO and KEGG ID**	**GO and KEGG category**	**Gene Set Description**	**P value**	**FDR**
**FUS ChIP-seq gene set enrichment analysis (FDR value < 0.01)**				
GO:0006414	Gene Ontology Biological Process	translational elongation	1.27E-08	5.13E-05
GO:0030530	Gene Ontology Cellular Component	heterogeneous nuclear ribonucleoprotein complex	7.58E-08	3.74E-05
GO:2000602	Gene Ontology Biological Process	regulation of interphase of mitotic cell cycle	2.64E-07	5.34E-04
GO:0022626	Gene Ontology Cellular Component	cytosolic ribosome	1.56E-06	3.85E-04
GO:0006415	Gene Ontology Biological Process	translational termination	2.55E-06	0.0027
GO:0007346	Gene Ontology Biological Process	regulation of mitotic cell cycle	2.67E-06	0.0027
path:hsa03010	KEGG Pathway	Ribosome	5.79E-06	0.00108
GO:0006614	Gene Ontology Biological Process	SRP-dependent cotranslational protein targeting to membrane	6.51E-06	0.00404
GO:0045047	Gene Ontology Biological Process	protein targeting to ER	6.96E-06	0.00404
GO:0006613	Gene Ontology Biological Process	cotranslational protein targeting to membrane	6.99E-06	0.00404
GO:0043566	Gene Ontology Molecular Function	structure-specific DNA binding	8.88E-06	0.0077
GO:0070972	Gene Ontology Biological Process	protein localization to endoplasmic reticulum	1.52E-05	0.00727
GO:0000184	Gene Ontology Biological Process	nuclear-transcribed mRNA catabolic process, NMD	1.62E-05	0.00727
GO:0007050	Gene Ontology Biological Process	cell cycle arrest	1.84E-05	0.00743
GO:0030529	Gene Ontology Cellular Component	ribonucleoprotein complex	2.20E-05	0.00347
GO:0051329	Gene Ontology Biological Process	interphase of mitotic cell cycle	2.28E-05	0.00839
GO:0051325	Gene Ontology Biological Process	interphase	2.77E-05	0.00934
GO:0044391	Gene Ontology Cellular Component	ribosomal subunit	2.81E-05	0.00347
GO:0022625	Gene Ontology Cellular Component	cytosolic large ribosomal subunit	3.54E-05	0.00349
**EWS ChIP-seq gene set enrichment analysis (FDR value < 0.01)**				
GO:0006414	Gene Ontology Biological Process	translational elongation	7.86E-13	3.18E-09
GO:0006613	Gene Ontology Biological Process	cotranslational protein targeting to membrane	1.58E-12	3.19E-09
GO:0022626	Gene Ontology Cellular Component	cytosolic ribosome	1.92E-11	9.49E-09
GO:0006415	Gene Ontology Biological Process	translational termination	4.60E-11	6.20E-08
GO:0006614	Gene Ontology Biological Process	SRP-dependent cotranslational protein targeting to membrane	4.17E-10	3.91E-07
GO:0045047	Gene Ontology Biological Process	protein targeting to ER	4.83E-10	3.91E-07
GO:0006612	Gene Ontology Biological Process	protein targeting to membrane	1.10E-09	7.38E-07
GO:0000184	Gene Ontology Biological Process	nuclear-transcribed mRNA catabolic process, NMD	2.39E-09	1.24E-06
GO:0070972	Gene Ontology Biological Process	protein localization to endoplasmic reticulum	2.45E-09	1.24E-06
GO:0044391	Gene Ontology Cellular Component	ribosomal subunit	4.70E-09	8.13E-07
GO:0030530	Gene Ontology Cellular Component	heterogeneous nuclear ribonucleoprotein complex	4.95E-09	8.13E-07
GO:0003735	Gene Ontology Molecular Function	structural constituent of ribosome	5.34E-08	2.70E-05
GO:0019080	Gene Ontology Biological Process	viral genome expression	5.54E-08	2.49E-05
GO:0003723	Gene Ontology Molecular Function	RNA binding	6.21E-08	2.70E-05
GO:0006413	Gene Ontology Biological Process	translational initiation	8.75E-08	3.54E-05
GO:0030529	Gene Ontology Cellular Component	ribonucleoprotein complex	1.67E-07	2.06E-05
GO:0043624	Gene Ontology Biological Process	cellular protein complex disassembly	2.12E-07	7.78E-05
path:hsa03010	KEGG Pathway	Ribosome	2.32E-07	4.33E-05
GO:0005730	Gene Ontology Cellular Component	nucleolus	2.95E-07	2.69E-05
GO:0043241	Gene Ontology Biological Process	protein complex disassembly	3.02E-07	9.95E-05
GO:0003697	Gene Ontology Molecular Function	single-stranded DNA binding	3.13E-07	9.06E-05
GO:0000956	Gene Ontology Biological Process	nuclear-transcribed mRNA catabolic process	3.20E-07	9.95E-05
GO:0022627	Gene Ontology Cellular Component	cytosolic small ribosomal subunit	3.28E-07	2.69E-05
GO:0044445	Gene Ontology Cellular Component	cytosolic part	4.21E-07	2.97E-05
GO:0019058	Gene Ontology Biological Process	viral infectious cycle	4.53E-07	1.31E-04
GO:0006402	Gene Ontology Biological Process	mRNA catabolic process	6.12E-07	1.65E-04
GO:0005840	Gene Ontology Cellular Component	ribosome	7.38E-07	4.55E-05
GO:0034623	Gene Ontology Biological Process	cellular macromolecular complex disassembly	1.02E-06	2.59E-04
GO:0032984	Gene Ontology Biological Process	macromolecular complex disassembly	1.38E-06	3.28E-04
GO:0022415	Gene Ontology Biological Process	viral reproductive process	2.25E-06	5.06E-04
GO:0016071	Gene Ontology Biological Process	mRNA metabolic process	2.76E-06	5.87E-04
GO:0072594	Gene Ontology Biological Process	establishment of protein localization to organelle	3.17E-06	6.40E-04
GO:0006401	Gene Ontology Biological Process	RNA catabolic process	3.49E-06	6.72E-04
GO:0022625	Gene Ontology Cellular Component	cytosolic large ribosomal subunit	3.75E-06	2.05E-04
GO:0048610	Gene Ontology Biological Process	cellular process involved in reproduction	6.84E-06	0.00126
GO:0003727	Gene Ontology Molecular Function	single-stranded RNA binding	9.55E-06	0.00207
GO:0007162	Gene Ontology Biological Process	negative regulation of cell adhesion	1.14E-05	0.00198
GO:0071845	Gene Ontology Biological Process	cellular component disassembly at cellular level	1.18E-05	0.00198
GO:0022411	Gene Ontology Biological Process	cellular component disassembly	1.35E-05	0.00218
GO:0015935	Gene Ontology Cellular Component	small ribosomal subunit	1.67E-05	8.22E-04
GO:0043566	Gene Ontology Molecular Function	structure-specific DNA binding	2.21E-05	0.00365
GO:0008186	Gene Ontology Molecular Function	RNA-dependent ATPase activity	2.52E-05	0.00365
GO:0034110	Gene Ontology Biological Process	regulation of homotypic cell-cell adhesion	2.82E-05	0.00439
GO:0001958	Gene Ontology Biological Process	endochondral ossification	4.38E-05	0.00656
GO:0015934	Gene Ontology Cellular Component	large ribosomal subunit	5.92E-05	0.00265
GO:0016032	Gene Ontology Biological Process	viral reproduction	6.73E-05	0.00972
GO:0019843	Gene Ontology Molecular Function	rRNA binding	6.86E-05	0.00751
GO:0043394	Gene Ontology Molecular Function	proteoglycan binding	6.92E-05	0.00751

NMD, nonsense-mediated decay
