# Supplementary material for: EWS and FUS bind a subset of transcribed genes encoding proteins enriched in RNA regulatory functions
Source: BMC Genomics. 2015 Nov 14;16:929. doi: 10.1186/s12864-015-2125-9 (PMC4647676; doi:10.1186/s12864-015-2125-9)
Supplement: Additional file 6: — Cross-comparison of the genes identified to be associated with FUS, EWS or Ac-H3K9 by ChIP-seq analysis. The displayed overlap categories are FUS and EWS, FUS and Ac-H3K9, EWS and Ac-H3K9, FUS and EWS and Ac-H3K9, FUS and EWS. Chromosome (CH): the peak start and end in base pairs; Length: the peak length in base pairs; Tags: the number of sequences included in the peak; p-value: p-value on log2 scale; fold-change (fc): relative to input sample; FDR: false discovery rate in percent; ENS ID: gene ID in the ensemble database; gene location: location of the enrichment in the gene (upstream, downstream, intron, exon); and T: number of known transcript variants produced from the given gene. (DOCX 325 kb) [file 12864_2015_2125_MOESM6_ESM.docx]

| **Additional file 6.** Cross comparison of the identified genes in the FUS, EWS, and Ac-H3K9 ChIP samples. | | | | | | | | | | | | |
| --- | --- | --- | --- | --- | --- | --- | --- | --- | --- | --- | --- | --- |
| **FUS and EWS** | |  |  |  |  |  |  |  |  |  |  |  |
| **Sample** | **Ch.** | **peak start** | **peak end** | **length** | **tags** | **p-value** | **F.C** | **FDR** | **ENS ID** | **Gene name** | **location** | **T** |
| FUS | 5 | 1.77E+08 | 1.77E+08 | 661 | 15 | 84.47 | 12.1 | 100 | ENSG00000131188 | PRR7 | Up | 5 |
| EWS | 5 | 1.77E+08 | 1.77E+08 | 1116 | 32 | 209.84 | 20.0 | 100 | ENSG00000131188 | PRR7 | Up | 5 |
| EWS | 19 | 4.37E+07 | 4.37E+07 | 1604 | 95 | 508.57 | 13.0 | 100 | ENSG00000204941 | PSG5 | Up | 9 |
| EWS | 19 | 4.37E+07 | 4.37E+07 | 2371 | 126 | 519.09 | 13.8 | 100 | ENSG00000204941 | PSG5 | Up | 9 |
| FUS | 19 | 4.37E+07 | 4.37E+07 | 4008 | 167 | 605.17 | 11.8 | 100 | ENSG00000204941 | PSG5 | Up | 9 |
| FUS | 19 | 5.13E+07 | 5.13E+07 | 837 | 14 | 90.31 | 12.1 | 100 | ENSG00000221233 | AC010325.2 | Down | 1 |
| EWS | 19 | 5.13E+07 | 5.13E+07 | 1774 | 45 | 266.73 | 20.0 | 100 | ENSG00000221233 | AC010325.2 | Down | 1 |
| FUS | 19 | 5.13E+07 | 5.13E+07 | 1001 | 20 | 139.43 | 12.1 | 100 | ENSG00000221233 | AC010325.2 | Down | 1 |
| FUS | 3 | 8.63E+06 | 8.63E+06 | 1073 | 24 | 80.98 | 6.4 | 100 | ENSG00000224884 | AC034187.2 | Intron | 1 |
| EWS | 3 | 8.63E+06 | 8.63E+06 | 1479 | 47 | 209.56 | 11.4 | 100 | ENSG00000224884 | AC034187.2 | Intron | 1 |
| EWS | 2 | 1.78E+08 | 1.78E+08 | 3253 | 92 | 154.27 | 7.3 | 100 | ENSG00000225808 | AC079305.9 | Down | 1 |
| FUS | 2 | 1.78E+08 | 1.78E+08 | 1785 | 33 | 154.27 | 8.0 | 100 | ENSG00000225808 | AC079305.9 | Down | 1 |
| FUS | 3 | 8.63E+06 | 8.63E+06 | 1073 | 24 | 80.98 | 6.4 | 100 | ENSG00000227110 | AC087859.1 | Intron | 7 |
| EWS | 3 | 8.63E+06 | 8.63E+06 | 1479 | 47 | 209.56 | 11.4 | 100 | ENSG00000227110 | AC087859.1 | Intron | 7 |
| FUS | 19 | 4.37E+07 | 4.37E+07 | 4008 | 167 | 605.17 | 11.8 | 100 | ENSG00000243137 | PSG4 | Down | 6 |
| EWS | 19 | 4.37E+07 | 4.37E+07 | 2371 | 126 | 519.09 | 13.8 | 100 | ENSG00000243137 | PSG4 | Down | 6 |
| EWS | 19 | 4.37E+07 | 4.37E+07 | 1604 | 95 | 508.57 | 13.0 | 100 | ENSG00000243137 | PSG4 | Down | 6 |
| FUS | 5 | 1.77E+08 | 1.77E+08 | 661 | 15 | 84.47 | 12.1 | 100 | ENSG00000246334 | RP11-1334A24.4 | Intron | 4 |
| EWS | 5 | 1.77E+08 | 1.77E+08 | 1116 | 32 | 209.84 | 20.0 | 100 | ENSG00000246334 | RP11-1334A24.4 | Intron | 4 |
| FUS | 5 | 1.77E+08 | 1.77E+08 | 661 | 15 | 84.47 | 12.1 | 100 | ENSG00000248342 | AC145098.1 | Down | 1 |
| EWS | 5 | 1.77E+08 | 1.77E+08 | 1116 | 32 | 209.84 | 20.0 | 100 | ENSG00000248342 | AC145098.1 | Down | 1 |
|  |  |  |  |  |  |  |  |  |  |  |  |  |
| **FUS and Ac-H3K9** | | |  |  |  |  |  |  |  |  |  |  |
| **Sample** | **Ch.** | **peak start** | **peak end** | **length** | **tags** | **p-value** | **F.C** | **FDR** | **ENS ID** | **Gene name** | **location** | **T** |
| Ac-H3K9 | 2 | 1.02E+08 | 1.02E+08 | 2273 | 152 | 1290.1 | 44.5 | 0.92 | ENSG00000071082 | RPL31 | Exon | 11 |
| FUS | 2 | 1.02E+08 | 1.02E+08 | 1037 | 18 | 84.45 | 7.5 | 100 | ENSG00000071082 | RPL31 | Intron | 11 |
| FUS | 16 | 7.43E+07 | 7.43E+07 | 692 | 13 | 88.91 | 9.7 | 100 | ENSG00000103035 | PSMD7 | Exon | 2 |
| Ac-H3K9 | 16 | 7.43E+07 | 7.43E+07 | 968 | 28 | 163.79 | 14.9 | 0.64 | ENSG00000103035 | PSMD7 | Intron | 2 |
| Ac-H3K9 | 16 | 7.43E+07 | 7.43E+07 | 917 | 29 | 108.88 | 11.4 | 0.74 | ENSG00000103035 | PSMD7 | Up | 2 |
| Ac-H3K9 | 2 | 8.82E+06 | 8.83E+06 | 622 | 21 | 133.73 | 16.9 | 0.68 | ENSG00000115738 | ID2 | Down | 4 |
| FUS | 2 | 8.82E+06 | 8.82E+06 | 611 | 14 | 96.3 | 12.5 | 100 | ENSG00000115738 | ID2 | Exon | 4 |
| Ac-H3K9 | 2 | 8.82E+06 | 8.82E+06 | 2430 | 136 | 1090.3 | 34.8 | 0.84 | ENSG00000115738 | ID2 | Exon | 4 |
| Ac-H3K9 | 2 | 8.82E+06 | 8.82E+06 | 1081 | 41 | 174.36 | 13.2 | 0.55 | ENSG00000115738 | ID2 | Intron | 4 |
| Ac-H3K9 | 2 | 8.82E+06 | 8.82E+06 | 1016 | 30 | 167.44 | 27.6 | 0.61 | ENSG00000115738 | ID2 | Intron | 4 |
| Ac-H3K9 | 2 | 8.82E+06 | 8.82E+06 | 533 | 21 | 152.57 | 26.2 | 0.65 | ENSG00000115738 | ID2 | Up | 4 |
| FUS | 1 | 6.82E+07 | 6.82E+07 | 691 | 18 | 84.49 | 9.2 | 100 | ENSG00000116717 | GADD45A | Down | 4 |
| Ac-H3K9 | 1 | 6.82E+07 | 6.82E+07 | 2291 | 85 | 366.65 | 14.2 | 0.44 | ENSG00000116717 | GADD45A | Intron | 4 |
| Ac-H3K9 | 13 | 7.47E+07 | 7.47E+07 | 2837 | 157 | 705.51 | 20.5 | 0.56 | ENSG00000118922 | KLF12 | Intron | 4 |
| FUS | 13 | 7.44E+07 | 7.44E+07 | 1121 | 24 | 82.35 | 7.6 | 100 | ENSG00000118922 | KLF12 | Intron | 4 |
| Ac-H3K9 | 13 | 7.47E+07 | 7.47E+07 | 520 | 28 | 139.54 | 14.3 | 0.67 | ENSG00000118922 | KLF12 | Up | 4 |
| Ac-H3K9 | 13 | 7.47E+07 | 7.47E+07 | 1075 | 54 | 244.53 | 14.7 | 0.49 | ENSG00000118922 | KLF12 | Up | 4 |
| Ac-H3K9 | 13 | 7.47E+07 | 7.47E+07 | 519 | 21 | 86.04 | 10.7 | 0.79 | ENSG00000118922 | KLF12 | Up | 4 |
| Ac-H3K9 | 16 | 1.18E+07 | 1.18E+07 | 486 | 20 | 126.15 | 17.2 | 0.68 | ENSG00000122299 | ZC3H7A | Down | 2 |
| Ac-H3K9 | 16 | 1.18E+07 | 1.18E+07 | 1409 | 42 | 261.45 | 22.4 | 0.51 | ENSG00000122299 | ZC3H7A | Down | 2 |
| Ac-H3K9 | 16 | 1.19E+07 | 1.19E+07 | 519 | 17 | 123.42 | 29.9 | 0.7 | ENSG00000122299 | ZC3H7A | Intron | 2 |
| FUS | 16 | 1.19E+07 | 1.19E+07 | 302 | 9 | 87.29 | 11.3 | 100 | ENSG00000122299 | ZC3H7A | Intron | 2 |
| Ac-H3K9 | 16 | 1.19E+07 | 1.19E+07 | 725 | 27 | 134.25 | 12.3 | 0.69 | ENSG00000122299 | ZC3H7A | Up | 2 |
| Ac-H3K9 | 17 | 7.49E+06 | 7.49E+06 | 1797 | 93 | 545.39 | 37.8 | 0.51 | ENSG00000129194 | SOX15 | Down | 2 |
| FUS | 17 | 7.48E+06 | 7.48E+06 | 726 | 13 | 86 | 12.9 | 100 | ENSG00000129194 | SOX15 | Down | 2 |
| Ac-H3K9 | 17 | 7.49E+06 | 7.49E+06 | 1797 | 93 | 545.39 | 37.8 | 0.51 | ENSG00000129226 | CD68 | Down | 2 |
| Ac-H3K9 | 17 | 7.48E+06 | 7.48E+06 | 1997 | 95 | 468.76 | 16.7 | 0.43 | ENSG00000129226 | CD68 | Up | 2 |
| FUS | 17 | 7.48E+06 | 7.48E+06 | 726 | 13 | 86 | 12.9 | 100 | ENSG00000129226 | CD68 | Up | 2 |
| Ac-H3K9 | 17 | 7.49E+06 | 7.49E+06 | 1797 | 93 | 545.39 | 37.8 | 0.51 | ENSG00000129255 | MPDU1 | Intron | 5 |
| Ac-H3K9 | 17 | 7.48E+06 | 7.48E+06 | 1997 | 95 | 468.76 | 16.7 | 0.43 | ENSG00000129255 | MPDU1 | Up | 5 |
| FUS | 17 | 7.48E+06 | 7.48E+06 | 726 | 13 | 86 | 12.9 | 100 | ENSG00000129255 | MPDU1 | Up | 5 |
| Ac-H3K9 | 19 | 1.02E+07 | 1.02E+07 | 1102 | 54 | 439.39 | 31.8 | 0.43 | ENSG00000130810 | PPAN | Down | 10 |
| Ac-H3K9 | 19 | 1.02E+07 | 1.02E+07 | 1460 | 81 | 451.94 | 22.8 | 0.45 | ENSG00000130810 | PPAN | Exon | 10 |
| FUS | 19 | 1.02E+07 | 1.02E+07 | 1072 | 21 | 142.23 | 13.9 | 100 | ENSG00000130810 | PPAN | Intron | 10 |
| FUS | 19 | 1.02E+07 | 1.02E+07 | 1072 | 21 | 142.23 | 13.9 | 100 | ENSG00000130811 | EIF3G | Down | 1 |
| Ac-H3K9 | 19 | 1.02E+07 | 1.02E+07 | 1460 | 81 | 451.94 | 22.8 | 0.45 | ENSG00000130811 | EIF3G | Down | 1 |
| Ac-H3K9 | 19 | 1.02E+07 | 1.02E+07 | 1102 | 54 | 439.39 | 31.8 | 0.43 | ENSG00000130811 | EIF3G | Intron | 1 |
| FUS | 13 | 4.59E+07 | 4.59E+07 | 1393 | 32 | 106.51 | 9.3 | 100 | ENSG00000133112 | TPT1 | Down | 13 |
| Ac-H3K9 | 13 | 4.59E+07 | 4.59E+07 | 1978 | 114 | 955.13 | 50.3 | 0.58 | ENSG00000133112 | TPT1 | Exon | 13 |
| Ac-H3K9 | 13 | 4.59E+07 | 4.59E+07 | 1159 | 36 | 204.49 | 18.4 | 0.53 | ENSG00000133112 | TPT1 | Up | 13 |
| Ac-H3K9 | 13 | 7.61E+07 | 7.61E+07 | 2964 | 129 | 465.94 | 14.3 | 0.43 | ENSG00000136111 | TBC1D4 | Intron | 8 |
| FUS | 13 | 7.59E+07 | 7.59E+07 | 1139 | 27 | 85.31 | 6.9 | 100 | ENSG00000136111 | TBC1D4 | Intron | 8 |
| Ac-H3K9 | 13 | 7.61E+07 | 7.61E+07 | 895 | 41 | 103.27 | 12.3 | 0.76 | ENSG00000136111 | TBC1D4 | Up | 8 |
| Ac-H3K9 | 11 | 7.29E+07 | 7.29E+07 | 2458 | 88 | 301.76 | 15.3 | 0.48 | ENSG00000137478 | FCHSD2 | Intron | 10 |
| FUS | 11 | 7.26E+07 | 7.26E+07 | 520 | 13 | 85.01 | 12.5 | 100 | ENSG00000137478 | FCHSD2 | Intron | 10 |
| Ac-H3K9 | 5 | 4.08E+07 | 4.08E+07 | 2084 | 156 | 953.66 | 23.0 | 0.58 | ENSG00000145592 | RPL37 | Exon | 6 |
| FUS | 5 | 4.08E+07 | 4.08E+07 | 1760 | 34 | 108.99 | 8.0 | 100 | ENSG00000145592 | RPL37 | Exon | 6 |
| Ac-H3K9 | 5 | 4.08E+07 | 4.08E+07 | 901 | 56 | 337.07 | 23.7 | 0.45 | ENSG00000145592 | RPL37 | Up | 6 |
| Ac-H3K9 | 17 | 7.48E+06 | 7.48E+06 | 1997 | 95 | 468.76 | 16.7 | 0.43 | ENSG00000161956 | SENP3 | Down | 2 |
| FUS | 17 | 7.48E+06 | 7.48E+06 | 726 | 13 | 86 | 12.9 | 100 | ENSG00000161956 | SENP3 | Down | 2 |
| Ac-H3K9 | 17 | 7.46E+06 | 7.47E+06 | 2372 | 100 | 663.9 | 35.3 | 0.57 | ENSG00000161956 | SENP3 | Up | 2 |
| FUS | 17 | 7.48E+06 | 7.48E+06 | 726 | 13 | 86 | 12.9 | 100 | ENSG00000161960 | EIF4A1 | Down | 3 |
| Ac-H3K9 | 17 | 7.49E+06 | 7.49E+06 | 1797 | 93 | 545.39 | 37.8 | 0.51 | ENSG00000161960 | EIF4A1 | Down | 3 |
| Ac-H3K9 | 17 | 7.48E+06 | 7.48E+06 | 1997 | 95 | 468.76 | 16.7 | 0.43 | ENSG00000161960 | EIF4A1 | Intron | 3 |
| Ac-H3K9 | 17 | 7.46E+06 | 7.47E+06 | 2372 | 100 | 663.9 | 35.3 | 0.57 | ENSG00000161960 | EIF4A1 | Up | 3 |
| FUS | 1 | 7.84E+07 | 7.84E+07 | 1856 | 41 | 107.89 | 6.1 | 100 | ENSG00000162613 | FUBP1 | Down | 16 |
| Ac-H3K9 | 1 | 7.84E+07 | 7.84E+07 | 5392 | 469 | 2616.8 | 36.0 | 11.1 | ENSG00000162613 | FUBP1 | Intron | 16 |
| Ac-H3K9 | 1 | 7.84E+07 | 7.84E+07 | 1295 | 46 | 166.55 | 15.1 | 0.62 | ENSG00000162614 | NEXN | Intron | 10 |
| FUS | 1 | 7.84E+07 | 7.84E+07 | 1856 | 41 | 107.89 | 6.1 | 100 | ENSG00000162614 | NEXN | Intron | 10 |
| FUS | 14 | 3.49E+07 | 3.49E+07 | 1015 | 18 | 86.05 | 7.5 | 100 | ENSG00000165389 | C14orf147 | Intron | 1 |
| Ac-H3K9 | 14 | 3.49E+07 | 3.49E+07 | 838 | 20 | 83.71 | 7.4 | 0.79 | ENSG00000165389 | C14orf147 | Intron | 1 |
| Ac-H3K9 | 14 | 3.49E+07 | 3.49E+07 | 939 | 41 | 186.17 | 11.5 | 0.53 | ENSG00000165389 | C14orf147 | Intron | 1 |
| Ac-H3K9 | 14 | 3.49E+07 | 3.49E+07 | 560 | 14 | 88.66 | 14.9 | 0.76 | ENSG00000165389 | C14orf147 | Up | 1 |
| Ac-H3K9 | 13 | 4.59E+07 | 4.59E+07 | 1159 | 36 | 204.49 | 18.4 | 0.53 | ENSG00000170919 | XXyac-R12DG2.2 | Intron | 21 |
| FUS | 13 | 4.59E+07 | 4.59E+07 | 1393 | 32 | 106.51 | 9.3 | 100 | ENSG00000170919 | XXyac-R12DG2.2 | Up | 21 |
| Ac-H3K9 | 13 | 4.59E+07 | 4.59E+07 | 1978 | 114 | 955.13 | 50.3 | 0.58 | ENSG00000170919 | XXyac-R12DG2.2 | Up | 21 |
| FUS | 22 | 4.30E+07 | 4.30E+07 | 562 | 12 | 84.45 | 15.0 | 100 | ENSG00000182841 | RP1-222E13.10 | Intron | 4 |
| Ac-H3K9 | 22 | 4.30E+07 | 4.30E+07 | 1371 | 81 | 625.83 | 39.3 | 0.51 | ENSG00000182841 | RP1-222E13.10 | Intron | 4 |
| Ac-H3K9 | 22 | 4.30E+07 | 4.30E+07 | 1371 | 81 | 625.83 | 39.3 | 0.51 | ENSG00000183569 | SERHL2 | Down | 11 |
| FUS | 22 | 4.30E+07 | 4.30E+07 | 562 | 12 | 84.45 | 15.0 | 100 | ENSG00000183569 | SERHL2 | Intron | 11 |
| FUS | 13 | 4.59E+07 | 4.59E+07 | 1393 | 32 | 106.51 | 9.3 | 100 | ENSG00000199477 | SNORA31 | Down | 1 |
| Ac-H3K9 | 13 | 4.59E+07 | 4.59E+07 | 1159 | 36 | 204.49 | 18.4 | 0.53 | ENSG00000199477 | SNORA31 | Up | 1 |
| Ac-H3K9 | 13 | 4.59E+07 | 4.59E+07 | 1978 | 114 | 955.13 | 50.3 | 0.58 | ENSG00000199477 | SNORA31 | Up | 1 |
| FUS | 17 | 1.90E+07 | 1.90E+07 | 439 | 13 | 113.36 | 15.2 | 100 | ENSG00000200229 | SNORD3B-1 | Down | 1 |
| Ac-H3K9 | 17 | 1.90E+07 | 1.90E+07 | 1007 | 51 | 383.03 | 49.4 | 0.41 | ENSG00000200229 | SNORD3B-1 | Down | 1 |
| Ac-H3K9 | 17 | 1.90E+07 | 1.90E+07 | 878 | 26 | 107.51 | 13.3 | 0.75 | ENSG00000200229 | SNORD3B-1 | Down | 1 |
| Ac-H3K9 | 17 | 1.90E+07 | 1.90E+07 | 663 | 23 | 165.95 | 33.7 | 0.63 | ENSG00000200229 | SNORD3B-1 | Down | 1 |
| Ac-H3K9 | 17 | 1.90E+07 | 1.90E+07 | 724 | 50 | 371.88 | 36.6 | 0.42 | ENSG00000200229 | SNORD3B-1 | Up | 1 |
| Ac-H3K9 | 17 | 1.90E+07 | 1.90E+07 | 878 | 26 | 107.51 | 13.3 | 0.75 | ENSG00000201750 | SNORD3B-2 | Down | 1 |
| Ac-H3K9 | 17 | 1.90E+07 | 1.90E+07 | 724 | 50 | 371.88 | 36.6 | 0.42 | ENSG00000201750 | SNORD3B-2 | Down | 1 |
| FUS | 17 | 1.90E+07 | 1.90E+07 | 439 | 13 | 113.36 | 15.2 | 100 | ENSG00000201750 | SNORD3B-2 | Exon | 1 |
| Ac-H3K9 | 17 | 1.90E+07 | 1.90E+07 | 1007 | 51 | 383.03 | 49.4 | 0.41 | ENSG00000201750 | SNORD3B-2 | Up | 1 |
| Ac-H3K9 | 17 | 1.90E+07 | 1.90E+07 | 663 | 23 | 165.95 | 33.7 | 0.63 | ENSG00000201750 | SNORD3B-2 | Up | 1 |
| Ac-H3K9 | 2 | 1.02E+08 | 1.02E+08 | 2273 | 152 | 1290.1 | 44.5 | 0.92 | ENSG00000204634 | TBC1D8 | Down | 10 |
| FUS | 2 | 1.02E+08 | 1.02E+08 | 1037 | 18 | 84.45 | 7.5 | 100 | ENSG00000204634 | TBC1D8 | Exon | 10 |
| Ac-H3K9 | 2 | 1.02E+08 | 1.02E+08 | 545 | 23 | 128.03 | 11.3 | 0.68 | ENSG00000204634 | TBC1D8 | Intron | 10 |
| Ac-H3K9 | 2 | 1.02E+08 | 1.02E+08 | 1142 | 36 | 168.79 | 11.7 | 0.6 | ENSG00000204634 | TBC1D8 | Up | 10 |
| FUS | 17 | 7.48E+06 | 7.48E+06 | 726 | 13 | 86 | 12.9 | 100 | ENSG00000207152 | SNORA67 | Down | 1 |
| Ac-H3K9 | 17 | 7.49E+06 | 7.49E+06 | 1797 | 93 | 545.39 | 37.8 | 0.51 | ENSG00000207152 | SNORA67 | Down | 1 |
| Ac-H3K9 | 17 | 7.48E+06 | 7.48E+06 | 1997 | 95 | 468.76 | 16.7 | 0.43 | ENSG00000207152 | SNORA67 | Up | 1 |
| FUS | 17 | 7.48E+06 | 7.48E+06 | 726 | 13 | 86 | 12.9 | 100 | ENSG00000209582 | SNORA48 | Down | 1 |
| Ac-H3K9 | 17 | 7.49E+06 | 7.49E+06 | 1797 | 93 | 545.39 | 37.8 | 0.51 | ENSG00000209582 | SNORA48 | Down | 1 |
| Ac-H3K9 | 17 | 7.48E+06 | 7.48E+06 | 1997 | 95 | 468.76 | 16.7 | 0.43 | ENSG00000209582 | SNORA48 | Up | 1 |
| FUS | 19 | 1.02E+07 | 1.02E+07 | 1072 | 21 | 142.23 | 13.9 | 100 | ENSG00000209645 | SNORD105 | Down | 1 |
| Ac-H3K9 | 19 | 1.02E+07 | 1.02E+07 | 1460 | 81 | 451.94 | 22.8 | 0.45 | ENSG00000209645 | SNORD105 | Up | 1 |
| FUS | 5 | 4.08E+07 | 4.08E+07 | 1760 | 34 | 108.99 | 8.0 | 100 | ENSG00000212296 | SNORD72 | Down | 1 |
| Ac-H3K9 | 5 | 4.08E+07 | 4.08E+07 | 901 | 56 | 337.07 | 23.7 | 0.45 | ENSG00000212296 | SNORD72 | Up | 1 |
| Ac-H3K9 | 5 | 4.08E+07 | 4.08E+07 | 2084 | 156 | 953.66 | 23.0 | 0.58 | ENSG00000212296 | SNORD72 | Up | 1 |
| Ac-H3K9 | 2 | 1.02E+08 | 1.02E+08 | 2273 | 152 | 1290.1 | 44.5 | 0.92 | ENSG00000223947 | AC016738.4 | Up | 1 |
| FUS | 2 | 1.02E+08 | 1.02E+08 | 1037 | 18 | 84.45 | 7.5 | 100 | ENSG00000223947 | AC016738.4 | Up | 1 |
| FUS | 17 | 7.48E+06 | 7.48E+06 | 726 | 13 | 86 | 12.9 | 100 | ENSG00000233223 | AC113189.5 | Down | 2 |
| Ac-H3K9 | 17 | 7.48E+06 | 7.48E+06 | 1997 | 95 | 468.76 | 16.7 | 0.43 | ENSG00000233223 | AC113189.5 | Down | 2 |
| Ac-H3K9 | 17 | 7.49E+06 | 7.49E+06 | 1797 | 93 | 545.39 | 37.8 | 0.51 | ENSG00000233223 | AC113189.5 | Up | 2 |
| Ac-H3K9 | 2 | 8.82E+06 | 8.82E+06 | 1016 | 30 | 167.44 | 27.6 | 0.61 | ENSG00000235092 | AC011747.7 | Intron | 7 |
| Ac-H3K9 | 2 | 8.82E+06 | 8.82E+06 | 1081 | 41 | 174.36 | 13.2 | 0.55 | ENSG00000235092 | AC011747.7 | Intron | 7 |
| Ac-H3K9 | 2 | 8.82E+06 | 8.82E+06 | 533 | 21 | 152.57 | 26.2 | 0.65 | ENSG00000235092 | AC011747.7 | Intron | 7 |
| Ac-H3K9 | 2 | 8.82E+06 | 8.83E+06 | 622 | 21 | 133.73 | 16.9 | 0.68 | ENSG00000235092 | AC011747.7 | Up | 7 |
| FUS | 2 | 8.82E+06 | 8.82E+06 | 611 | 14 | 96.3 | 12.5 | 100 | ENSG00000235092 | AC011747.7 | Up | 7 |
| Ac-H3K9 | 2 | 8.82E+06 | 8.82E+06 | 2430 | 136 | 1090.3 | 34.8 | 0.84 | ENSG00000235092 | AC011747.7 | Up | 7 |
| FUS | 19 | 1.02E+07 | 1.02E+07 | 1072 | 21 | 142.23 | 13.9 | 100 | ENSG00000238531 | SNORD105B | Down | 1 |
| Ac-H3K9 | 19 | 1.02E+07 | 1.02E+07 | 1102 | 54 | 439.39 | 31.8 | 0.43 | ENSG00000238531 | SNORD105B | Down | 1 |
| Ac-H3K9 | 19 | 1.02E+07 | 1.02E+07 | 1460 | 81 | 451.94 | 22.8 | 0.45 | ENSG00000238531 | SNORD105B | Up | 1 |
| FUS | 17 | 7.48E+06 | 7.48E+06 | 726 | 13 | 86 | 12.9 | 100 | ENSG00000238917 | SNORD10 | Down | 1 |
| Ac-H3K9 | 17 | 7.49E+06 | 7.49E+06 | 1797 | 93 | 545.39 | 37.8 | 0.51 | ENSG00000238917 | SNORD10 | Down | 1 |
| Ac-H3K9 | 17 | 7.48E+06 | 7.48E+06 | 1997 | 95 | 468.76 | 16.7 | 0.43 | ENSG00000238917 | SNORD10 | Up | 1 |
| Ac-H3K9 | 19 | 1.02E+07 | 1.02E+07 | 1102 | 54 | 439.39 | 31.8 | 0.43 | ENSG00000243207 | PPAN-P2RY11 | Down | 2 |
| Ac-H3K9 | 19 | 1.02E+07 | 1.02E+07 | 1460 | 81 | 451.94 | 22.8 | 0.45 | ENSG00000243207 | PPAN-P2RY11 | Intron | 2 |
| FUS | 19 | 1.02E+07 | 1.02E+07 | 1072 | 21 | 142.23 | 13.9 | 100 | ENSG00000243207 | PPAN-P2RY11 | Intron | 2 |
| Ac-H3K9 | 19 | 1.02E+07 | 1.02E+07 | 1102 | 54 | 439.39 | 31.8 | 0.43 | ENSG00000244165 | P2RY11 | Down | 2 |
| FUS | 19 | 1.02E+07 | 1.02E+07 | 1072 | 21 | 142.23 | 13.9 | 100 | ENSG00000244165 | P2RY11 | Intron | 2 |
| Ac-H3K9 | 19 | 1.02E+07 | 1.02E+07 | 1460 | 81 | 451.94 | 22.8 | 0.45 | ENSG00000244165 | P2RY11 | Up | 2 |
| FUS | 13 | 4.59E+07 | 4.59E+07 | 1393 | 32 | 106.51 | 9.3 | 100 | ENSG00000253051 | SNORA31.25 | Down | 1 |
| Ac-H3K9 | 13 | 4.59E+07 | 4.59E+07 | 1159 | 36 | 204.49 | 18.4 | 0.53 | ENSG00000253051 | SNORA31.25 | Up | 1 |
| Ac-H3K9 | 13 | 4.59E+07 | 4.59E+07 | 1978 | 114 | 955.13 | 50.3 | 0.58 | ENSG00000253051 | SNORA31.25 | Up | 1 |
| FUS | 13 | 4.59E+07 | 4.59E+07 | 1393 | 32 | 106.51 | 9.3 | 100 | ENSG00000255137 | RP11-290D2.4 | Down | 1 |
| Ac-H3K9 | 13 | 4.59E+07 | 4.59E+07 | 1159 | 36 | 204.49 | 18.4 | 0.53 | ENSG00000255137 | RP11-290D2.4 | Up | 1 |
| Ac-H3K9 | 13 | 4.59E+07 | 4.59E+07 | 1978 | 114 | 955.13 | 50.3 | 0.58 | ENSG00000255137 | RP11-290D2.4 | Up | 1 |
|  |  |  |  |  |  |  |  |  |  |  |  |  |
| **EWS and Ac-H3K9** | | |  |  |  |  |  |  |  |  |  |  |
| **Sample** | **Ch.** | **peak start** | **peak end** | **length** | **tags** | **p-value** | **F.C** | **FDR** | **ENS ID** | **Gene name** | **location** | **T** |
| EWS | 3 | 1.70E+08 | 1.70E+08 | 826 | 15 | 86.92 | 15.7 | 65.7 | ENSG00000008952 | SEC62 | Exon | 12 |
| Ac-H3K9 | 3 | 1.70E+08 | 1.70E+08 | 1796 | 80 | 419.98 | 22.0 | 0.4 | ENSG00000008952 | SEC62 | Up | 12 |
| EWS | 6 | 1.37E+08 | 1.37E+08 | 1242 | 30 | 107.98 | 7.1 | 82.8 | ENSG00000029363 | BCLAF1 | Down | 23 |
| EWS | 6 | 1.37E+08 | 1.37E+08 | 1227 | 31 | 86.63 | 7.2 | 65.1 | ENSG00000029363 | BCLAF1 | Down | 23 |
| Ac-H3K9 | 6 | 1.37E+08 | 1.37E+08 | 1111 | 47 | 209.04 | 17.7 | 0.53 | ENSG00000029363 | BCLAF1 | Down | 23 |
| Ac-H3K9 | 6 | 1.37E+08 | 1.37E+08 | 1171 | 46 | 194.74 | 10.2 | 0.54 | ENSG00000029363 | BCLAF1 | Down | 23 |
| Ac-H3K9 | 6 | 1.37E+08 | 1.37E+08 | 2514 | 140 | 642.89 | 35.1 | 0.53 | ENSG00000029363 | BCLAF1 | Intron | 23 |
| Ac-H3K9 | 6 | 1.37E+08 | 1.37E+08 | 1420 | 101 | 725.14 | 41.6 | 0.49 | ENSG00000029363 | BCLAF1 | Up | 23 |
| Ac-H3K9 | 15 | 6.08E+07 | 6.08E+07 | 1425 | 64 | 442.21 | 30.7 | 0.43 | ENSG00000069667 | RORA | Down | 4 |
| Ac-H3K9 | 15 | 6.08E+07 | 6.08E+07 | 600 | 16 | 97.48 | 15.3 | 0.78 | ENSG00000069667 | RORA | Down | 4 |
| EWS | 15 | 6.08E+07 | 6.08E+07 | 715 | 17 | 128.12 | 20.4 | 100 | ENSG00000069667 | RORA | Intron | 4 |
| Ac-H3K9 | 17 | 7.47E+07 | 7.47E+07 | 1318 | 56 | 407.28 | 42.1 | 0.37 | ENSG00000070495 | JMJD6 | Down | 5 |
| Ac-H3K9 | 17 | 7.47E+07 | 7.47E+07 | 3446 | 188 | 1255.8 | 33.1 | 0.84 | ENSG00000070495 | JMJD6 | Up | 5 |
| EWS | 17 | 7.47E+07 | 7.47E+07 | 1001 | 19 | 100.74 | 9.5 | 75.3 | ENSG00000070495 | JMJD6 | Up | 5 |
| EWS | 17 | 7.47E+07 | 7.47E+07 | 1140 | 21 | 80.16 | 7.0 | 56.1 | ENSG00000070495 | JMJD6 | Up | 5 |
| Ac-H3K9 | 17 | 7.47E+07 | 7.47E+07 | 1098 | 32 | 109.38 | 13.2 | 0.75 | ENSG00000070495 | JMJD6 | Up | 5 |
| EWS | 1 | 2.28E+08 | 2.28E+08 | 1688 | 32 | 141.7 | 7.6 | 100 | ENSG00000081692 | JMJD4 | Down | 5 |
| Ac-H3K9 | 1 | 2.28E+08 | 2.28E+08 | 1064 | 54 | 283.14 | 17.5 | 0.55 | ENSG00000081692 | JMJD4 | Exon | 5 |
| EWS | 9 | 3.30E+07 | 3.30E+07 | 1235 | 25 | 108.9 | 8.2 | 85.3 | ENSG00000086061 | DNAJA1 | Down | 5 |
| Ac-H3K9 | 9 | 3.30E+07 | 3.30E+07 | 2548 | 144 | 1124.2 | 50.6 | 0.91 | ENSG00000086061 | DNAJA1 | Up | 5 |
| Ac-H3K9 | 19 | 5.00E+07 | 5.00E+07 | 2346 | 298 | 2807.1 | 63.7 | 12.5 | ENSG00000090554 | FLT3LG | Down | 2 |
| EWS | 19 | 5.00E+07 | 5.00E+07 | 1490 | 35 | 115.84 | 8.6 | 86 | ENSG00000090554 | FLT3LG | Down | 2 |
| Ac-H3K9 | 19 | 5.00E+07 | 5.00E+07 | 1731 | 58 | 285.35 | 18.4 | 0.54 | ENSG00000090554 | FLT3LG | Down | 2 |
| Ac-H3K9 | 17 | 7.47E+07 | 7.47E+07 | 801 | 20 | 87.16 | 8.6 | 0.78 | ENSG00000092931 | MFSD11 | Exon | 2 |
| Ac-H3K9 | 17 | 7.47E+07 | 7.47E+07 | 1185 | 42 | 289.73 | 27.9 | 0.55 | ENSG00000092931 | MFSD11 | Intron | 2 |
| Ac-H3K9 | 17 | 7.47E+07 | 7.47E+07 | 1098 | 32 | 109.38 | 13.2 | 0.75 | ENSG00000092931 | MFSD11 | Up | 2 |
| EWS | 17 | 7.47E+07 | 7.47E+07 | 1140 | 21 | 80.16 | 7.0 | 56.1 | ENSG00000092931 | MFSD11 | Up | 2 |
| EWS | 17 | 7.47E+07 | 7.47E+07 | 1001 | 19 | 100.74 | 9.5 | 75.3 | ENSG00000092931 | MFSD11 | Up | 2 |
| Ac-H3K9 | 17 | 7.47E+07 | 7.47E+07 | 3446 | 188 | 1255.8 | 33.1 | 0.84 | ENSG00000092931 | MFSD11 | Up | 2 |
| Ac-H3K9 | 12 | 5.47E+07 | 5.47E+07 | 1966 | 164 | 1148.2 | 23.3 | 0.98 | ENSG00000094916 | CBX5 | Intron | 2 |
| EWS | 12 | 5.47E+07 | 5.47E+07 | 1207 | 31 | 117.63 | 9.3 | 93.3 | ENSG00000094916 | CBX5 | Up | 2 |
| Ac-H3K9 | 12 | 5.47E+07 | 5.47E+07 | 4376 | 307 | 1777 | 37.6 | 2.63 | ENSG00000094916 | CBX5 | Up | 2 |
| Ac-H3K9 | 10 | 1.02E+08 | 1.02E+08 | 836 | 24 | 100.67 | 10.6 | 0.76 | ENSG00000099194 | SCD | Down | 2 |
| Ac-H3K9 | 10 | 1.02E+08 | 1.02E+08 | 1282 | 116 | 934.3 | 57.3 | 0.54 | ENSG00000099194 | SCD | Down | 2 |
| Ac-H3K9 | 10 | 1.02E+08 | 1.02E+08 | 1916 | 143 | 1253.2 | 35.1 | 0.83 | ENSG00000099194 | SCD | Exon | 2 |
| EWS | 10 | 1.02E+08 | 1.02E+08 | 719 | 18 | 95.23 | 10.0 | 69.9 | ENSG00000099194 | SCD | Exon | 2 |
| Ac-H3K9 | 10 | 1.02E+08 | 1.02E+08 | 1061 | 50 | 194.41 | 16.9 | 0.54 | ENSG00000099194 | SCD | Up | 2 |
| EWS | 22 | 2.01E+07 | 2.01E+07 | 933 | 17 | 106 | 11.7 | 81.8 | ENSG00000099901 | RANBP1 | Down | 15 |
| Ac-H3K9 | 22 | 2.01E+07 | 2.01E+07 | 1791 | 116 | 545.13 | 20.3 | 0.51 | ENSG00000099901 | RANBP1 | Intron | 15 |
| Ac-H3K9 | 22 | 2.01E+07 | 2.01E+07 | 1170 | 57 | 414.94 | 32.2 | 0.39 | ENSG00000099901 | RANBP1 | Intron | 15 |
| Ac-H3K9 | 22 | 2.01E+07 | 2.01E+07 | 1791 | 116 | 545.13 | 20.3 | 0.51 | ENSG00000099904 | ZDHHC8 | Up | 7 |
| EWS | 22 | 2.01E+07 | 2.01E+07 | 933 | 17 | 106 | 11.7 | 81.8 | ENSG00000099904 | ZDHHC8 | Up | 7 |
| EWS | 22 | 3.89E+07 | 3.89E+07 | 1598 | 41 | 137.79 | 6.7 | 100 | ENSG00000100201 | DDX17 | Down | 14 |
| Ac-H3K9 | 22 | 3.89E+07 | 3.89E+07 | 3921 | 252 | 1579.4 | 36.8 | 1.87 | ENSG00000100201 | DDX17 | Intron | 14 |
| Ac-H3K9 | 9 | 3.78E+07 | 3.78E+07 | 2278 | 78 | 501.43 | 29.8 | 0.49 | ENSG00000107371 | EXOSC3 | Exon | 8 |
| Ac-H3K9 | 9 | 3.78E+07 | 3.78E+07 | 1012 | 41 | 300.66 | 34.3 | 0.48 | ENSG00000107371 | EXOSC3 | Intron | 8 |
| EWS | 9 | 3.78E+07 | 3.78E+07 | 728 | 15 | 91.64 | 13.5 | 66 | ENSG00000107371 | EXOSC3 | Up | 8 |
| Ac-H3K9 | 9 | 3.78E+07 | 3.78E+07 | 2705 | 124 | 478.86 | 13.0 | 0.45 | ENSG00000107371 | EXOSC3 | Up | 8 |
| EWS | 9 | 3.30E+07 | 3.30E+07 | 1235 | 25 | 108.9 | 8.2 | 85.3 | ENSG00000122692 | SMU1 | Down | 2 |
| Ac-H3K9 | 9 | 3.31E+07 | 3.31E+07 | 1348 | 79 | 703.5 | 46.7 | 0.56 | ENSG00000122692 | SMU1 | Intron | 2 |
| Ac-H3K9 | 9 | 3.78E+07 | 3.78E+07 | 1012 | 41 | 300.66 | 34.3 | 0.48 | ENSG00000122741 | DCAF10 | Exon | 5 |
| Ac-H3K9 | 9 | 3.78E+07 | 3.78E+07 | 2705 | 124 | 478.86 | 13.0 | 0.45 | ENSG00000122741 | DCAF10 | Intron | 5 |
| EWS | 9 | 3.78E+07 | 3.78E+07 | 728 | 15 | 91.64 | 13.5 | 66 | ENSG00000122741 | DCAF10 | Intron | 5 |
| Ac-H3K9 | 12 | 5.47E+07 | 5.47E+07 | 4376 | 307 | 1777 | 37.6 | 2.63 | ENSG00000123405 | NFE2 | Down | 3 |
| EWS | 12 | 5.47E+07 | 5.47E+07 | 1207 | 31 | 117.63 | 9.3 | 93.3 | ENSG00000123405 | NFE2 | Down | 3 |
| EWS | 20 | 3.43E+07 | 3.43E+07 | 613 | 14 | 83.93 | 11.8 | 60.7 | ENSG00000125995 | ROMO1 | Down | 5 |
| Ac-H3K9 | 20 | 3.43E+07 | 3.43E+07 | 1155 | 53 | 374.41 | 23.0 | 0.39 | ENSG00000125995 | ROMO1 | Exon | 5 |
| Ac-H3K9 | 20 | 3.43E+07 | 3.43E+07 | 668 | 19 | 113.42 | 14.5 | 0.75 | ENSG00000125995 | ROMO1 | Up | 5 |
| EWS | 7 | 1.30E+08 | 1.30E+08 | 441 | 12 | 92.47 | 13.6 | 68.1 | ENSG00000128607 | KLHDC10 | Intron | 4 |
| Ac-H3K9 | 7 | 1.30E+08 | 1.30E+08 | 1566 | 47 | 290.51 | 22.3 | 0.53 | ENSG00000128607 | KLHDC10 | Intron | 4 |
| Ac-H3K9 | 7 | 1.30E+08 | 1.30E+08 | 1081 | 52 | 214.03 | 13.0 | 0.52 | ENSG00000128607 | KLHDC10 | Up | 4 |
| Ac-H3K9 | 14 | 3.50E+07 | 3.50E+07 | 632 | 21 | 149.4 | 31.8 | 0.64 | ENSG00000129515 | SNX6 | Down | 4 |
| EWS | 14 | 3.50E+07 | 3.50E+07 | 969 | 19 | 97.1 | 11.3 | 72.5 | ENSG00000129515 | SNX6 | Down | 4 |
| Ac-H3K9 | 14 | 3.51E+07 | 3.51E+07 | 1023 | 28 | 143.85 | 14.6 | 0.66 | ENSG00000129515 | SNX6 | Intron | 4 |
| EWS | 19 | 5.70E+06 | 5.70E+06 | 1226 | 20 | 127.85 | 11.9 | 100 | ENSG00000130255 | RPL36 | Down | 2 |
| Ac-H3K9 | 19 | 5.69E+06 | 5.69E+06 | 1493 | 119 | 1177.2 | 54.1 | 1.06 | ENSG00000130255 | RPL36 | Exon | 2 |
| Ac-H3K9 | 19 | 5.68E+06 | 5.68E+06 | 829 | 27 | 184.52 | 20.6 | 0.54 | ENSG00000130255 | RPL36 | Up | 2 |
| Ac-H3K9 | 20 | 3.43E+07 | 3.43E+07 | 1155 | 53 | 374.41 | 23.0 | 0.39 | ENSG00000131051 | RBM39 | Down | 45 |
| EWS | 20 | 3.43E+07 | 3.43E+07 | 613 | 14 | 83.93 | 11.8 | 60.7 | ENSG00000131051 | RBM39 | Down | 45 |
| Ac-H3K9 | 20 | 3.43E+07 | 3.43E+07 | 668 | 19 | 113.42 | 14.5 | 0.75 | ENSG00000131051 | RBM39 | Down | 45 |
| Ac-H3K9 | 20 | 3.43E+07 | 3.43E+07 | 2038 | 119 | 842.14 | 45.0 | 0.42 | ENSG00000131051 | RBM39 | Intron | 45 |
| Ac-H3K9 | 20 | 3.43E+07 | 3.43E+07 | 1371 | 126 | 1195 | 85.6 | 1.12 | ENSG00000131051 | RBM39 | Up | 45 |
| EWS | 11 | 3.64E+07 | 3.64E+07 | 1198 | 24 | 120.03 | 9.6 | 95.5 | ENSG00000135362 | PRR5L | Intron | 21 |
| Ac-H3K9 | 11 | 3.63E+07 | 3.63E+07 | 754 | 23 | 153.79 | 20.6 | 0.64 | ENSG00000135362 | PRR5L | Up | 21 |
| EWS | 12 | 5.47E+07 | 5.47E+07 | 1207 | 31 | 117.63 | 9.3 | 93.3 | ENSG00000135486 | HNRNPA1 | Down | 3 |
| Ac-H3K9 | 12 | 5.47E+07 | 5.47E+07 | 4376 | 307 | 1777 | 37.6 | 2.63 | ENSG00000135486 | HNRNPA1 | Intron | 3 |
| Ac-H3K9 | 12 | 5.47E+07 | 5.47E+07 | 1966 | 164 | 1148.2 | 23.3 | 0.98 | ENSG00000135486 | HNRNPA1 | Up | 3 |
| Ac-H3K9 | 17 | 6.22E+07 | 6.22E+07 | 1648 | 106 | 627.31 | 21.9 | 0.51 | ENSG00000136478 | TEX2 | Down | 1 |
| Ac-H3K9 | 17 | 6.22E+07 | 6.22E+07 | 919 | 25 | 107.58 | 11.6 | 0.75 | ENSG00000136478 | TEX2 | Down | 1 |
| EWS | 17 | 6.22E+07 | 6.22E+07 | 1487 | 38 | 162.5 | 9.1 | 100 | ENSG00000136478 | TEX2 | Exon | 1 |
| Ac-H3K9 | 17 | 6.23E+07 | 6.23E+07 | 792 | 21 | 113.45 | 13.8 | 0.75 | ENSG00000136478 | TEX2 | Intron | 1 |
| Ac-H3K9 | 17 | 6.23E+07 | 6.23E+07 | 598 | 16 | 90.9 | 18.4 | 0.77 | ENSG00000136478 | TEX2 | Up | 1 |
| EWS | 3 | 1.86E+08 | 1.86E+08 | 1123 | 20 | 96.88 | 9.2 | 71.6 | ENSG00000136527 | TRA2B | Down | 16 |
| Ac-H3K9 | 3 | 1.86E+08 | 1.86E+08 | 5056 | 318 | 2035.6 | 34.0 | 3.57 | ENSG00000136527 | TRA2B | Exon | 16 |
| EWS | 1 | 2.20E+08 | 2.20E+08 | 955 | 24 | 102.02 | 7.2 | 78.6 | ENSG00000136628 | EPRS | Down | 7 |
| Ac-H3K9 | 1 | 2.20E+08 | 2.20E+08 | 1006 | 51 | 298.77 | 19.4 | 0.48 | ENSG00000136628 | EPRS | Intron | 7 |
| Ac-H3K9 | 16 | 7.74E+07 | 7.74E+07 | 826 | 19 | 93.67 | 12.3 | 0.78 | ENSG00000140873 | ADAMTS18 | Intron | 3 |
| EWS | 16 | 7.74E+07 | 7.74E+07 | 849 | 22 | 112.6 | 12.0 | 88.7 | ENSG00000140873 | ADAMTS18 | Intron | 3 |
| Ac-H3K9 | 16 | 1.99E+06 | 1.99E+06 | 441 | 14 | 102.96 | 20.6 | 0.77 | ENSG00000140986 | RPL3L | Down | 1 |
| Ac-H3K9 | 16 | 1.99E+06 | 1.99E+06 | 609 | 17 | 112.13 | 24.3 | 0.75 | ENSG00000140986 | RPL3L | Down | 1 |
| Ac-H3K9 | 16 | 2.01E+06 | 2.01E+06 | 2609 | 200 | 982.64 | 18.4 | 0.62 | ENSG00000140986 | RPL3L | Up | 1 |
| EWS | 16 | 2.01E+06 | 2.01E+06 | 1114 | 22 | 88.6 | 8.0 | 64.4 | ENSG00000140986 | RPL3L | Up | 1 |
| Ac-H3K9 | 16 | 2.01E+06 | 2.01E+06 | 1124 | 32 | 92.25 | 8.4 | 0.77 | ENSG00000140986 | RPL3L | Up | 1 |
| Ac-H3K9 | 16 | 2.01E+06 | 2.01E+06 | 1486 | 70 | 416.63 | 25.5 | 0.39 | ENSG00000140986 | RPL3L | Up | 1 |
| EWS | 16 | 2.01E+06 | 2.01E+06 | 1114 | 22 | 88.6 | 8.0 | 64.4 | ENSG00000140988 | RPS2 | Down | 17 |
| Ac-H3K9 | 16 | 2.01E+06 | 2.01E+06 | 1124 | 32 | 92.25 | 8.4 | 0.77 | ENSG00000140988 | RPS2 | Down | 17 |
| Ac-H3K9 | 16 | 2.01E+06 | 2.01E+06 | 1486 | 70 | 416.63 | 25.5 | 0.39 | ENSG00000140988 | RPS2 | Down | 17 |
| Ac-H3K9 | 16 | 2.01E+06 | 2.01E+06 | 2609 | 200 | 982.64 | 18.4 | 0.62 | ENSG00000140988 | RPS2 | Exon | 17 |
| Ac-H3K9 | 16 | 2.02E+06 | 2.02E+06 | 1430 | 51 | 349.89 | 44.9 | 0.44 | ENSG00000140988 | RPS2 | Up | 17 |
| Ac-H3K9 | 16 | 2.01E+06 | 2.02E+06 | 1300 | 61 | 415.46 | 28.5 | 0.39 | ENSG00000140988 | RPS2 | Up | 17 |
| Ac-H3K9 | 16 | 2.01E+06 | 2.01E+06 | 2609 | 200 | 982.64 | 18.4 | 0.62 | ENSG00000140990 | NDUFB10 | Down | 2 |
| Ac-H3K9 | 16 | 2.01E+06 | 2.02E+06 | 1300 | 61 | 415.46 | 28.5 | 0.39 | ENSG00000140990 | NDUFB10 | Down | 2 |
| Ac-H3K9 | 16 | 2.02E+06 | 2.02E+06 | 1430 | 51 | 349.89 | 44.9 | 0.44 | ENSG00000140990 | NDUFB10 | Down | 2 |
| EWS | 16 | 2.01E+06 | 2.01E+06 | 1114 | 22 | 88.6 | 8.0 | 64.4 | ENSG00000140990 | NDUFB10 | Exon | 2 |
| Ac-H3K9 | 16 | 2.01E+06 | 2.01E+06 | 1486 | 70 | 416.63 | 25.5 | 0.39 | ENSG00000140990 | NDUFB10 | Intron | 2 |
| Ac-H3K9 | 16 | 2.01E+06 | 2.01E+06 | 1124 | 32 | 92.25 | 8.4 | 0.77 | ENSG00000140990 | NDUFB10 | Intron | 2 |
| EWS | 17 | 7.98E+07 | 7.98E+07 | 838 | 15 | 91.94 | 14.1 | 66.7 | ENSG00000141522 | ARHGDIA | Down | 3 |
| Ac-H3K9 | 17 | 7.98E+07 | 7.98E+07 | 956 | 49 | 402.69 | 36.1 | 0.37 | ENSG00000141522 | ARHGDIA | Down | 3 |
| Ac-H3K9 | 17 | 7.98E+07 | 7.98E+07 | 1000 | 41 | 239.09 | 27.0 | 0.48 | ENSG00000141522 | ARHGDIA | Down | 3 |
| Ac-H3K9 | 17 | 7.98E+07 | 7.98E+07 | 1491 | 59 | 426.33 | 31.8 | 0.4 | ENSG00000141522 | ARHGDIA | Intron | 3 |
| Ac-H3K9 | 17 | 7.98E+07 | 7.98E+07 | 1402 | 81 | 715.52 | 50.5 | 0.57 | ENSG00000141522 | ARHGDIA | Up | 3 |
| Ac-H3K9 | 19 | 5.00E+07 | 5.00E+07 | 1602 | 173 | 1514.7 | 59.3 | 1.53 | ENSG00000142534 | RPS11 | Intron | 1 |
| Ac-H3K9 | 19 | 5.00E+07 | 5.00E+07 | 2346 | 298 | 2807.1 | 63.7 | 12.5 | ENSG00000142534 | RPS11 | Up | 1 |
| EWS | 19 | 5.00E+07 | 5.00E+07 | 1490 | 35 | 115.84 | 8.6 | 86 | ENSG00000142534 | RPS11 | Up | 1 |
| Ac-H3K9 | 19 | 5.00E+07 | 5.00E+07 | 1731 | 58 | 285.35 | 18.4 | 0.54 | ENSG00000142534 | RPS11 | Up | 1 |
| EWS | 19 | 5.00E+07 | 5.00E+07 | 1490 | 35 | 115.84 | 8.6 | 86 | ENSG00000142541 | RPL13A | Down | 13 |
| Ac-H3K9 | 19 | 5.00E+07 | 5.00E+07 | 1731 | 58 | 285.35 | 18.4 | 0.54 | ENSG00000142541 | RPL13A | Down | 13 |
| Ac-H3K9 | 19 | 5.00E+07 | 5.00E+07 | 1602 | 173 | 1514.7 | 59.3 | 1.53 | ENSG00000142541 | RPL13A | Down | 13 |
| Ac-H3K9 | 19 | 5.00E+07 | 5.00E+07 | 2346 | 298 | 2807.1 | 63.7 | 12.5 | ENSG00000142541 | RPL13A | Intron | 13 |
| EWS | 1 | 6.79E+07 | 6.79E+07 | 1193 | 28 | 83.2 | 6.4 | 59 | ENSG00000142864 | SERBP1 | Exon | 8 |
| Ac-H3K9 | 1 | 6.79E+07 | 6.79E+07 | 4024 | 289 | 1856.1 | 30.7 | 2.9 | ENSG00000142864 | SERBP1 | Intron | 8 |
| Ac-H3K9 | 1 | 6.79E+07 | 6.79E+07 | 1252 | 51 | 130.95 | 8.7 | 0.68 | ENSG00000142864 | SERBP1 | Up | 8 |
| Ac-H3K9 | 1 | 6.79E+07 | 6.79E+07 | 1328 | 89 | 470.76 | 20.4 | 0.43 | ENSG00000142864 | SERBP1 | Up | 8 |
| Ac-H3K9 | 1 | 4.52E+07 | 4.52E+07 | 2156 | 180 | 1382.1 | 35.8 | 1.16 | ENSG00000142959 | BEST4 | Down | 1 |
| Ac-H3K9 | 1 | 4.52E+07 | 4.52E+07 | 774 | 33 | 257.75 | 48.6 | 0.5 | ENSG00000142959 | BEST4 | Down | 1 |
| Ac-H3K9 | 1 | 4.53E+07 | 4.53E+07 | 1017 | 23 | 126.18 | 15.0 | 0.68 | ENSG00000142959 | BEST4 | Exon | 1 |
| EWS | 1 | 4.53E+07 | 4.53E+07 | 2119 | 65 | 184.45 | 6.5 | 100 | ENSG00000142959 | BEST4 | Up | 1 |
| Ac-H3K9 | 1 | 2.28E+08 | 2.28E+08 | 1064 | 54 | 283.14 | 17.5 | 0.55 | ENSG00000143740 | SNAP47 | Intron | 11 |
| EWS | 1 | 2.28E+08 | 2.28E+08 | 1688 | 32 | 141.7 | 7.6 | 100 | ENSG00000143740 | SNAP47 | Up | 11 |
| Ac-H3K9 | 1 | 2.26E+08 | 2.26E+08 | 3045 | 126 | 553.62 | 15.1 | 0.53 | ENSG00000143799 | PARP1 | Intron | 11 |
| Ac-H3K9 | 1 | 2.24E+08 | 2.24E+08 | 657 | 33 | 168.1 | 16.1 | 0.6 | ENSG00000143799 | PARP1 | Intron | 11 |
| Ac-H3K9 | 1 | 2.24E+08 | 2.24E+08 | 1620 | 99 | 650.66 | 29.1 | 0.55 | ENSG00000143799 | PARP1 | Intron | 11 |
| EWS | 1 | 2.26E+08 | 2.26E+08 | 1743 | 42 | 120.75 | 7.5 | 100 | ENSG00000143799 | PARP1 | Intron | 11 |
| Ac-H3K9 | 1 | 2.26E+08 | 2.26E+08 | 1294 | 79 | 407.72 | 20.7 | 0.38 | ENSG00000143799 | PARP1 | Intron | 11 |
| Ac-H3K9 | 1 | 2.26E+08 | 2.26E+08 | 3111 | 266 | 1139.2 | 17.8 | 0.95 | ENSG00000143799 | PARP1 | Intron | 11 |
| Ac-H3K9 | 1 | 2.26E+08 | 2.26E+08 | 1347 | 43 | 180.78 | 11.4 | 0.54 | ENSG00000143799 | PARP1 | Intron | 11 |
| Ac-H3K9 | 1 | 2.26E+08 | 2.26E+08 | 1187 | 41 | 210.06 | 14.6 | 0.51 | ENSG00000143799 | PARP1 | Intron | 11 |
| Ac-H3K9 | 1 | 2.26E+08 | 2.26E+08 | 681 | 26 | 104.31 | 10.0 | 0.75 | ENSG00000143799 | PARP1 | Intron | 11 |
| Ac-H3K9 | 1 | 2.26E+08 | 2.26E+08 | 1507 | 99 | 452.33 | 19.1 | 0.45 | ENSG00000143799 | PARP1 | Intron | 11 |
| Ac-H3K9 | 1 | 2.26E+08 | 2.26E+08 | 808 | 36 | 187.03 | 14.2 | 0.53 | ENSG00000143799 | PARP1 | Intron | 11 |
| Ac-H3K9 | 1 | 2.26E+08 | 2.26E+08 | 3804 | 174 | 466.58 | 12.9 | 0.43 | ENSG00000143799 | PARP1 | Intron | 11 |
| Ac-H3K9 | 1 | 2.26E+08 | 2.26E+08 | 1090 | 76 | 256.8 | 11.9 | 0.5 | ENSG00000143799 | PARP1 | Intron | 11 |
| Ac-H3K9 | 1 | 2.26E+08 | 2.26E+08 | 1949 | 88 | 533.65 | 27.0 | 0.49 | ENSG00000143799 | PARP1 | Intron | 11 |
| Ac-H3K9 | 1 | 2.26E+08 | 2.26E+08 | 3235 | 204 | 924.96 | 23.9 | 0.53 | ENSG00000143799 | PARP1 | Intron | 11 |
| Ac-H3K9 | 1 | 2.26E+08 | 2.26E+08 | 3935 | 302 | 1365 | 24.1 | 1.1 | ENSG00000143799 | PARP1 | Intron | 11 |
| Ac-H3K9 | 1 | 2.26E+08 | 2.26E+08 | 1314 | 50 | 154.05 | 11.5 | 0.64 | ENSG00000143799 | PARP1 | Intron | 11 |
| Ac-H3K9 | 1 | 2.26E+08 | 2.26E+08 | 2372 | 87 | 426.55 | 16.8 | 0.4 | ENSG00000143799 | PARP1 | Intron | 11 |
| Ac-H3K9 | 1 | 2.26E+08 | 2.26E+08 | 1141 | 65 | 303.63 | 17.7 | 0.49 | ENSG00000143799 | PARP1 | Intron | 11 |
| Ac-H3K9 | 1 | 2.26E+08 | 2.26E+08 | 1647 | 96 | 368.16 | 18.6 | 0.41 | ENSG00000143799 | PARP1 | Intron | 11 |
| Ac-H3K9 | 1 | 2.26E+08 | 2.26E+08 | 1128 | 67 | 252.37 | 13.8 | 0.51 | ENSG00000143799 | PARP1 | Intron | 11 |
| Ac-H3K9 | 1 | 2.27E+08 | 2.27E+08 | 3173 | 186 | 952.07 | 30.7 | 0.58 | ENSG00000143799 | PARP1 | Intron | 11 |
| Ac-H3K9 | 1 | 2.25E+08 | 2.25E+08 | 1632 | 80 | 436.05 | 34.5 | 0.42 | ENSG00000143799 | PARP1 | Intron | 11 |
| Ac-H3K9 | 1 | 2.25E+08 | 2.25E+08 | 2554 | 118 | 305.66 | 9.4 | 0.49 | ENSG00000143799 | PARP1 | Intron | 11 |
| Ac-H3K9 | 1 | 2.25E+08 | 2.25E+08 | 2136 | 119 | 620.97 | 22.1 | 0.57 | ENSG00000143799 | PARP1 | Intron | 11 |
| Ac-H3K9 | 1 | 2.25E+08 | 2.25E+08 | 2765 | 240 | 1558.3 | 32.5 | 1.75 | ENSG00000143799 | PARP1 | Intron | 11 |
| Ac-H3K9 | 1 | 2.25E+08 | 2.25E+08 | 1724 | 76 | 259.85 | 9.2 | 0.5 | ENSG00000143799 | PARP1 | Intron | 11 |
| Ac-H3K9 | 1 | 2.24E+08 | 2.24E+08 | 914 | 39 | 151.69 | 13.3 | 0.65 | ENSG00000143799 | PARP1 | Intron | 11 |
| Ac-H3K9 | 1 | 2.24E+08 | 2.24E+08 | 2034 | 104 | 594.64 | 27.2 | 0.59 | ENSG00000143799 | PARP1 | Intron | 11 |
| Ac-H3K9 | 1 | 2.24E+08 | 2.24E+08 | 2428 | 151 | 569.49 | 13.4 | 0.55 | ENSG00000143799 | PARP1 | Intron | 11 |
| Ac-H3K9 | 1 | 2.27E+08 | 2.27E+08 | 582 | 33 | 177.18 | 22.2 | 0.55 | ENSG00000143799 | PARP1 | Up | 11 |
| EWS | 3 | 1.29E+07 | 1.29E+07 | 921 | 17 | 84 | 11.0 | 60.3 | ENSG00000144712 | CAND2 | Intron | 5 |
| Ac-H3K9 | 3 | 1.29E+07 | 1.29E+07 | 2779 | 169 | 1362.3 | 44.5 | 1.09 | ENSG00000144712 | CAND2 | Intron | 5 |
| Ac-H3K9 | 3 | 1.28E+07 | 1.28E+07 | 526 | 14 | 81.24 | 12.3 | 0.8 | ENSG00000144712 | CAND2 | Up | 5 |
| Ac-H3K9 | 3 | 1.29E+07 | 1.29E+07 | 2779 | 169 | 1362.3 | 44.5 | 1.09 | ENSG00000144713 | RPL32 | Exon | 8 |
| EWS | 3 | 1.29E+07 | 1.29E+07 | 921 | 17 | 84 | 11.0 | 60.3 | ENSG00000144713 | RPL32 | Exon | 8 |
| EWS | 4 | 2.03E+07 | 2.03E+07 | 960 | 24 | 137.3 | 11.4 | 100 | ENSG00000145147 | SLIT2 | Intron | 12 |
| Ac-H3K9 | 4 | 2.03E+07 | 2.03E+07 | 5685 | 262 | 1942.4 | 43.8 | 3.12 | ENSG00000145147 | SLIT2 | Intron | 12 |
| Ac-H3K9 | 4 | 2.03E+07 | 2.03E+07 | 435 | 14 | 89.32 | 17.5 | 0.77 | ENSG00000145147 | SLIT2 | Intron | 12 |
| Ac-H3K9 | 4 | 2.03E+07 | 2.03E+07 | 853 | 37 | 275.01 | 27.3 | 0.54 | ENSG00000145147 | SLIT2 | Up | 12 |
| Ac-H3K9 | 6 | 1.37E+08 | 1.37E+08 | 1171 | 46 | 194.74 | 10.2 | 0.54 | ENSG00000146410 | FAM54A | Exon | 7 |
| EWS | 6 | 1.37E+08 | 1.37E+08 | 1242 | 30 | 107.98 | 7.1 | 82.8 | ENSG00000146410 | FAM54A | Up | 7 |
| EWS | 6 | 1.37E+08 | 1.37E+08 | 1227 | 31 | 86.63 | 7.2 | 65.1 | ENSG00000146410 | FAM54A | Up | 7 |
| Ac-H3K9 | 6 | 1.37E+08 | 1.37E+08 | 1111 | 47 | 209.04 | 17.7 | 0.53 | ENSG00000146410 | FAM54A | Up | 7 |
| Ac-H3K9 | X | 9.59E+07 | 9.59E+07 | 2265 | 101 | 471.93 | 15.8 | 0.44 | ENSG00000147202 | DIAPH2 | Intron | 6 |
| EWS | X | 9.64E+07 | 9.64E+07 | 1347 | 23 | 100.76 | 9.5 | 76.4 | ENSG00000147202 | DIAPH2 | Intron | 6 |
| EWS | 1 | 2.45E+08 | 2.45E+08 | 3824 | 114 | 158.54 | 5.7 | 100 | ENSG00000153187 | HNRNPU | Down | 9 |
| EWS | 1 | 2.45E+08 | 2.45E+08 | 2487 | 56 | 109.04 | 5.7 | 86.4 | ENSG00000153187 | HNRNPU | Down | 9 |
| Ac-H3K9 | 1 | 2.45E+08 | 2.45E+08 | 2906 | 248 | 1161.8 | 17.9 | 1.01 | ENSG00000153187 | HNRNPU | Exon | 9 |
| Ac-H3K9 | 1 | 2.45E+08 | 2.45E+08 | 1146 | 106 | 715.43 | 23.1 | 0.57 | ENSG00000153187 | HNRNPU | Exon | 9 |
| Ac-H3K9 | 1 | 2.45E+08 | 2.45E+08 | 2663 | 187 | 798.25 | 24.0 | 0.36 | ENSG00000153187 | HNRNPU | Up | 9 |
| Ac-H3K9 | 1 | 2.45E+08 | 2.45E+08 | 1276 | 50 | 197.58 | 12.8 | 0.51 | ENSG00000153187 | HNRNPU | Up | 9 |
| EWS | 4 | 7.34E+07 | 7.34E+07 | 1576 | 32 | 92.05 | 7.1 | 67.4 | ENSG00000156140 | ADAMTS3 | Intron | 3 |
| Ac-H3K9 | 4 | 7.34E+07 | 7.34E+07 | 2865 | 89 | 302.32 | 11.4 | 0.48 | ENSG00000156140 | ADAMTS3 | Intron | 3 |
| EWS | 21 | 4.45E+07 | 4.45E+07 | 704 | 20 | 97.72 | 10.0 | 75.3 | ENSG00000160200 | CBS | Down | 20 |
| Ac-H3K9 | 21 | 4.45E+07 | 4.45E+07 | 1834 | 83 | 647.73 | 33.7 | 0.54 | ENSG00000160200 | CBS | Exon | 20 |
| Ac-H3K9 | 21 | 4.45E+07 | 4.45E+07 | 743 | 22 | 145.22 | 20.6 | 0.65 | ENSG00000160200 | CBS | Exon | 20 |
| Ac-H3K9 | 21 | 4.45E+07 | 4.45E+07 | 643 | 18 | 117.91 | 18.7 | 0.74 | ENSG00000160200 | CBS | Up | 20 |
| EWS | 22 | 2.20E+07 | 2.20E+07 | 831 | 21 | 110.05 | 11.4 | 87.7 | ENSG00000161179 | YDJC | Down | 7 |
| Ac-H3K9 | 22 | 2.20E+07 | 2.20E+07 | 1819 | 72 | 475.91 | 37.8 | 0.44 | ENSG00000161179 | YDJC | Exon | 7 |
| Ac-H3K9 | 22 | 2.20E+07 | 2.20E+07 | 1085 | 40 | 130.75 | 9.5 | 0.68 | ENSG00000161180 | CCDC116 | Down | 2 |
| Ac-H3K9 | 22 | 2.20E+07 | 2.20E+07 | 1225 | 37 | 191.95 | 13.9 | 0.54 | ENSG00000161180 | CCDC116 | Down | 2 |
| EWS | 22 | 2.20E+07 | 2.20E+07 | 831 | 21 | 110.05 | 11.4 | 87.7 | ENSG00000161180 | CCDC116 | Up | 2 |
| Ac-H3K9 | 22 | 2.20E+07 | 2.20E+07 | 1819 | 72 | 475.91 | 37.8 | 0.44 | ENSG00000161180 | CCDC116 | Up | 2 |
| EWS | 17 | 7.47E+07 | 7.47E+07 | 1001 | 19 | 100.74 | 9.5 | 75.3 | ENSG00000161547 | SRSF2 | Down | 5 |
| EWS | 17 | 7.47E+07 | 7.47E+07 | 1140 | 21 | 80.16 | 7.0 | 56.1 | ENSG00000161547 | SRSF2 | Down | 5 |
| Ac-H3K9 | 17 | 7.47E+07 | 7.47E+07 | 1098 | 32 | 109.38 | 13.2 | 0.75 | ENSG00000161547 | SRSF2 | Down | 5 |
| Ac-H3K9 | 17 | 7.47E+07 | 7.47E+07 | 3446 | 188 | 1255.8 | 33.1 | 0.84 | ENSG00000161547 | SRSF2 | Exon | 5 |
| Ac-H3K9 | 17 | 7.47E+07 | 7.47E+07 | 801 | 20 | 87.16 | 8.6 | 0.78 | ENSG00000161547 | SRSF2 | Up | 5 |
| Ac-H3K9 | 17 | 7.47E+07 | 7.47E+07 | 1185 | 42 | 289.73 | 27.9 | 0.55 | ENSG00000161547 | SRSF2 | Up | 5 |
| EWS | 1 | 2.26E+08 | 2.26E+08 | 1743 | 42 | 120.75 | 7.5 | 100 | ENSG00000163041 | H3F3A | Down | 4 |
| Ac-H3K9 | 1 | 2.26E+08 | 2.26E+08 | 3111 | 266 | 1139.2 | 17.8 | 0.95 | ENSG00000163041 | H3F3A | Intron | 4 |
| Ac-H3K9 | 1 | 2.26E+08 | 2.26E+08 | 1294 | 79 | 407.72 | 20.7 | 0.38 | ENSG00000163041 | H3F3A | Up | 4 |
| Ac-H3K9 | 15 | 7.96E+07 | 7.96E+07 | 1128 | 46 | 325.33 | 25.6 | 0.5 | ENSG00000166557 | TMED3 | Intron | 4 |
| EWS | 15 | 7.96E+07 | 7.96E+07 | 1011 | 23 | 107.7 | 12.5 | 83.1 | ENSG00000166557 | TMED3 | Intron | 4 |
| EWS | 18 | 3.26E+07 | 3.26E+07 | 1415 | 36 | 128.8 | 7.1 | 100 | ENSG00000166974 | MAPRE2 | Intron | 4 |
| Ac-H3K9 | 18 | 3.26E+07 | 3.26E+07 | 996 | 22 | 82.82 | 8.4 | 0.79 | ENSG00000166974 | MAPRE2 | Intron | 4 |
| Ac-H3K9 | 18 | 3.26E+07 | 3.26E+07 | 2329 | 105 | 379.05 | 16.2 | 0.4 | ENSG00000166974 | MAPRE2 | Intron | 4 |
| Ac-H3K9 | 19 | 5.69E+06 | 5.69E+06 | 1493 | 119 | 1177.2 | 54.1 | 1.06 | ENSG00000167733 | HSD11B1L | Down | 6 |
| EWS | 19 | 5.70E+06 | 5.70E+06 | 1226 | 20 | 127.85 | 11.9 | 100 | ENSG00000167733 | HSD11B1L | Down | 6 |
| Ac-H3K9 | 19 | 5.68E+06 | 5.68E+06 | 829 | 27 | 184.52 | 20.6 | 0.54 | ENSG00000167733 | HSD11B1L | Up | 6 |
| EWS | 3 | 3.95E+07 | 3.95E+07 | 1066 | 19 | 85.8 | 7.1 | 63.9 | ENSG00000168028 | RPSA | Down | 10 |
| Ac-H3K9 | 3 | 3.94E+07 | 3.95E+07 | 3059 | 227 | 1491.2 | 34.2 | 1.46 | ENSG00000168028 | RPSA | Intron | 10 |
| Ac-H3K9 | 19 | 5.47E+07 | 5.47E+07 | 1763 | 109 | 791.19 | 34.4 | 0.36 | ENSG00000170889 | RPS9 | Exon | 12 |
| Ac-H3K9 | 19 | 5.47E+07 | 5.47E+07 | 1149 | 28 | 113.31 | 11.0 | 0.75 | ENSG00000170889 | RPS9 | Intron | 12 |
| EWS | 19 | 5.47E+07 | 5.47E+07 | 1220 | 22 | 100.1 | 7.5 | 74.3 | ENSG00000170889 | RPS9 | Intron | 12 |
| Ac-H3K9 | 15 | 6.61E+07 | 6.61E+07 | 1411 | 73 | 487.79 | 22.1 | 0.46 | ENSG00000174485 | DENND4A | Intron | 3 |
| EWS | 15 | 6.60E+07 | 6.60E+07 | 801 | 19 | 94.51 | 11.0 | 66.7 | ENSG00000174485 | DENND4A | Intron | 3 |
| Ac-H3K9 | 15 | 6.61E+07 | 6.61E+07 | 828 | 46 | 337.34 | 24.0 | 0.45 | ENSG00000174485 | DENND4A | Up | 3 |
| Ac-H3K9 | 3 | 2.40E+07 | 2.40E+07 | 1949 | 125 | 845.87 | 22.2 | 0.42 | ENSG00000174748 | RPL15 | Exon | 14 |
| EWS | 3 | 2.40E+07 | 2.40E+07 | 1425 | 29 | 111.34 | 9.4 | 87.3 | ENSG00000174748 | RPL15 | Intron | 14 |
| Ac-H3K9 | 3 | 2.40E+07 | 2.40E+07 | 1258 | 47 | 136.45 | 9.8 | 0.69 | ENSG00000174748 | RPL15 | Up | 14 |
| EWS | 17 | 8.08E+06 | 8.08E+06 | 811 | 15 | 100.02 | 13.6 | 73.3 | ENSG00000179029 | TMEM107 | Down | 10 |
| Ac-H3K9 | 17 | 8.08E+06 | 8.08E+06 | 355 | 25 | 196.14 | 26.1 | 0.51 | ENSG00000179029 | TMEM107 | Exon | 10 |
| Ac-H3K9 | 17 | 8.09E+06 | 8.09E+06 | 435 | 23 | 148.79 | 14.3 | 0.64 | ENSG00000179029 | TMEM107 | Up | 10 |
| Ac-H3K9 | 16 | 2.02E+06 | 2.02E+06 | 1430 | 51 | 349.89 | 44.9 | 0.44 | ENSG00000179580 | RNF151 | Down | 1 |
| Ac-H3K9 | 16 | 2.01E+06 | 2.01E+06 | 1486 | 70 | 416.63 | 25.5 | 0.39 | ENSG00000179580 | RNF151 | Up | 1 |
| EWS | 16 | 2.01E+06 | 2.01E+06 | 1114 | 22 | 88.6 | 8.0 | 64.4 | ENSG00000179580 | RNF151 | Up | 1 |
| Ac-H3K9 | 16 | 2.01E+06 | 2.01E+06 | 1124 | 32 | 92.25 | 8.4 | 0.77 | ENSG00000179580 | RNF151 | Up | 1 |
| Ac-H3K9 | 16 | 2.01E+06 | 2.01E+06 | 2609 | 200 | 982.64 | 18.4 | 0.62 | ENSG00000179580 | RNF151 | Up | 1 |
| Ac-H3K9 | 16 | 2.01E+06 | 2.02E+06 | 1300 | 61 | 415.46 | 28.5 | 0.39 | ENSG00000179580 | RNF151 | Up | 1 |
| Ac-H3K9 | 17 | 7.47E+07 | 7.47E+07 | 3446 | 188 | 1255.8 | 33.1 | 0.84 | ENSG00000181038 | C17orf95 | Down | 2 |
| Ac-H3K9 | 17 | 7.47E+07 | 7.47E+07 | 1185 | 42 | 289.73 | 27.9 | 0.55 | ENSG00000181038 | C17orf95 | Down | 2 |
| Ac-H3K9 | 17 | 7.47E+07 | 7.47E+07 | 801 | 20 | 87.16 | 8.6 | 0.78 | ENSG00000181038 | C17orf95 | Down | 2 |
| Ac-H3K9 | 17 | 7.47E+07 | 7.47E+07 | 1098 | 32 | 109.38 | 13.2 | 0.75 | ENSG00000181038 | C17orf95 | Intron | 2 |
| EWS | 17 | 7.47E+07 | 7.47E+07 | 1140 | 21 | 80.16 | 7.0 | 56.1 | ENSG00000181038 | C17orf95 | Intron | 2 |
| EWS | 17 | 7.47E+07 | 7.47E+07 | 1001 | 19 | 100.74 | 9.5 | 75.3 | ENSG00000181038 | C17orf95 | Intron | 2 |
| Ac-H3K9 | 5 | 1.71E+08 | 1.71E+08 | 927 | 144 | 1396.5 | 65.7 | 1.18 | ENSG00000181163 | NPM1 | Intron | 12 |
| Ac-H3K9 | 5 | 1.71E+08 | 1.71E+08 | 3819 | 243 | 1530.2 | 34.5 | 1.57 | ENSG00000181163 | NPM1 | Intron | 12 |
| EWS | 5 | 1.71E+08 | 1.71E+08 | 1372 | 24 | 115.83 | 9.2 | 84.3 | ENSG00000181163 | NPM1 | Intron | 12 |
| EWS | 22 | 3.93E+07 | 3.93E+07 | 1211 | 25 | 90.05 | 7.5 | 67 | ENSG00000183741 | CBX6 | Down | 3 |
| Ac-H3K9 | 22 | 3.93E+07 | 3.93E+07 | 1414 | 45 | 255.26 | 19.9 | 0.49 | ENSG00000183741 | CBX6 | Intron | 3 |
| EWS | 8 | 3.76E+07 | 3.76E+07 | 1734 | 36 | 84.4 | 6.2 | 62 | ENSG00000183779 | ZNF703 | Down | 2 |
| Ac-H3K9 | 8 | 3.76E+07 | 3.76E+07 | 5586 | 388 | 2740.4 | 51.4 | 20 | ENSG00000183779 | ZNF703 | Exon | 2 |
| Ac-H3K9 | 8 | 3.76E+07 | 3.76E+07 | 1801 | 77 | 585.26 | 29.9 | 0.58 | ENSG00000183779 | ZNF703 | Intron | 2 |
| Ac-H3K9 | 8 | 3.75E+07 | 3.75E+07 | 748 | 20 | 92.22 | 13.5 | 0.77 | ENSG00000183779 | ZNF703 | Up | 2 |
| Ac-H3K9 | 8 | 3.76E+07 | 3.76E+07 | 1164 | 60 | 498.3 | 31.8 | 0.48 | ENSG00000183779 | ZNF703 | Up | 2 |
| Ac-H3K9 | 10 | 5.28E+07 | 5.28E+07 | 1083 | 28 | 164.03 | 14.9 | 0.64 | ENSG00000185532 | PRKG1 | Exon | 6 |
| Ac-H3K9 | 10 | 5.28E+07 | 5.28E+07 | 776 | 24 | 115.49 | 10.4 | 0.75 | ENSG00000185532 | PRKG1 | Intron | 6 |
| EWS | 10 | 5.29E+07 | 5.29E+07 | 1005 | 25 | 93.16 | 7.9 | 70.5 | ENSG00000185532 | PRKG1 | Intron | 6 |
| Ac-H3K9 | 10 | 5.35E+07 | 5.35E+07 | 1772 | 99 | 680.92 | 29.5 | 0.52 | ENSG00000185532 | PRKG1 | Intron | 6 |
| Ac-H3K9 | 17 | 7.98E+07 | 7.98E+07 | 784 | 26 | 180.31 | 26.2 | 0.54 | ENSG00000185624 | P4HB | Down | 16 |
| Ac-H3K9 | 17 | 7.98E+07 | 7.98E+07 | 1000 | 41 | 239.09 | 27.0 | 0.48 | ENSG00000185624 | P4HB | Intron | 16 |
| EWS | 17 | 7.98E+07 | 7.98E+07 | 838 | 15 | 91.94 | 14.1 | 66.7 | ENSG00000185624 | P4HB | Up | 16 |
| Ac-H3K9 | 17 | 7.98E+07 | 7.98E+07 | 1491 | 59 | 426.33 | 31.8 | 0.4 | ENSG00000185624 | P4HB | Up | 16 |
| Ac-H3K9 | 17 | 7.98E+07 | 7.98E+07 | 956 | 49 | 402.69 | 36.1 | 0.37 | ENSG00000185624 | P4HB | Up | 16 |
| EWS | 22 | 2.20E+07 | 2.20E+07 | 831 | 21 | 110.05 | 11.4 | 87.7 | ENSG00000185651 | UBE2L3 | Down | 4 |
| Ac-H3K9 | 22 | 2.20E+07 | 2.20E+07 | 1819 | 72 | 475.91 | 37.8 | 0.44 | ENSG00000185651 | UBE2L3 | Down | 4 |
| Ac-H3K9 | 22 | 2.19E+07 | 2.19E+07 | 2444 | 89 | 274.12 | 10.9 | 0.53 | ENSG00000185651 | UBE2L3 | Intron | 4 |
| EWS | 12 | 7.64E+07 | 7.64E+07 | 1182 | 25 | 108.43 | 9.8 | 83.9 | ENSG00000187109 | NAP1L1 | Exon | 7 |
| Ac-H3K9 | 12 | 7.65E+07 | 7.65E+07 | 600 | 16 | 93.98 | 16.1 | 0.78 | ENSG00000187109 | NAP1L1 | Intron | 7 |
| Ac-H3K9 | 12 | 7.65E+07 | 7.65E+07 | 2153 | 86 | 480.7 | 20.9 | 0.45 | ENSG00000187109 | NAP1L1 | Intron | 7 |
| EWS | 2 | 2.33E+08 | 2.33E+08 | 2182 | 63 | 250.74 | 10.7 | 100 | ENSG00000187514 | PTMA | Down | 13 |
| Ac-H3K9 | 2 | 2.33E+08 | 2.33E+08 | 1261 | 39 | 244.38 | 23.5 | 0.49 | ENSG00000187514 | PTMA | Exon | 13 |
| Ac-H3K9 | 2 | 2.33E+08 | 2.33E+08 | 1651 | 64 | 455.31 | 43.0 | 0.41 | ENSG00000187514 | PTMA | Exon | 13 |
| Ac-H3K9 | 2 | 2.33E+08 | 2.33E+08 | 854 | 58 | 549.31 | 43.0 | 0.52 | ENSG00000187514 | PTMA | Intron | 13 |
| Ac-H3K9 | 2 | 2.33E+08 | 2.33E+08 | 1013 | 53 | 446.07 | 35.5 | 0.44 | ENSG00000187514 | PTMA | Up | 13 |
| EWS | 2 | 2.35E+08 | 2.35E+08 | 588 | 13 | 82.49 | 10.0 | 58.9 | ENSG00000188042 | ARL4C | Down | 2 |
| Ac-H3K9 | 2 | 2.35E+08 | 2.35E+08 | 981 | 26 | 112.52 | 10.8 | 0.75 | ENSG00000188042 | ARL4C | Exon | 2 |
| Ac-H3K9 | 2 | 2.35E+08 | 2.35E+08 | 526 | 14 | 84.12 | 17.8 | 0.79 | ENSG00000188042 | ARL4C | Up | 2 |
| Ac-H3K9 | 1 | 2.45E+08 | 2.45E+08 | 4621 | 294 | 1214.2 | 20.7 | 1.17 | ENSG00000188206 | NCRNA00201 | Down | 3 |
| EWS | 1 | 2.45E+08 | 2.45E+08 | 2487 | 56 | 109.04 | 5.7 | 86.4 | ENSG00000188206 | NCRNA00201 | Exon | 3 |
| EWS | 1 | 2.45E+08 | 2.45E+08 | 3824 | 114 | 158.54 | 5.7 | 100 | ENSG00000188206 | NCRNA00201 | Intron | 3 |
| Ac-H3K9 | 1 | 2.45E+08 | 2.45E+08 | 2663 | 187 | 798.25 | 24.0 | 0.36 | ENSG00000188206 | NCRNA00201 | Up | 3 |
| Ac-H3K9 | 1 | 2.45E+08 | 2.45E+08 | 1146 | 106 | 715.43 | 23.1 | 0.57 | ENSG00000188206 | NCRNA00201 | Up | 3 |
| Ac-H3K9 | 1 | 2.45E+08 | 2.45E+08 | 2906 | 248 | 1161.8 | 17.9 | 1.01 | ENSG00000188206 | NCRNA00201 | Up | 3 |
| EWS | 1 | 9.30E+05 | 9.31E+05 | 888 | 17 | 109.74 | 16.7 | 86.2 | ENSG00000188290 | HES4 | Down | 4 |
| Ac-H3K9 | 1 | 9.35E+05 | 9.35E+05 | 686 | 18 | 113.15 | 18.7 | 0.75 | ENSG00000188290 | HES4 | Exon | 4 |
| Ac-H3K9 | 1 | 9.36E+05 | 9.36E+05 | 594 | 28 | 210.43 | 30.7 | 0.51 | ENSG00000188290 | HES4 | Up | 4 |
| Ac-H3K9 | 1 | 9.36E+05 | 9.37E+05 | 1037 | 68 | 511.34 | 31.9 | 0.46 | ENSG00000188290 | HES4 | Up | 4 |
| Ac-H3K9 | 19 | 5.69E+06 | 5.69E+06 | 1493 | 119 | 1177.2 | 54.1 | 1.06 | ENSG00000196365 | LONP1 | Down | 3 |
| Ac-H3K9 | 19 | 5.72E+06 | 5.72E+06 | 1702 | 90 | 757.62 | 48.6 | 0.43 | ENSG00000196365 | LONP1 | Intron | 3 |
| EWS | 19 | 5.70E+06 | 5.70E+06 | 1226 | 20 | 127.85 | 11.9 | 100 | ENSG00000196365 | LONP1 | Intron | 3 |
| Ac-H3K9 | 19 | 5.72E+06 | 5.72E+06 | 346 | 18 | 165.04 | 31.8 | 0.63 | ENSG00000196365 | LONP1 | Up | 3 |
| Ac-H3K9 | 3 | 2.40E+07 | 2.40E+07 | 1258 | 47 | 136.45 | 9.8 | 0.69 | ENSG00000197885 | NKIRAS1 | Exon | 8 |
| Ac-H3K9 | 3 | 2.40E+07 | 2.40E+07 | 934 | 34 | 215.55 | 24.5 | 0.53 | ENSG00000197885 | NKIRAS1 | Intron | 8 |
| EWS | 3 | 2.40E+07 | 2.40E+07 | 1425 | 29 | 111.34 | 9.4 | 87.3 | ENSG00000197885 | NKIRAS1 | Intron | 8 |
| Ac-H3K9 | 3 | 2.40E+07 | 2.40E+07 | 1949 | 125 | 845.87 | 22.2 | 0.42 | ENSG00000197885 | NKIRAS1 | Intron | 8 |
| Ac-H3K9 | 17 | 5.67E+07 | 5.67E+07 | 924 | 37 | 202.7 | 20.4 | 0.53 | ENSG00000199426 | U1.5 | Down | 1 |
| EWS | 17 | 5.67E+07 | 5.67E+07 | 810 | 17 | 116.76 | 13.3 | 93.5 | ENSG00000199426 | U1.5 | Down | 1 |
| EWS | 19 | 5.00E+07 | 5.00E+07 | 1490 | 35 | 115.84 | 8.6 | 86 | ENSG00000199631 | SNORD33 | Down | 1 |
| Ac-H3K9 | 19 | 5.00E+07 | 5.00E+07 | 1731 | 58 | 285.35 | 18.4 | 0.54 | ENSG00000199631 | SNORD33 | Down | 1 |
| Ac-H3K9 | 19 | 5.00E+07 | 5.00E+07 | 1602 | 173 | 1514.7 | 59.3 | 1.53 | ENSG00000199631 | SNORD33 | Down | 1 |
| Ac-H3K9 | 19 | 5.00E+07 | 5.00E+07 | 2346 | 298 | 2807.1 | 63.7 | 12.5 | ENSG00000199631 | SNORD33 | Up | 1 |
| Ac-H3K9 | 17 | 6.22E+07 | 6.22E+07 | 1648 | 106 | 627.31 | 21.9 | 0.51 | ENSG00000199753 | SNORD104 | Down | 1 |
| EWS | 17 | 6.22E+07 | 6.22E+07 | 1487 | 38 | 162.5 | 9.1 | 100 | ENSG00000199753 | SNORD104 | Down | 1 |
| Ac-H3K9 | 17 | 6.22E+07 | 6.22E+07 | 919 | 25 | 107.58 | 11.6 | 0.75 | ENSG00000199753 | SNORD104 | Up | 1 |
| EWS | 19 | 5.00E+07 | 5.00E+07 | 1490 | 35 | 115.84 | 8.6 | 86 | ENSG00000200259 | SNORD35A | Down | 1 |
| Ac-H3K9 | 19 | 5.00E+07 | 5.00E+07 | 1731 | 58 | 285.35 | 18.4 | 0.54 | ENSG00000200259 | SNORD35A | Down | 1 |
| Ac-H3K9 | 19 | 5.00E+07 | 5.00E+07 | 1602 | 173 | 1514.7 | 59.3 | 1.53 | ENSG00000200259 | SNORD35A | Down | 1 |
| Ac-H3K9 | 19 | 5.00E+07 | 5.00E+07 | 2346 | 298 | 2807.1 | 63.7 | 12.5 | ENSG00000200259 | SNORD35A | Up | 1 |
| EWS | 17 | 8.08E+06 | 8.08E+06 | 811 | 15 | 100.02 | 13.6 | 73.3 | ENSG00000200463 | U8.4 | Down | 1 |
| Ac-H3K9 | 17 | 8.08E+06 | 8.08E+06 | 355 | 25 | 196.14 | 26.1 | 0.51 | ENSG00000200463 | U8.4 | Up | 1 |
| Ac-H3K9 | 19 | 5.00E+07 | 5.00E+07 | 2346 | 298 | 2807.1 | 63.7 | 12.5 | ENSG00000200530 | SNORD35B | Up | 1 |
| EWS | 19 | 5.00E+07 | 5.00E+07 | 1490 | 35 | 115.84 | 8.6 | 86 | ENSG00000200530 | SNORD35B | Up | 1 |
| Ac-H3K9 | 19 | 5.00E+07 | 5.00E+07 | 1731 | 58 | 285.35 | 18.4 | 0.54 | ENSG00000200530 | SNORD35B | Up | 1 |
| Ac-H3K9 | 19 | 5.00E+07 | 5.00E+07 | 1602 | 173 | 1514.7 | 59.3 | 1.53 | ENSG00000200530 | SNORD35B | Up | 1 |
| EWS | 19 | 5.00E+07 | 5.00E+07 | 1490 | 35 | 115.84 | 8.6 | 86 | ENSG00000201675 | SNORD32A | Down | 1 |
| Ac-H3K9 | 19 | 5.00E+07 | 5.00E+07 | 1731 | 58 | 285.35 | 18.4 | 0.54 | ENSG00000201675 | SNORD32A | Down | 1 |
| Ac-H3K9 | 19 | 5.00E+07 | 5.00E+07 | 1602 | 173 | 1514.7 | 59.3 | 1.53 | ENSG00000201675 | SNORD32A | Down | 1 |
| Ac-H3K9 | 19 | 5.00E+07 | 5.00E+07 | 2346 | 298 | 2807.1 | 63.7 | 12.5 | ENSG00000201675 | SNORD32A | Up | 1 |
| Ac-H3K9 | 6 | 8.64E+07 | 8.64E+07 | 2833 | 229 | 1353 | 25.9 | 1.05 | ENSG00000201865 | SNORD50B | Down | 1 |
| EWS | 6 | 8.64E+07 | 8.64E+07 | 680 | 15 | 90.99 | 11.3 | 66.3 | ENSG00000201865 | SNORD50B | Down | 1 |
| Ac-H3K9 | 6 | 8.64E+07 | 8.64E+07 | 1691 | 90 | 438.28 | 20.7 | 0.42 | ENSG00000201865 | SNORD50B | Up | 1 |
| EWS | 17 | 5.67E+07 | 5.67E+07 | 810 | 17 | 116.76 | 13.3 | 93.5 | ENSG00000202077 | U1.57 | Down | 1 |
| Ac-H3K9 | 17 | 5.67E+07 | 5.67E+07 | 924 | 37 | 202.7 | 20.4 | 0.53 | ENSG00000202077 | U1.57 | Up | 1 |
| EWS | 3 | 3.95E+07 | 3.95E+07 | 1066 | 19 | 85.8 | 7.1 | 63.9 | ENSG00000202363 | SNORA62.4 | Down | 1 |
| Ac-H3K9 | 3 | 3.94E+07 | 3.95E+07 | 3059 | 227 | 1491.2 | 34.2 | 1.46 | ENSG00000202363 | SNORA62.4 | Up | 1 |
| EWS | 19 | 5.00E+07 | 5.00E+07 | 1490 | 35 | 115.84 | 8.6 | 86 | ENSG00000202503 | SNORD34 | Down | 1 |
| Ac-H3K9 | 19 | 5.00E+07 | 5.00E+07 | 1731 | 58 | 285.35 | 18.4 | 0.54 | ENSG00000202503 | SNORD34 | Down | 1 |
| Ac-H3K9 | 19 | 5.00E+07 | 5.00E+07 | 1602 | 173 | 1514.7 | 59.3 | 1.53 | ENSG00000202503 | SNORD34 | Down | 1 |
| Ac-H3K9 | 19 | 5.00E+07 | 5.00E+07 | 2346 | 298 | 2807.1 | 63.7 | 12.5 | ENSG00000202503 | SNORD34 | Up | 1 |
| EWS | 1 | 2.45E+08 | 2.45E+08 | 3824 | 114 | 158.54 | 5.7 | 100 | ENSG00000203667 | FAM36A | Down | 5 |
| EWS | 1 | 2.45E+08 | 2.45E+08 | 2487 | 56 | 109.04 | 5.7 | 86.4 | ENSG00000203667 | FAM36A | Intron | 5 |
| Ac-H3K9 | 1 | 2.45E+08 | 2.45E+08 | 4621 | 294 | 1214.2 | 20.7 | 1.17 | ENSG00000203667 | FAM36A | Up | 5 |
| EWS | 6 | 8.64E+07 | 8.64E+07 | 680 | 15 | 90.99 | 11.3 | 66.3 | ENSG00000203875 | SNHG5 | Down | 10 |
| Ac-H3K9 | 6 | 8.64E+07 | 8.64E+07 | 2833 | 229 | 1353 | 25.9 | 1.05 | ENSG00000203875 | SNHG5 | Exon | 10 |
| Ac-H3K9 | 6 | 8.64E+07 | 8.64E+07 | 1691 | 90 | 438.28 | 20.7 | 0.42 | ENSG00000203875 | SNHG5 | Up | 10 |
| Ac-H3K9 | 14 | 3.50E+07 | 3.50E+07 | 481 | 14 | 84.12 | 14.8 | 0.79 | ENSG00000206588 | RNU1-8 | Down | 1 |
| EWS | 14 | 3.50E+07 | 3.50E+07 | 969 | 19 | 97.1 | 11.3 | 72.5 | ENSG00000206588 | RNU1-8 | Down | 1 |
| Ac-H3K9 | 14 | 3.50E+07 | 3.50E+07 | 632 | 21 | 149.4 | 31.8 | 0.64 | ENSG00000206588 | RNU1-8 | Up | 1 |
| EWS | 14 | 3.50E+07 | 3.50E+07 | 969 | 19 | 97.1 | 11.3 | 72.5 | ENSG00000206596 | RNU1-7 | Down | 1 |
| Ac-H3K9 | 14 | 3.50E+07 | 3.50E+07 | 632 | 21 | 149.4 | 31.8 | 0.64 | ENSG00000206596 | RNU1-7 | Down | 1 |
| Ac-H3K9 | 14 | 3.50E+07 | 3.50E+07 | 1230 | 69 | 593.84 | 44.9 | 0.59 | ENSG00000206596 | RNU1-7 | Up | 1 |
| Ac-H3K9 | 14 | 3.50E+07 | 3.50E+07 | 481 | 14 | 84.12 | 14.8 | 0.79 | ENSG00000206596 | RNU1-7 | Up | 1 |
| EWS | 3 | 3.95E+07 | 3.95E+07 | 1066 | 19 | 85.8 | 7.1 | 63.9 | ENSG00000206760 | SNORA6 | Down | 1 |
| Ac-H3K9 | 3 | 3.94E+07 | 3.95E+07 | 3059 | 227 | 1491.2 | 34.2 | 1.46 | ENSG00000206760 | SNORA6 | Up | 1 |
| Ac-H3K9 | 16 | 2.01E+06 | 2.01E+06 | 1124 | 32 | 92.25 | 8.4 | 0.77 | ENSG00000206811 | SNORA10 | Down | 1 |
| EWS | 16 | 2.01E+06 | 2.01E+06 | 1114 | 22 | 88.6 | 8.0 | 64.4 | ENSG00000206811 | SNORA10 | Down | 1 |
| Ac-H3K9 | 16 | 2.01E+06 | 2.01E+06 | 1486 | 70 | 416.63 | 25.5 | 0.39 | ENSG00000206811 | SNORA10 | Down | 1 |
| Ac-H3K9 | 16 | 2.02E+06 | 2.02E+06 | 1430 | 51 | 349.89 | 44.9 | 0.44 | ENSG00000206811 | SNORA10 | Up | 1 |
| Ac-H3K9 | 16 | 2.01E+06 | 2.02E+06 | 1300 | 61 | 415.46 | 28.5 | 0.39 | ENSG00000206811 | SNORA10 | Up | 1 |
| Ac-H3K9 | 16 | 2.01E+06 | 2.01E+06 | 2609 | 200 | 982.64 | 18.4 | 0.62 | ENSG00000206811 | SNORA10 | Up | 1 |
| Ac-H3K9 | 6 | 8.64E+07 | 8.64E+07 | 2833 | 229 | 1353 | 25.9 | 1.05 | ENSG00000207066 | SNORD50A | Down | 1 |
| EWS | 6 | 8.64E+07 | 8.64E+07 | 680 | 15 | 90.99 | 11.3 | 66.3 | ENSG00000207066 | SNORD50A | Down | 1 |
| Ac-H3K9 | 6 | 8.64E+07 | 8.64E+07 | 1691 | 90 | 438.28 | 20.7 | 0.42 | ENSG00000207066 | SNORD50A | Up | 1 |
| EWS | 16 | 2.01E+06 | 2.01E+06 | 1114 | 22 | 88.6 | 8.0 | 64.4 | ENSG00000207405 | SNORA64 | Down | 1 |
| Ac-H3K9 | 16 | 2.01E+06 | 2.01E+06 | 1124 | 32 | 92.25 | 8.4 | 0.77 | ENSG00000207405 | SNORA64 | Down | 1 |
| Ac-H3K9 | 16 | 2.01E+06 | 2.01E+06 | 1486 | 70 | 416.63 | 25.5 | 0.39 | ENSG00000207405 | SNORA64 | Down | 1 |
| Ac-H3K9 | 16 | 2.02E+06 | 2.02E+06 | 1430 | 51 | 349.89 | 44.9 | 0.44 | ENSG00000207405 | SNORA64 | Up | 1 |
| Ac-H3K9 | 16 | 2.01E+06 | 2.02E+06 | 1300 | 61 | 415.46 | 28.5 | 0.39 | ENSG00000207405 | SNORA64 | Up | 1 |
| Ac-H3K9 | 16 | 2.01E+06 | 2.01E+06 | 2609 | 200 | 982.64 | 18.4 | 0.62 | ENSG00000207405 | SNORA64 | Up | 1 |
| EWS | 3 | 1.29E+07 | 1.29E+07 | 921 | 17 | 84 | 11.0 | 60.3 | ENSG00000207496 | SNORA7A | Down | 1 |
| Ac-H3K9 | 3 | 1.29E+07 | 1.29E+07 | 2779 | 169 | 1362.3 | 44.5 | 1.09 | ENSG00000207496 | SNORA7A | Up | 1 |
| Ac-H3K9 | 17 | 7.47E+07 | 7.47E+07 | 3446 | 188 | 1255.8 | 33.1 | 0.84 | ENSG00000207556 | MIR636 | Down | 1 |
| EWS | 17 | 7.47E+07 | 7.47E+07 | 1001 | 19 | 100.74 | 9.5 | 75.3 | ENSG00000207556 | MIR636 | Down | 1 |
| EWS | 17 | 7.47E+07 | 7.47E+07 | 1140 | 21 | 80.16 | 7.0 | 56.1 | ENSG00000207556 | MIR636 | Down | 1 |
| Ac-H3K9 | 17 | 7.47E+07 | 7.47E+07 | 1098 | 32 | 109.38 | 13.2 | 0.75 | ENSG00000207556 | MIR636 | Down | 1 |
| Ac-H3K9 | 17 | 7.47E+07 | 7.47E+07 | 801 | 20 | 87.16 | 8.6 | 0.78 | ENSG00000207556 | MIR636 | Up | 1 |
| Ac-H3K9 | 17 | 7.47E+07 | 7.47E+07 | 1185 | 42 | 289.73 | 27.9 | 0.55 | ENSG00000207556 | MIR636 | Up | 1 |
| Ac-H3K9 | 19 | 5.00E+07 | 5.00E+07 | 1602 | 173 | 1514.7 | 59.3 | 1.53 | ENSG00000207782 | MIR150 | Down | 1 |
| Ac-H3K9 | 19 | 5.00E+07 | 5.00E+07 | 1731 | 58 | 285.35 | 18.4 | 0.54 | ENSG00000207782 | MIR150 | Down | 1 |
| EWS | 19 | 5.00E+07 | 5.00E+07 | 1490 | 35 | 115.84 | 8.6 | 86 | ENSG00000207782 | MIR150 | Down | 1 |
| Ac-H3K9 | X | 4.12E+07 | 4.12E+07 | 1463 | 91 | 761.77 | 45.6 | 0.43 | ENSG00000215301 | DDX3X | Intron | 7 |
| Ac-H3K9 | X | 4.12E+07 | 4.12E+07 | 1412 | 42 | 164.65 | 13.3 | 0.63 | ENSG00000215301 | DDX3X | Intron | 7 |
| EWS | X | 4.12E+07 | 4.12E+07 | 1473 | 44 | 123.23 | 6.5 | 100 | ENSG00000215301 | DDX3X | Intron | 7 |
| Ac-H3K9 | X | 4.12E+07 | 4.12E+07 | 852 | 26 | 153.45 | 17.3 | 0.64 | ENSG00000215301 | DDX3X | Up | 7 |
| Ac-H3K9 | 17 | 8.05E+06 | 8.06E+06 | 791 | 20 | 120.81 | 18.7 | 0.72 | ENSG00000220205 | VAMP2 | Intron | 5 |
| Ac-H3K9 | 17 | 8.08E+06 | 8.08E+06 | 355 | 25 | 196.14 | 26.1 | 0.51 | ENSG00000220205 | VAMP2 | Up | 5 |
| EWS | 17 | 8.08E+06 | 8.08E+06 | 811 | 15 | 100.02 | 13.6 | 73.3 | ENSG00000220205 | VAMP2 | Up | 5 |
| EWS | 17 | 6.22E+07 | 6.22E+07 | 1487 | 38 | 162.5 | 9.1 | 100 | ENSG00000221462 | SNORA76 | Down | 1 |
| Ac-H3K9 | 17 | 6.22E+07 | 6.22E+07 | 919 | 25 | 107.58 | 11.6 | 0.75 | ENSG00000221462 | SNORA76 | Up | 1 |
| Ac-H3K9 | 17 | 6.22E+07 | 6.22E+07 | 1648 | 106 | 627.31 | 21.9 | 0.51 | ENSG00000221462 | SNORA76 | Up | 1 |
| Ac-H3K9 | 1 | 6.79E+07 | 6.79E+07 | 4024 | 289 | 1856.1 | 30.7 | 2.9 | ENSG00000223263 | U6.930 | Down | 1 |
| EWS | 1 | 6.79E+07 | 6.79E+07 | 1193 | 28 | 83.2 | 6.4 | 59 | ENSG00000223263 | U6.930 | Up | 1 |
| EWS | 19 | 5.47E+07 | 5.47E+07 | 1220 | 22 | 100.1 | 7.5 | 74.3 | ENSG00000223660 | AC012314.20 | Up | 1 |
| Ac-H3K9 | 19 | 5.47E+07 | 5.47E+07 | 1149 | 28 | 113.31 | 11.0 | 0.75 | ENSG00000223660 | AC012314.20 | Up | 1 |
| Ac-H3K9 | 19 | 5.47E+07 | 5.47E+07 | 1763 | 109 | 791.19 | 34.4 | 0.36 | ENSG00000223660 | AC012314.20 | Up | 1 |
| Ac-H3K9 | 19 | 5.47E+07 | 5.47E+07 | 1149 | 28 | 113.31 | 11.0 | 0.75 | ENSG00000224579 | AC012314.19 | Down | 1 |
| Ac-H3K9 | 19 | 5.47E+07 | 5.47E+07 | 1763 | 109 | 791.19 | 34.4 | 0.36 | ENSG00000224579 | AC012314.19 | Down | 1 |
| EWS | 19 | 5.47E+07 | 5.47E+07 | 1220 | 22 | 100.1 | 7.5 | 74.3 | ENSG00000224579 | AC012314.19 | Up | 1 |
| Ac-H3K9 | 2 | 1.78E+08 | 1.78E+08 | 1555 | 152 | 956.7 | 19.0 | 0.59 | ENSG00000229337 | AC079305.8 | Intron | 1 |
| Ac-H3K9 | 2 | 1.78E+08 | 1.78E+08 | 625 | 21 | 99.74 | 14.3 | 0.76 | ENSG00000229337 | AC079305.8 | Intron | 1 |
| EWS | 2 | 1.78E+08 | 1.78E+08 | 3253 | 92 | 154.27 | 7.3 | 100 | ENSG00000229337 | AC079305.8 | Up | 1 |
| Ac-H3K9 | 2 | 1.78E+08 | 1.78E+08 | 529 | 19 | 115 | 17.0 | 0.75 | ENSG00000229337 | AC079305.8 | Up | 1 |
| Ac-H3K9 | 2 | 1.78E+08 | 1.78E+08 | 1403 | 136 | 775.67 | 25.3 | 0.34 | ENSG00000229337 | AC079305.8 | Up | 1 |
| Ac-H3K9 | 10 | 1.02E+08 | 1.02E+08 | 1282 | 116 | 934.3 | 57.3 | 0.54 | ENSG00000235823 | NCRNA00263 | Exon | 1 |
| EWS | 10 | 1.02E+08 | 1.02E+08 | 719 | 18 | 95.23 | 10.0 | 69.9 | ENSG00000235823 | NCRNA00263 | Up | 1 |
| Ac-H3K9 | 10 | 1.02E+08 | 1.02E+08 | 836 | 24 | 100.67 | 10.6 | 0.76 | ENSG00000235823 | NCRNA00263 | Up | 1 |
| Ac-H3K9 | 16 | 2.01E+06 | 2.02E+06 | 1300 | 61 | 415.46 | 28.5 | 0.39 | ENSG00000238671 | SNORA78 | Down | 1 |
| Ac-H3K9 | 16 | 2.02E+06 | 2.02E+06 | 1430 | 51 | 349.89 | 44.9 | 0.44 | ENSG00000238671 | SNORA78 | Down | 1 |
| Ac-H3K9 | 16 | 2.01E+06 | 2.01E+06 | 1486 | 70 | 416.63 | 25.5 | 0.39 | ENSG00000238671 | SNORA78 | Up | 1 |
| EWS | 16 | 2.01E+06 | 2.01E+06 | 1114 | 22 | 88.6 | 8.0 | 64.4 | ENSG00000238671 | SNORA78 | Up | 1 |
| Ac-H3K9 | 16 | 2.01E+06 | 2.01E+06 | 1124 | 32 | 92.25 | 8.4 | 0.77 | ENSG00000238671 | SNORA78 | Up | 1 |
| Ac-H3K9 | 16 | 2.01E+06 | 2.01E+06 | 2609 | 200 | 982.64 | 18.4 | 0.62 | ENSG00000238671 | SNORA78 | Up | 1 |
| Ac-H3K9 | 22 | 2.01E+07 | 2.01E+07 | 1791 | 116 | 545.13 | 20.3 | 0.51 | ENSG00000240816 | AC006547.1 | Down | 1 |
| EWS | 22 | 2.01E+07 | 2.01E+07 | 933 | 17 | 106 | 11.7 | 81.8 | ENSG00000240816 | AC006547.1 | Up | 1 |
| Ac-H3K9 | 20 | 3.43E+07 | 3.43E+07 | 668 | 19 | 113.42 | 14.5 | 0.75 | ENSG00000244005 | NFS1 | Exon | 19 |
| Ac-H3K9 | 20 | 3.43E+07 | 3.43E+07 | 909 | 38 | 104.38 | 6.7 | 0.75 | ENSG00000244005 | NFS1 | Intron | 19 |
| Ac-H3K9 | 20 | 3.43E+07 | 3.43E+07 | 1164 | 53 | 325.37 | 23.3 | 0.5 | ENSG00000244005 | NFS1 | Intron | 19 |
| Ac-H3K9 | 20 | 3.43E+07 | 3.43E+07 | 1155 | 53 | 374.41 | 23.0 | 0.39 | ENSG00000244005 | NFS1 | Up | 19 |
| EWS | 20 | 3.43E+07 | 3.43E+07 | 613 | 14 | 83.93 | 11.8 | 60.7 | ENSG00000244005 | NFS1 | Up | 19 |
| Ac-H3K9 | 11 | 6.52E+07 | 6.52E+07 | 1921 | 84 | 520.74 | 20.9 | 0.47 | ENSG00000245532 | NEAT1 | Exon | 2 |
| EWS | 11 | 6.52E+07 | 6.52E+07 | 1364 | 25 | 96.18 | 7.0 | 70.7 | ENSG00000245532 | NEAT1 | Exon | 2 |
| Ac-H3K9 | 11 | 6.52E+07 | 6.52E+07 | 693 | 18 | 99.92 | 15.7 | 0.76 | ENSG00000245532 | NEAT1 | Up | 2 |
| Ac-H3K9 | 11 | 6.52E+07 | 6.52E+07 | 976 | 23 | 92.97 | 8.6 | 0.78 | ENSG00000245532 | NEAT1 | Up | 2 |
| Ac-H3K9 | 11 | 6.52E+07 | 6.52E+07 | 552 | 19 | 105.57 | 19.3 | 0.75 | ENSG00000245532 | NEAT1 | Up | 2 |
| Ac-H3K9 | 15 | 6.08E+07 | 6.08E+07 | 600 | 16 | 97.48 | 15.3 | 0.78 | ENSG00000245534 | AC087385.1 | Intron | 1 |
| EWS | 15 | 6.08E+07 | 6.08E+07 | 715 | 17 | 128.12 | 20.4 | 100 | ENSG00000245534 | AC087385.1 | Intron | 1 |
| Ac-H3K9 | 15 | 6.08E+07 | 6.08E+07 | 1425 | 64 | 442.21 | 30.7 | 0.43 | ENSG00000245534 | AC087385.1 | Up | 1 |
| Ac-H3K9 | 11 | 8.28E+07 | 8.28E+07 | 1643 | 102 | 882.75 | 52.8 | 0.47 | ENSG00000246067 | RP11-113K21.5 | Intron | 8 |
| EWS | 11 | 8.28E+07 | 8.28E+07 | 860 | 15 | 80.61 | 9.5 | 57 | ENSG00000246067 | RP11-113K21.5 | Intron | 8 |
| EWS | 4 | 1.49E+07 | 1.49E+07 | 1391 | 30 | 80.64 | 6.9 | 57.5 | ENSG00000247624 | AC006296.3 | Intron | 2 |
| Ac-H3K9 | 4 | 1.50E+07 | 1.50E+07 | 451 | 13 | 80.81 | 12.3 | 0.82 | ENSG00000247624 | AC006296.3 | Up | 2 |
| EWS | 16 | 2.01E+06 | 2.01E+06 | 1114 | 22 | 88.6 | 8.0 | 64.4 | ENSG00000255066 | AC005363.7 | Down | 1 |
| Ac-H3K9 | 16 | 2.01E+06 | 2.01E+06 | 1124 | 32 | 92.25 | 8.4 | 0.77 | ENSG00000255066 | AC005363.7 | Down | 1 |
| Ac-H3K9 | 16 | 2.01E+06 | 2.01E+06 | 1486 | 70 | 416.63 | 25.5 | 0.39 | ENSG00000255066 | AC005363.7 | Down | 1 |
| Ac-H3K9 | 16 | 2.02E+06 | 2.02E+06 | 1430 | 51 | 349.89 | 44.9 | 0.44 | ENSG00000255066 | AC005363.7 | Up | 1 |
| Ac-H3K9 | 16 | 2.01E+06 | 2.02E+06 | 1300 | 61 | 415.46 | 28.5 | 0.39 | ENSG00000255066 | AC005363.7 | Up | 1 |
| Ac-H3K9 | 16 | 2.01E+06 | 2.01E+06 | 2609 | 200 | 982.64 | 18.4 | 0.62 | ENSG00000255066 | AC005363.7 | Up | 1 |
| Ac-H3K9 | 16 | 2.01E+06 | 2.02E+06 | 1300 | 61 | 415.46 | 28.5 | 0.39 | ENSG00000255198 | SNHG9 | Down | 1 |
| Ac-H3K9 | 16 | 2.02E+06 | 2.02E+06 | 1430 | 51 | 349.89 | 44.9 | 0.44 | ENSG00000255198 | SNHG9 | Down | 1 |
| Ac-H3K9 | 16 | 2.01E+06 | 2.01E+06 | 1486 | 70 | 416.63 | 25.5 | 0.39 | ENSG00000255198 | SNHG9 | Up | 1 |
| EWS | 16 | 2.01E+06 | 2.01E+06 | 1114 | 22 | 88.6 | 8.0 | 64.4 | ENSG00000255198 | SNHG9 | Up | 1 |
| Ac-H3K9 | 16 | 2.01E+06 | 2.01E+06 | 1124 | 32 | 92.25 | 8.4 | 0.77 | ENSG00000255198 | SNHG9 | Up | 1 |
| Ac-H3K9 | 16 | 2.01E+06 | 2.01E+06 | 2609 | 200 | 982.64 | 18.4 | 0.62 | ENSG00000255198 | SNHG9 | Up | 1 |
| Ac-H3K9 | 16 | 2.01E+06 | 2.01E+06 | 1124 | 32 | 92.25 | 8.4 | 0.77 | ENSG00000255278 | AC005363.8 | Down | 1 |
| EWS | 16 | 2.01E+06 | 2.01E+06 | 1114 | 22 | 88.6 | 8.0 | 64.4 | ENSG00000255278 | AC005363.8 | Down | 1 |
| Ac-H3K9 | 16 | 2.01E+06 | 2.01E+06 | 1486 | 70 | 416.63 | 25.5 | 0.39 | ENSG00000255278 | AC005363.8 | Down | 1 |
| Ac-H3K9 | 16 | 2.02E+06 | 2.02E+06 | 1430 | 51 | 349.89 | 44.9 | 0.44 | ENSG00000255278 | AC005363.8 | Up | 1 |
| Ac-H3K9 | 16 | 2.01E+06 | 2.02E+06 | 1300 | 61 | 415.46 | 28.5 | 0.39 | ENSG00000255278 | AC005363.8 | Up | 1 |
| Ac-H3K9 | 16 | 2.01E+06 | 2.01E+06 | 2609 | 200 | 982.64 | 18.4 | 0.62 | ENSG00000255278 | AC005363.8 | Up | 1 |
| Ac-H3K9 | 11 | 8.28E+07 | 8.28E+07 | 1643 | 102 | 882.75 | 52.8 | 0.47 | ENSG00000255503 | RP11-113K21.4 | Down | 1 |
| EWS | 11 | 8.28E+07 | 8.28E+07 | 860 | 15 | 80.61 | 9.5 | 57 | ENSG00000255503 | RP11-113K21.4 | Intron | 1 |
| EWS | 16 | 2.01E+06 | 2.01E+06 | 1114 | 22 | 88.6 | 8.0 | 64.4 | ENSG00000255513 | AC005363.9 | Down | 1 |
| Ac-H3K9 | 16 | 2.01E+06 | 2.01E+06 | 1124 | 32 | 92.25 | 8.4 | 0.77 | ENSG00000255513 | AC005363.9 | Down | 1 |
| Ac-H3K9 | 16 | 2.01E+06 | 2.01E+06 | 1486 | 70 | 416.63 | 25.5 | 0.39 | ENSG00000255513 | AC005363.9 | Down | 1 |
| Ac-H3K9 | 16 | 2.01E+06 | 2.01E+06 | 2609 | 200 | 982.64 | 18.4 | 0.62 | ENSG00000255513 | AC005363.9 | Exon | 1 |
| Ac-H3K9 | 16 | 2.02E+06 | 2.02E+06 | 1430 | 51 | 349.89 | 44.9 | 0.44 | ENSG00000255513 | AC005363.9 | Up | 1 |
| Ac-H3K9 | 16 | 2.01E+06 | 2.02E+06 | 1300 | 61 | 415.46 | 28.5 | 0.39 | ENSG00000255513 | AC005363.9 | Up | 1 |
| Ac-H3K9 | 9 | 3.76E+07 | 3.76E+07 | 1371 | 59 | 417.71 | 34.8 | 0.39 | ENSG00000255872 | RP11-613M10.9 | Intron | 1 |
| Ac-H3K9 | 9 | 3.79E+07 | 3.79E+07 | 1074 | 37 | 192.48 | 13.5 | 0.54 | ENSG00000255872 | RP11-613M10.9 | Intron | 1 |
| Ac-H3K9 | 9 | 3.79E+07 | 3.79E+07 | 628 | 25 | 172.54 | 23.0 | 0.55 | ENSG00000255872 | RP11-613M10.9 | Intron | 1 |
| EWS | 9 | 3.78E+07 | 3.78E+07 | 728 | 15 | 91.64 | 13.5 | 66 | ENSG00000255872 | RP11-613M10.9 | Intron | 1 |
| Ac-H3K9 | 9 | 3.78E+07 | 3.78E+07 | 2278 | 78 | 501.43 | 29.8 | 0.49 | ENSG00000255872 | RP11-613M10.9 | Intron | 1 |
| Ac-H3K9 | 9 | 3.78E+07 | 3.78E+07 | 1012 | 41 | 300.66 | 34.3 | 0.48 | ENSG00000255872 | RP11-613M10.9 | Intron | 1 |
| Ac-H3K9 | 9 | 3.78E+07 | 3.78E+07 | 2705 | 124 | 478.86 | 13.0 | 0.45 | ENSG00000255872 | RP11-613M10.9 | Intron | 1 |
| Ac-H3K9 | 9 | 3.78E+07 | 3.78E+07 | 673 | 29 | 230.27 | 33.7 | 0.51 | ENSG00000255872 | RP11-613M10.9 | Intron | 1 |
| Ac-H3K9 | 10 | 1.02E+08 | 1.02E+08 | 1282 | 116 | 934.3 | 57.3 | 0.54 | ENSG00000256545 | AL139819.1 | Down | 1 |
| EWS | 10 | 1.02E+08 | 1.02E+08 | 719 | 18 | 95.23 | 10.0 | 69.9 | ENSG00000256545 | AL139819.1 | Down | 1 |
| Ac-H3K9 | 10 | 1.02E+08 | 1.02E+08 | 836 | 24 | 100.67 | 10.6 | 0.76 | ENSG00000256545 | AL139819.1 | Down | 1 |
|  |  |  |  |  |  |  |  |  |  |  |  |  |
| **FUS, EWS, and Ac-H3K9** | | |  |  |  |  |  |  |  |  |  |  |
| **Sample** | **Ch.** | **peak start** | **peak end** | **length** | **tags** | **p-value** | **F.C** | **FDR** | **ENS ID** | **Gene name** | **location** | **T** |
| FUS | 7 | 2.62E+07 | 2.62E+07 | 3011 | 95 | 504.94 | 15.2 | 100 | ENSG00000050344 | NFE2L3 | Down | 2 |
| Ac-H3K9 | 7 | 2.62E+07 | 2.62E+07 | 4637 | 359 | 1772 | 25.1 | 2.6 | ENSG00000050344 | NFE2L3 | Down | 2 |
| FUS | 7 | 2.62E+07 | 2.62E+07 | 2147 | 51 | 201.43 | 9.2 | 100 | ENSG00000050344 | NFE2L3 | Exon | 2 |
| EWS | 7 | 2.62E+07 | 2.62E+07 | 742 | 24 | 131.86 | 9.2 | 100 | ENSG00000050344 | NFE2L3 | Exon | 2 |
| EWS | 7 | 2.62E+07 | 2.62E+07 | 4900 | 178 | 968.63 | 20.3 | 100 | ENSG00000050344 | NFE2L3 | Exon | 2 |
| EWS | 7 | 2.62E+07 | 2.62E+07 | 1155 | 26 | 85.75 | 6.4 | 63.3 | ENSG00000050344 | NFE2L3 | Intron | 2 |
| EWS | 1 | 7.63E+07 | 7.63E+07 | 2217 | 73 | 285.41 | 8.1 | 100 | ENSG00000057468 | MSH4 | Intron | 1 |
| Ac-H3K9 | 1 | 7.62E+07 | 7.63E+07 | 6074 | 709 | 3100 | 25.9 | 16.7 | ENSG00000057468 | MSH4 | Up | 1 |
| FUS | 1 | 7.63E+07 | 7.63E+07 | 1524 | 39 | 179.54 | 9.5 | 100 | ENSG00000057468 | MSH4 | Up | 1 |
| FUS | 2 | 8.58E+07 | 8.58E+07 | 1733 | 42 | 180.31 | 9.5 | 100 | ENSG00000115486 | GGCX | Down | 10 |
| EWS | 2 | 8.58E+07 | 8.58E+07 | 1036 | 21 | 87.99 | 8.0 | 63.7 | ENSG00000115486 | GGCX | Down | 10 |
| EWS | 2 | 8.58E+07 | 8.58E+07 | 1050 | 18 | 87.16 | 7.9 | 64.4 | ENSG00000115486 | GGCX | Down | 10 |
| Ac-H3K9 | 2 | 8.58E+07 | 8.58E+07 | 4623 | 297 | 1956.4 | 41.9 | 3.28 | ENSG00000115486 | GGCX | Down | 10 |
| Ac-H3K9 | 2 | 8.58E+07 | 8.58E+07 | 773 | 46 | 382.63 | 36.4 | 0.41 | ENSG00000115486 | GGCX | Up | 10 |
| EWS | 2 | 1.78E+08 | 1.78E+08 | 3253 | 92 | 154.27 | 7.3 | 100 | ENSG00000116044 | NFE2L2 | Down | 12 |
| FUS | 2 | 1.78E+08 | 1.78E+08 | 1785 | 33 | 154.27 | 8.0 | 100 | ENSG00000116044 | NFE2L2 | Down | 12 |
| Ac-H3K9 | 2 | 1.78E+08 | 1.78E+08 | 1125 | 50 | 358.32 | 24.2 | 0.43 | ENSG00000116044 | NFE2L2 | Exon | 12 |
| Ac-H3K9 | 2 | 1.78E+08 | 1.78E+08 | 1117 | 28 | 148.06 | 13.3 | 0.64 | ENSG00000116044 | NFE2L2 | Intron | 12 |
| Ac-H3K9 | 2 | 1.78E+08 | 1.78E+08 | 1150 | 45 | 138.22 | 9.8 | 0.67 | ENSG00000116044 | NFE2L2 | Intron | 12 |
| Ac-H3K9 | 2 | 1.78E+08 | 1.78E+08 | 1529 | 45 | 213.91 | 13.1 | 0.52 | ENSG00000116044 | NFE2L2 | Up | 12 |
| FUS | 1 | 7.63E+07 | 7.63E+07 | 1524 | 39 | 179.54 | 9.5 | 100 | ENSG00000117054 | ACADM | Down | 19 |
| EWS | 1 | 7.63E+07 | 7.63E+07 | 2217 | 73 | 285.41 | 8.1 | 100 | ENSG00000117054 | ACADM | Down | 19 |
| Ac-H3K9 | 1 | 7.62E+07 | 7.62E+07 | 3295 | 202 | 1169.9 | 26.3 | 1.04 | ENSG00000117054 | ACADM | Intron | 19 |
| Ac-H3K9 | 1 | 7.62E+07 | 7.63E+07 | 6074 | 709 | 3100 | 25.9 | 16.7 | ENSG00000117054 | ACADM | Intron | 19 |
| FUS | 1 | 1.74E+08 | 1.74E+08 | 1673 | 47 | 251.36 | 11.5 | 100 | ENSG00000117593 | DARS2 | Down | 4 |
| EWS | 1 | 1.74E+08 | 1.74E+08 | 1261 | 34 | 156.97 | 10.3 | 100 | ENSG00000117593 | DARS2 | Down | 4 |
| Ac-H3K9 | 1 | 1.74E+08 | 1.74E+08 | 1513 | 38 | 108.04 | 7.0 | 0.76 | ENSG00000117593 | DARS2 | Down | 4 |
| Ac-H3K9 | 1 | 1.74E+08 | 1.74E+08 | 2862 | 311 | 2345.4 | 29.1 | 6.25 | ENSG00000117593 | DARS2 | Down | 4 |
| Ac-H3K9 | 1 | 1.74E+08 | 1.74E+08 | 2506 | 184 | 1567.4 | 48.5 | 1.8 | ENSG00000117593 | DARS2 | Exon | 4 |
| FUS | 17 | 5.68E+07 | 5.68E+07 | 988 | 26 | 107.78 | 10.7 | 100 | ENSG00000121101 | TEX14 | Intron | 3 |
| EWS | 17 | 5.68E+07 | 5.68E+07 | 1103 | 35 | 156.68 | 13.6 | 100 | ENSG00000121101 | TEX14 | Intron | 3 |
| Ac-H3K9 | 17 | 5.68E+07 | 5.68E+07 | 504 | 29 | 159.49 | 19.2 | 0.62 | ENSG00000121101 | TEX14 | Intron | 3 |
| Ac-H3K9 | 17 | 5.67E+07 | 5.67E+07 | 924 | 37 | 202.7 | 20.4 | 0.53 | ENSG00000121101 | TEX14 | Intron | 3 |
| EWS | 17 | 5.67E+07 | 5.67E+07 | 810 | 17 | 116.76 | 13.3 | 93.5 | ENSG00000121101 | TEX14 | Intron | 3 |
| Ac-H3K9 | 17 | 5.67E+07 | 5.67E+07 | 1315 | 81 | 442.8 | 31.4 | 0.43 | ENSG00000121101 | TEX14 | Intron | 3 |
| Ac-H3K9 | 17 | 5.68E+07 | 5.68E+07 | 1905 | 101 | 672.68 | 33.1 | 0.59 | ENSG00000121101 | TEX14 | Up | 3 |
| FUS | 7 | 2.62E+07 | 2.62E+07 | 3011 | 95 | 504.94 | 15.2 | 100 | ENSG00000122566 | HNRNPA2B1 | Down | 8 |
| EWS | 7 | 2.62E+07 | 2.62E+07 | 4900 | 178 | 968.63 | 20.3 | 100 | ENSG00000122566 | HNRNPA2B1 | Down | 8 |
| FUS | 7 | 2.62E+07 | 2.62E+07 | 2147 | 51 | 201.43 | 9.2 | 100 | ENSG00000122566 | HNRNPA2B1 | Down | 8 |
| EWS | 7 | 2.62E+07 | 2.62E+07 | 742 | 24 | 131.86 | 9.2 | 100 | ENSG00000122566 | HNRNPA2B1 | Down | 8 |
| EWS | 7 | 2.62E+07 | 2.62E+07 | 1155 | 26 | 85.75 | 6.4 | 63.3 | ENSG00000122566 | HNRNPA2B1 | Down | 8 |
| Ac-H3K9 | 7 | 2.62E+07 | 2.62E+07 | 4637 | 359 | 1772 | 25.1 | 2.6 | ENSG00000122566 | HNRNPA2B1 | Exon | 8 |
| Ac-H3K9 | 7 | 2.62E+07 | 2.62E+07 | 2894 | 185 | 1325.6 | 40.1 | 1 | ENSG00000122566 | HNRNPA2B1 | Up | 8 |
| Ac-H3K9 | 6 | 7.42E+07 | 7.42E+07 | 2409 | 230 | 2086 | 46.3 | 3.92 | ENSG00000135297 | MTO1 | Down | 20 |
| EWS | 6 | 7.42E+07 | 7.42E+07 | 1322 | 34 | 108.22 | 8.8 | 84.1 | ENSG00000135297 | MTO1 | Down | 20 |
| FUS | 6 | 7.42E+07 | 7.42E+07 | 1387 | 32 | 107.03 | 7.1 | 100 | ENSG00000135297 | MTO1 | Down | 20 |
| Ac-H3K9 | 6 | 7.42E+07 | 7.42E+07 | 1095 | 66 | 534.57 | 35.3 | 0.49 | ENSG00000135297 | MTO1 | Exon | 20 |
| FUS | 8 | 1.29E+08 | 1.29E+08 | 1533 | 31 | 89.81 | 7.9 | 100 | ENSG00000136997 | MYC | Down | 6 |
| EWS | 8 | 1.29E+08 | 1.29E+08 | 1385 | 35 | 151.69 | 8.7 | 100 | ENSG00000136997 | MYC | Down | 6 |
| Ac-H3K9 | 8 | 1.29E+08 | 1.29E+08 | 665 | 17 | 106.01 | 18.7 | 0.76 | ENSG00000136997 | MYC | Exon | 6 |
| Ac-H3K9 | 8 | 1.29E+08 | 1.29E+08 | 847 | 28 | 183.67 | 22.5 | 0.54 | ENSG00000136997 | MYC | Intron | 6 |
| Ac-H3K9 | 8 | 1.29E+08 | 1.29E+08 | 735 | 17 | 85.81 | 16.9 | 0.8 | ENSG00000136997 | MYC | Intron | 6 |
| Ac-H3K9 | 8 | 1.29E+08 | 1.29E+08 | 631 | 24 | 177.97 | 25.3 | 0.55 | ENSG00000136997 | MYC | Up | 6 |
| Ac-H3K9 | 9 | 1.94E+07 | 1.94E+07 | 3850 | 351 | 2729 | 50.6 | 18.2 | ENSG00000137145 | DENND4C | Down | 10 |
| EWS | 9 | 1.94E+07 | 1.94E+07 | 1298 | 35 | 116.41 | 6.3 | 89.6 | ENSG00000137145 | DENND4C | Exon | 10 |
| FUS | 9 | 1.94E+07 | 1.94E+07 | 1232 | 38 | 148.76 | 9.4 | 100 | ENSG00000137145 | DENND4C | Exon | 10 |
| FUS | 9 | 1.94E+07 | 1.94E+07 | 1232 | 38 | 148.76 | 9.4 | 100 | ENSG00000137154 | RPS6 | Down | 5 |
| EWS | 9 | 1.94E+07 | 1.94E+07 | 1298 | 35 | 116.41 | 6.3 | 89.6 | ENSG00000137154 | RPS6 | Down | 5 |
| Ac-H3K9 | 9 | 1.94E+07 | 1.94E+07 | 3850 | 351 | 2729 | 50.6 | 18.2 | ENSG00000137154 | RPS6 | Exon | 5 |
| EWS | 1 | 7.63E+07 | 7.63E+07 | 2217 | 73 | 285.41 | 8.1 | 100 | ENSG00000137955 | RABGGTB | Down | 16 |
| FUS | 1 | 7.63E+07 | 7.63E+07 | 1524 | 39 | 179.54 | 9.5 | 100 | ENSG00000137955 | RABGGTB | Down | 16 |
| Ac-H3K9 | 1 | 7.62E+07 | 7.63E+07 | 6074 | 709 | 3100 | 25.9 | 16.7 | ENSG00000137955 | RABGGTB | Up | 16 |
| Ac-H3K9 | 19 | 5.13E+07 | 5.13E+07 | 2108 | 174 | 1759.9 | 70.2 | 2.47 | ENSG00000142513 | ACPT | Down | 2 |
| FUS | 19 | 5.13E+07 | 5.13E+07 | 1001 | 20 | 139.43 | 12.1 | 100 | ENSG00000142513 | ACPT | Exon | 2 |
| FUS | 19 | 5.13E+07 | 5.13E+07 | 837 | 14 | 90.31 | 12.1 | 100 | ENSG00000142513 | ACPT | Intron | 2 |
| EWS | 19 | 5.13E+07 | 5.13E+07 | 1774 | 45 | 266.73 | 20.0 | 100 | ENSG00000142513 | ACPT | Intron | 2 |
| Ac-H3K9 | 4 | 8.34E+07 | 8.34E+07 | 1569 | 47 | 233.49 | 15.0 | 0.5 | ENSG00000145293 | ENOPH1 | Intron | 4 |
| EWS | 4 | 8.33E+07 | 8.33E+07 | 1303 | 30 | 142.71 | 9.0 | 100 | ENSG00000145293 | ENOPH1 | Up | 4 |
| FUS | 4 | 8.33E+07 | 8.33E+07 | 1671 | 37 | 155.59 | 7.4 | 100 | ENSG00000145293 | ENOPH1 | Up | 4 |
| Ac-H3K9 | 4 | 8.33E+07 | 8.34E+07 | 2118 | 105 | 540.27 | 33.3 | 0.5 | ENSG00000145293 | ENOPH1 | Up | 4 |
| Ac-H3K9 | 4 | 8.34E+07 | 8.34E+07 | 790 | 49 | 367.39 | 37.6 | 0.41 | ENSG00000145293 | ENOPH1 | Up | 4 |
| FUS | 5 | 1.81E+08 | 1.81E+08 | 1656 | 49 | 202.06 | 10.4 | 100 | ENSG00000146063 | TRIM41 | Down | 11 |
| EWS | 5 | 1.81E+08 | 1.81E+08 | 2070 | 55 | 203.32 | 8.6 | 100 | ENSG00000146063 | TRIM41 | Down | 11 |
| Ac-H3K9 | 5 | 1.81E+08 | 1.81E+08 | 5156 | 281 | 1658.6 | 28.6 | 2.15 | ENSG00000146063 | TRIM41 | Down | 11 |
| Ac-H3K9 | 5 | 1.81E+08 | 1.81E+08 | 3088 | 266 | 2358.1 | 57.4 | 6.67 | ENSG00000146063 | TRIM41 | Down | 11 |
| Ac-H3K9 | 5 | 1.81E+08 | 1.81E+08 | 975 | 34 | 224.08 | 29.0 | 0.55 | ENSG00000146063 | TRIM41 | Exon | 11 |
| Ac-H3K9 | 5 | 1.81E+08 | 1.81E+08 | 647 | 17 | 101.52 | 15.3 | 0.76 | ENSG00000146063 | TRIM41 | Intron | 11 |
| Ac-H3K9 | 5 | 1.81E+08 | 1.81E+08 | 1238 | 33 | 179.27 | 14.9 | 0.55 | ENSG00000146063 | TRIM41 | Up | 11 |
| EWS | 4 | 8.33E+07 | 8.33E+07 | 1303 | 30 | 142.71 | 9.0 | 100 | ENSG00000152795 | HNRPDL | Down | 5 |
| Ac-H3K9 | 4 | 8.34E+07 | 8.34E+07 | 790 | 49 | 367.39 | 37.6 | 0.41 | ENSG00000152795 | HNRPDL | Exon | 5 |
| FUS | 4 | 8.33E+07 | 8.33E+07 | 1671 | 37 | 155.59 | 7.4 | 100 | ENSG00000152795 | HNRPDL | Exon | 5 |
| Ac-H3K9 | 4 | 8.33E+07 | 8.34E+07 | 2118 | 105 | 540.27 | 33.3 | 0.5 | ENSG00000152795 | HNRPDL | Intron | 5 |
| Ac-H3K9 | 4 | 8.34E+07 | 8.34E+07 | 1569 | 47 | 233.49 | 15.0 | 0.5 | ENSG00000152795 | HNRPDL | Up | 5 |
| FUS | 6 | 7.42E+07 | 7.42E+07 | 1387 | 32 | 107.03 | 7.1 | 100 | ENSG00000156508 | EEF1A1 | Down | 12 |
| EWS | 6 | 7.42E+07 | 7.42E+07 | 1322 | 34 | 108.22 | 8.8 | 84.1 | ENSG00000156508 | EEF1A1 | Down | 12 |
| Ac-H3K9 | 6 | 7.42E+07 | 7.42E+07 | 1334 | 38 | 198.42 | 13.5 | 0.52 | ENSG00000156508 | EEF1A1 | Exon | 12 |
| Ac-H3K9 | 6 | 7.42E+07 | 7.42E+07 | 2409 | 230 | 2086 | 46.3 | 3.92 | ENSG00000156508 | EEF1A1 | Intron | 12 |
| Ac-H3K9 | 6 | 7.42E+07 | 7.42E+07 | 962 | 37 | 265.44 | 25.3 | 0.52 | ENSG00000156508 | EEF1A1 | Up | 12 |
| FUS | 3 | 1.87E+08 | 1.87E+08 | 596 | 14 | 91.11 | 12.5 | 100 | ENSG00000156976 | EIF4A2 | Down | 28 |
| EWS | 3 | 1.87E+08 | 1.87E+08 | 980 | 27 | 180.95 | 17.6 | 100 | ENSG00000156976 | EIF4A2 | Down | 28 |
| Ac-H3K9 | 3 | 1.86E+08 | 1.87E+08 | 4178 | 296 | 2539.6 | 42.2 | 9.09 | ENSG00000156976 | EIF4A2 | Exon | 28 |
| Ac-H3K9 | 3 | 1.86E+08 | 1.87E+08 | 4178 | 296 | 2539.6 | 42.2 | 9.09 | ENSG00000163918 | RFC4 | Down | 14 |
| FUS | 3 | 1.87E+08 | 1.87E+08 | 596 | 14 | 91.11 | 12.5 | 100 | ENSG00000163918 | RFC4 | Exon | 14 |
| EWS | 3 | 1.87E+08 | 1.87E+08 | 980 | 27 | 180.95 | 17.6 | 100 | ENSG00000163918 | RFC4 | Intron | 14 |
| Ac-H3K9 | 3 | 1.87E+08 | 1.87E+08 | 1965 | 102 | 846 | 37.4 | 0.42 | ENSG00000163918 | RFC4 | Intron | 14 |
| Ac-H3K9 | 9 | 8.66E+07 | 8.66E+07 | 1307 | 39 | 229.99 | 20.5 | 0.51 | ENSG00000165118 | C9orf64 | Exon | 3 |
| FUS | 9 | 8.66E+07 | 8.66E+07 | 722 | 16 | 91.77 | 9.5 | 100 | ENSG00000165118 | C9orf64 | Up | 3 |
| EWS | 9 | 8.66E+07 | 8.66E+07 | 930 | 22 | 120.23 | 13.1 | 100 | ENSG00000165118 | C9orf64 | Up | 3 |
| FUS | 9 | 8.66E+07 | 8.66E+07 | 722 | 16 | 91.77 | 9.5 | 100 | ENSG00000165119 | HNRNPK | Down | 15 |
| EWS | 9 | 8.66E+07 | 8.66E+07 | 930 | 22 | 120.23 | 13.1 | 100 | ENSG00000165119 | HNRNPK | Down | 15 |
| Ac-H3K9 | 9 | 8.66E+07 | 8.66E+07 | 4715 | 280 | 2126.3 | 55.6 | 4.17 | ENSG00000165119 | HNRNPK | Intron | 15 |
| FUS | 19 | 5.13E+07 | 5.13E+07 | 1001 | 20 | 139.43 | 12.1 | 100 | ENSG00000167747 | C19orf48 | Down | 2 |
| FUS | 19 | 5.13E+07 | 5.13E+07 | 837 | 14 | 90.31 | 12.1 | 100 | ENSG00000167747 | C19orf48 | Down | 2 |
| EWS | 19 | 5.13E+07 | 5.13E+07 | 1774 | 45 | 266.73 | 20.0 | 100 | ENSG00000167747 | C19orf48 | Down | 2 |
| Ac-H3K9 | 19 | 5.13E+07 | 5.13E+07 | 2108 | 174 | 1759.9 | 70.2 | 2.47 | ENSG00000167747 | C19orf48 | Intron | 2 |
| EWS | 2 | 8.58E+07 | 8.58E+07 | 1050 | 18 | 87.16 | 7.9 | 64.4 | ENSG00000168906 | MAT2A | Down | 7 |
| FUS | 2 | 8.58E+07 | 8.58E+07 | 1733 | 42 | 180.31 | 9.5 | 100 | ENSG00000168906 | MAT2A | Down | 7 |
| EWS | 2 | 8.58E+07 | 8.58E+07 | 1036 | 21 | 87.99 | 8.0 | 63.7 | ENSG00000168906 | MAT2A | Down | 7 |
| Ac-H3K9 | 2 | 8.58E+07 | 8.58E+07 | 4623 | 297 | 1956.4 | 41.9 | 3.28 | ENSG00000168906 | MAT2A | Up | 7 |
| FUS | 2 | 1.78E+08 | 1.78E+08 | 1785 | 33 | 154.27 | 8.0 | 100 | ENSG00000170144 | HNRNPA3 | Down | 8 |
| EWS | 2 | 1.78E+08 | 1.78E+08 | 3253 | 92 | 154.27 | 7.3 | 100 | ENSG00000170144 | HNRNPA3 | Down | 8 |
| Ac-H3K9 | 2 | 1.78E+08 | 1.78E+08 | 529 | 19 | 115 | 17.0 | 0.75 | ENSG00000170144 | HNRNPA3 | Intron | 8 |
| Ac-H3K9 | 2 | 1.78E+08 | 1.78E+08 | 1403 | 136 | 775.67 | 25.3 | 0.34 | ENSG00000170144 | HNRNPA3 | Intron | 8 |
| Ac-H3K9 | 2 | 1.78E+08 | 1.78E+08 | 1555 | 152 | 956.7 | 19.0 | 0.59 | ENSG00000170144 | HNRNPA3 | Up | 8 |
| FUS | 1 | 2.88E+07 | 2.88E+07 | 1523 | 77 | 409.11 | 15.0 | 100 | ENSG00000180198 | RCC1 | Intron | 12 |
| EWS | 1 | 2.88E+07 | 2.88E+07 | 2270 | 110 | 495.44 | 17.0 | 100 | ENSG00000180198 | RCC1 | Intron | 12 |
| EWS | 1 | 2.88E+07 | 2.88E+07 | 1371 | 38 | 132.6 | 8.7 | 100 | ENSG00000180198 | RCC1 | Intron | 12 |
| FUS | 1 | 2.88E+07 | 2.88E+07 | 1267 | 36 | 150.89 | 7.1 | 100 | ENSG00000180198 | RCC1 | Intron | 12 |
| Ac-H3K9 | 1 | 2.88E+07 | 2.88E+07 | 1651 | 90 | 492.75 | 24.5 | 0.47 | ENSG00000180198 | RCC1 | Intron | 12 |
| Ac-H3K9 | 1 | 2.88E+07 | 2.88E+07 | 6730 | 807 | 3100 | 40.7 | 16.7 | ENSG00000180198 | RCC1 | Up | 12 |
| Ac-H3K9 | 1 | 1.74E+08 | 1.74E+08 | 716 | 23 | 119.88 | 17.2 | 0.73 | ENSG00000185278 | ZBTB37 | Intron | 7 |
| FUS | 1 | 1.74E+08 | 1.74E+08 | 1673 | 47 | 251.36 | 11.5 | 100 | ENSG00000185278 | ZBTB37 | Up | 7 |
| EWS | 1 | 1.74E+08 | 1.74E+08 | 1261 | 34 | 156.97 | 10.3 | 100 | ENSG00000185278 | ZBTB37 | Up | 7 |
| Ac-H3K9 | 1 | 1.74E+08 | 1.74E+08 | 1513 | 38 | 108.04 | 7.0 | 0.76 | ENSG00000185278 | ZBTB37 | Up | 7 |
| Ac-H3K9 | 1 | 1.74E+08 | 1.74E+08 | 2862 | 311 | 2345.4 | 29.1 | 6.25 | ENSG00000185278 | ZBTB37 | Up | 7 |
| FUS | 5 | 1.77E+08 | 1.77E+08 | 661 | 15 | 84.47 | 12.1 | 100 | ENSG00000198055 | GRK6 | Down | 11 |
| EWS | 5 | 1.77E+08 | 1.77E+08 | 1116 | 32 | 209.84 | 20.0 | 100 | ENSG00000198055 | GRK6 | Down | 11 |
| Ac-H3K9 | 5 | 1.77E+08 | 1.77E+08 | 1472 | 80 | 686.59 | 35.5 | 0.53 | ENSG00000198055 | GRK6 | Intron | 11 |
| Ac-H3K9 | 13 | 9.20E+07 | 9.20E+07 | 1257 | 30 | 89.04 | 6.5 | 0.77 | ENSG00000199149 | MIR20A | Down | 1 |
| EWS | 13 | 9.20E+07 | 9.20E+07 | 5252 | 144 | 294.99 | 6.4 | 100 | ENSG00000199149 | MIR20A | Down | 1 |
| FUS | 13 | 9.20E+07 | 9.20E+07 | 2989 | 72 | 179.39 | 6.7 | 100 | ENSG00000199149 | MIR20A | Down | 1 |
| FUS | 13 | 9.20E+07 | 9.20E+07 | 1954 | 55 | 125.65 | 6.7 | 100 | ENSG00000199149 | MIR20A | Down | 1 |
| FUS | 13 | 9.20E+07 | 9.20E+07 | 2877 | 82 | 211.85 | 6.4 | 100 | ENSG00000199149 | MIR20A | Down | 1 |
| Ac-H3K9 | 13 | 9.20E+07 | 9.20E+07 | 1574 | 81 | 398.62 | 17.5 | 0.36 | ENSG00000199149 | MIR20A | Up | 1 |
| Ac-H3K9 | 13 | 9.20E+07 | 9.20E+07 | 1108 | 31 | 94.77 | 9.8 | 0.78 | ENSG00000199149 | MIR20A | Up | 1 |
| Ac-H3K9 | 13 | 9.20E+07 | 9.20E+07 | 1680 | 286 | 2213.6 | 36.8 | 4.88 | ENSG00000199149 | MIR20A | Up | 1 |
| EWS | 13 | 9.20E+07 | 9.20E+07 | 5252 | 144 | 294.99 | 6.4 | 100 | ENSG00000199180 | MIR18A | Down | 1 |
| FUS | 13 | 9.20E+07 | 9.20E+07 | 2989 | 72 | 179.39 | 6.7 | 100 | ENSG00000199180 | MIR18A | Down | 1 |
| Ac-H3K9 | 13 | 9.20E+07 | 9.20E+07 | 1257 | 30 | 89.04 | 6.5 | 0.77 | ENSG00000199180 | MIR18A | Down | 1 |
| FUS | 13 | 9.20E+07 | 9.20E+07 | 1954 | 55 | 125.65 | 6.7 | 100 | ENSG00000199180 | MIR18A | Down | 1 |
| FUS | 13 | 9.20E+07 | 9.20E+07 | 2877 | 82 | 211.85 | 6.4 | 100 | ENSG00000199180 | MIR18A | Down | 1 |
| Ac-H3K9 | 13 | 9.20E+07 | 9.20E+07 | 1574 | 81 | 398.62 | 17.5 | 0.36 | ENSG00000199180 | MIR18A | Up | 1 |
| Ac-H3K9 | 13 | 9.20E+07 | 9.20E+07 | 1108 | 31 | 94.77 | 9.8 | 0.78 | ENSG00000199180 | MIR18A | Up | 1 |
| Ac-H3K9 | 13 | 9.20E+07 | 9.20E+07 | 1680 | 286 | 2213.6 | 36.8 | 4.88 | ENSG00000199180 | MIR18A | Up | 1 |
| Ac-H3K9 | 1 | 1.74E+08 | 1.74E+08 | 1513 | 38 | 108.04 | 7.0 | 0.76 | ENSG00000200016 | SNORD76 | Down | 1 |
| EWS | 1 | 1.74E+08 | 1.74E+08 | 1261 | 34 | 156.97 | 10.3 | 100 | ENSG00000200016 | SNORD76 | Down | 1 |
| FUS | 1 | 1.74E+08 | 1.74E+08 | 1673 | 47 | 251.36 | 11.5 | 100 | ENSG00000200016 | SNORD76 | Down | 1 |
| Ac-H3K9 | 1 | 1.74E+08 | 1.74E+08 | 716 | 23 | 119.88 | 17.2 | 0.73 | ENSG00000200016 | SNORD76 | Up | 1 |
| Ac-H3K9 | 1 | 1.74E+08 | 1.74E+08 | 2862 | 311 | 2345.4 | 29.1 | 6.25 | ENSG00000200016 | SNORD76 | Up | 1 |
| Ac-H3K9 | 1 | 2.88E+07 | 2.88E+07 | 1651 | 90 | 492.75 | 24.5 | 0.47 | ENSG00000200087 | SNORA73B | Down | 1 |
| FUS | 1 | 2.88E+07 | 2.88E+07 | 1523 | 77 | 409.11 | 15.0 | 100 | ENSG00000200087 | SNORA73B | Down | 1 |
| EWS | 1 | 2.88E+07 | 2.88E+07 | 2270 | 110 | 495.44 | 17.0 | 100 | ENSG00000200087 | SNORA73B | Down | 1 |
| FUS | 1 | 2.88E+07 | 2.88E+07 | 1267 | 36 | 150.89 | 7.1 | 100 | ENSG00000200087 | SNORA73B | Down | 1 |
| EWS | 1 | 2.88E+07 | 2.88E+07 | 1371 | 38 | 132.6 | 8.7 | 100 | ENSG00000200087 | SNORA73B | Down | 1 |
| Ac-H3K9 | 1 | 2.88E+07 | 2.88E+07 | 6730 | 807 | 3100 | 40.7 | 16.7 | ENSG00000200087 | SNORA73B | Up | 1 |
| FUS | 3 | 1.87E+08 | 1.87E+08 | 596 | 14 | 91.11 | 12.5 | 100 | ENSG00000200320 | SNORA63 | Down | 1 |
| EWS | 3 | 1.87E+08 | 1.87E+08 | 980 | 27 | 180.95 | 17.6 | 100 | ENSG00000200320 | SNORA63 | Down | 1 |
| Ac-H3K9 | 3 | 1.86E+08 | 1.87E+08 | 4178 | 296 | 2539.6 | 42.2 | 9.09 | ENSG00000200320 | SNORA63 | Up | 1 |
| FUS | 3 | 1.87E+08 | 1.87E+08 | 596 | 14 | 91.11 | 12.5 | 100 | ENSG00000200418 | SNORA63.6 | Down | 1 |
| EWS | 3 | 1.87E+08 | 1.87E+08 | 980 | 27 | 180.95 | 17.6 | 100 | ENSG00000200418 | SNORA63.6 | Down | 1 |
| Ac-H3K9 | 3 | 1.86E+08 | 1.87E+08 | 4178 | 296 | 2539.6 | 42.2 | 9.09 | ENSG00000200418 | SNORA63.6 | Up | 1 |
| EWS | 1 | 1.74E+08 | 1.74E+08 | 1261 | 34 | 156.97 | 10.3 | 100 | ENSG00000200710 | SNORD81 | Down | 1 |
| FUS | 1 | 1.74E+08 | 1.74E+08 | 1673 | 47 | 251.36 | 11.5 | 100 | ENSG00000200710 | SNORD81 | Down | 1 |
| Ac-H3K9 | 1 | 1.74E+08 | 1.74E+08 | 1513 | 38 | 108.04 | 7.0 | 0.76 | ENSG00000200710 | SNORD81 | Up | 1 |
| Ac-H3K9 | 1 | 1.74E+08 | 1.74E+08 | 716 | 23 | 119.88 | 17.2 | 0.73 | ENSG00000200710 | SNORD81 | Up | 1 |
| Ac-H3K9 | 1 | 1.74E+08 | 1.74E+08 | 2862 | 311 | 2345.4 | 29.1 | 6.25 | ENSG00000200710 | SNORD81 | Up | 1 |
| Ac-H3K9 | 1 | 1.74E+08 | 1.74E+08 | 1513 | 38 | 108.04 | 7.0 | 0.76 | ENSG00000200729 | SNORD79 | Down | 1 |
| EWS | 1 | 1.74E+08 | 1.74E+08 | 1261 | 34 | 156.97 | 10.3 | 100 | ENSG00000200729 | SNORD79 | Down | 1 |
| FUS | 1 | 1.74E+08 | 1.74E+08 | 1673 | 47 | 251.36 | 11.5 | 100 | ENSG00000200729 | SNORD79 | Down | 1 |
| Ac-H3K9 | 1 | 1.74E+08 | 1.74E+08 | 716 | 23 | 119.88 | 17.2 | 0.73 | ENSG00000200729 | SNORD79 | Up | 1 |
| Ac-H3K9 | 1 | 1.74E+08 | 1.74E+08 | 2862 | 311 | 2345.4 | 29.1 | 6.25 | ENSG00000200729 | SNORD79 | Up | 1 |
| Ac-H3K9 | 1 | 1.74E+08 | 1.74E+08 | 1513 | 38 | 108.04 | 7.0 | 0.76 | ENSG00000200954 | SNORD74 | Down | 1 |
| Ac-H3K9 | 1 | 1.74E+08 | 1.74E+08 | 2862 | 311 | 2345.4 | 29.1 | 6.25 | ENSG00000200954 | SNORD74 | Down | 1 |
| EWS | 1 | 1.74E+08 | 1.74E+08 | 1261 | 34 | 156.97 | 10.3 | 100 | ENSG00000200954 | SNORD74 | Down | 1 |
| FUS | 1 | 1.74E+08 | 1.74E+08 | 1673 | 47 | 251.36 | 11.5 | 100 | ENSG00000200954 | SNORD74 | Down | 1 |
| Ac-H3K9 | 1 | 1.74E+08 | 1.74E+08 | 716 | 23 | 119.88 | 17.2 | 0.73 | ENSG00000200954 | SNORD74 | Up | 1 |
| FUS | 17 | 5.68E+07 | 5.68E+07 | 988 | 26 | 107.78 | 10.7 | 100 | ENSG00000200997 | U1.36 | Down | 1 |
| EWS | 17 | 5.68E+07 | 5.68E+07 | 1103 | 35 | 156.68 | 13.6 | 100 | ENSG00000200997 | U1.36 | Down | 1 |
| Ac-H3K9 | 17 | 5.68E+07 | 5.68E+07 | 504 | 29 | 159.49 | 19.2 | 0.62 | ENSG00000200997 | U1.36 | Up | 1 |
| FUS | 1 | 7.63E+07 | 7.63E+07 | 1524 | 39 | 179.54 | 9.5 | 100 | ENSG00000201487 | SNORD45B | Down | 1 |
| EWS | 1 | 7.63E+07 | 7.63E+07 | 2217 | 73 | 285.41 | 8.1 | 100 | ENSG00000201487 | SNORD45B | Down | 1 |
| Ac-H3K9 | 1 | 7.62E+07 | 7.63E+07 | 6074 | 709 | 3100 | 25.9 | 16.7 | ENSG00000201487 | SNORD45B | Up | 1 |
| EWS | 1 | 1.74E+08 | 1.74E+08 | 1261 | 34 | 156.97 | 10.3 | 100 | ENSG00000201692 | SNORD80 | Down | 1 |
| Ac-H3K9 | 1 | 1.74E+08 | 1.74E+08 | 1513 | 38 | 108.04 | 7.0 | 0.76 | ENSG00000201692 | SNORD80 | Down | 1 |
| FUS | 1 | 1.74E+08 | 1.74E+08 | 1673 | 47 | 251.36 | 11.5 | 100 | ENSG00000201692 | SNORD80 | Down | 1 |
| Ac-H3K9 | 1 | 1.74E+08 | 1.74E+08 | 716 | 23 | 119.88 | 17.2 | 0.73 | ENSG00000201692 | SNORD80 | Up | 1 |
| Ac-H3K9 | 1 | 1.74E+08 | 1.74E+08 | 2862 | 311 | 2345.4 | 29.1 | 6.25 | ENSG00000201692 | SNORD80 | Up | 1 |
| EWS | 1 | 2.88E+07 | 2.88E+07 | 2270 | 110 | 495.44 | 17.0 | 100 | ENSG00000201808 | SNORA73A | Down | 1 |
| FUS | 1 | 2.88E+07 | 2.88E+07 | 1523 | 77 | 409.11 | 15.0 | 100 | ENSG00000201808 | SNORA73A | Down | 1 |
| EWS | 1 | 2.88E+07 | 2.88E+07 | 1371 | 38 | 132.6 | 8.7 | 100 | ENSG00000201808 | SNORA73A | Down | 1 |
| FUS | 1 | 2.88E+07 | 2.88E+07 | 1267 | 36 | 150.89 | 7.1 | 100 | ENSG00000201808 | SNORA73A | Down | 1 |
| Ac-H3K9 | 1 | 2.88E+07 | 2.88E+07 | 6730 | 807 | 3100 | 40.7 | 16.7 | ENSG00000201808 | SNORA73A | Up | 1 |
| EWS | 1 | 1.74E+08 | 1.74E+08 | 1261 | 34 | 156.97 | 10.3 | 100 | ENSG00000202394 | SNORD47 | Down | 1 |
| FUS | 1 | 1.74E+08 | 1.74E+08 | 1673 | 47 | 251.36 | 11.5 | 100 | ENSG00000202394 | SNORD47 | Down | 1 |
| Ac-H3K9 | 1 | 1.74E+08 | 1.74E+08 | 1513 | 38 | 108.04 | 7.0 | 0.76 | ENSG00000202394 | SNORD47 | Up | 1 |
| Ac-H3K9 | 1 | 1.74E+08 | 1.74E+08 | 716 | 23 | 119.88 | 17.2 | 0.73 | ENSG00000202394 | SNORD47 | Up | 1 |
| Ac-H3K9 | 1 | 1.74E+08 | 1.74E+08 | 2862 | 311 | 2345.4 | 29.1 | 6.25 | ENSG00000202394 | SNORD47 | Up | 1 |
| FUS | 1 | 2.88E+07 | 2.88E+07 | 1523 | 77 | 409.11 | 15.0 | 100 | ENSG00000204138 | PHACTR4 | Down | 5 |
| EWS | 1 | 2.88E+07 | 2.88E+07 | 2270 | 110 | 495.44 | 17.0 | 100 | ENSG00000204138 | PHACTR4 | Down | 5 |
| Ac-H3K9 | 1 | 2.88E+07 | 2.88E+07 | 6730 | 807 | 3100 | 40.7 | 16.7 | ENSG00000204138 | PHACTR4 | Down | 5 |
| Ac-H3K9 | 1 | 2.87E+07 | 2.87E+07 | 1256 | 70 | 319.85 | 15.7 | 0.49 | ENSG00000204138 | PHACTR4 | Intron | 5 |
| FUS | 6 | 3.29E+07 | 3.30E+07 | 1829 | 50 | 226.58 | 11.6 | 100 | ENSG00000204256 | BRD2 | Down | 16 |
| EWS | 6 | 3.29E+07 | 3.30E+07 | 1473 | 42 | 211.43 | 12.9 | 100 | ENSG00000204256 | BRD2 | Down | 16 |
| Ac-H3K9 | 6 | 3.29E+07 | 3.29E+07 | 3378 | 243 | 1977.6 | 41.3 | 3.45 | ENSG00000204256 | BRD2 | Intron | 16 |
| Ac-H3K9 | 6 | 3.29E+07 | 3.29E+07 | 4124 | 230 | 1585.6 | 35.4 | 1.89 | ENSG00000204256 | BRD2 | Intron | 16 |
| Ac-H3K9 | 6 | 3.29E+07 | 3.29E+07 | 662 | 32 | 262.59 | 28.9 | 0.51 | ENSG00000204256 | BRD2 | Up | 16 |
| FUS | 5 | 1.81E+08 | 1.81E+08 | 1656 | 49 | 202.06 | 10.4 | 100 | ENSG00000204628 | GNB2L1 | Down | 35 |
| EWS | 5 | 1.81E+08 | 1.81E+08 | 2070 | 55 | 203.32 | 8.6 | 100 | ENSG00000204628 | GNB2L1 | Down | 35 |
| Ac-H3K9 | 5 | 1.81E+08 | 1.81E+08 | 3088 | 266 | 2358.1 | 57.4 | 6.67 | ENSG00000204628 | GNB2L1 | Exon | 35 |
| Ac-H3K9 | 5 | 1.81E+08 | 1.81E+08 | 5156 | 281 | 1658.6 | 28.6 | 2.15 | ENSG00000204628 | GNB2L1 | Intron | 35 |
| Ac-H3K9 | 1 | 1.74E+08 | 1.74E+08 | 1513 | 38 | 108.04 | 7.0 | 0.76 | ENSG00000206607 | SNORD44 | Down | 1 |
| EWS | 1 | 1.74E+08 | 1.74E+08 | 1261 | 34 | 156.97 | 10.3 | 100 | ENSG00000206607 | SNORD44 | Down | 1 |
| FUS | 1 | 1.74E+08 | 1.74E+08 | 1673 | 47 | 251.36 | 11.5 | 100 | ENSG00000206607 | SNORD44 | Down | 1 |
| Ac-H3K9 | 1 | 1.74E+08 | 1.74E+08 | 716 | 23 | 119.88 | 17.2 | 0.73 | ENSG00000206607 | SNORD44 | Up | 1 |
| Ac-H3K9 | 1 | 1.74E+08 | 1.74E+08 | 2862 | 311 | 2345.4 | 29.1 | 6.25 | ENSG00000206607 | SNORD44 | Up | 1 |
| EWS | 1 | 7.63E+07 | 7.63E+07 | 2217 | 73 | 285.41 | 8.1 | 100 | ENSG00000206620 | SNORD45C | Down | 1 |
| FUS | 1 | 7.63E+07 | 7.63E+07 | 1524 | 39 | 179.54 | 9.5 | 100 | ENSG00000206620 | SNORD45C | Down | 1 |
| Ac-H3K9 | 1 | 7.62E+07 | 7.63E+07 | 6074 | 709 | 3100 | 25.9 | 16.7 | ENSG00000206620 | SNORD45C | Up | 1 |
| Ac-H3K9 | 17 | 5.68E+07 | 5.68E+07 | 504 | 29 | 159.49 | 19.2 | 0.62 | ENSG00000206917 | U1.78 | Up | 1 |
| FUS | 17 | 5.68E+07 | 5.68E+07 | 988 | 26 | 107.78 | 10.7 | 100 | ENSG00000206917 | U1.78 | Up | 1 |
| EWS | 17 | 5.68E+07 | 5.68E+07 | 1103 | 35 | 156.68 | 13.6 | 100 | ENSG00000206917 | U1.78 | Up | 1 |
| FUS | 1 | 7.63E+07 | 7.63E+07 | 1524 | 39 | 179.54 | 9.5 | 100 | ENSG00000207241 | SNORD45A | Down | 1 |
| EWS | 1 | 7.63E+07 | 7.63E+07 | 2217 | 73 | 285.41 | 8.1 | 100 | ENSG00000207241 | SNORD45A | Down | 1 |
| Ac-H3K9 | 1 | 7.62E+07 | 7.63E+07 | 6074 | 709 | 3100 | 25.9 | 16.7 | ENSG00000207241 | SNORD45A | Up | 1 |
| Ac-H3K9 | 13 | 9.20E+07 | 9.20E+07 | 1257 | 30 | 89.04 | 6.5 | 0.77 | ENSG00000207560 | MIR19B1 | Down | 1 |
| EWS | 13 | 9.20E+07 | 9.20E+07 | 5252 | 144 | 294.99 | 6.4 | 100 | ENSG00000207560 | MIR19B1 | Down | 1 |
| FUS | 13 | 9.20E+07 | 9.20E+07 | 2989 | 72 | 179.39 | 6.7 | 100 | ENSG00000207560 | MIR19B1 | Down | 1 |
| FUS | 13 | 9.20E+07 | 9.20E+07 | 1954 | 55 | 125.65 | 6.7 | 100 | ENSG00000207560 | MIR19B1 | Down | 1 |
| FUS | 13 | 9.20E+07 | 9.20E+07 | 2877 | 82 | 211.85 | 6.4 | 100 | ENSG00000207560 | MIR19B1 | Down | 1 |
| Ac-H3K9 | 13 | 9.20E+07 | 9.20E+07 | 1574 | 81 | 398.62 | 17.5 | 0.36 | ENSG00000207560 | MIR19B1 | Up | 1 |
| Ac-H3K9 | 13 | 9.20E+07 | 9.20E+07 | 1108 | 31 | 94.77 | 9.8 | 0.78 | ENSG00000207560 | MIR19B1 | Up | 1 |
| Ac-H3K9 | 13 | 9.20E+07 | 9.20E+07 | 1680 | 286 | 2213.6 | 36.8 | 4.88 | ENSG00000207560 | MIR19B1 | Up | 1 |
| FUS | 9 | 8.66E+07 | 8.66E+07 | 722 | 16 | 91.77 | 9.5 | 100 | ENSG00000207603 | MIR7-1 | Down | 1 |
| EWS | 9 | 8.66E+07 | 8.66E+07 | 930 | 22 | 120.23 | 13.1 | 100 | ENSG00000207603 | MIR7-1 | Down | 1 |
| Ac-H3K9 | 9 | 8.66E+07 | 8.66E+07 | 4715 | 280 | 2126.3 | 55.6 | 4.17 | ENSG00000207603 | MIR7-1 | Up | 1 |
| EWS | 13 | 9.20E+07 | 9.20E+07 | 5252 | 144 | 294.99 | 6.4 | 100 | ENSG00000207610 | MIR19A | Down | 1 |
| FUS | 13 | 9.20E+07 | 9.20E+07 | 2989 | 72 | 179.39 | 6.7 | 100 | ENSG00000207610 | MIR19A | Down | 1 |
| Ac-H3K9 | 13 | 9.20E+07 | 9.20E+07 | 1257 | 30 | 89.04 | 6.5 | 0.77 | ENSG00000207610 | MIR19A | Down | 1 |
| FUS | 13 | 9.20E+07 | 9.20E+07 | 1954 | 55 | 125.65 | 6.7 | 100 | ENSG00000207610 | MIR19A | Down | 1 |
| FUS | 13 | 9.20E+07 | 9.20E+07 | 2877 | 82 | 211.85 | 6.4 | 100 | ENSG00000207610 | MIR19A | Down | 1 |
| Ac-H3K9 | 13 | 9.20E+07 | 9.20E+07 | 1574 | 81 | 398.62 | 17.5 | 0.36 | ENSG00000207610 | MIR19A | Up | 1 |
| Ac-H3K9 | 13 | 9.20E+07 | 9.20E+07 | 1108 | 31 | 94.77 | 9.8 | 0.78 | ENSG00000207610 | MIR19A | Up | 1 |
| Ac-H3K9 | 13 | 9.20E+07 | 9.20E+07 | 1680 | 286 | 2213.6 | 36.8 | 4.88 | ENSG00000207610 | MIR19A | Up | 1 |
| FUS | 13 | 9.20E+07 | 9.20E+07 | 2877 | 82 | 211.85 | 6.4 | 100 | ENSG00000207745 | MIR17 | Down | 1 |
| EWS | 13 | 9.20E+07 | 9.20E+07 | 5252 | 144 | 294.99 | 6.4 | 100 | ENSG00000207745 | MIR17 | Down | 1 |
| Ac-H3K9 | 13 | 9.20E+07 | 9.20E+07 | 1257 | 30 | 89.04 | 6.5 | 0.77 | ENSG00000207745 | MIR17 | Down | 1 |
| FUS | 13 | 9.20E+07 | 9.20E+07 | 2989 | 72 | 179.39 | 6.7 | 100 | ENSG00000207745 | MIR17 | Down | 1 |
| FUS | 13 | 9.20E+07 | 9.20E+07 | 1954 | 55 | 125.65 | 6.7 | 100 | ENSG00000207745 | MIR17 | Down | 1 |
| Ac-H3K9 | 13 | 9.20E+07 | 9.20E+07 | 1574 | 81 | 398.62 | 17.5 | 0.36 | ENSG00000207745 | MIR17 | Up | 1 |
| Ac-H3K9 | 13 | 9.20E+07 | 9.20E+07 | 1108 | 31 | 94.77 | 9.8 | 0.78 | ENSG00000207745 | MIR17 | Up | 1 |
| Ac-H3K9 | 13 | 9.20E+07 | 9.20E+07 | 1680 | 286 | 2213.6 | 36.8 | 4.88 | ENSG00000207745 | MIR17 | Up | 1 |
| Ac-H3K9 | 13 | 9.20E+07 | 9.20E+07 | 1257 | 30 | 89.04 | 6.5 | 0.77 | ENSG00000207968 | MIR92A1 | Down | 1 |
| EWS | 13 | 9.20E+07 | 9.20E+07 | 5252 | 144 | 294.99 | 6.4 | 100 | ENSG00000207968 | MIR92A1 | Down | 1 |
| FUS | 13 | 9.20E+07 | 9.20E+07 | 2989 | 72 | 179.39 | 6.7 | 100 | ENSG00000207968 | MIR92A1 | Down | 1 |
| FUS | 13 | 9.20E+07 | 9.20E+07 | 1954 | 55 | 125.65 | 6.7 | 100 | ENSG00000207968 | MIR92A1 | Down | 1 |
| FUS | 13 | 9.20E+07 | 9.20E+07 | 2877 | 82 | 211.85 | 6.4 | 100 | ENSG00000207968 | MIR92A1 | Down | 1 |
| Ac-H3K9 | 13 | 9.20E+07 | 9.20E+07 | 1574 | 81 | 398.62 | 17.5 | 0.36 | ENSG00000207968 | MIR92A1 | Up | 1 |
| Ac-H3K9 | 13 | 9.20E+07 | 9.20E+07 | 1108 | 31 | 94.77 | 9.8 | 0.78 | ENSG00000207968 | MIR92A1 | Up | 1 |
| Ac-H3K9 | 13 | 9.20E+07 | 9.20E+07 | 1680 | 286 | 2213.6 | 36.8 | 4.88 | ENSG00000207968 | MIR92A1 | Up | 1 |
| Ac-H3K9 | 1 | 1.74E+08 | 1.74E+08 | 1513 | 38 | 108.04 | 7.0 | 0.76 | ENSG00000208310 | SNORD75 | Down | 1 |
| EWS | 1 | 1.74E+08 | 1.74E+08 | 1261 | 34 | 156.97 | 10.3 | 100 | ENSG00000208310 | SNORD75 | Down | 1 |
| FUS | 1 | 1.74E+08 | 1.74E+08 | 1673 | 47 | 251.36 | 11.5 | 100 | ENSG00000208310 | SNORD75 | Down | 1 |
| Ac-H3K9 | 1 | 1.74E+08 | 1.74E+08 | 716 | 23 | 119.88 | 17.2 | 0.73 | ENSG00000208310 | SNORD75 | Up | 1 |
| Ac-H3K9 | 1 | 1.74E+08 | 1.74E+08 | 2862 | 311 | 2345.4 | 29.1 | 6.25 | ENSG00000208310 | SNORD75 | Up | 1 |
| Ac-H3K9 | 1 | 1.74E+08 | 1.74E+08 | 1513 | 38 | 108.04 | 7.0 | 0.76 | ENSG00000208313 | SNORD77 | Down | 1 |
| EWS | 1 | 1.74E+08 | 1.74E+08 | 1261 | 34 | 156.97 | 10.3 | 100 | ENSG00000208313 | SNORD77 | Down | 1 |
| FUS | 1 | 1.74E+08 | 1.74E+08 | 1673 | 47 | 251.36 | 11.5 | 100 | ENSG00000208313 | SNORD77 | Down | 1 |
| Ac-H3K9 | 1 | 1.74E+08 | 1.74E+08 | 716 | 23 | 119.88 | 17.2 | 0.73 | ENSG00000208313 | SNORD77 | Up | 1 |
| Ac-H3K9 | 1 | 1.74E+08 | 1.74E+08 | 2862 | 311 | 2345.4 | 29.1 | 6.25 | ENSG00000208313 | SNORD77 | Up | 1 |
| Ac-H3K9 | 1 | 1.74E+08 | 1.74E+08 | 1513 | 38 | 108.04 | 7.0 | 0.76 | ENSG00000208317 | SNORD78 | Down | 1 |
| EWS | 1 | 1.74E+08 | 1.74E+08 | 1261 | 34 | 156.97 | 10.3 | 100 | ENSG00000208317 | SNORD78 | Down | 1 |
| FUS | 1 | 1.74E+08 | 1.74E+08 | 1673 | 47 | 251.36 | 11.5 | 100 | ENSG00000208317 | SNORD78 | Down | 1 |
| Ac-H3K9 | 1 | 1.74E+08 | 1.74E+08 | 716 | 23 | 119.88 | 17.2 | 0.73 | ENSG00000208317 | SNORD78 | Up | 1 |
| Ac-H3K9 | 1 | 1.74E+08 | 1.74E+08 | 2862 | 311 | 2345.4 | 29.1 | 6.25 | ENSG00000208317 | SNORD78 | Up | 1 |
| FUS | 5 | 1.81E+08 | 1.81E+08 | 1656 | 49 | 202.06 | 10.4 | 100 | ENSG00000208342 | SNORD96A | Down | 1 |
| EWS | 5 | 1.81E+08 | 1.81E+08 | 2070 | 55 | 203.32 | 8.6 | 100 | ENSG00000208342 | SNORD96A | Down | 1 |
| Ac-H3K9 | 5 | 1.81E+08 | 1.81E+08 | 5156 | 281 | 1658.6 | 28.6 | 2.15 | ENSG00000208342 | SNORD96A | Up | 1 |
| Ac-H3K9 | 5 | 1.81E+08 | 1.81E+08 | 3088 | 266 | 2358.1 | 57.4 | 6.67 | ENSG00000208342 | SNORD96A | Up | 1 |
| FUS | 13 | 9.20E+07 | 9.20E+07 | 2989 | 72 | 179.39 | 6.7 | 100 | ENSG00000215417 | MIR17HG | Down | 1 |
| FUS | 13 | 9.20E+07 | 9.20E+07 | 1954 | 55 | 125.65 | 6.7 | 100 | ENSG00000215417 | MIR17HG | Down | 1 |
| FUS | 13 | 9.20E+07 | 9.20E+07 | 2877 | 82 | 211.85 | 6.4 | 100 | ENSG00000215417 | MIR17HG | Down | 1 |
| EWS | 13 | 9.20E+07 | 9.20E+07 | 5252 | 144 | 294.99 | 6.4 | 100 | ENSG00000215417 | MIR17HG | Down | 1 |
| Ac-H3K9 | 13 | 9.20E+07 | 9.20E+07 | 1108 | 31 | 94.77 | 9.8 | 0.78 | ENSG00000215417 | MIR17HG | Exon | 1 |
| Ac-H3K9 | 13 | 9.20E+07 | 9.20E+07 | 1680 | 286 | 2213.6 | 36.8 | 4.88 | ENSG00000215417 | MIR17HG | Intron | 1 |
| Ac-H3K9 | 13 | 9.20E+07 | 9.20E+07 | 1257 | 30 | 89.04 | 6.5 | 0.77 | ENSG00000215417 | MIR17HG | Intron | 1 |
| Ac-H3K9 | 13 | 9.20E+07 | 9.20E+07 | 1574 | 81 | 398.62 | 17.5 | 0.36 | ENSG00000215417 | MIR17HG | Up | 1 |
| FUS | 19 | 5.13E+07 | 5.13E+07 | 1001 | 20 | 139.43 | 12.1 | 100 | ENSG00000220988 | SNORD88C | Down | 1 |
| EWS | 19 | 5.13E+07 | 5.13E+07 | 1774 | 45 | 266.73 | 20.0 | 100 | ENSG00000220988 | SNORD88C | Down | 1 |
| FUS | 19 | 5.13E+07 | 5.13E+07 | 837 | 14 | 90.31 | 12.1 | 100 | ENSG00000220988 | SNORD88C | Down | 1 |
| Ac-H3K9 | 19 | 5.13E+07 | 5.13E+07 | 2108 | 174 | 1759.9 | 70.2 | 2.47 | ENSG00000220988 | SNORD88C | Up | 1 |
| FUS | 19 | 5.13E+07 | 5.13E+07 | 1001 | 20 | 139.43 | 12.1 | 100 | ENSG00000221241 | SNORD88A | Down | 1 |
| EWS | 19 | 5.13E+07 | 5.13E+07 | 1774 | 45 | 266.73 | 20.0 | 100 | ENSG00000221241 | SNORD88A | Down | 1 |
| FUS | 19 | 5.13E+07 | 5.13E+07 | 837 | 14 | 90.31 | 12.1 | 100 | ENSG00000221241 | SNORD88A | Down | 1 |
| Ac-H3K9 | 19 | 5.13E+07 | 5.13E+07 | 2108 | 174 | 1759.9 | 70.2 | 2.47 | ENSG00000221241 | SNORD88A | Up | 1 |
| FUS | 19 | 5.13E+07 | 5.13E+07 | 1001 | 20 | 139.43 | 12.1 | 100 | ENSG00000221381 | SNORD88B | Down | 1 |
| EWS | 19 | 5.13E+07 | 5.13E+07 | 1774 | 45 | 266.73 | 20.0 | 100 | ENSG00000221381 | SNORD88B | Down | 1 |
| FUS | 19 | 5.13E+07 | 5.13E+07 | 837 | 14 | 90.31 | 12.1 | 100 | ENSG00000221381 | SNORD88B | Down | 1 |
| Ac-H3K9 | 19 | 5.13E+07 | 5.13E+07 | 2108 | 174 | 1759.9 | 70.2 | 2.47 | ENSG00000221381 | SNORD88B | Up | 1 |
| FUS | 3 | 1.87E+08 | 1.87E+08 | 596 | 14 | 91.11 | 12.5 | 100 | ENSG00000221420 | SNORA81 | Down | 1 |
| EWS | 3 | 1.87E+08 | 1.87E+08 | 980 | 27 | 180.95 | 17.6 | 100 | ENSG00000221420 | SNORA81 | Down | 1 |
| Ac-H3K9 | 3 | 1.86E+08 | 1.87E+08 | 4178 | 296 | 2539.6 | 42.2 | 9.09 | ENSG00000221420 | SNORA81 | Up | 1 |
| Ac-H3K9 | 6 | 7.43E+07 | 7.43E+07 | 670 | 15 | 87.41 | 15.0 | 0.78 | ENSG00000229862 | RP11-505P4.7 | Down | 2 |
| Ac-H3K9 | 6 | 7.42E+07 | 7.42E+07 | 962 | 37 | 265.44 | 25.3 | 0.52 | ENSG00000229862 | RP11-505P4.7 | Intron | 2 |
| FUS | 6 | 7.42E+07 | 7.42E+07 | 1387 | 32 | 107.03 | 7.1 | 100 | ENSG00000229862 | RP11-505P4.7 | Up | 2 |
| EWS | 6 | 7.42E+07 | 7.42E+07 | 1322 | 34 | 108.22 | 8.8 | 84.1 | ENSG00000229862 | RP11-505P4.7 | Up | 2 |
| Ac-H3K9 | 6 | 7.42E+07 | 7.42E+07 | 2409 | 230 | 2086 | 46.3 | 3.92 | ENSG00000229862 | RP11-505P4.7 | Up | 2 |
| Ac-H3K9 | 6 | 7.42E+07 | 7.42E+07 | 1334 | 38 | 198.42 | 13.5 | 0.52 | ENSG00000229862 | RP11-505P4.7 | Up | 2 |
| Ac-H3K9 | 1 | 1.74E+08 | 1.74E+08 | 2862 | 311 | 2345.4 | 29.1 | 6.25 | ENSG00000231792 | RP5-1198E17.1 | Down | 1 |
| Ac-H3K9 | 1 | 1.74E+08 | 1.74E+08 | 1513 | 38 | 108.04 | 7.0 | 0.76 | ENSG00000231792 | RP5-1198E17.1 | Down | 1 |
| Ac-H3K9 | 1 | 1.74E+08 | 1.74E+08 | 716 | 23 | 119.88 | 17.2 | 0.73 | ENSG00000231792 | RP5-1198E17.1 | Down | 1 |
| FUS | 1 | 1.74E+08 | 1.74E+08 | 1673 | 47 | 251.36 | 11.5 | 100 | ENSG00000231792 | RP5-1198E17.1 | Up | 1 |
| EWS | 1 | 1.74E+08 | 1.74E+08 | 1261 | 34 | 156.97 | 10.3 | 100 | ENSG00000231792 | RP5-1198E17.1 | Up | 1 |
| Ac-H3K9 | 5 | 1.81E+08 | 1.81E+08 | 5156 | 281 | 1658.6 | 28.6 | 2.15 | ENSG00000233937 | CTC-338M12.4 | Exon | 4 |
| Ac-H3K9 | 5 | 1.81E+08 | 1.81E+08 | 648 | 18 | 83.14 | 11.6 | 0.79 | ENSG00000233937 | CTC-338M12.4 | Intron | 4 |
| Ac-H3K9 | 5 | 1.81E+08 | 1.81E+08 | 1888 | 61 | 189.77 | 11.6 | 0.54 | ENSG00000233937 | CTC-338M12.4 | Intron | 4 |
| Ac-H3K9 | 5 | 1.81E+08 | 1.81E+08 | 1091 | 55 | 392.6 | 28.5 | 0.39 | ENSG00000233937 | CTC-338M12.4 | Intron | 4 |
| FUS | 5 | 1.81E+08 | 1.81E+08 | 1656 | 49 | 202.06 | 10.4 | 100 | ENSG00000233937 | CTC-338M12.4 | Up | 4 |
| EWS | 5 | 1.81E+08 | 1.81E+08 | 2070 | 55 | 203.32 | 8.6 | 100 | ENSG00000233937 | CTC-338M12.4 | Up | 4 |
| Ac-H3K9 | 5 | 1.81E+08 | 1.81E+08 | 3088 | 266 | 2358.1 | 57.4 | 6.67 | ENSG00000233937 | CTC-338M12.4 | Up | 4 |
| EWS | 1 | 1.74E+08 | 1.74E+08 | 1261 | 34 | 156.97 | 10.3 | 100 | ENSG00000234741 | GAS5 | Down | 29 |
| FUS | 1 | 1.74E+08 | 1.74E+08 | 1673 | 47 | 251.36 | 11.5 | 100 | ENSG00000234741 | GAS5 | Down | 29 |
| Ac-H3K9 | 1 | 1.74E+08 | 1.74E+08 | 2862 | 311 | 2345.4 | 29.1 | 6.25 | ENSG00000234741 | GAS5 | Exon | 29 |
| Ac-H3K9 | 1 | 1.74E+08 | 1.74E+08 | 1513 | 38 | 108.04 | 7.0 | 0.76 | ENSG00000234741 | GAS5 | Exon | 29 |
| Ac-H3K9 | 1 | 1.74E+08 | 1.74E+08 | 716 | 23 | 119.88 | 17.2 | 0.73 | ENSG00000234741 | GAS5 | Up | 29 |
| FUS | 9 | 1.94E+07 | 1.94E+07 | 1232 | 38 | 148.76 | 9.4 | 100 | ENSG00000234853 | RP11-513M16.5 | Down | 1 |
| EWS | 9 | 1.94E+07 | 1.94E+07 | 1298 | 35 | 116.41 | 6.3 | 89.6 | ENSG00000234853 | RP11-513M16.5 | Down | 1 |
| Ac-H3K9 | 9 | 1.94E+07 | 1.94E+07 | 3850 | 351 | 2729 | 50.6 | 18.2 | ENSG00000234853 | RP11-513M16.5 | Down | 1 |
| Ac-H3K9 | 9 | 8.66E+07 | 8.66E+07 | 4715 | 280 | 2126.3 | 55.6 | 4.17 | ENSG00000235298 | RP11-575L7.8 | Down | 1 |
| FUS | 9 | 8.66E+07 | 8.66E+07 | 722 | 16 | 91.77 | 9.5 | 100 | ENSG00000235298 | RP11-575L7.8 | Up | 1 |
| EWS | 9 | 8.66E+07 | 8.66E+07 | 930 | 22 | 120.23 | 13.1 | 100 | ENSG00000235298 | RP11-575L7.8 | Up | 1 |
| Ac-H3K9 | 1 | 2.88E+07 | 2.88E+07 | 1651 | 90 | 492.75 | 24.5 | 0.47 | ENSG00000238821 | snoU13.263 | Down | 1 |
| Ac-H3K9 | 1 | 2.88E+07 | 2.88E+07 | 6730 | 807 | 3100 | 40.7 | 16.7 | ENSG00000238821 | snoU13.263 | Up | 1 |
| EWS | 1 | 2.88E+07 | 2.88E+07 | 2270 | 110 | 495.44 | 17.0 | 100 | ENSG00000238821 | snoU13.263 | Up | 1 |
| FUS | 1 | 2.88E+07 | 2.88E+07 | 1523 | 77 | 409.11 | 15.0 | 100 | ENSG00000238821 | snoU13.263 | Up | 1 |
| FUS | 1 | 2.88E+07 | 2.88E+07 | 1267 | 36 | 150.89 | 7.1 | 100 | ENSG00000238821 | snoU13.263 | Up | 1 |
| EWS | 1 | 2.88E+07 | 2.88E+07 | 1371 | 38 | 132.6 | 8.7 | 100 | ENSG00000238821 | snoU13.263 | Up | 1 |
| Ac-H3K9 | 3 | 1.86E+08 | 1.87E+08 | 4178 | 296 | 2539.6 | 42.2 | 9.09 | ENSG00000238942 | snR39B.2 | Down | 1 |
| FUS | 3 | 1.87E+08 | 1.87E+08 | 596 | 14 | 91.11 | 12.5 | 100 | ENSG00000238942 | snR39B.2 | Down | 1 |
| EWS | 3 | 1.87E+08 | 1.87E+08 | 980 | 27 | 180.95 | 17.6 | 100 | ENSG00000238942 | snR39B.2 | Down | 1 |
| EWS | 13 | 9.20E+07 | 9.20E+07 | 5252 | 144 | 294.99 | 6.4 | 100 | ENSG00000239594 | RP11-282D2.3 | Down | 1 |
| FUS | 13 | 9.20E+07 | 9.20E+07 | 2989 | 72 | 179.39 | 6.7 | 100 | ENSG00000239594 | RP11-282D2.3 | Down | 1 |
| Ac-H3K9 | 13 | 9.20E+07 | 9.20E+07 | 1257 | 30 | 89.04 | 6.5 | 0.77 | ENSG00000239594 | RP11-282D2.3 | Down | 1 |
| FUS | 13 | 9.20E+07 | 9.20E+07 | 1954 | 55 | 125.65 | 6.7 | 100 | ENSG00000239594 | RP11-282D2.3 | Down | 1 |
| FUS | 13 | 9.20E+07 | 9.20E+07 | 2877 | 82 | 211.85 | 6.4 | 100 | ENSG00000239594 | RP11-282D2.3 | Down | 1 |
| Ac-H3K9 | 13 | 9.20E+07 | 9.20E+07 | 1574 | 81 | 398.62 | 17.5 | 0.36 | ENSG00000239594 | RP11-282D2.3 | Up | 1 |
| Ac-H3K9 | 13 | 9.20E+07 | 9.20E+07 | 1108 | 31 | 94.77 | 9.8 | 0.78 | ENSG00000239594 | RP11-282D2.3 | Up | 1 |
| Ac-H3K9 | 13 | 9.20E+07 | 9.20E+07 | 1680 | 286 | 2213.6 | 36.8 | 4.88 | ENSG00000239594 | RP11-282D2.3 | Up | 1 |
| Ac-H3K9 | 13 | 9.20E+07 | 9.20E+07 | 1257 | 30 | 89.04 | 6.5 | 0.77 | ENSG00000240532 | RP11-282D2.5 | Down | 1 |
| EWS | 13 | 9.20E+07 | 9.20E+07 | 5252 | 144 | 294.99 | 6.4 | 100 | ENSG00000240532 | RP11-282D2.5 | Down | 1 |
| FUS | 13 | 9.20E+07 | 9.20E+07 | 2989 | 72 | 179.39 | 6.7 | 100 | ENSG00000240532 | RP11-282D2.5 | Down | 1 |
| FUS | 13 | 9.20E+07 | 9.20E+07 | 1954 | 55 | 125.65 | 6.7 | 100 | ENSG00000240532 | RP11-282D2.5 | Down | 1 |
| FUS | 13 | 9.20E+07 | 9.20E+07 | 2877 | 82 | 211.85 | 6.4 | 100 | ENSG00000240532 | RP11-282D2.5 | Down | 1 |
| Ac-H3K9 | 13 | 9.20E+07 | 9.20E+07 | 1574 | 81 | 398.62 | 17.5 | 0.36 | ENSG00000240532 | RP11-282D2.5 | Up | 1 |
| Ac-H3K9 | 13 | 9.20E+07 | 9.20E+07 | 1108 | 31 | 94.77 | 9.8 | 0.78 | ENSG00000240532 | RP11-282D2.5 | Up | 1 |
| Ac-H3K9 | 13 | 9.20E+07 | 9.20E+07 | 1680 | 286 | 2213.6 | 36.8 | 4.88 | ENSG00000240532 | RP11-282D2.5 | Up | 1 |
| FUS | 13 | 9.20E+07 | 9.20E+07 | 2877 | 82 | 211.85 | 6.4 | 100 | ENSG00000242008 | RP11-282D2.2 | Down | 1 |
| EWS | 13 | 9.20E+07 | 9.20E+07 | 5252 | 144 | 294.99 | 6.4 | 100 | ENSG00000242008 | RP11-282D2.2 | Down | 1 |
| Ac-H3K9 | 13 | 9.20E+07 | 9.20E+07 | 1257 | 30 | 89.04 | 6.5 | 0.77 | ENSG00000242008 | RP11-282D2.2 | Down | 1 |
| FUS | 13 | 9.20E+07 | 9.20E+07 | 2989 | 72 | 179.39 | 6.7 | 100 | ENSG00000242008 | RP11-282D2.2 | Down | 1 |
| FUS | 13 | 9.20E+07 | 9.20E+07 | 1954 | 55 | 125.65 | 6.7 | 100 | ENSG00000242008 | RP11-282D2.2 | Down | 1 |
| Ac-H3K9 | 13 | 9.20E+07 | 9.20E+07 | 1574 | 81 | 398.62 | 17.5 | 0.36 | ENSG00000242008 | RP11-282D2.2 | Up | 1 |
| Ac-H3K9 | 13 | 9.20E+07 | 9.20E+07 | 1108 | 31 | 94.77 | 9.8 | 0.78 | ENSG00000242008 | RP11-282D2.2 | Up | 1 |
| Ac-H3K9 | 13 | 9.20E+07 | 9.20E+07 | 1680 | 286 | 2213.6 | 36.8 | 4.88 | ENSG00000242008 | RP11-282D2.2 | Up | 1 |
| FUS | 1 | 2.88E+07 | 2.88E+07 | 1523 | 77 | 409.11 | 15.0 | 100 | ENSG00000242125 | SNHG3 | Down | 3 |
| FUS | 1 | 2.88E+07 | 2.88E+07 | 1267 | 36 | 150.89 | 7.1 | 100 | ENSG00000242125 | SNHG3 | Down | 3 |
| EWS | 1 | 2.88E+07 | 2.88E+07 | 1371 | 38 | 132.6 | 8.7 | 100 | ENSG00000242125 | SNHG3 | Down | 3 |
| Ac-H3K9 | 1 | 2.88E+07 | 2.88E+07 | 1651 | 90 | 492.75 | 24.5 | 0.47 | ENSG00000242125 | SNHG3 | Down | 3 |
| EWS | 1 | 2.88E+07 | 2.88E+07 | 2270 | 110 | 495.44 | 17.0 | 100 | ENSG00000242125 | SNHG3 | Exon | 3 |
| Ac-H3K9 | 1 | 2.88E+07 | 2.88E+07 | 6730 | 807 | 3100 | 40.7 | 16.7 | ENSG00000242125 | SNHG3 | Up | 3 |
| Ac-H3K9 | 13 | 9.20E+07 | 9.20E+07 | 1257 | 30 | 89.04 | 6.5 | 0.77 | ENSG00000243108 | RP11-282D2.6 | Down | 1 |
| EWS | 13 | 9.20E+07 | 9.20E+07 | 5252 | 144 | 294.99 | 6.4 | 100 | ENSG00000243108 | RP11-282D2.6 | Down | 1 |
| FUS | 13 | 9.20E+07 | 9.20E+07 | 2989 | 72 | 179.39 | 6.7 | 100 | ENSG00000243108 | RP11-282D2.6 | Down | 1 |
| FUS | 13 | 9.20E+07 | 9.20E+07 | 1954 | 55 | 125.65 | 6.7 | 100 | ENSG00000243108 | RP11-282D2.6 | Down | 1 |
| FUS | 13 | 9.20E+07 | 9.20E+07 | 2877 | 82 | 211.85 | 6.4 | 100 | ENSG00000243108 | RP11-282D2.6 | Down | 1 |
| Ac-H3K9 | 13 | 9.20E+07 | 9.20E+07 | 1574 | 81 | 398.62 | 17.5 | 0.36 | ENSG00000243108 | RP11-282D2.6 | Up | 1 |
| Ac-H3K9 | 13 | 9.20E+07 | 9.20E+07 | 1108 | 31 | 94.77 | 9.8 | 0.78 | ENSG00000243108 | RP11-282D2.6 | Up | 1 |
| Ac-H3K9 | 13 | 9.20E+07 | 9.20E+07 | 1680 | 286 | 2213.6 | 36.8 | 4.88 | ENSG00000243108 | RP11-282D2.6 | Up | 1 |
| EWS | 13 | 9.20E+07 | 9.20E+07 | 5252 | 144 | 294.99 | 6.4 | 100 | ENSG00000243839 | RP11-282D2.4 | Down | 1 |
| FUS | 13 | 9.20E+07 | 9.20E+07 | 2989 | 72 | 179.39 | 6.7 | 100 | ENSG00000243839 | RP11-282D2.4 | Down | 1 |
| Ac-H3K9 | 13 | 9.20E+07 | 9.20E+07 | 1257 | 30 | 89.04 | 6.5 | 0.77 | ENSG00000243839 | RP11-282D2.4 | Down | 1 |
| FUS | 13 | 9.20E+07 | 9.20E+07 | 1954 | 55 | 125.65 | 6.7 | 100 | ENSG00000243839 | RP11-282D2.4 | Down | 1 |
| FUS | 13 | 9.20E+07 | 9.20E+07 | 2877 | 82 | 211.85 | 6.4 | 100 | ENSG00000243839 | RP11-282D2.4 | Down | 1 |
| Ac-H3K9 | 13 | 9.20E+07 | 9.20E+07 | 1574 | 81 | 398.62 | 17.5 | 0.36 | ENSG00000243839 | RP11-282D2.4 | Up | 1 |
| Ac-H3K9 | 13 | 9.20E+07 | 9.20E+07 | 1108 | 31 | 94.77 | 9.8 | 0.78 | ENSG00000243839 | RP11-282D2.4 | Up | 1 |
| Ac-H3K9 | 13 | 9.20E+07 | 9.20E+07 | 1680 | 286 | 2213.6 | 36.8 | 4.88 | ENSG00000243839 | RP11-282D2.4 | Up | 1 |
| Ac-H3K9 | 1 | 2.88E+07 | 2.88E+07 | 6730 | 807 | 3100 | 40.7 | 16.7 | ENSG00000245178 | AL513497.1 | Down | 1 |
| EWS | 1 | 2.88E+07 | 2.88E+07 | 2270 | 110 | 495.44 | 17.0 | 100 | ENSG00000245178 | AL513497.1 | Up | 1 |
| Ac-H3K9 | 1 | 2.88E+07 | 2.88E+07 | 1651 | 90 | 492.75 | 24.5 | 0.47 | ENSG00000245178 | AL513497.1 | Up | 1 |
| FUS | 1 | 2.88E+07 | 2.88E+07 | 1267 | 36 | 150.89 | 7.1 | 100 | ENSG00000245178 | AL513497.1 | Up | 1 |
| EWS | 1 | 2.88E+07 | 2.88E+07 | 1371 | 38 | 132.6 | 8.7 | 100 | ENSG00000245178 | AL513497.1 | Up | 1 |
| FUS | 1 | 2.88E+07 | 2.88E+07 | 1523 | 77 | 409.11 | 15.0 | 100 | ENSG00000245178 | AL513497.1 | Up | 1 |
| Ac-H3K9 | 5 | 1.81E+08 | 1.81E+08 | 975 | 34 | 224.08 | 29.0 | 0.55 | ENSG00000247049 | CTC-338M12.7 | Down | 1 |
| Ac-H3K9 | 5 | 1.81E+08 | 1.81E+08 | 647 | 17 | 101.52 | 15.3 | 0.76 | ENSG00000247049 | CTC-338M12.7 | Down | 1 |
| Ac-H3K9 | 5 | 1.81E+08 | 1.81E+08 | 1238 | 33 | 179.27 | 14.9 | 0.55 | ENSG00000247049 | CTC-338M12.7 | Down | 1 |
| FUS | 5 | 1.81E+08 | 1.81E+08 | 1656 | 49 | 202.06 | 10.4 | 100 | ENSG00000247049 | CTC-338M12.7 | Up | 1 |
| EWS | 5 | 1.81E+08 | 1.81E+08 | 2070 | 55 | 203.32 | 8.6 | 100 | ENSG00000247049 | CTC-338M12.7 | Up | 1 |
| FUS | 8 | 1.29E+08 | 1.29E+08 | 1533 | 31 | 89.81 | 7.9 | 100 | ENSG00000249375 | RP11-1136L8.1 | Up | 3 |
| EWS | 8 | 1.29E+08 | 1.29E+08 | 1385 | 35 | 151.69 | 8.7 | 100 | ENSG00000249375 | RP11-1136L8.1 | Up | 3 |
| Ac-H3K9 | 8 | 1.29E+08 | 1.29E+08 | 735 | 17 | 85.81 | 16.9 | 0.8 | ENSG00000249375 | RP11-1136L8.1 | Up | 3 |
| Ac-H3K9 | 8 | 1.29E+08 | 1.29E+08 | 847 | 28 | 183.67 | 22.5 | 0.54 | ENSG00000249375 | RP11-1136L8.1 | Up | 3 |
| Ac-H3K9 | 8 | 1.29E+08 | 1.29E+08 | 665 | 17 | 106.01 | 18.7 | 0.76 | ENSG00000249375 | RP11-1136L8.1 | Up | 3 |
| Ac-H3K9 | 8 | 1.29E+08 | 1.29E+08 | 631 | 24 | 177.97 | 25.3 | 0.55 | ENSG00000249375 | RP11-1136L8.1 | Up | 3 |
| Ac-H3K9 | 8 | 1.29E+08 | 1.29E+08 | 735 | 17 | 85.81 | 16.9 | 0.8 | ENSG00000256129 | AC103819.1 | Down | 1 |
| Ac-H3K9 | 8 | 1.29E+08 | 1.29E+08 | 847 | 28 | 183.67 | 22.5 | 0.54 | ENSG00000256129 | AC103819.1 | Down | 1 |
| EWS | 8 | 1.29E+08 | 1.29E+08 | 1385 | 35 | 151.69 | 8.7 | 100 | ENSG00000256129 | AC103819.1 | Down | 1 |
| FUS | 8 | 1.29E+08 | 1.29E+08 | 1533 | 31 | 89.81 | 7.9 | 100 | ENSG00000256129 | AC103819.1 | Down | 1 |
| Ac-H3K9 | 8 | 1.29E+08 | 1.29E+08 | 665 | 17 | 106.01 | 18.7 | 0.76 | ENSG00000256129 | AC103819.1 | Exon | 1 |
| Ac-H3K9 | 8 | 1.29E+08 | 1.29E+08 | 631 | 24 | 177.97 | 25.3 | 0.55 | ENSG00000256129 | AC103819.1 | Up | 1 |
| Ac-H3K9 | 1 | 2.88E+07 | 2.88E+07 | 6730 | 807 | 3100 | 40.7 | 16.7 | ENSG00000256960 | AL513497.2 | Down | 1 |
| FUS | 1 | 2.88E+07 | 2.88E+07 | 1267 | 36 | 150.89 | 7.1 | 100 | ENSG00000256960 | AL513497.2 | Down | 1 |
| EWS | 1 | 2.88E+07 | 2.88E+07 | 1371 | 38 | 132.6 | 8.7 | 100 | ENSG00000256960 | AL513497.2 | Down | 1 |
| FUS | 1 | 2.88E+07 | 2.88E+07 | 1523 | 77 | 409.11 | 15.0 | 100 | ENSG00000256960 | AL513497.2 | Down | 1 |
| EWS | 1 | 2.88E+07 | 2.88E+07 | 2270 | 110 | 495.44 | 17.0 | 100 | ENSG00000256960 | AL513497.2 | Down | 1 |
| Ac-H3K9 | 1 | 2.88E+07 | 2.88E+07 | 1651 | 90 | 492.75 | 24.5 | 0.47 | ENSG00000256960 | AL513497.2 | Intron | 1 |
|  |  |  |  |  |  |  |  |  |  |  |  |  |
| **FUS** |  |  |  |  |  |  |  |  |  |  |  |  |
| **Sample** | **Ch.** | **peak start** | **peak end** | **length** | **tags** | **p-value** | **F.C** | **FDR** | **ENS ID** | **Gene name** | **location** | **T** |
| FUS | 22 | 4.30E+07 | 4.30E+07 | 562 | 12 | 84.45 | 15.0 | 100 | ENSG00000202058 | 7SK.80 | Up | 1 |
| FUS | 16 | 7.43E+07 | 7.43E+07 | 692 | 13 | 88.91 | 9.7 | 100 | ENSG00000239763 | AC009120.3 | Down | 1 |
| FUS | 22 | 4.30E+07 | 4.30E+07 | 562 | 12 | 84.45 | 15.0 | 100 | ENSG00000251913 | U6.1018 | Down | 1 |
|  |  |  |  |  |  |  |  |  |  |  |  |  |
| **EWS** |  |  |  |  |  |  |  |  |  |  |  |  |
| **Sample** | **Ch.** | **peak start** | **peak end** | **length** | **tags** | **p-value** | **F.C** | **FDR** | **ENS ID** | **Gene name** | **location** | **T** |
| EWS | 6 | 7.28E+07 | 7.28E+07 | 739 | 14 | 81.19 | 10.6 | 57.9 | ENSG00000079841 | RIMS1 | Intron | 26 |
| EWS | 22 | 3.89E+07 | 3.89E+07 | 1598 | 41 | 137.79 | 6.7 | 100 | ENSG00000100196 | KDELR3 | Exon | 3 |
| EWS | 4 | 8.61E+06 | 8.61E+06 | 744 | 14 | 87.82 | 11.9 | 65.1 | ENSG00000109625 | CPZ | Intron | 11 |
| EWS | 2 | 1.12E+07 | 1.12E+07 | 1080 | 24 | 94.66 | 7.5 | 67.4 | ENSG00000145063 | AC062028.1 | Down | 5 |
| EWS | 5 | 1.36E+08 | 1.36E+08 | 795 | 15 | 101.38 | 15.3 | 77.5 | ENSG00000152377 | SPOCK1 | Intron | 10 |
| EWS | 4 | 8.61E+06 | 8.61E+06 | 744 | 14 | 87.82 | 11.9 | 65.1 | ENSG00000155269 | GPR78 | Intron | 7 |
| EWS | 1 | 6.05E+07 | 6.05E+07 | 684 | 20 | 84.07 | 8.9 | 60.9 | ENSG00000162598 | C1orf87 | Intron | 6 |
| EWS | 8 | 8.73E+07 | 8.73E+07 | 733 | 14 | 85.42 | 12.9 | 62.7 | ENSG00000164893 | SLC7A13 | Intron | 3 |
| EWS | 16 | 2.07E+07 | 2.07E+07 | 852 | 16 | 105.49 | 13.6 | 79.4 | ENSG00000166743 | ACSM1 | Intron | 7 |
| EWS | 12 | 2.05E+07 | 2.05E+07 | 735 | 16 | 105.55 | 17.7 | 80.6 | ENSG00000172572 | PDE3A | Intron | 3 |
| EWS | 1 | 4.53E+07 | 4.53E+07 | 2119 | 65 | 184.45 | 6.5 | 100 | ENSG00000173846 | PLK3 | Up | 8 |
| EWS | 22 | 1.87E+07 | 1.87E+07 | 2002 | 71 | 125.09 | 7.2 | 100 | ENSG00000182824 | AC008132.1 | Up | 1 |
| EWS | 22 | 2.07E+07 | 2.07E+07 | 1237 | 45 | 88.84 | 5.0 | 65 | ENSG00000188280 | AC007731.1 | Up | 2 |
| EWS | 1 | 4.53E+07 | 4.53E+07 | 2119 | 65 | 184.45 | 6.5 | 100 | ENSG00000188396 | TCTEX1D4 | Down | 2 |
| EWS | 1 | 2.20E+08 | 2.20E+08 | 955 | 24 | 102.02 | 7.2 | 78.6 | ENSG00000196660 | SLC30A10 | Up | 6 |
| EWS | 19 | 5.47E+07 | 5.47E+07 | 1220 | 22 | 100.1 | 7.5 | 74.3 | ENSG00000204577 | LILRB3 | Down | 11 |
| EWS | 17 | 3.71E+07 | 3.71E+07 | 977 | 22 | 100.01 | 8.0 | 72.4 | ENSG00000204952 | FBXO47 | Intron | 1 |
| EWS | 7 | 1.42E+08 | 1.42E+08 | 1094 | 36 | 103.09 | 8.6 | 79.7 | ENSG00000204987 | RP11-1220K2.2 | Intron | 5 |
| EWS | 22 | 2.15E+07 | 2.15E+07 | 1201 | 39 | 125.84 | 7.0 | 100 | ENSG00000215498 | KB-1183D5.11 | Up | 9 |
| EWS | 1 | 2.20E+08 | 2.20E+08 | 955 | 24 | 102.02 | 7.2 | 78.6 | ENSG00000221673 | U3.54 | Up | 1 |
| EWS | 9 | 3.30E+07 | 3.30E+07 | 1235 | 25 | 108.9 | 8.2 | 85.3 | ENSG00000222169 | AL162590.1 | Down | 1 |
| EWS | 1 | 1.92E+08 | 1.92E+08 | 1091 | 37 | 97.63 | 7.5 | 74.4 | ENSG00000228215 | RP11-541F9.2 | Intron | 1 |
| EWS | 13 | 6.33E+07 | 6.33E+07 | 1309 | 46 | 116.22 | 6.2 | 87.8 | ENSG00000228669 | RP11-234O23.1 | Intron | 2 |
| EWS | 13 | 8.99E+07 | 8.99E+07 | 1272 | 37 | 108.93 | 6.9 | 86.7 | ENSG00000234660 | RP11-309H8.3 | Up | 1 |
| EWS | 2 | 1.82E+08 | 1.82E+08 | 759 | 16 | 92.53 | 13.9 | 69.7 | ENSG00000234663 | AC104820.2 | Intron | 5 |
| EWS | 15 | 6.60E+07 | 6.60E+07 | 801 | 19 | 94.51 | 11.0 | 66.7 | ENSG00000238311 | snoU13.9 | Up | 1 |
| EWS | 19 | 3.50E+07 | 3.50E+07 | 649 | 13 | 80.23 | 13.6 | 56.5 | ENSG00000239210 | CTD-2588C8.1 | Down | 1 |
| EWS | 3 | 1.70E+08 | 1.70E+08 | 826 | 15 | 86.92 | 15.7 | 65.7 | ENSG00000240373 | RP11-379K17.7 | Intron | 1 |
| EWS | 3 | 1.63E+08 | 1.63E+08 | 799 | 15 | 95.16 | 14.1 | 69.1 | ENSG00000241168 | RP11-10O22.1 | Intron | 1 |
| EWS | 19 | 5.47E+07 | 5.47E+07 | 1220 | 22 | 100.1 | 7.5 | 74.3 | ENSG00000244482 | LILRA6 | Down | 9 |
| EWS | 16 | 2.07E+07 | 2.07E+07 | 852 | 16 | 105.49 | 13.6 | 79.4 | ENSG00000246328 | AC020926.1 | Up | 1 |
| EWS | 5 | 1.52E+08 | 1.52E+08 | 669 | 13 | 83.92 | 11.7 | 60.2 | ENSG00000249484 | AC091969.1 | Intron | 4 |
| EWS | 4 | 1.13E+08 | 1.13E+08 | 596 | 13 | 80.13 | 11.1 | 55.6 | ENSG00000249815 | RP11-269F21.3 | Intron | 1 |
