# Supplementary material for: EWS and FUS bind a subset of transcribed genes encoding proteins enriched in RNA regulatory functions
Source: BMC Genomics. 2015 Nov 14;16:929. doi: 10.1186/s12864-015-2125-9 (PMC4647676; doi:10.1186/s12864-015-2125-9)
Supplement: Additional file 14: — Comparative analysis and alignment to the human genome (UCSC hg19) of raw FUS ChIP-seq reads from this study and recalculated from the few data files presented in the study by Schwartz et al. [30]. (DOCX 16 kb) [file 12864_2015_2125_MOESM14_ESM.docx]

## Additional file 14

| **samples** | **sample name** | **Number of reads** | **Average read length** | **mapped reads** | **Mapped reads (%)** |
| --- | --- | --- | --- | --- | --- |
| FUS.si | siFUS-ref ^a^ | 22588717 | 41 | 3290866 | 14.57% ^f^ |
| FUS.untreated | unFUS-ref ^b^ | 19249230 | 50 | 13743053 | 71.40% |
| control | input-ref ^c^ | 73394528 | 100 | 65038171 | 88.61% |
| JB1.fq.gz | FUS-JB ^d^ | 9795918 | 49 | 9301703 | 94.95% |
| JB6.fq.gz | input-JB ^e^ | 9795918 | 49 | 9208323 | 94.00% |

a, Schwartz et al: control FUS ChIP-seq data (with pretreatment of cells with a siRNA against FUS, siFUS)

b, Schwartz et al: FUS ChIP-seq data (without pretreatment of cells with a siRNA against FUS unFUS)

c, Schwartz et al: Input sample.

d, Our study: FUS ChIP-seq data

E, Our study: input sample.

F, Note the low mapping rate to the human genome in alignment with an expected minimal enrichment of specific DNA.
