# Supplementary material for: EWS and FUS bind a subset of transcribed genes encoding proteins enriched in RNA regulatory functions
Source: BMC Genomics. 2015 Nov 14;16:929. doi: 10.1186/s12864-015-2125-9 (PMC4647676; doi:10.1186/s12864-015-2125-9)
Supplement: Additional file 15: — Comparative distribution of p-values for ChIP-seq peaks called by FindPeaks and MACS of data from this study and the study by Schwarts et al. [30]. A. Distribution of p-values for ChIP-seq peaks called by FindPeaks (F). Most of the ChIP-seq peaks from the hereby presented FUS ChIP-seq data (unFUS_JB) have a low p-value. Most of the ChIP-seq peaks from Schwartz’s et al. (unFUS_ref) have a high P-value. B. Distribution of p-values for ChIP-seq peaks called by MACS. A lower number of ChIP-seq peaks were identified compared to with usage of FindPeaks and the p-value distribution is more comparable between the two datasets. (DOCX 106 kb) [file 12864_2015_2125_MOESM15_ESM.docx]

## Additional File 15

## A

##

**B**
