# Supplementary material for: EWS and FUS bind a subset of transcribed genes encoding proteins enriched in RNA regulatory functions
Source: BMC Genomics. 2015 Nov 14;16:929. doi: 10.1186/s12864-015-2125-9 (PMC4647676; doi:10.1186/s12864-015-2125-9)

## Additional File 16

**# of hits**

**# of hits**

**A B**

**unFUS-JB unFUS-ref**


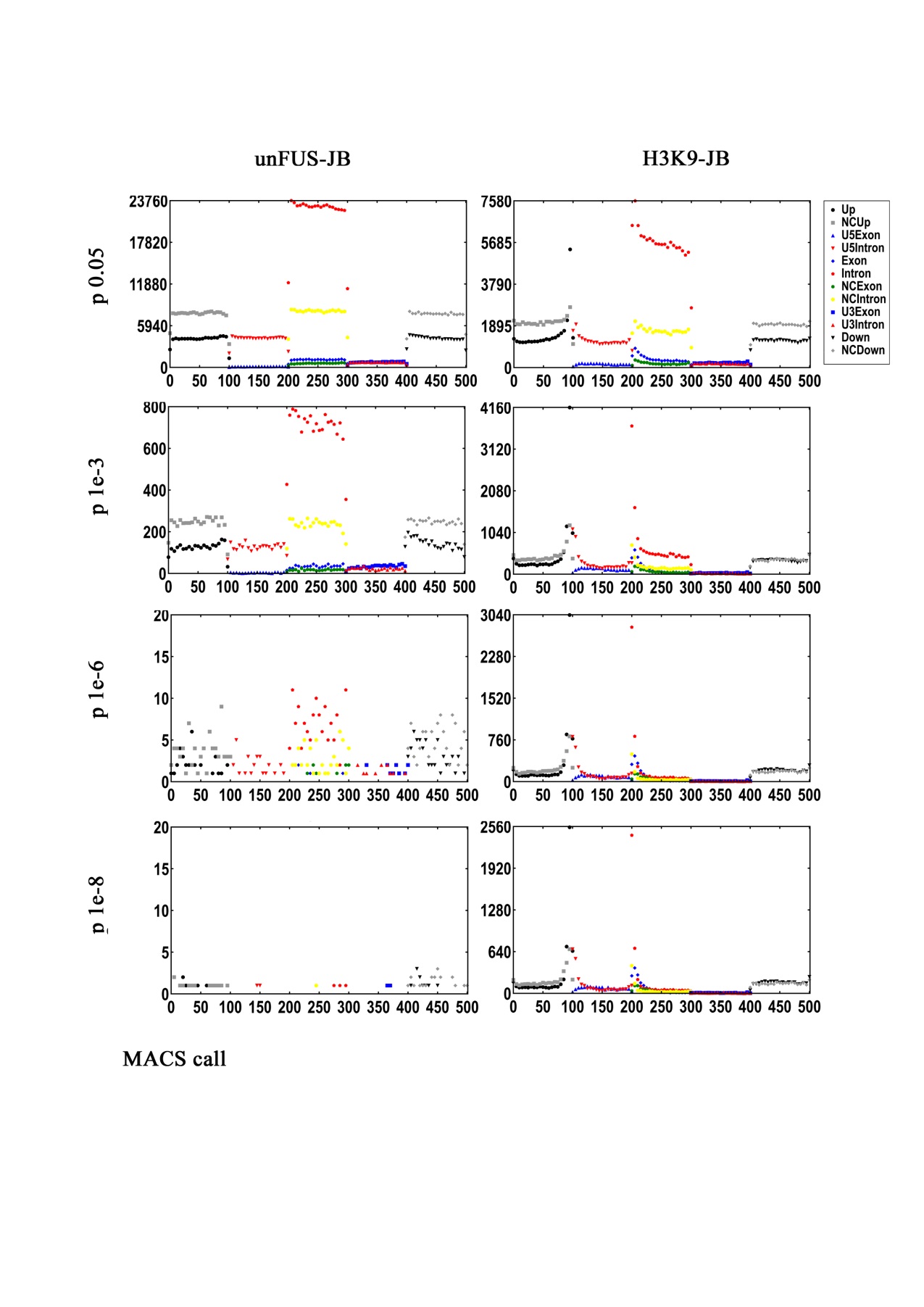

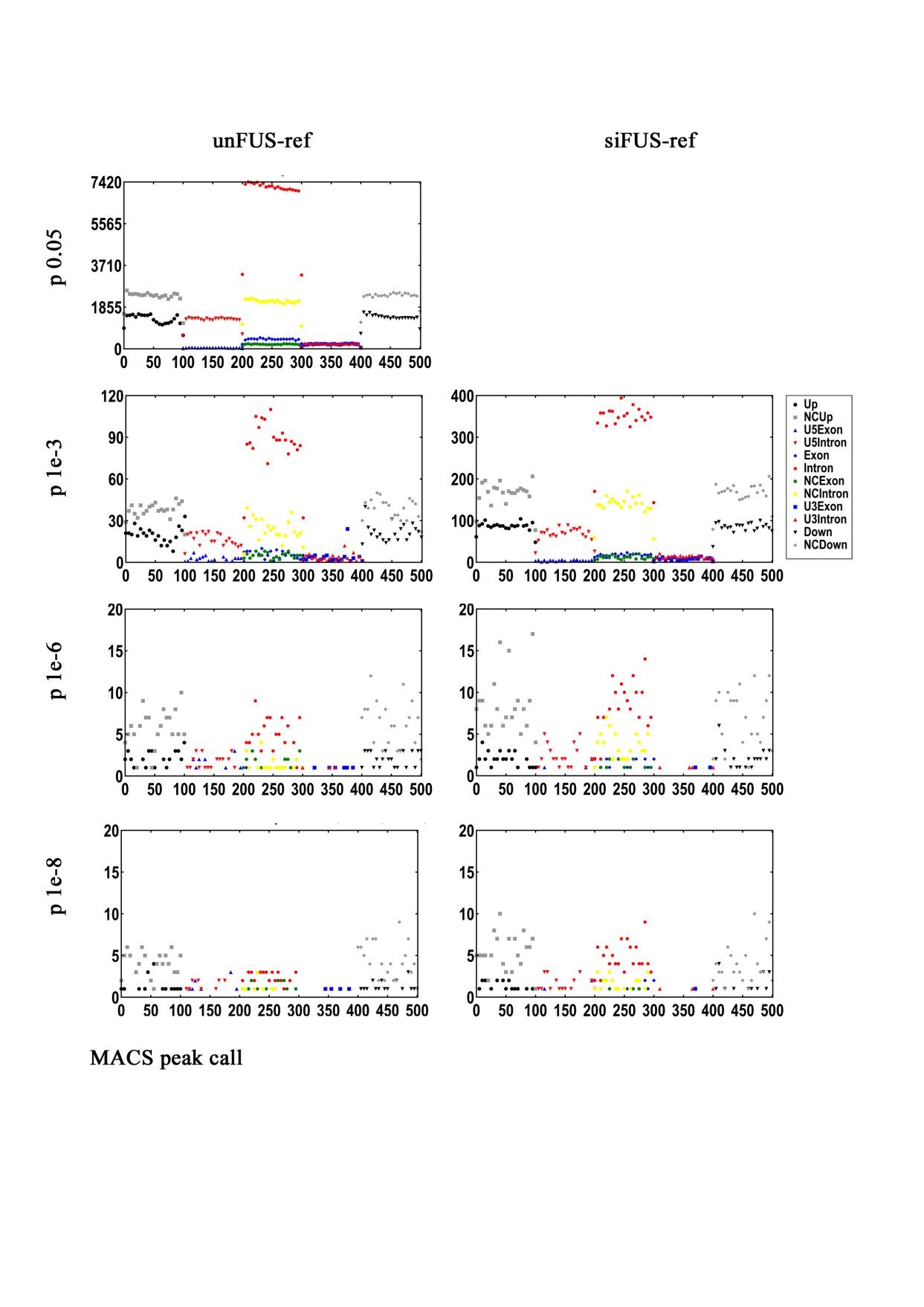

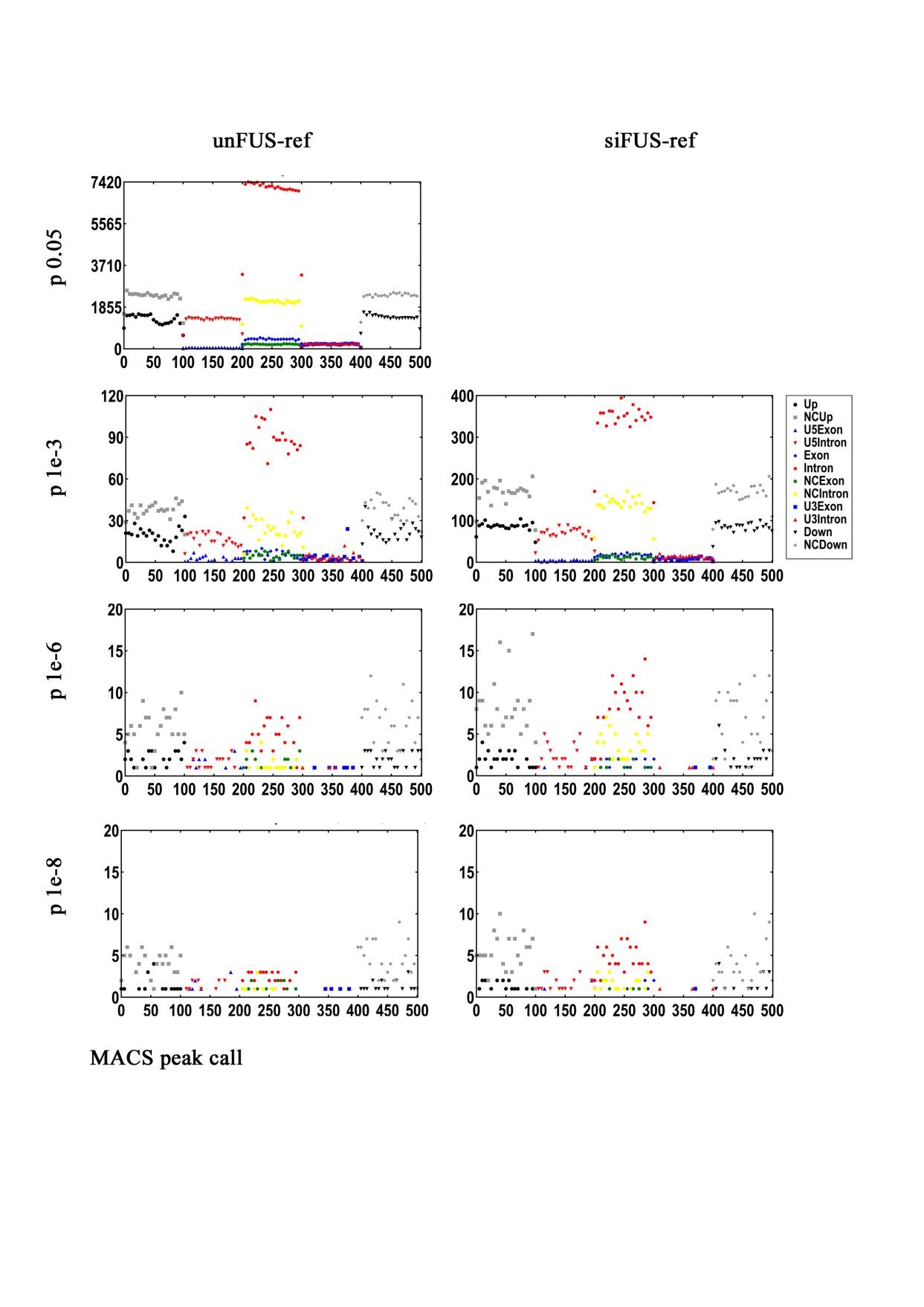


**# of hits**

**# of hits**

**# of hits**

**# of hits**

**position of hits in gene model**

**position of hits in gene model**

**C**


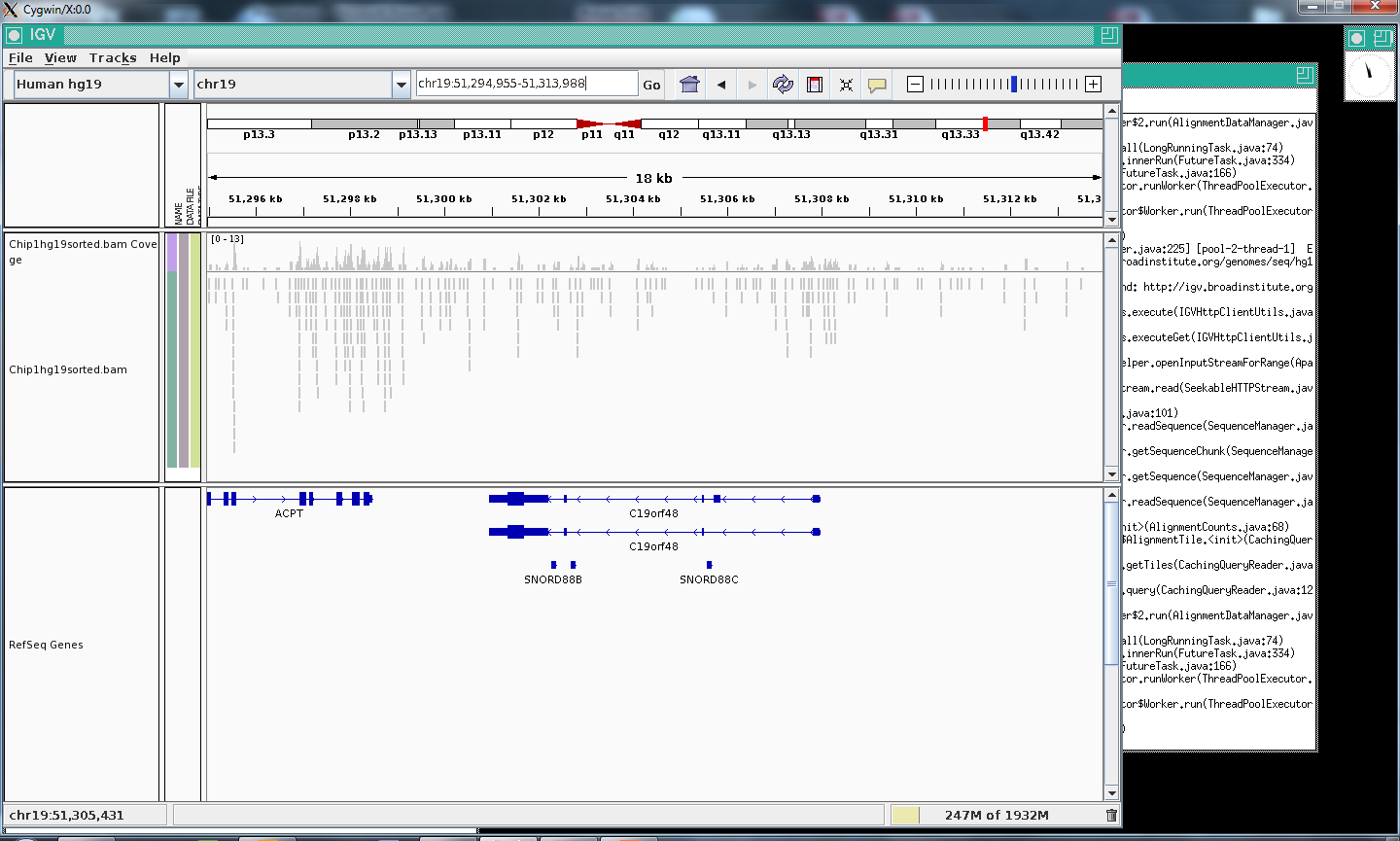


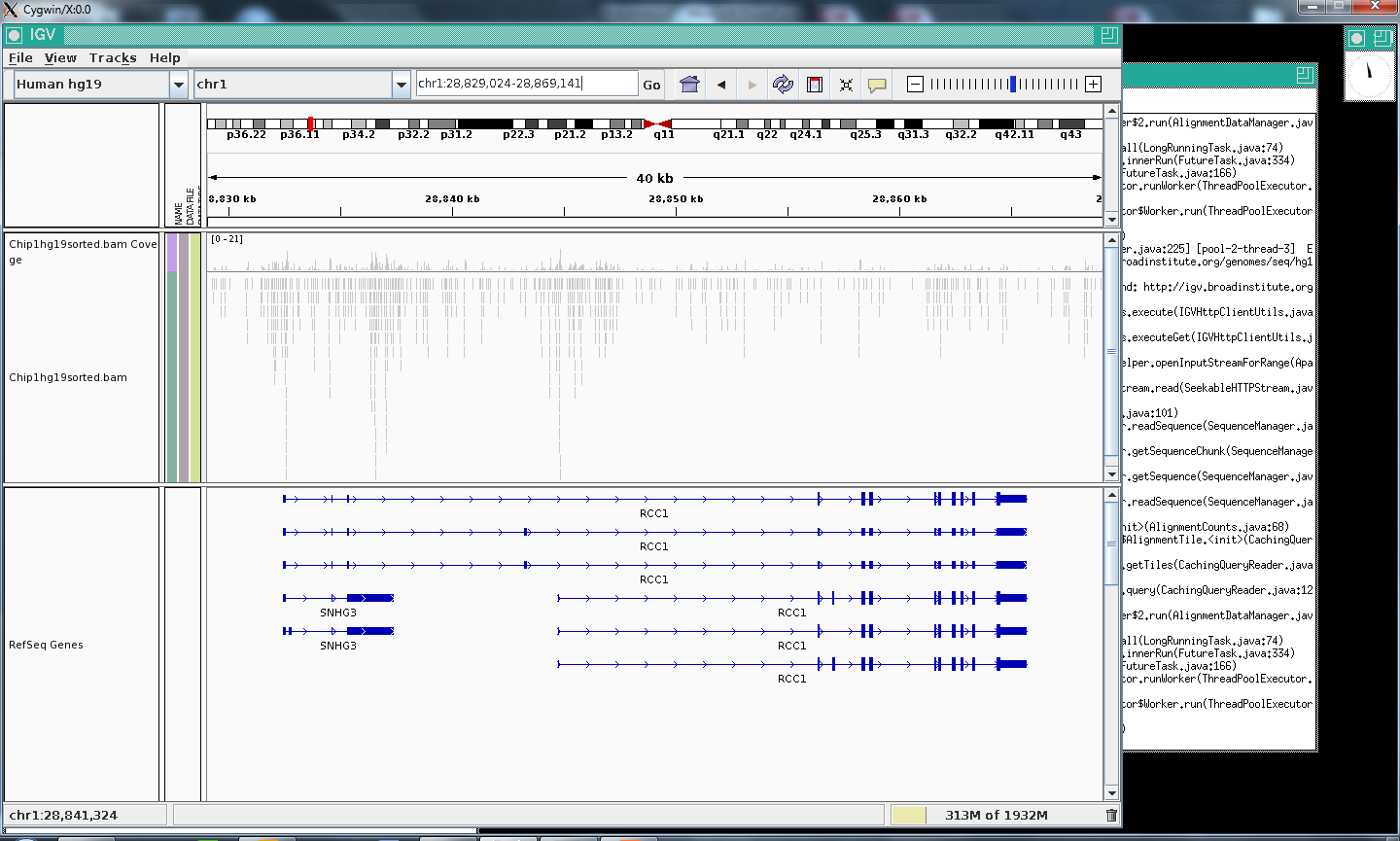


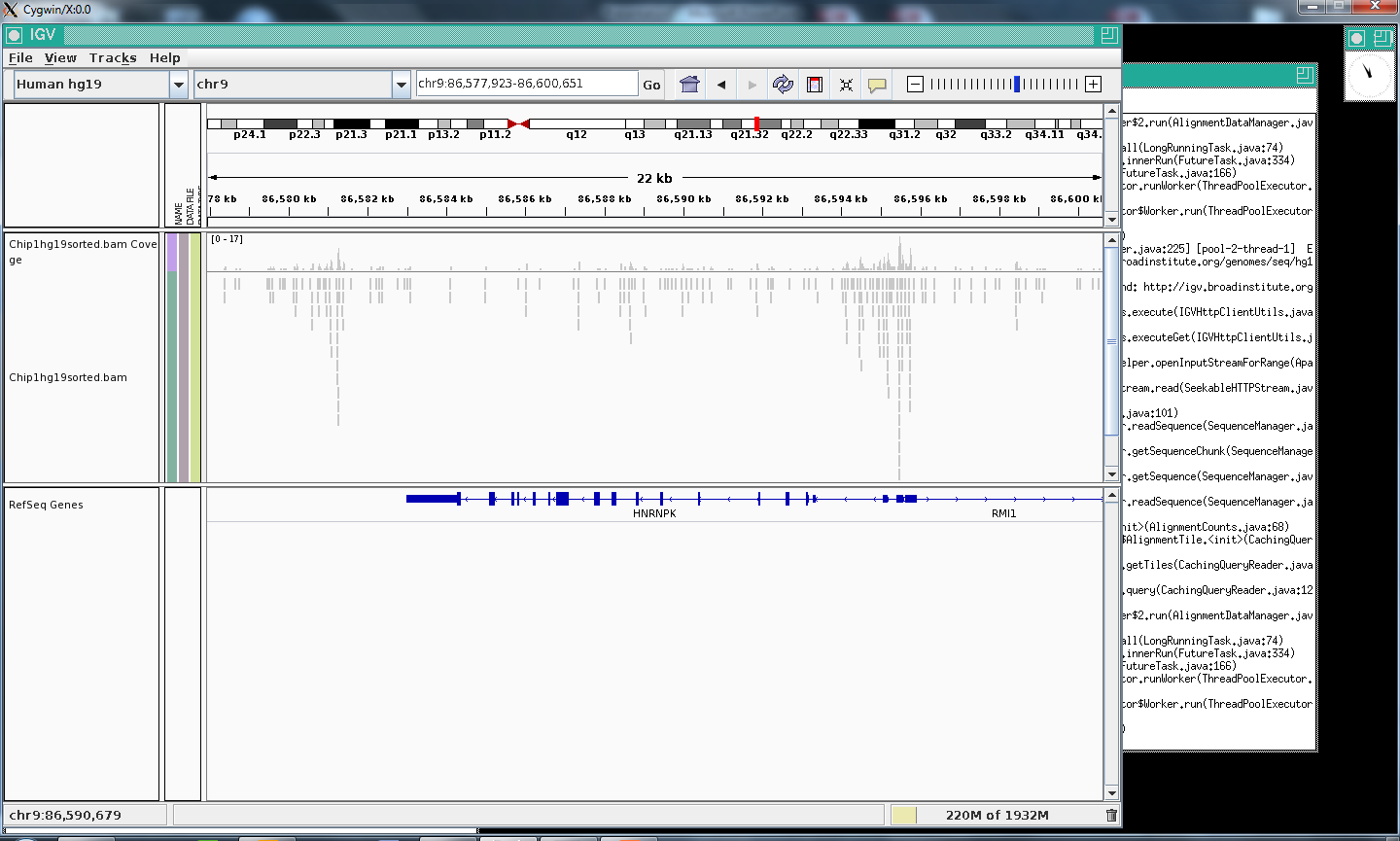

Supplement: Additional file 16: — Comparison of the distribution of MACS called ChIP-seq peaks across a gene model using data from this study or the study by Schwarts et al. [30]. A. ChIP-seq data from this study for FUS (unFUS-JB) and B. ChIP-seq data from Schwarts et al. for FUS from cells not pretreated with FUS siRNA (unFUS-ref). Cut-off levels for P-values are indicated to the left for each figure panel. Data were plotted using gene modelling with number of hits, which represents the number of annotated UCSC transcripts corresponding to the position of a given ChIP-seq peak, shown at the y-axis. The gene model presented on the x-axis is based on the following features: position 1 – 100: UP, 10 kb upstream region of the transcription start site of coding genes; NCUp, 10 kb upstream region of the transcription start site of noncoding genes; Position 100–200: U5Exon, 5’ untranslated exon region; U5Intron, 5’ intron region upstream of the translation start site; Position 200–300: Exon, Coding region of the exon sequences; Intron, Intron region between the translation start site and stop site; NCExon, Exon region of noncoding genes; NCIntron, Intron region of noncoding genes; Position 300–400: U3Exon, 3’ untranslated exon region; U3Intron, 3’ intron region downstream of the translation stop site; Position 400–500: Down, 10 kb downstream region of the poly(A)-signal of coding genes; UCDown, 10 kb downstream region of the transcription poly(A) of noncoding genes. The features were subdivided in smaller segments for precision mapping. C. MACS peak output from Schwarts et al. data of the exemplified ACPT and C19ORF48, SNHG3 and RCC1 and HNRNPK genes. (DOCX 842 kb) [file 12864_2015_2125_MOESM16_ESM.docx]
