# Supplementary material for: EWS and FUS bind a subset of transcribed genes encoding proteins enriched in RNA regulatory functions
Source: BMC Genomics. 2015 Nov 14;16:929. doi: 10.1186/s12864-015-2125-9 (PMC4647676; doi:10.1186/s12864-015-2125-9)
Supplement: Additional file 17: — Comparison of the distribution of FindPeaks called ChIP-seq peaks across a model gene using data from this study or the study by Schwarts et al. [30]. For A. and B. legend details see Additional file 16. (DOCX 693 kb) [file 12864_2015_2125_MOESM17_ESM.docx]

## Additional File 17

**# of hits**

**# of hits**

**A B**


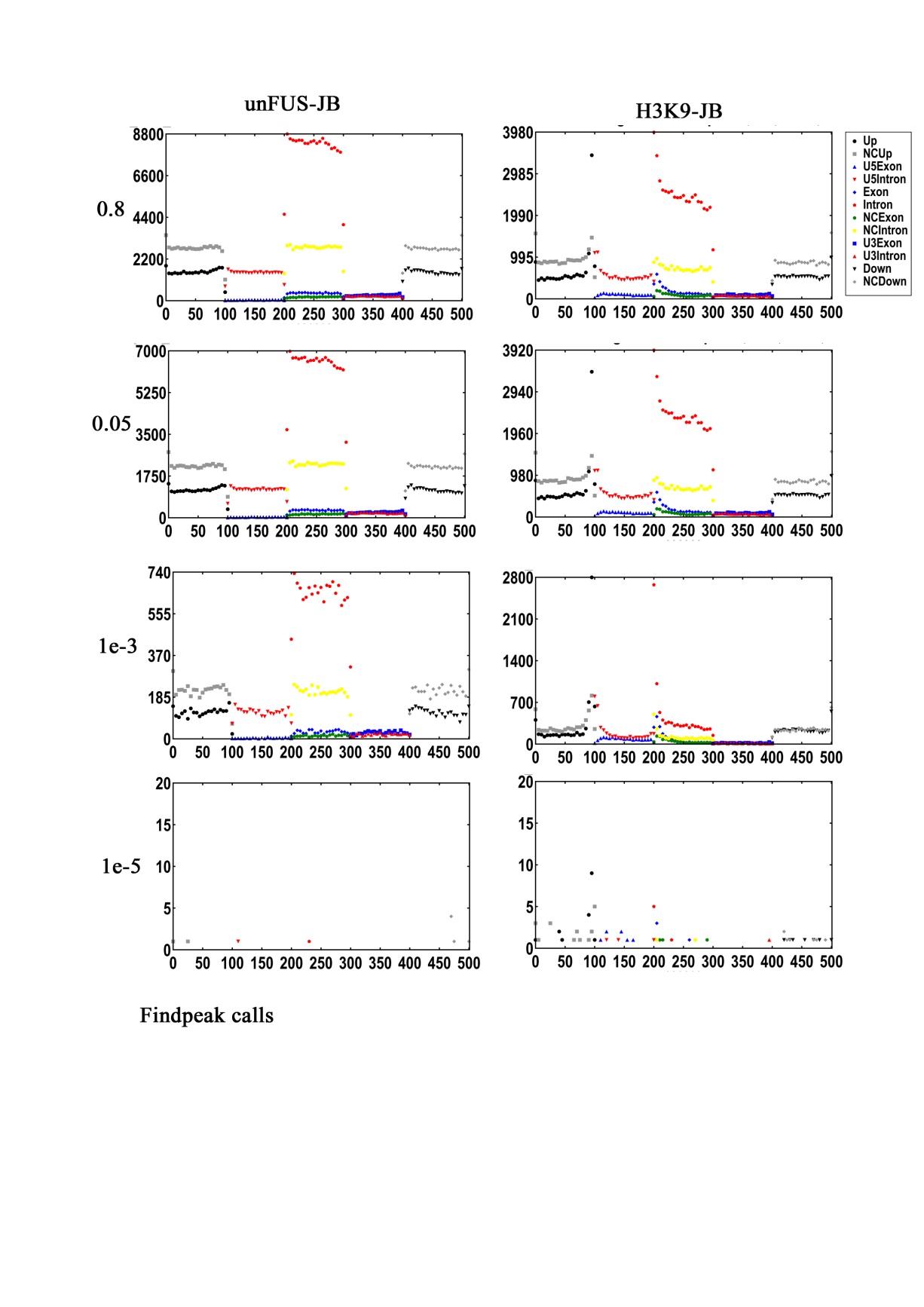
 **
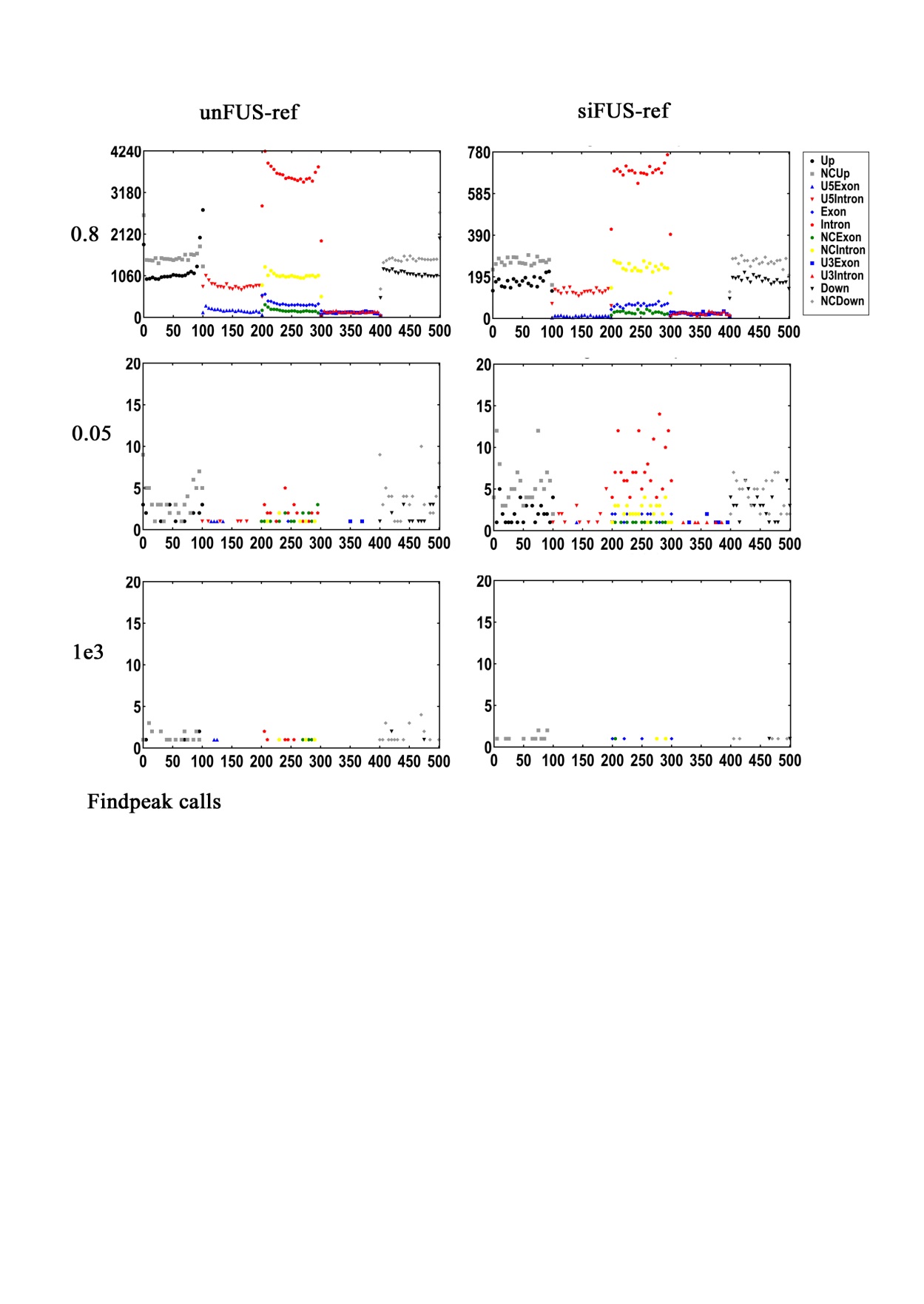
**
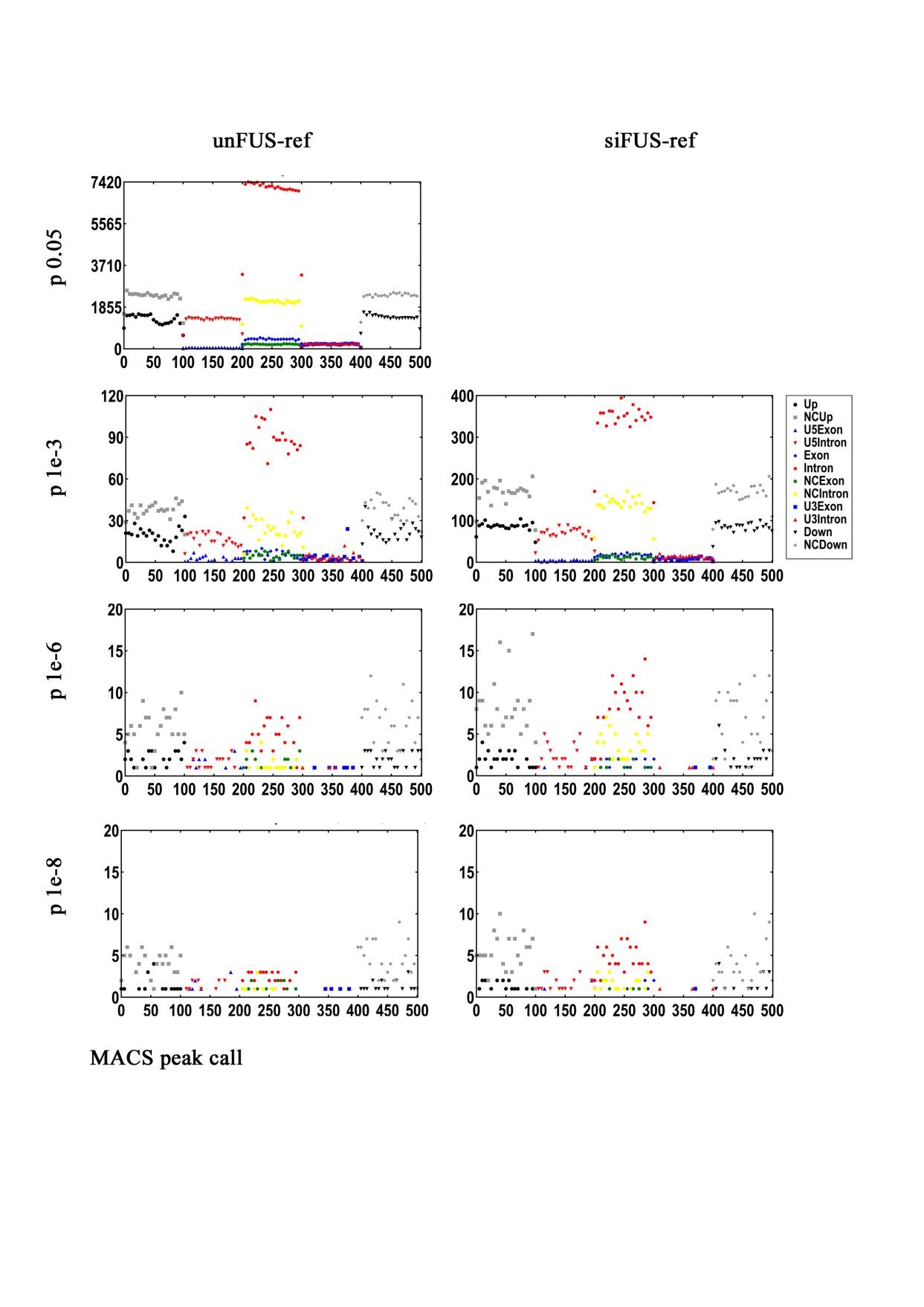


**# of hits**

**# of hits**

**# of hits**

**# of hits**

**position of hits in gene model**

**position of hits in gene model**
