# Supplementary material for: EWS and FUS bind a subset of transcribed genes encoding proteins enriched in RNA regulatory functions
Source: BMC Genomics. 2015 Nov 14;16:929. doi: 10.1186/s12864-015-2125-9 (PMC4647676; doi:10.1186/s12864-015-2125-9)
Supplement: Additional file 18: — Genes co-identified in the hereby presented ChIP-seq data and RNA-CLIP from Hoell et al. [26]. (DOCX 37 kb) [file 12864_2015_2125_MOESM18_ESM.docx]

| **Additional file 18.** Comparison of genes identified by ChIP-seq peaks in this study and in RNA-CLIP by Hoell et al. | | | | | | | | | | | | |
| --- | --- | --- | --- | --- | --- | --- | --- | --- | --- | --- | --- | --- |
| **FUS ChIP-seq v FUS CLIP** | | |  |  |  |  |  |  |  |  |  |  |
| **Sample** | **Ch.** | **peak start** | **peak end** | **length** | **tags** | **p-value** | **F.C.** | **FDR** | **ENS ID** | **Gene name** | **location** | **T** |
| FUS | 1 | 6.8E+07 | 6.8E+07 | 691 | 18 | 84.49 | 9.17 | 100 | ENSG00000116717 | GADD45A | Down | 4 |
| FUS | 9 | 1.9E+07 | 1.9E+07 | 1232 | 38 | 148.76 | 9.38 | 100 | ENSG00000137154 | RPS6 | Down | 5 |
| FUS | 1 | 1.7E+08 | 1.7E+08 | 1673 | 47 | 251.36 | 11.54 | 100 | ENSG00000234741 | GAS5 | Down | 29 |
| FUS | 2 | 8823718 | 8824328 | 611 | 14 | 96.3 | 12.5 | 100 | ENSG00000115738 | ID2 | Exon | 4 |
| FUS | 3 | 1.9E+08 | 1.9E+08 | 596 | 14 | 91.11 | 12.5 | 100 | ENSG00000156976 | EIF4A2 | Down | 28 |
| FUS | 13 | 9.2E+07 | 9.2E+07 | 2989 | 72 | 179.39 | 6.67 | 100 | ENSG00000215417 | MIR17HG | Down | 1 |
| FUS | 13 | 9.2E+07 | 9.2E+07 | 1954 | 55 | 125.65 | 6.66 | 100 | ENSG00000215417 | MIR17HG | Down | 1 |
| FUS | 13 | 9.2E+07 | 9.2E+07 | 2877 | 82 | 211.85 | 6.37 | 100 | ENSG00000215417 | MIR17HG | Down | 1 |
| FUS | 19 | 5.1E+07 | 5.1E+07 | 837 | 14 | 90.31 | 12.14 | 100 | ENSG00000167747 | C19orf48 | Down | 2 |
| FUS | 19 | 5.1E+07 | 5.1E+07 | 1001 | 20 | 139.43 | 12.14 | 100 | ENSG00000167747 | C19orf48 | Down | 2 |
| FUS | 4 | 8.3E+07 | 8.3E+07 | 1671 | 37 | 155.59 | 7.43 | 100 | ENSG00000152795 | HNRPDL | Exon | 5 |
| FUS | 2 | 1.8E+08 | 1.8E+08 | 1785 | 33 | 154.27 | 7.95 | 100 | ENSG00000170144 | HNRNPA3 | Down | 8 |
| FUS | 17 | 7482412 | 7483137 | 726 | 13 | 86 | 12.9 | 100 | ENSG00000129255 | MPDU1 | Up | 5 |
| FUS | 1 | 7.6E+07 | 7.6E+07 | 1524 | 39 | 179.54 | 9.48 | 100 | ENSG00000137955 | RABGGTB | Down | 16 |
| FUS | 5 | 4.1E+07 | 4.1E+07 | 1760 | 34 | 108.99 | 8 | 100 | ENSG00000145592 | RPL37 | Exon | 6 |
| FUS | 5 | 1.8E+08 | 1.8E+08 | 1656 | 49 | 202.06 | 10.35 | 100 | ENSG00000204628 | GNB2L1 | Down | 35 |
| FUS | 7 | 2.6E+07 | 2.6E+07 | 2147 | 51 | 201.43 | 9.17 | 100 | ENSG00000122566 | HNRNPA2B1 | Down | 8 |
| FUS | 7 | 2.6E+07 | 2.6E+07 | 3011 | 95 | 504.94 | 15.18 | 100 | ENSG00000122566 | HNRNPA2B1 | Down | 8 |
| FUS | 9 | 8.7E+07 | 8.7E+07 | 722 | 16 | 91.77 | 9.52 | 100 | ENSG00000165119 | HNRNPK | Down | 15 |
| FUS | 19 | 4.4E+07 | 4.4E+07 | 4008 | 167 | 605.17 | 11.82 | 100 | ENSG00000243137 | PSG4 | Down | 6 |
| FUS | 16 | 7.4E+07 | 7.4E+07 | 692 | 13 | 88.91 | 9.68 | 100 | ENSG00000103035 | PSMD7 | Exon | 2 |
| FUS | 3 | 1.9E+08 | 1.9E+08 | 596 | 14 | 91.11 | 12.5 | 100 | ENSG00000163918 | RFC4 | Exon | 14 |
| FUS | 9 | 8.7E+07 | 8.7E+07 | 722 | 16 | 91.77 | 9.52 | 100 | ENSG00000165118 | C9orf64 | Up | 3 |
| FUS | 2 | 1E+08 | 1E+08 | 1037 | 18 | 84.45 | 7.5 | 100 | ENSG00000071082 | RPL31 | Intron | 11 |
| FUS | 1 | 1.7E+08 | 1.7E+08 | 1673 | 47 | 251.36 | 11.54 | 100 | ENSG00000185278 | ZBTB37 | Up | 7 |
| FUS | 14 | 3.5E+07 | 3.5E+07 | 1015 | 18 | 86.05 | 7.5 | 100 | ENSG00000165389 | C14orf147 | Intron | 1 |
| FUS | 4 | 8.3E+07 | 8.3E+07 | 1671 | 37 | 155.59 | 7.43 | 100 | ENSG00000145293 | ENOPH1 | Up | 4 |
| FUS | 1 | 2.9E+07 | 2.9E+07 | 1523 | 77 | 409.11 | 15 | 100 | ENSG00000180198 | RCC1 | Intron | 12 |
| FUS | 1 | 2.9E+07 | 2.9E+07 | 1267 | 36 | 150.89 | 7.14 | 100 | ENSG00000180198 | RCC1 | Intron | 12 |
| FUS | 1 | 1.7E+08 | 1.7E+08 | 1673 | 47 | 251.36 | 11.54 | 100 | ENSG00000117593 | DARS2 | Down | 4 |
| FUS | 1 | 7.8E+07 | 7.8E+07 | 1856 | 41 | 107.89 | 6.1 | 100 | ENSG00000162613 | FUBP1 | Down | 16 |
| FUS | 9 | 1.9E+07 | 1.9E+07 | 1232 | 38 | 148.76 | 9.38 | 100 | ENSG00000137145 | DENND4C | Exon | 10 |
| FUS | 1 | 2.9E+07 | 2.9E+07 | 1523 | 77 | 409.11 | 15 | 100 | ENSG00000204138 | PHACTR4 | Down | 5 |
| FUS | 2 | 1.8E+08 | 1.8E+08 | 1785 | 33 | 154.27 | 7.95 | 100 | ENSG00000116044 | NFE2L2 | Down | 12 |
| FUS | 13 | 7.6E+07 | 7.6E+07 | 1139 | 27 | 85.31 | 6.88 | 100 | ENSG00000136111 | TBC1D4 | Intron | 8 |
| FUS | 11 | 7.3E+07 | 7.3E+07 | 520 | 13 | 85.01 | 12.5 | 100 | ENSG00000137478 | FCHSD2 | Intron | 10 |
| FUS | 13 | 7.4E+07 | 7.4E+07 | 1121 | 24 | 82.35 | 7.58 | 100 | ENSG00000118922 | KLF12 | Intron | 4 |
|  |  |  |  |  |  |  |  |  |  |  |  |  |
| **EWS ChIP-seq v EWS CLIP** | | |  |  |  |  |  |  |  |  |  |  |
| **Sample** | **Ch.** | **peak start** | **peak end** | **length** | **tags** | **p-value** | **F.C.** | **FDR** | **ENS ID** | **Gene name** | **location** | **T** |
| EWS | 19 | 5696678 | 5697903 | 1226 | 20 | 127.85 | 11.92 | 100 | ENSG00000130255 | RPL36 | Down | 2 |
| EWS | 16 | 2010376 | 2011489 | 1114 | 22 | 88.6 | 8 | 64.4 | ENSG00000140988 | RPS2 | Down | 17 |
| EWS | 17 | 7.5E+07 | 7.5E+07 | 1001 | 19 | 100.74 | 9.46 | 75.3 | ENSG00000161547 | SRSF2 | Down | 5 |
| EWS | 17 | 7.5E+07 | 7.5E+07 | 1140 | 21 | 80.16 | 7 | 56.1 | ENSG00000161547 | SRSF2 | Down | 5 |
| EWS | 8 | 3.8E+07 | 3.8E+07 | 1734 | 36 | 84.4 | 6.19 | 62 | ENSG00000183779 | ZNF703 | Down | 2 |
| EWS | 9 | 1.9E+07 | 1.9E+07 | 1298 | 35 | 116.41 | 6.25 | 89.6 | ENSG00000137154 | RPS6 | Down | 5 |
| EWS | 1 | 1.7E+08 | 1.7E+08 | 1261 | 34 | 156.97 | 10.34 | 100 | ENSG00000234741 | GAS5 | Down | 29 |
| EWS | 2 | 8.6E+07 | 8.6E+07 | 1050 | 18 | 87.16 | 7.88 | 64.4 | ENSG00000168906 | MAT2A | Down | 7 |
| EWS | 2 | 8.6E+07 | 8.6E+07 | 1036 | 21 | 87.99 | 8 | 63.7 | ENSG00000168906 | MAT2A | Down | 7 |
| EWS | 3 | 1.9E+08 | 1.9E+08 | 980 | 27 | 180.95 | 17.57 | 100 | ENSG00000156976 | EIF4A2 | Down | 28 |
| EWS | 13 | 9.2E+07 | 9.2E+07 | 5252 | 144 | 294.99 | 6.36 | 100 | ENSG00000215417 | MIR17HG | Down | 1 |
| EWS | 19 | 5.1E+07 | 5.1E+07 | 1774 | 45 | 266.73 | 20 | 100 | ENSG00000167747 | C19orf48 | Down | 2 |
| EWS | 3 | 1.3E+07 | 1.3E+07 | 921 | 17 | 84 | 10.98 | 60.3 | ENSG00000144713 | RPL32 | Exon | 8 |
| EWS | 3 | 2.4E+07 | 2.4E+07 | 1425 | 29 | 111.34 | 9.43 | 87.3 | ENSG00000174748 | RPL15 | Intron | 14 |
| EWS | 6 | 7.4E+07 | 7.4E+07 | 1322 | 34 | 108.22 | 8.75 | 84.1 | ENSG00000156508 | EEF1A1 | Down | 12 |
| EWS | 2 | 1.8E+08 | 1.8E+08 | 3253 | 92 | 154.27 | 7.31 | 100 | ENSG00000170144 | HNRNPA3 | Down | 8 |
| EWS | 1 | 7.6E+07 | 7.6E+07 | 2217 | 73 | 285.41 | 8.12 | 100 | ENSG00000137955 | RABGGTB | Down | 16 |
| EWS | 22 | 3.9E+07 | 3.9E+07 | 1211 | 25 | 90.05 | 7.5 | 67 | ENSG00000183741 | CBX6 | Down | 3 |
| EWS | 5 | 1.8E+08 | 1.8E+08 | 2070 | 55 | 203.32 | 8.57 | 100 | ENSG00000204628 | GNB2L1 | Down | 35 |
| EWS | 7 | 2.6E+07 | 2.6E+07 | 4900 | 178 | 968.63 | 20.34 | 100 | ENSG00000122566 | HNRNPA2B1 | Down | 8 |
| EWS | 7 | 2.6E+07 | 2.6E+07 | 742 | 24 | 131.86 | 9.17 | 100 | ENSG00000122566 | HNRNPA2B1 | Down | 8 |
| EWS | 7 | 2.6E+07 | 2.6E+07 | 1155 | 26 | 85.75 | 6.43 | 63.3 | ENSG00000122566 | HNRNPA2B1 | Down | 8 |
| EWS | 9 | 8.7E+07 | 8.7E+07 | 930 | 22 | 120.23 | 13.1 | 100 | ENSG00000165119 | HNRNPK | Down | 15 |
| EWS | 17 | 8076101 | 8076911 | 811 | 15 | 100.02 | 13.63 | 73.3 | ENSG00000220205 | VAMP2 | Up | 5 |
| EWS | 19 | 4.4E+07 | 4.4E+07 | 2371 | 126 | 519.09 | 13.75 | 100 | ENSG00000243137 | PSG4 | Down | 6 |
| EWS | 19 | 4.4E+07 | 4.4E+07 | 1604 | 95 | 508.57 | 13 | 100 | ENSG00000243137 | PSG4 | Down | 6 |
| EWS | 1 | 2.5E+08 | 2.5E+08 | 3824 | 114 | 158.54 | 5.67 | 100 | ENSG00000153187 | HNRNPU | Down | 9 |
| EWS | 1 | 2.5E+08 | 2.5E+08 | 2487 | 56 | 109.04 | 5.67 | 86.4 | ENSG00000153187 | HNRNPU | Down | 9 |
| EWS | 9 | 3.3E+07 | 3.3E+07 | 1235 | 25 | 108.9 | 8.23 | 85.3 | ENSG00000086061 | DNAJA1 | Down | 5 |
| EWS | 1 | 2.5E+08 | 2.5E+08 | 2487 | 56 | 109.04 | 5.67 | 86.4 | ENSG00000188206 | NCRNA00201 | Exon | 3 |
| EWS | 1 | 2.5E+08 | 2.5E+08 | 3824 | 114 | 158.54 | 5.67 | 100 | ENSG00000188206 | NCRNA00201 | Intron | 3 |
| EWS | 3 | 1.9E+08 | 1.9E+08 | 980 | 27 | 180.95 | 17.57 | 100 | ENSG00000163918 | RFC4 | Intron | 14 |
| EWS | 10 | 1E+08 | 1E+08 | 719 | 18 | 95.23 | 10 | 69.9 | ENSG00000099194 | SCD | Exon | 2 |
| EWS | 3 | 1.9E+08 | 1.9E+08 | 1123 | 20 | 96.88 | 9.21 | 71.6 | ENSG00000136527 | TRA2B | Down | 16 |
| EWS | 1 | 6.8E+07 | 6.8E+07 | 1193 | 28 | 83.2 | 6.41 | 59 | ENSG00000142864 | SERBP1 | Exon | 8 |
| EWS | 11 | 6.5E+07 | 6.5E+07 | 1364 | 25 | 96.18 | 7 | 70.7 | ENSG00000245532 | NEAT1 | Exon | 2 |
| EWS | 21 | 4.4E+07 | 4.4E+07 | 704 | 20 | 97.72 | 10 | 75.3 | ENSG00000160200 | CBS | Down | 20 |
| EWS | 5 | 1.7E+08 | 1.7E+08 | 1372 | 24 | 115.83 | 9.21 | 84.3 | ENSG00000181163 | NPM1 | Intron | 12 |
| EWS | 22 | 3.9E+07 | 3.9E+07 | 1598 | 41 | 137.79 | 6.66 | 100 | ENSG00000100201 | DDX17 | Down | 14 |
| EWS | 4 | 8.3E+07 | 8.3E+07 | 1303 | 30 | 142.71 | 9 | 100 | ENSG00000145293 | ENOPH1 | Up | 4 |
| EWS | X | 4.1E+07 | 4.1E+07 | 1473 | 44 | 123.23 | 6.5 | 100 | ENSG00000215301 | DDX3X | Intron | 7 |
| EWS | 3 | 1.7E+08 | 1.7E+08 | 826 | 15 | 86.92 | 15.71 | 65.7 | ENSG00000008952 | SEC62 | Exon | 12 |
| EWS | 6 | 1.4E+08 | 1.4E+08 | 1242 | 30 | 107.98 | 7.14 | 82.8 | ENSG00000029363 | BCLAF1 | Down | 23 |
| EWS | 6 | 1.4E+08 | 1.4E+08 | 1227 | 31 | 86.63 | 7.22 | 65.1 | ENSG00000029363 | BCLAF1 | Down | 23 |
| EWS | 1 | 2.9E+07 | 2.9E+07 | 2270 | 110 | 495.44 | 17 | 100 | ENSG00000180198 | RCC1 | Intron | 12 |
| EWS | 1 | 2.9E+07 | 2.9E+07 | 1371 | 38 | 132.6 | 8.72 | 100 | ENSG00000180198 | RCC1 | Intron | 12 |
| EWS | 9 | 3.3E+07 | 3.3E+07 | 1235 | 25 | 108.9 | 8.23 | 85.3 | ENSG00000122692 | SMU1 | Down | 2 |
| EWS | 20 | 3.4E+07 | 3.4E+07 | 613 | 14 | 83.93 | 11.84 | 60.7 | ENSG00000131051 | RBM39 | Down | 45 |
| EWS | 12 | 7.6E+07 | 7.6E+07 | 1182 | 25 | 108.43 | 9.8 | 83.9 | ENSG00000187109 | NAP1L1 | Exon | 7 |
| EWS | 12 | 5.5E+07 | 5.5E+07 | 1207 | 31 | 117.63 | 9.29 | 93.3 | ENSG00000094916 | CBX5 | Up | 2 |
| EWS | 1 | 2.3E+08 | 2.3E+08 | 1688 | 32 | 141.7 | 7.61 | 100 | ENSG00000143740 | SNAP47 | Up | 11 |
| EWS | 7 | 1.3E+08 | 1.3E+08 | 441 | 12 | 92.47 | 13.64 | 68.1 | ENSG00000128607 | KLHDC10 | Intron | 4 |
| EWS | 9 | 3.8E+07 | 3.8E+07 | 728 | 15 | 91.64 | 13.51 | 66 | ENSG00000122741 | DCAF10 | Intron | 5 |
| EWS | 1 | 2.2E+08 | 2.2E+08 | 955 | 24 | 102.02 | 7.16 | 78.6 | ENSG00000136628 | EPRS | Down | 7 |
| EWS | 9 | 1.9E+07 | 1.9E+07 | 1298 | 35 | 116.41 | 6.25 | 89.6 | ENSG00000137145 | DENND4C | Exon | 10 |
| EWS | 1 | 7.6E+07 | 7.6E+07 | 2217 | 73 | 285.41 | 8.12 | 100 | ENSG00000057468 | MSH4 | Intron | 1 |
| EWS | 1 | 2.9E+07 | 2.9E+07 | 2270 | 110 | 495.44 | 17 | 100 | ENSG00000204138 | PHACTR4 | Down | 5 |
| EWS | 15 | 6.6E+07 | 6.6E+07 | 801 | 19 | 94.51 | 11 | 66.7 | ENSG00000174485 | DENND4A | Intron | 3 |
| EWS | 17 | 5.7E+07 | 5.7E+07 | 1103 | 35 | 156.68 | 13.57 | 100 | ENSG00000121101 | TEX14 | Intron | 3 |
| EWS | 17 | 5.7E+07 | 5.7E+07 | 810 | 17 | 116.76 | 13.33 | 93.5 | ENSG00000121101 | TEX14 | Intron | 3 |
| EWS | 2 | 1.8E+08 | 1.8E+08 | 3253 | 92 | 154.27 | 7.31 | 100 | ENSG00000116044 | NFE2L2 | Down | 12 |
| EWS | 12 | 2.1E+07 | 2.1E+07 | 735 | 16 | 105.55 | 17.65 | 80.6 | ENSG00000172572 | PDE3A | Intron | 3 |
| EWS | 4 | 2E+07 | 2E+07 | 960 | 24 | 137.3 | 11.36 | 100 | ENSG00000145147 | SLIT2 | Intron | 12 |
| EWS | 6 | 7.3E+07 | 7.3E+07 | 739 | 14 | 81.19 | 10.61 | 57.9 | ENSG00000079841 | RIMS1 | Intron | 26 |
| EWS | 15 | 6.1E+07 | 6.1E+07 | 715 | 17 | 128.12 | 20.44 | 100 | ENSG00000069667 | RORA | Intron | 4 |
| EWS | X | 9.6E+07 | 9.6E+07 | 1347 | 23 | 100.76 | 9.52 | 76.4 | ENSG00000147202 | DIAPH2 | Intron | 6 |
| EWS | 10 | 5.3E+07 | 5.3E+07 | 1005 | 25 | 93.16 | 7.86 | 70.5 | ENSG00000185532 | PRKG1 | Intron | 6 |
| EWS | 1 | 2.3E+08 | 2.3E+08 | 1743 | 42 | 120.75 | 7.5 | 100 | ENSG00000143799 | PARP1 | Intron | 11 |
