# Supplementary material for: EWS and FUS bind a subset of transcribed genes encoding proteins enriched in RNA regulatory functions
Source: BMC Genomics. 2015 Nov 14;16:929. doi: 10.1186/s12864-015-2125-9 (PMC4647676; doi:10.1186/s12864-015-2125-9)
Supplement: Additional file 19: — List of primer sequences for PCR. (DOCX 20 kb) [file 12864_2015_2125_MOESM19_ESM.docx]

| **Additional file 19.** Primer sequences | | | | | | |
| --- | --- | --- | --- | --- | --- | --- |
| Gene name | method | Additional information | Forward primer  5’ to 3’ | | Reverse primer  5’ to 3’ | |
| *C19ORF48/ACPT* | qPCR | ChIP |  | AAAGCAAGGGGTGATGTGTC |  | ACCTCCTCCCTCCTGTCATT |
| *RCC1/SNHG3* | qPCR | ChIP |  | GAGTTACTCAGCCCCTCTGC |  | ATGCTGCTGCACTCCATCTA |
| *HNRNPK/MIR7* | qPCR | ChIP |  | TAAGCCAGGGATTTTTGTGC |  | GATCAGGCTGACACTGCTGA |
| *IFRG28* | qPCR | ChIP control |  | GCTCTTTATCTCTCTCTCAGCAAG |  | CAGTTTCGGTGTTCGGTTCA |
| *FUS* | RT-qPCR | expression |  | CCCTACGGACAGCAGAGTTA |  | CTGGGGAGTTGACTGAGTTC |
| *EWS* | RT-qPCR | expression |  | CTACAGCCAAGCTCCAAGTC |  | CCCATAAACACCCATGCTAC |
| *TAF15* | RT-qPCR | expression |  | GGGAGCACAGTGTCATTT |  | ATTTCCGCATGACGGATTAG |
| *Pri-MIR7-1* | RT-qPCR | expression |  | GCCATGGTGTCTCAACCTTT |  | GGTTAAGGCTTCTTCCAGCA |
| *Pre-MIR-7-1* | RT-qPCR | expression |  | GTTGGCCTAGTTCTGTGTGG |  | CAGACTGTGATTTGTTGTCGAT |
| *Pre-MIR-7-1** | cDNA | expression |  |  |  | CCTGTGCCATATGGCAGACT |
| *RNU48* | RT-qPCR | expression |  | GTGATGATGACCCCAGGTAAC |  | GTGATGGCATCAGCGACAC |
| *RNU48** | cDNA | expression |  |  |  | GGTCAGAGCGCTGCGGTGAT |
| *HNRNPK* | RT-qPCR | expression | E1A | CAGACGCCATTATCCTCTGT | E2 | TATTAAACGGGCACACCAAT |
| *HNRNPK* | RT-qPCR | expression | E1B | CTGCGCTCGTTTTCTGTCTA | E2 | TATTAAACGGGCACACCAAT |
| *HNRNPK* | RT-qPCR | expression | E3F | GCCAGAAGAAACCTTCCCTA | I3R | CCACACACTCCTAAGGCAAT |
| *HNRNPK* | RT-qPCR | expression | E9F | ACTTTGACTGCGAGTTGAGG | E10R | AAGCTTGATGGTGGTTTGAG |
| *HNRNPK* | RT-qPCR | expression | E10F | ATTGGAGGAAAACCCGATAG | I10R | GCTCTGAAGCTACTTTTGCAG |
| *HNRNPK* | RT-qPCR | expression | I10F | CCTTTGAGCCTTTGAATGAA | I10R | CTGAGGCACCTGAGATAGGA |
| *HNRNPK* | RT-qPCR | expression | I13F | GGCTCCGGATATGGTAAGTT | E14R | TAGGTCCACCAAGATCACCA |
| *HNRNPK* | RT-qPCR | expression | I14F | TGACAGGTTTAGGGAGCGTTA | E15R | CCTTTGCCAATAATAGATCCAG |
| *HNRNPK* | RT-qPCR | expression | E15F | GCCTTTAGAAGGATCCGAAG | E16R | CAACATCTGCATACTGCTTCAC |
| *HNRNPK* | RT-qPCR | expression | E15F | GGACCAGATACAGAATGCACA | I15R | CCAAAAGGTTGAGACACCAT |
| *HNRNPK* | RT-qPCR | expression | I15F | CAACCTTTTGGGGACCTAAC | I15R | GCTTGGTTAAGGCTTCTTCC |
| *HNRNPK* | RT-PCR | alt. splice. | E15F | GCCTTTAGAAGGATCCGAAG | E16RS | CCCCAAATGTTACAGTGACC |
| *ACPT* | RT-qPCR | expression | E8 | TCCTGCTGAATGCTATCCTTG | E10 | AGAAGAGGGAGACGGTGACA |
| *C19ORF48* | RT-qPCR | expression | E2 | TGTTAGCTTGGCCTCTGTCC | E5 | GGCAGGAAACAGCAGAGTGT |
| *C19ORF48* | RT-qPCR | expression | E1/3 | AGAGAGAAATGCTGGGGTGC | E5 | GGCAGGAAACAGCAGAGTGT |
| *C19ORF48* | RT-qPCR | expression | I3 | AGAGGCGATTGAGGGGTATC | I3 | CACATCTGTGGGGTTCAGTG |
| *C19ORF48* | RT-qPCR | expression | I4 | AGGTGATGGTGGGGGTGT | E5 | GGCAGGAAACAGCAGAGTGT |
| *SNORD88A* | RT-qPCR | expression |  | CCTCCATGATGTCCAGCAC |  | GCACCGTGTCCTCAGTGG |
| *SNORD88B* | RT-qPCR | expression |  | CAGCACTGGGCTCTGACTG |  | AGAACCCCGGATGTCAAAG |
| *Snord88C* | RT-qPCR | expression |  | TCCCATGATGTCCAGCACT |  | AGACCCCCAGGTGTCAAAG |
| *RCC1* | RT-qPCR | expression | E1/2 | TCGCTTCTTCTCCTTGGATT | E4 | CCCTGGGATCTCTGATCTCTC |
| *RCC1* | RT-qPCR | expression | E2/3 | GATCTGCACTTCGCATTTTG | E5/6 | GTTCTGTGCTGTGGGACCT |
| *RCC1* | RT-qPCR | expression | E4 | TCCAAAAGAGGAGTCCATGA | E6/7 | GTTCTGTGCTGTGGGACCT |
| *RCC1* | RT-qPCR | expression | E6/7 | AAAGTGGAGCTGCAAGAGAA | E8/11 | AACAGTCCAATCACACCGTTA |
| *RCC1* | RT-qPCR | expression | E1A | CCTTGTGGCCGACGTGCAC | E3/4A | GTTCTGTGCTGTGGGACCT |
| *SNHG3* | RT-qPCR | expression | S4 | TTCTAAAGGCCCTGAAACCT | S4 | CTTGCACTCAACCCAGACTT |
| *SNHG3* | RT-qPCR | expression | I2 | CCAGGCCGAAGATTTTCATA | I2 | AGGTACCGCAGAAGCAAAGA |
| *SNORA73A* | RT-qPCR | expression |  | CTGCCCCATGATGTACAAGT |  | CAGGACTCTGGGAAGCTGTA |
| *SNORA73B* | RT-qPCR | expression |  | TCCACAACGTTGAAGATGAA |  | TTTGTCTCCCCAGTCATTGT |
| *GAPDH* | RT-qPCR | normalisator |  | ATGGGGAAGGTGAAGGTCGGAG |  | GATGACAAGCTTCCCGTTCTCAGC |
| *TBP* | RT-qPCR | normalisator |  | TGCACAGGAGCCAAGAGTGAA |  | CACATCACAGCTCCCCACCA |
| *UBC* | RT-qPCR | normalisator |  | CCTGGTGCTCCGTCTTAGAG |  | TTTCCCAGCAAAGATCAACC |

The position of the primers is illustrated in additional file material. If relevant the exon (E) and intron (I) number positions are shown in the table and represent exon and intron numbers derived from individual transcript isoforms. * Gene specific reverse primers used only for cDNA synthesis.
